# Azine-N-oxides as effective controlling groups for Rh-catalysed intermolecular alkyne hydroacylation†

**DOI:** 10.1039/d1sc03915f

**Published:** 2021-09-14

**Authors:** Daniel F. Moseley, Jagadeesh Kalepu, Michael C. Willis

**Affiliations:** Chemistry Research Laboratory, University of Oxford Mansfield Road Oxford OX1 3TA UK michael.willis@chem.ox.ac.uk

## Abstract

Heterocycle-derived aldehydes are challenging substrates in metal-catalysed hydroacylation chemistry. We show that by using azine N-oxide substituted aldehydes, good reactivity can be achieved, and that they are highly effective substrates for the intermolecular hydroacylation of alkynes. Employing a Rh(i)-catalyst, we achieve a mild and scalable aldehyde C–H activation, that permits the coupling with unactivated terminal alkynes, in good yields and with high regioselectivities (up to >20 : 1 l:b). Both substrates can tolerate a broad variety of functional groups. The reaction can also be applied to diazine aldehydes that contain a free N-lone pair. We demonstrate conversion of the hydroacylation products to the corresponding azine, through a one-pot hydroacylation/deoxygenation sequence. A one-pot hydroacylation/cyclisation, using N-Boc propargylamine, additionally leads to the synthesis of a bidentate pyrrolyl ligand.

## Abstract 2

Heterocycle-derived aldehydes are challenging substrates in metal-catalysed hydroacylation chemistry; using the N-oxide derivatives allows efficient reactions to be achieved.

## Graphical abstract

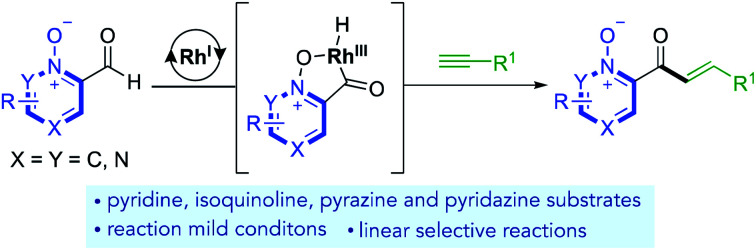

## Introduction

**Fig. 1:**
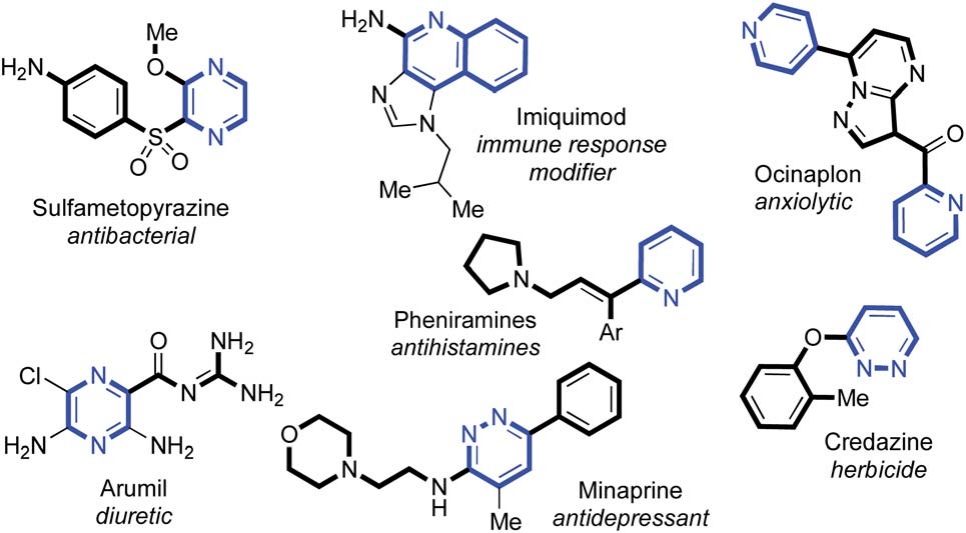
A selection of C(2)-substituted azines present in biologically important compounds.

C(2)-Substituted azines are becoming increasingly prevalent in a wide selection of pharmaceuticals and agrochemicals^1^ (Fig. 1). Incorporation of azines and other N-heterocyclic motifs into drug candidates can lead to a plethora of benefits, such as adjusted target specificity/potency, lipophilicity and aqueous solubility.^2^ Thus, their controlled functionalisation is of paramount importance in medicinal chemistry.

**Scheme 1:**
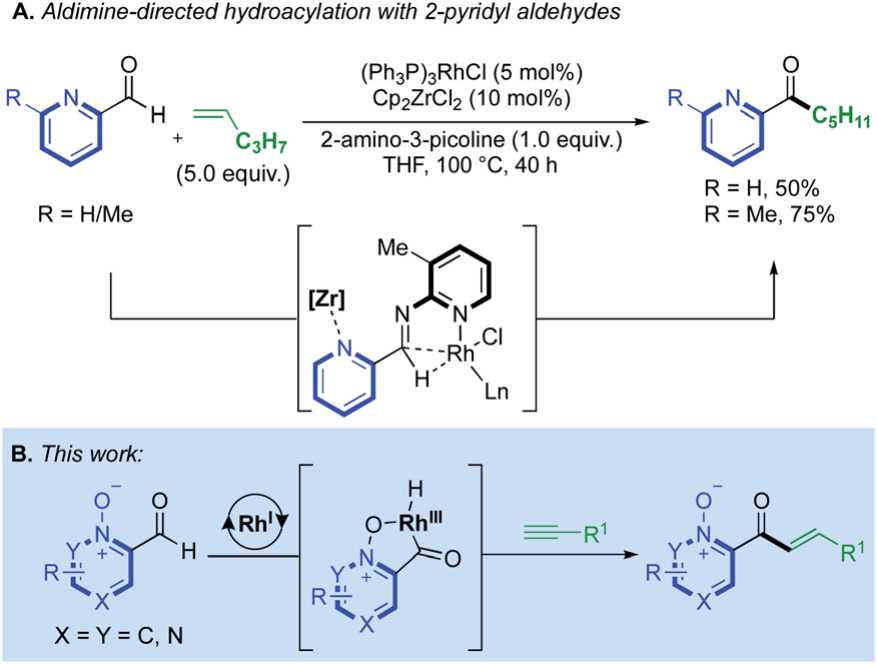
(A) Challenges faced with pyridyl groups in intermolecular hydroacylation; (B) azine-*N*-oxides as a solution to this compromised reactivity.

The utility of N-oxides has propelled the field of catalytic azine C–H functionalisation.^3^ From an atom-economy perspective, N-oxides are effective directing groups as their removal comprises the loss of a single ‘O’ atom in a straightforward redox process.^4^ This can sometimes be incorporated into a reaction's catalytic cycle, either through direct deoxygenation^5^ or O-atom transfer,^6^ thus relinquishing the need for external oxidants or subsequent reduction steps. A wealth of catalytic reactions exploit the enhanced reactivity that the N-oxide provides to the C(2)-position, priming the azine substrate for C–H bond cleavage.^7^ A variety of Pd^II^/Ag^I^/Ni^0^/Cu^I/II^/Rh^III^-catalysed couplings, towards C–C,^8,4*b*^ C–O,^9^ C–S^10^ and C–N^11^ functionalised products have been achieved using this strategy. Prior work on azine C(2)-functionalisation has suggested that a metal-coordinated N-oxide species is not necessarily an active catalytic intermediate.^4*b*^ However, a Rh^I^-catalysed alkenylation of quinoline-*N*-oxide by Shibata^12^ was the first method to demonstrate how the N-oxide could formally direct a C–H bond activation, through the generation of a rhodacycle intermediate, and deliver exclusive C(8)-H regioselectivity. This reactivity has been applied to a range of quinoline-*N*-oxide reactions, yielding C–C,^6*a*,*c*,*d*,12,13^ C–N^14^ and C–I^14^ functionalised products using Rh^III^/Ir^III^/Pd^II^ and Co^III^-catalysts. Despite these advances, methods that utilise N-oxides as a formal directing group remain in their infancy. The success of Shibata's chemistry prompted us to consider the use of N-oxides in intermolecular hydroacylation.^15^ Azine aldehydes are challenging substrates in hydroacylation as the pyridyl nitrogen can prevent the formation of chelated rhodacycle intermediates, which are key for reaction progression in many hydroacylation systems.^16,17^ Catalyst inhibition can also be problematic. Jun and Lee previously established an intermolecular alkene hydroacylation with 2-pyridyl aldehydes, *via* the *in situ* formation of an aldimine intermediate (Scheme 1A).^18^ Respectable yields could only be obtained on a single alkene example by hindering the disfavoured N-coordination of the 2-pyridyl group, either through the use of a Zr-additive or with a sterically obstructing *ortho*-methyl group. The utility of N-oxides as directing groups for aldehyde C–H activations in hydroacylation remains unexplored, and would provide a simple and attractive solution towards the challenges faced when using azines in hydroacylation. Herein, we report the first N-oxide directed hydroacylation of unactivated terminal alkynes using a range of azine aldehydes (Scheme 1B).

## Results and discussion

**Scheme 2:**
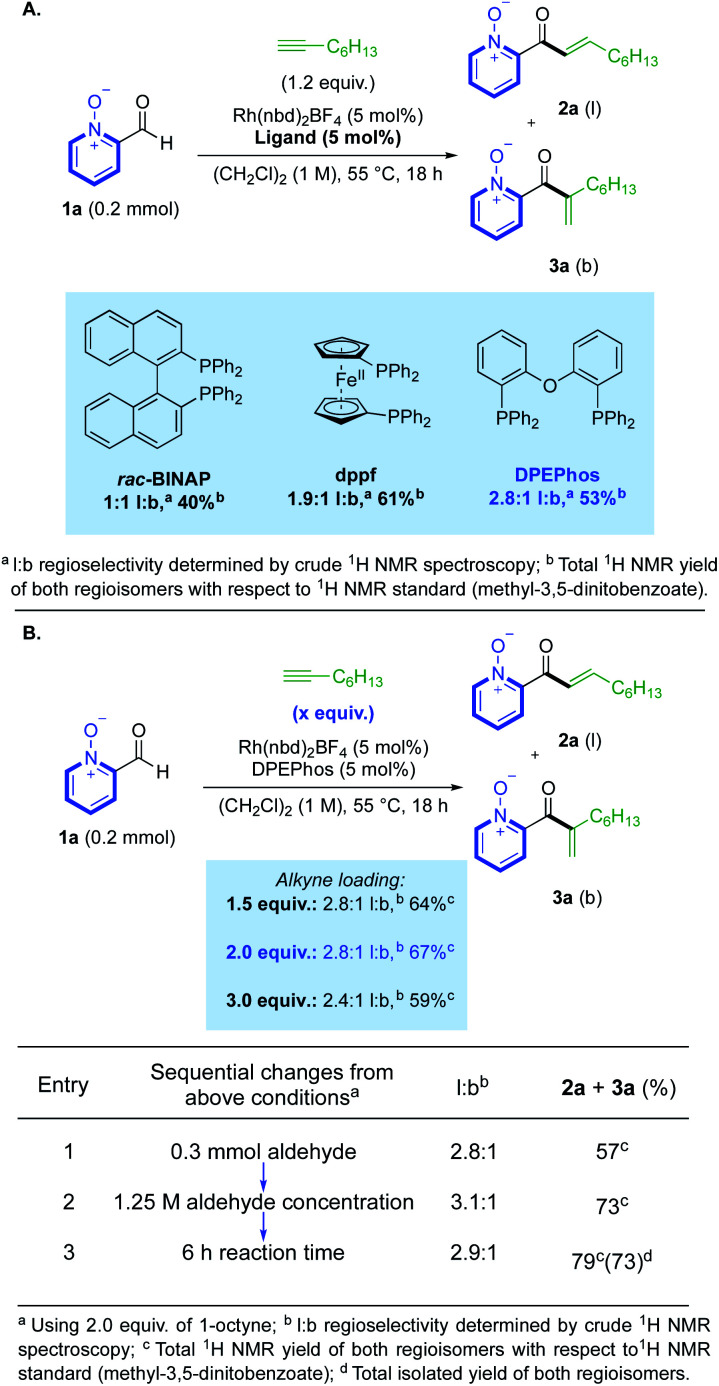
(A) Initial ligand evaluation for reactivity. (B) Selected optimisation of hydroacylation reactivity.

We began our study by investigating the reaction between aldehyde **1a** and 1-octyne using Rh(i) catalysts. Our initial ligand evaluation showed that three ligands could produce promising yields: *rac*-BINAP, dppf and DPEPhos (Scheme 2A). The high yield produced by the dppf-derived catalyst is significant, as this ligand has previously promoted hydroacylation directed by a salicylaldehyde phenolate-anion,^19^ which is a coordinating group *iso*-electronic to the pyridine-*N*-oxide substrate **1a**. We decided to further study the result obtained using the DPEPhos-derived catalyst,^20^ as it delivered the highest linear:branched (l:b) selectivity. Many reactions from the ligand assessment revealed full consumption of aldehyde **1a** after 18 h (see ESI, Section 3†), however, yields remained moderate. Pleasingly, increasing the loading of 1-octyne to 2.0 equivalents made a significant improvement in product yield to 67% (Scheme 2B). From this result, a higher yield could then be obtained by making sequential adjustments in scale, reaction concentration (M), and reaction time (h). We additionally investigated the use of alternative solvents; however, despite our efforts, no other solvent system provided an appropriate balance between yield and selectivity (see ESI, Section 3†). Control reactions established that some decomposition of both the aldehyde substrate, and enone products, was possible under the reaction conditions (see ESI, Section 6†). Rh(iii)-derived catalysts were not effective in the present system, with N–O reduction products dominating.^16*d*^

**Scheme 3:**
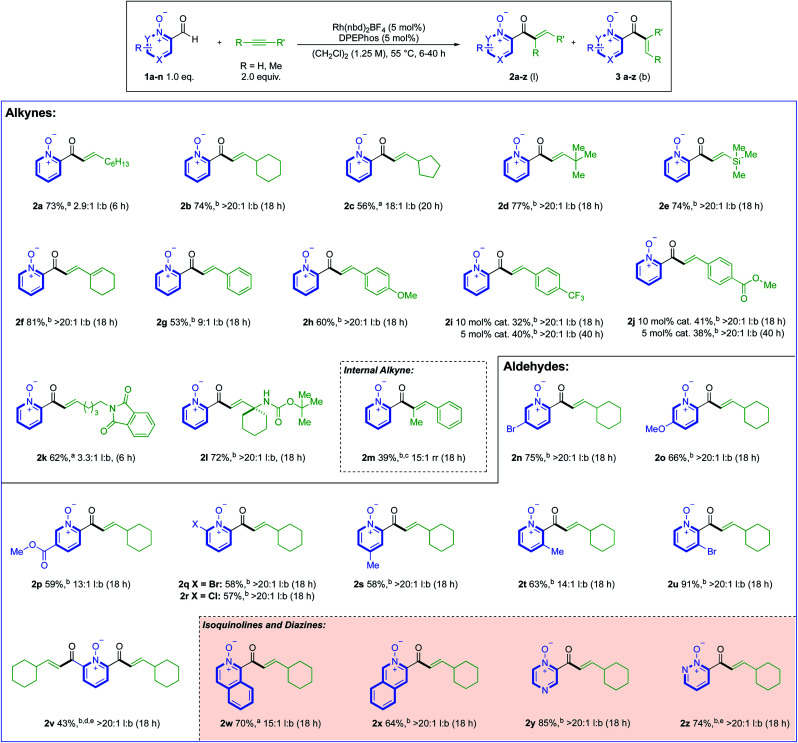
Reaction scope of both alkyne and aldehyde components using 0.3 mmol of aldehyde 1. ^a^Yield corresponds to the combined yield of both regioisomers. ^b^Yield corresponds to the isolated yield of a single regioisomer. ^c^Reaction temperature was 80 °C. ^d^ Using 4.0 equiv. alkyne. ^e^Reactions were performed on a 0.2 mmol scale.

With optimised conditions established, these were then used to explore the reactivity of different alkyne substrates (Scheme 3). Although a return to the longer reaction duration was necessary for more sterically hindered aliphatic alkynes, we were delighted to observe a significant increase in linear regioselectivity, up to >20 : 1 l:b, in examples **2b–d**. TMS-acetylene reacted in a similar fashion to *t*-Bu-acetylene, delivering linear hydroacylation product **2e** in 74% and a >20 : 1 l:b. ratio.

Cyclohexenyl substituted linear product **2f** provided the highest yield of 81%, with >20 : 1 l:b selectivity. Phenylacetylene proved to be a challenging substrate (**2g**); however, we could obtain >20 : 1 l:b selectivity by altering the substrate electronics (**2h–j**). For the electron-poor phenylacetylene substrates, full conversion to products **2i** and **2j** could only be achieved with a higher catalyst loading, or longer reaction duration (40 h). We next evaluated the efficacy of nitrogen-bearing alkynes as substrates, with a phthalimide-tethered alkyne (**2k**), and a cyclohexyl-substituted propargyl amine (**2l**) both working well. Internal alkynes were generally poor substrates, with both 3-octyne, and diphenylacetylene delivering only trace products. However, employing 1-propynylbenzene as substrate and reacting at 80 °C allowed 39% of trisubstituted-enone **2m** to be isolated (with 15 : 1 rr). Alkene substrates were unreactive (see ESI, Section 6†). We then turned our focus towards the scope of the aldehyde component, where ethynylcyclohexane was used as the alkyne coupling partner (Scheme 3). Substituents at the 5-position of the pyridine aldehyde provided excellent reactivity, with 5-bromo (**2n**), 5-methoxy (**2o**), and nicotinate (**2p**) groups providing the expected products in good yields. More sterically encumbered 6-halo-substituted aldehydes could also be used, and delivered highly selective reactions (**2q** and **2r**). 4- and 3-Me-substituted examples **2s** and **2t** were obtained in similar yields to 5- and 6-substituted products, and a 3-bromo-substituted aldehyde gave linear enone **2u** in excellent yield and selectivity. A double-hydroacylation to deliver 2,6-bis-functionalised product **2v**, was achieved in 43%, with >20 : 1 l:b selectivity. The low reaction conversion for this example was attributed to the poor solubility of the 2,6-bis-aldehyde precursor. Moving away from the pyridine core, we found that 3-formyl-2-isoquinoline-oxide and 4-formyl-4-isoquinoline-oxide both delivered good reactivity, providing enones **2w** and **2x**, respectively. The aldehyde scope was expanded to include diazines, with pyrazine (**2y**) and pyridazine (**2z**) derived N-oxides working well. These two results are significant, as related free azine aldehydes have previously been reported to be poorly reactive in hydroacylation chemistry.^18,21^

**Scheme 4:**
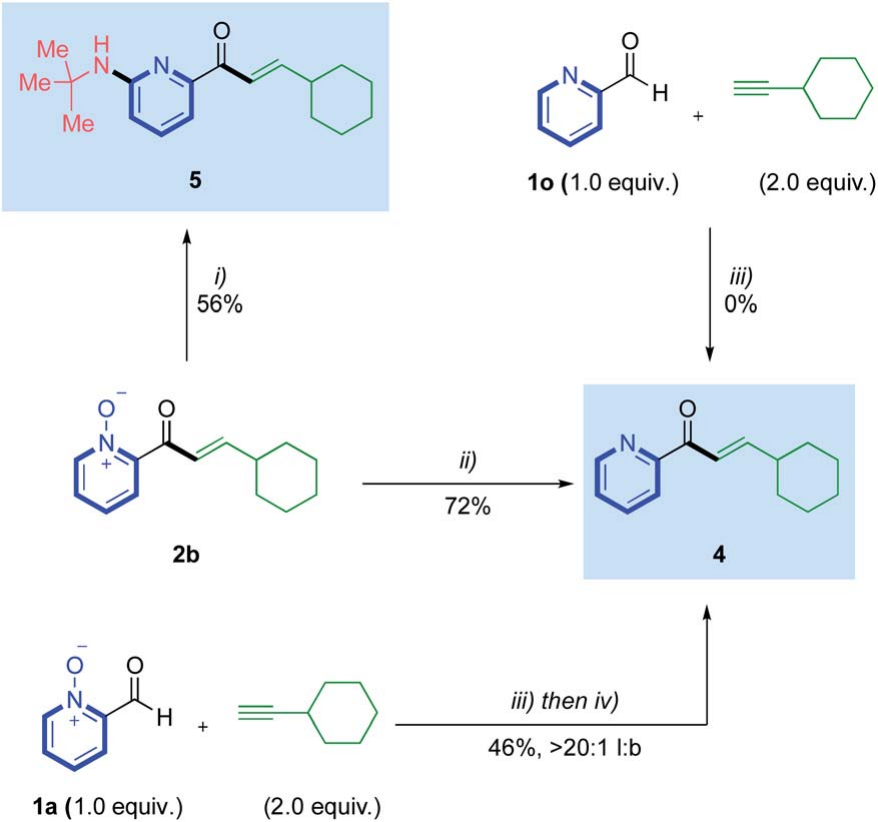
Access to free 2-pyridylic enones 4 and 5 from N-oxide deoxygenations; conditions: (i) Ts_2_O (3.5 equiv.), *t*-BuNH_2_ (8.0 equiv.), 2.5 : 1 PhCF_3_ : CH_2_Cl_2_, 30 °C, 18 h; (ii) PCl_3_ (1.2 equiv.), toluene (0.2 M), rt, 15 min; (iii) Rh(nbd)_2_BF_4_ (5 mol%), DPEPhos (5 mol%), (CH_2_Cl)_2_ (1.25 M), 55 °C, 18 h; (iv) PCl_3_ (1.2 equiv.), (CH_2_Cl)_2_ (0.2 M), rt, 15 min.

With an effective hydroacylation using azine N-oxide aldehydes achieved, we then set out to confirm we could access the corresponding free 2-pyridyl motifs, through product derivatisation (Scheme 4). Deoxygenation of the N–O bond in hydroacylation product **2b** was achieved using PCl_3_, delivering free pyridyl enone **4** in 72% yield. Previous literature accounts have reported various conditions that permit this deoxygenation in tandem with a non-catalytic C–H functionalization at the 6-position of the pyridine ring.^22^ Inspired by this work, we performed a deoxyamination reaction on enone **2b** to form 6-aminopyridylic enone **5** in a respectable 56% yield. Accessing the free 2-pyridyl enone directly from the starting N-oxide aldehyde, *via* an *in situ* reduction, was also achieved through a one-pot hydroacylation/deoxygenation sequence (Scheme 4). This used aldehyde **1a** and ethynylcyclohexane, and yielded pyridyl enone **4** in 46%, >20 : 1 l:b. A control reaction between 2-formylpyridine **1o** and ethynylcyclohexane, confirmed no reactivity, thus reinforcing the necessity for the N-oxide group in these transformations.

**Scheme 5:**
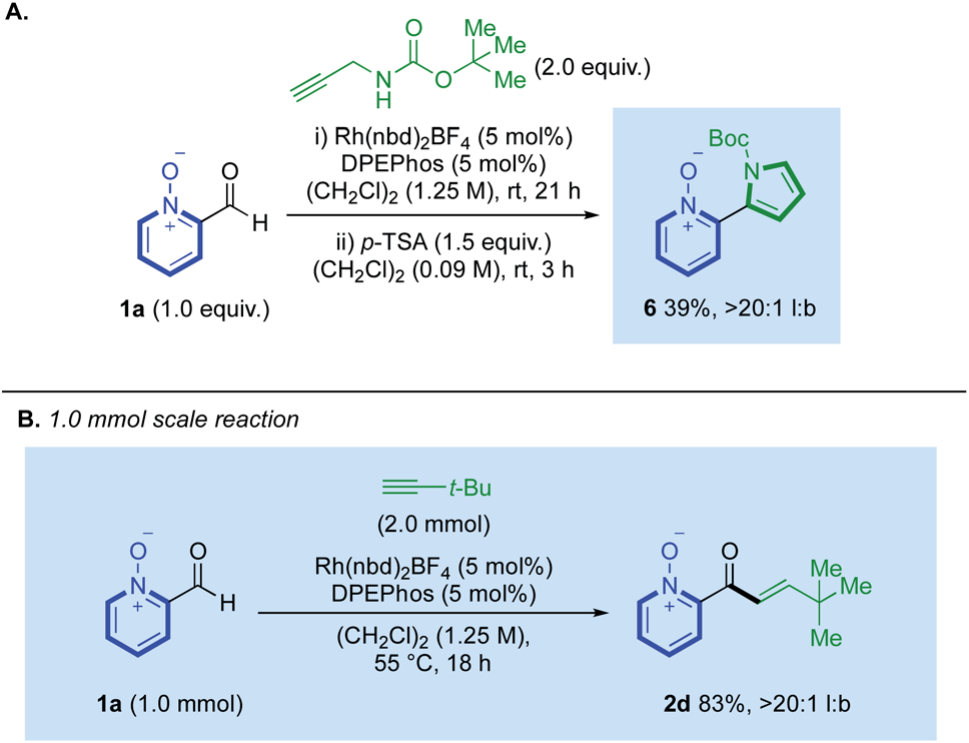
(A) A two-step one-pot hydroacylation/cyclisation towards pyrrolyl ligand 6; (B) employment of aldehyde **1a** on a 1.0 mmol scale.

We also demonstrated how hydroacylation using N-Boc-propargylamine could be performed in tandem with subsequent cyclisation, achieved with stoichiometric *p*-TSA,^23^ to generate pyrrolyl ligand **6**, from a single reaction pot, in 39% yield and >20 : 1 l:b (Scheme 5A). Similar products can be synthesised through a pyridine-N-oxide-directed C(2)-H heteroarylation; however, our strategy prevents the formation of 2-/3-pyrrolyl-coupled regioisomeric mixtures which were observed by Tzschucke.^24^ We additionally conducted a hydroacylation reaction on a larger 1.0 mmol scale, which provided clean conversion to *t*-Bu-substituted enone **2d** in 83%, >20 : 1 l:b (Scheme 5B).

## Conclusions

We have demonstrated that azine aldehyde N-oxides can be used as suitable substrates in intermolecular rhodium-catalysed hydroacylation reactions. Using milder temperatures than other equivalent Rh^I/III^-catalysed quinoline-*N*-oxide C(8)-H functionalisation reactions, this hydroacylation enables the coupling with unactivated terminal alkynes, generating the desired linear enone products in high regioselectivity. The ability for this methodology to tolerate diazines bearing a free nitrogen lone-pair, without the need for other additives, steric modifications or more complex directing groups, is testament to the method's potential synthetic application.

## Data availability

Full experimental and characterisation data are provided as part of the ESI.†

## Author contributions

D. F. M. and J. K. performed the experiments and analysed the data. All authors contributed to the discussion and prepared the manuscript. MCW directed the project.

## Conflicts of interest

There are no conflicts to declare.

## Supplementary Material

SC-012-D1SC03915F-s001
