# Supplementary material for: Azine-N-oxides as effective controlling groups for Rh-catalysed intermolecular alkyne hydroacylation
Source: Chem Sci. 2021 Sep 14;12(39):13068–73. doi: 10.1039/d1sc03915f (PMC8513814; doi:10.1039/d1sc03915f)
Supplement: SC-012-D1SC03915F-s001 [file SC-012-D1SC03915F-s001.pdf]

Electronic Supplementary Material (ESI) for Chemical Science.
This journal is © The Royal Society of Chemistry 2021

Azine *N*-oxides as Effective Controlling Groups for Rh-catalysed Intermolecular Alkyne Hydroacylation

Daniel F. Moseley,[a] Jagadeesh Kalepu[a] and Michael C. Willis[a]*

[a] Chemistry Research Laboratory, University of Oxford, Mansfield Road, Oxford, OX1 3TA, UK

michael.willis@chem.ox.ac.uk

Electronic Supporting Information

Table of Contents

1. **General Information** 3
2. **Substrate Synthesis** 4
   2.1 General Procedures 4
   2.2 Aldehyde Substrates 7
   2.3 Alkyne Substrates 28
3. **Reaction Optimisation** 29
4. **Rhodium-catalysed Hydroacylation** 33
   4.1 General procedure E - Optimised Conditions for *N*-Oxide-Directed Intramolecular Alkyne Hydroacylation 33
   4.2 Alkyne Scope 34
   4.3 Aldehyde Scope 48
5. **Product Derivatisations and Deoxygenations** 61
6. **Additional Findings** 65
7. **References** 70
8. **NMR Spectra of Novel *N*-oxide Substrates and Scope Products** 72

1. General Information

All reactions were performed in oven-dried glassware, under argon, using standard Schlenk techniques. Unless otherwise stated, solvents and reagents were supplied by Sigma Aldrich, FluoroChem, Alfa Aesar or Acros Organics. Acetone and $(CH_2Cl)_2$ (1,2-DCE) were dried over Drierite™ for 16 hours, distilled at atmospheric pressure and *freeze-pump-thawed* with argon before use. Anhydrous THF and DMF were obtained by filtration through anhydrous alumina columns, using an Innovative Technology Inc. PS-400-7 solvent purification system. "Petrol" refers to the fraction of petroleum ether that boils between 40 and 60 °C. Reactions were monitored by analytical Thin Layer Chromatography (TLC), using silica gel F254 aluminium plates. Spots were visualised under UV light (254 nm) or stained with a vanillin or potassium permanganate solution. Flash column chromatography was carried out with matrix 60 silica supplied by Sigma Aldrich. All infrared data were run neat on a Bruker Tensor 27 FT-IR spectrometer, with absorptions reported in wavenumbers (cm^{-1}). Melting points were recorded on the Stuart Scientific Melting Point Apparatus SMP1. ^{1}H and ^{13}C NMR spectra were obtained on a Bruker AVIII400 (400 MHz) using the residual solvent signal as an internal standard ($CDCl_3$: $\boldsymbol{\delta}_H$ = 7.26 ppm, $\boldsymbol{\delta}_C$ = 77.16 ppm). Chemical shifts (δ) are quoted in ppm with multiplicities reported as follows: s, singlet; d, doublet; t, triplet; q, quartet; quin., quintet; m, multiplet; b, broad; app, apparent. All coupling constants (J values) were reported in Hertz (Hz). High and Low resolution ESI mass spectra were recorded on a Waters LCT Premier spectrometer.

2. Substrate Synthesis

2.1 General Procedures

General Procedure A: 2-pyridyl Aldehyde Reductions using $NaBH_4$

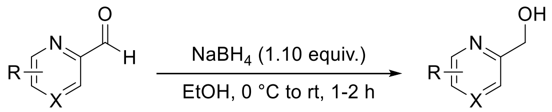

A stirred solution of the corresponding 2-formyl pyridine (1.00 equiv.) in EtOH (1.60 mL/mmol) was cooled to 0 °C before $NaBH_4$ (1.10 equiv.) was added portion-wise. The resulting mixture was warmed to room temperature and stirred for 1-2 h, ***(unless stated otherwise)***. After completion was shown by TLC, the mixture was cooled to 0 °C, quenched with 1 M $HCl_{(aq)}$ (10 mL for 4 mmol scale) and neutralised with sat. aq. $NaHCO_3$. The resulting mixture was extracted using EtOAc (3 × 10 mL for 4 mmol scale) and the combined organic layers were washed with brine, dried over Na_2SO_4 and concentrated *in vacuo* to afford the crude alcohol which was submitted directly into general procedure **C**.

General Procedure B: Methyl Ester Reductions using $CaCl_2$ and $NaBH_4$

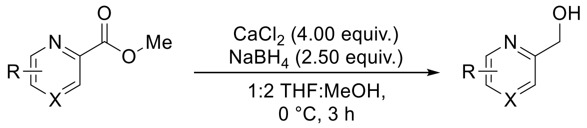

Prepared according to a modified literature procedure.[1] $NaBH_4$ (2.50 equiv.) was added in portions to a slurry of 2,5-pyridinedicarboxylic acid dimethyl ester (1.00 equiv.) and $CaCl_2$ (4.00 equiv.) in a mixture of dry THF (1.80 mL/mmol) and dry MeOH (3.60 mL/mmol) at 0 °C. The reaction was stirred at 0 °C for 3 h. After completion was shown by TLC, excess of $NaBH_4$ was then quenched at 0 °C by adding ice-cold water (30 mL for 7.70 mmol scale). After extraction with CH_2Cl_2 (3 × 20 mL), the combined organic layers were dried over anhydrous

$MgSO_4$ and concentrated *in vacuo* to afford the crude alcohol which was submitted directly into general procedure **C**.

General Procedure C: *N*-oxidations of 2-Pyridylic Alcohols

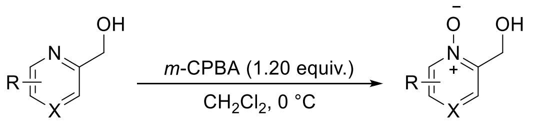

Prepared according to a modified literature procedure.[2] The crude alcohol from either general procedure **A** or **B** was re-dissolved in CH_2Cl_2 (4.50 mL/mmol) and the solution was cooled to 0 °C, with stirring. Then, *m*-CPBA (60% or 75% w/w, 1.20 equiv.) was added portion-wise and the mixture was warmed to room temperature with stirring overnight ***(12-20 h, unless stated otherwise)***. ***Note: For more the more sterically hindered 6-halopyridines, stirring at 80 °C in 1,2-DCE, instead of CH_2Cl_2, was required to achieve full conversion.*** After completion was shown by TLC, the solvent was removed *in vacuo*. Et_2O (4.50 mL/mmol) was then added and the solution was stirred vigorously at room temperature for 10-15 min. Büchner filtration then followed to yield the corresponding alcohol *N*-oxide, which was washed with Et_2O (3 × 10 mL for 4 mmol scale). If a solid precipitate did not form during stirring in Et_2O, the crude residue was concentrated *in vacuo* and purified by column chromatography.

General Procedure D: Pyridylic Alcohol Oxidations using MnO_2

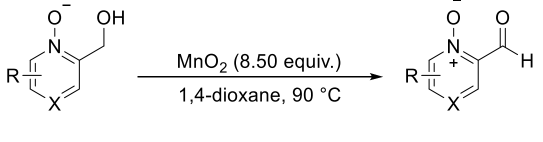

Prepared according to a modified literature procedure.[2] To a solution of the corresponding pyridylic alcohol *N*-oxide (1.00 equiv.) in dry 1,4-dioxane (7.8 mL/mmol) was added MnO_2 (8.50 equiv.) and the mixture was stirred at 90 °C for 1-3 h, ***unless stated otherwise***. After

completion was shown by TLC, the reaction mixture was cooled to room temperature before the addition of celite and solvent removal *in vacuo*. The celite-bound residue was then loaded directly onto a column and purified using either an EtOAc/Petrol or CH_2Cl_2:Acetone eluent system. ***Note: It is crucial that a MeOH/EtOH eluent mixture is not used during column chromatography, as this leads to product degradation***.

2.2 Aldehyde Substrates

2-(Hydroxymethyl)pyridine 1-oxide (S1) and 2-formylpyridine 1-oxide (1a):

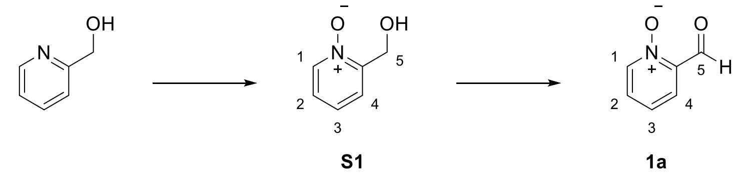

2-(Hydroxymethyl)pyridine 1-oxide (S1): Prepared according to general procedure **C**, using 2-pyridinemethanol (2.65 mL, 27.5 mmol, 1.00 equiv.) and *m*-CPBA (75% w/w, 7.60 g, 33.0 mmol, 1.20 equiv.) stirring for 60 h. After completion, Et_2O trituration gave crude bromo-2-(Hydroxymethyl)pyridine 1-oxide **S1** (2.65 g, 21.2 mmol, 77%) as an off-white powdered solid.

m.p 136-139 °C (Et_2O); {*Lit:* 130-132 °C}[3]; **δ_H** (400 MHz, $CDCl_3$) 8.23 (1H, dd, J = 6.4, 1.2 Hz, *Ar*), 7.41 (1H, dd, J = 7.7, 2.1 Hz, *Ar*), 7.34 (1H, td, J = 7.7, 1.2 Hz, *Ar*), 7.26 (1H, td, J = 7.5, 7.0, 2.2 Hz, *Ar*), 4.95-4.84 (1H, bs, O*H*), 4.80 (2H, s, C(5)*H*); **m/z LRMS** (ESI^+) 148.0 $[M + Na]^+$. *Data is consistent with literature[2]*

2-Formylpyridine 1-oxide (1a): Prepared according to a modified literature procedure,[2] using 2-(hydroxymethyl)pyridine 1-oxide **S1** (700 mg, 5.57 mmol, 1.00 equiv.) and MnO_2 (85% w/w, 4.90 g, 47.9 mmol, 8.50 equiv.) with stirring for 5 h. After this time, the mixture was filtered hot, through celite and the filter cake was separately washed with hot dioxane and EtOH, and the filtrate was concentrated *in* vacuo. Purification by column chromatography (SiO_2, neat EtOAc) yielded 2-formyl-5-methoxypyridine 1-oxide **1a** (98 mg, 0.64 mmol, 66%) as a white powdered solid.

m.p 72-78 °C (CH_2Cl_2); **δ_H** (400 MHz, $CDCl_3$) 10.63 (1H, d, J = 0.8 Hz, C(5)*H*), 8.21 (1H, dt, J = 6.5, 0.8 Hz, *Ar*), 7.82 (1H, dd, J = 7.8, 2.2 Hz, *Ar*), 7.46 (1H, ddd, J = 7.5, 6.5, 2.2 Hz, *Ar*),

7.32 (1H, tt, J = 7.7, 1.0 Hz, *Ar*); **δ$_C$** (101 MHz, $CDCl_3$) 185.8, 143.9, 140.5, 130.1, 125.7, 125.3; **m/z LRMS** (ESI^+) 124.0 $[M + H]^+$. *Data is consistent with literature[2]*

5-Bromo-2-formylpyridine 1-oxide (1b)

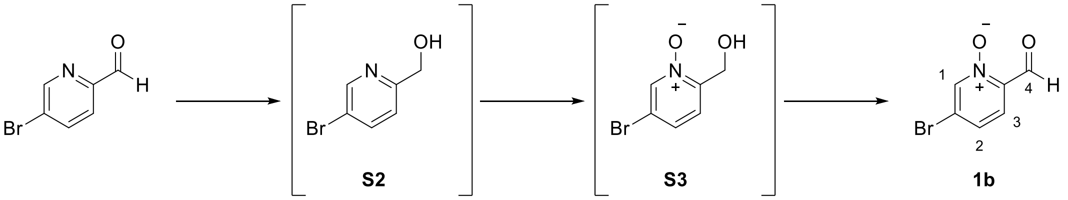

5-Bromo-2-formylpyridine 1-oxide (1b): Prepared according to general procedures **A** and **C**. Procedure **A** used 5-bromopicolinaldehyde (552 mg, 3.00 mmol, 1.00 equiv.) and $NaBH_4$ (125 mg, 3.30 mmol, 1.10 equiv.) with stirring for 1 h. Procedure **C** used crude alcohol **S2** obtained from procedure **A** and *m*-CPBA (60% w/w, 1.03 g, 3.60 mmol, 1.20 equiv.), with stirring for 12 h. After completion, Et_2O trituration gave crude bromo-2-(hydroxymethyl)pyridine-1-oxide **S3** (300 mg, 0.980 mmol). This crude compound was subjected to general method **D** without any further purification, using MnO_2 (10.9 g, 12.5 mmol). Purification by column chromatography (SiO_2, from 1:1 EtOAc: Petrol to 3:1 EtOAc: Petrol) yielded title compound **1b** (100 mg, 0.500 mmol, 17% over three steps) as a white powdered solid.

m.p 134-135 °C (CH_2Cl_2); **δ$_H$** (400 MHz, $CDCl_3$) 10.54 (1H, d, J = 0.8 Hz, C(4)*H*), 8.36 (1H, dd, J = 1.7, 0.5 Hz, *Ar*), 7.69 (1H, d, J = 8.5 Hz, *Ar*), 7.45 (1H, ddd, J = 8.5, 1.7, 0.8 Hz, *Ar*); **δ$_C$** (101 MHz, $CDCl_3$) 185.0, 142.9, 141.8, 128.5, 126.0, 125.7; **ν_{max}**/cm^{-1} ($CHCl_3$) 2981, 1685, 1375, 1242, 1177, 1083, 955, 819; **m/z HRMS** (ESI^+) $[M + H]^+$ $C_6H_5O_2N^{79}Br^+$ calc. 201.9498, found 201.9500. *Data is consistent with literature[2]*

2-(Hydroxymethyl)-5-methoxypyridine 1-oxide (S5) and 2-formyl-5-methoxypyridine 1-oxide (1c)

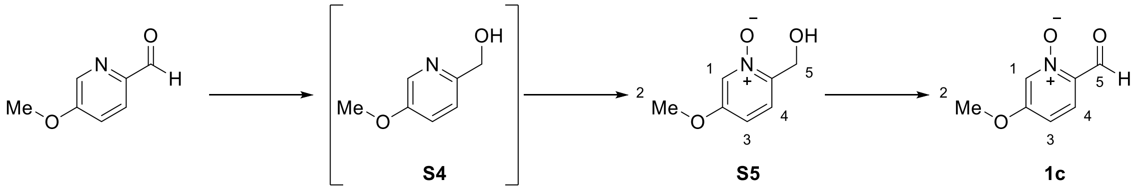

2-(Hydroxymethyl)-5-methoxypyridine 1-oxide (S5): Prepared according to general procedures **A** and **C**. Procedure **A** used 5-methoxypicolinaldehyde (200 mg, 1.45 mmol, 1.00 equiv.) and $NaBH_4$ (60.0 mg, 1.60 mmol, 1.10 equiv.) with stirring for 1 h. Procedure **C** used crude alcohol **S4** obtained from procedure **A** and *m*-CPBA (60% w/w, 499 mg, 1.74 mmol, 1.20 equiv.) stirring for 12 h. After completion, Et_2O trituration gave pure 2-(hydroxymethyl)-5-methoxypyridine 1-oxide **S5** (150 mg, 0.97 mmol, 67% yield over two steps) as an off-white powdered solid.

m.p 40-42 °C (Et_2O); δ_H (400 MHz, $CDCl_3$) 7.96 (1H, d, J = 2.4 Hz, *Ar*), 7.23 (1H, d, J = 8.8 Hz, *Ar*), 6.90 (1H, dd, J = 8.8, 2.4 Hz, *Ar*), 5.05-4.86 (1H, bs, O*H*), 4.71 (2H, s, C(5)*H*), 3.81 (3H, s, OC(2)H_3); δ_C (101 MHz, $CDCl_3$) 157.2, 143.0, 127.6, 124.3, 114.3, 60.7, 56.3; ν_{max}/cm^{-1} ($CHCl_3$) 3233, 2839, 1600, 1501, 1290, 1270, 1248, 1067, 1028, 830; **m/z HRMS** (ESI$^+$) $[M + H]^+$ $C_7H_{10}O_3N^+$ calc. 156.0655, found 156.0655.

2-Formyl-5-methoxypyridine 1-oxide (1c): Prepared according to general procedure **D**, using 2-(hydroxymethyl)-5-methoxypyridine 1-oxide **S5** (150 mg, 0.970 mmol, 1.00 equiv.) and MnO_2 (725 mg, 8.33 mmol, 8.50 equiv.), with stirring for 1 h. Purification by column chromatography (SiO_2, from 1:1 EtOAc: Petrol to 3:1 EtOAc: Petrol) yielded 2-formyl-5-methoxypyridine 1-oxide **1c** (98 mg, 0.64 mmol, 66%) as a white powdered solid.

m.p 149-151 °C (CH_2Cl_2); **δ_H** (400 MHz, $CDCl_3$) 10.49 (1H, d, J = 0.8 Hz, C(5)*H*), 7.91 (1H, d, J = 2.3 Hz, *Ar*), 7.74 (1H, d, J = 9.0 Hz, *Ar*), 6.88 (1H, ddd, J = 9.0, 2.3, 0.8 Hz, *Ar*), 3.90 (3H, s, OC(2)H_3); **δ_C** (101 MHz, $CDCl_3$) 185.0, 161.5, 138.5, 127.9, 126.1, 112.5, 56.7; **ν_{max}**/cm^{-1} ($CHCl_3$) 2981, 2886, 1683, 1592, 1406, 1375, 1316, 1275, 1195, 1176, 1151, 964, 832, 813, 662; **m/z HRMS** (ESI$^+$) [M + Na]$^+$ $C_7H_7O_3N^{23}Na^+$ calc. 176.0316, found 176.0320.

Methyl 6-(hydroxymethyl)nicotinate (S6), 2-(hydroxymethyl)-5-(methoxycarbonyl)pyridine 1-oxide (S7) and 2-bromo-6-formylpyridine 1-oxide (1d)

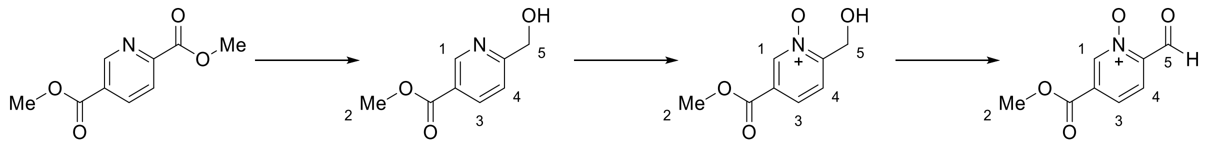

S6 S7 1d

Methyl 6-(hydroxymethyl)nicotinate (S6): Prepared according to general procedure **B**, using dimethyl pyridine-2,5-dicarboxylate (1.50 g, 7.70 mmol, 1.00 equiv.), $CaCl_2$ (3.42 g, 30.8 mmol, 4.00 equiv.) and $NaBH_4$ (726 mg, 19.2 mmol, 2.50 equiv.), with stirring for 3 h. Work-up yielded pure alcohol **S6** (951 mg, 5.69 mmol, 74%) as an off-white solid.

m.p 64-68 °C (CH_2Cl_2); {*Lit:* 75-78 °C (Et_2O)}[4]; **δ_H** (400 MHz, $CDCl_3$) 9.16-9.11 (1H, m, *Ar*), 8.28 (1H, dd, J = 8.2, 2.1 Hz, *Ar*), 7.38 (1H, d, J = 8.1 Hz, *Ar*), 4.83 (2H, s, C(5)H_2), 3.94 (3H, s, C(2)H_3); **δ_C** (101 MHz, $CDCl_3$) 165.7, 163.8, 150.0, 138.0, 125.1, 120.2, 64.4, 52.5; **ν_{max}**/cm^{-1} ($CHCl_3$) 3341, 2981, 1723, 1600, 1436, 1386, 1289, 1117, 1064, 1024, 955, 754; **m/z LRMS** (ESI$^+$) 168.0 [M + H]$^+$. *Data is consistent with literature*[1]

2-(Hydroxymethyl)-5-(methoxycarbonyl)pyridine 1-oxide (S7): Prepared according to procedure **C**, using alcohol **S6** (400 mg, 2.39 mmol, 1.00 equiv.) and *m*-CPBA (60% w/w, 823 g, 2.87 mmol, 1.20 equiv.), with stirring for 12 h. After completion, Et_2O trituration gave pure

2-(Hydroxymethyl)-5-(methoxycarbonyl)pyridine 1-oxide **S7** (380 mg, 2.08 mmol, 87%) as an off-white solid.

m.p 124-126 °C (Et_2O); **δ_H** (400 MHz, $CDCl_3$) 8.78 (1H, d, *J* = 1.6 Hz, *Ar*), 7.91 (1H, dd, *J* = 8.1, 1.5 Hz, *Ar*), 7.54 (1H, d, *J* = 8.1 Hz, *Ar*), 4.84 (2H, s, C(5)*H_2*), 3.95 (3H, s, C(2)*H_3*); **δ_C** (101 MHz, $CDCl_3$) 163.2, 154.2, 140.3, 128.3, 127.5, 124.2, 60.8, 53.1; **ν_{max}**/cm^{-1} ($CHCl_3$) 3134, 3053, 2972, 1732, 1387, 1294, 1214, 1104, 1077, 988, 755; **m/z HRMS** (ESI^+) $[M + H]^+$ $C_8H_{10}O_4N^+$ calc. 184.0604, found 184.0606.

2-Formyl-5-(methoxycarbonyl)pyridine 1-oxide (1d): Prepared according to general procedure **D**, using 2-bromo-6-(hydroxymethyl)pyridine 1-oxide **S7** (350 mg, 1.91 mmol, 1.00 equiv.) and MnO_2 (1.66 g, 19.1 mmol, 10.0 equiv.), with stirring for 2 h. Purification by column chromatography (SiO_2, from 1:1 EtOAc:Petrol to 3:1 EtOAc:Petrol) yielded 2-formyl-5-(methoxycarbonyl)pyridine 1-oxide **1d** (180 mg, 0.990 mmol, 52%). ***Note: a higher loading of MnO_2 was used here, to achieve full conversion.***

m.p 158-160 °C (CH_2Cl_2); **δ_H** (400 MHz, $CDCl_3$) 10.60 (1H, d, *J* = 0.7 Hz, *Ar*), 8.77-8.74 (1H, m, *J* = 1.0 Hz, *Ar*), 7.90-7.81 (2H, m, *Ar*), 3.99 (3H, s, C(2)*H_3*); **δ_C** (101 MHz, $CDCl_3$) 185.3, 162.9, 145.8, 141.7, 132.8, 125.5, 125.4, 53.6; **ν_{max}**/cm^{-1} ($CHCl_3$) 2981, 1718, 1695, 1302, 1178, 1089, 994, 754; **m/z HRMS** (ESI^+) $[M + H]^+$ $C_8H_8O_4N^+$ calc. 182.0448, found 182.0451.

2-Bromo-6-(hydroxymethyl)pyridine 1-oxide (S9) and 2-bromo-6-formylpyridine 1-oxide (1e)

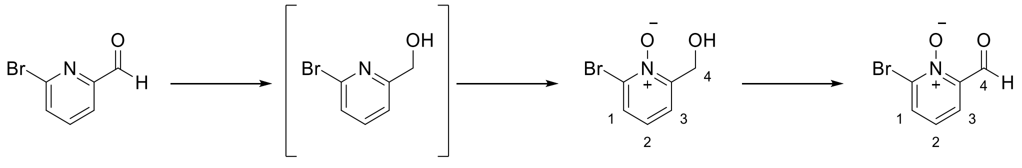

2-Bromo-6-(hydroxymethyl)pyridine 1-oxide (S9): Prepared according to general procedures **A** and **C**. Procedure **A** used 6-bromopicolinealdehyde (1.00 g, 5.37 mmol, 1.00 equiv.) and $NaBH_4$ (224 mg, 5.91 mmol, 1.10 equiv.), with stirring for 1 h. Procedure **C** used crude alcohol **S8** obtained from procedure **A** and *m*-CPBA (60% w/w, 1.85 g, 6.44 mmol, 1.20 equiv.), ***and the reaction was instead stirred at 80 °C in 1,2-DCE for 18 h, in order to achieve full conversion***. After completion, Et_2O trituration gave pure 2-bromo-6-(hydroxymethyl)pyridine 1-oxide **S9** (760 mg, 3.72 mmol, 69% yield over two steps) as an off-white powdered solid.

m.p 146-149 °C (Et_2O); **δ_H** (400 MHz, $CDCl_3$) 7.65 (1H, dd, *J* = 8.1, 2.0 Hz, *Ar*), 7.36 (1H, dd, *J* = 7.7, 2.0 Hz, *Ar*), 7.17 (1H, app. t, *J* = 7.9 Hz, *Ar*), 4.82 (2H, s, C(4)H_2), 4.57-4.37 (1H, bs, O*H*); **δ_C** (101 MHz, $CDCl_3$) 151.8, 133.5, 129.7, 126.5, 123.5, 62.0; **ν_{max}**/cm^{-1} ($CHCl_3$) 3217, 2981, 1471, 1386, 1235, 1083, 1067, 847, 791, 659; **m/z HRMS** (ESI$^+$) [M + H]$^+$ $C_6H_7O_2N^{79}Br^+$ calc. 203.9655, found 203.9657. *Data is consistent with literature*[2]

2-Bromo-6-formylpyridine-1-oxide (1e): Prepared according to general procedure **D**, using 2-bromo-6-(hydroxymethyl)pyridine 1-oxide **S9** (400 mg, 1.96 mmol) and MnO_2 (1.45 g, 16.7 mmol, 8.50 equiv.), with stirring for 2 h. Purification by column chromatography (SiO_2, from 1:1 EtOAc:Petrol to 3:1 EtOAc:Petrol) yielded 2-bromo-6-formylpyridine-1-oxide **1e** (180 mg, 0.89 mmol, 45% yield) as an off-white powdered solid.

m.p 110-112 °C (CH_2Cl_2); **δ_H** 10.58 (1H, d, *J* = 0.8 Hz, C(4)*H*), 7.86 (1H, dd, *J* = 8.1, 2.1 Hz, *Ar*), 7.79 (1H, dd, *J* = 7.8, 2.1 Hz, *Ar*), 7.18 (1H, app. td, *J* = 7.9, 0.8 Hz, *Ar*); **δ_C** (101 MHz,

$CDCl_3$) 185.7, 145.3, 134.7, 134.6, 124.9, 124.6; **ν$_{max}$**/cm^{-1} ($CHCl_3$) 2981, 1689, 1592, 1383, 1357, 1234, 1128, 716; **m/z HRMS** (ESI$^+$) [M + H]$^+$ $C_6H_5O_2N^{79}Br^+$ calc. 201.9498, found 201.9501. *Data is consistent with literature[2]*

2-Chloro-6-(hydroxymethyl)pyridine 1-oxide (S11) and 2-Chloro-6-formylpyridine-1-oxide (1f)

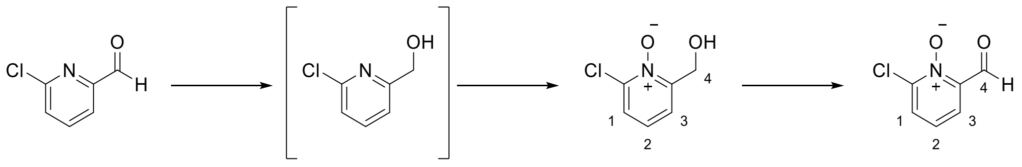

S10 **S11** **1f**

2-Chloro-6-(hydroxymethyl)pyridine 1-oxide (S11): Prepared according to general procedures **A** and **C**. Procedure **A** used 6-chloropicolinaldehyde (500 mg, 3.53 mmol, 1.00 equiv.) and $NaBH_4$ (148 mg, 3.89 mmol, 1.10 equiv.), with stirring for 1 h. Procedure **C** used crude alcohol **S10** obtained from procedure **A** and *m*-CPBA (60% w/w, 1.21 g, 4.24 mmol, 1.20 equiv.), ***and the reaction was instead stirred at 80 °C in 1,2-DCE for 18 h, in order to achieve full conversion***. After completion, Et_2O trituration gave pure 2-chloro-6-(hydroxymethyl)pyridine 1-oxide **S11** (350 mg, 2.19 mmol, 62% yield over two steps) as an off-white powdered solid.

m.p 110-114 °C (Et_2O); **δ$_H$** (400 MHz, $CDCl_3$) 7.46 (1H, dd, *J* = 8.1, 2.0 Hz, *Ar*), 7.39 (1H, dd, *J* = 7.8, 2.0 Hz, *Ar*), 7.25 (1H, app. t, *J* = 7.9 Hz, *Ar*), 4.80 (2H, s, C(4)H_2), 4.78-4.60 (1H, bs, O*H*); **δ$_C$** (101 MHz, $CDCl_3$) 152.4, 142.1, 126.6, 125.6, 122.4, 61.2; **ν$_{max}$**/cm^{-1} ($CHCl_3$) 3199, 1474, 1391, 1335, 1236, 1205, 1184, 1157, 1087, 1070, 867, 845, 795, 681; **m/z HRMS** (ESI$^+$) [M + H]$^+$ $C_6H_7O_2N^{35}Cl^+$ calc. 160.0160, found 160.0161.

2-Chloro-6-formylpyridine-1-oxide (1f): Prepared according to general procedure **D**, using 2-chloro-6-(hydroxymethyl)pyridine 1-oxide **S11** (315 mg, 1.98 mmol, 1.00 equiv.) and a higher loading of MnO_2 (2.00 g, 23.0 mmol, 11.6 equiv.), with stirring for 2 h. Purification by column chromatography (SiO_2, from 1:1 EtOAc:Petrol to 3:1 EtOAc:Petrol) yielded 2-chloro-6-

formylpyridine-1-oxide **1f** (130 mg, 0.82 mmol, 42% yield) as an off-white powdered solid. ***Note: a higher loading of MnO_2 was used here, to achieve full conversion.***

m.p 101-105 °C (CH_2Cl_2); **δ_H** 10.58 (1H, d, *J* = 0.8 Hz, C(4)*H*), 7.75 (1H, dd, *J* = 7.8, 2.1 Hz, *Ar*), 7.69 (1H, dd, *J* = 8.1, 2.1 Hz, *Ar*), 7.26 (1H, dd, *J* = 15.9, 0.8 Hz, *Ar*); **δ_C** (101 MHz, $CDCl_3$) 185.4, 145.1, 143.1, 130.9, 124.8, 123.8; **ν_{max}**/cm^{-1} ($CHCl_3$) 2976, 1695, 1612, 1387, 1367, 847, 784, 738; **m/z HRMS** (ESI^+) $[M + H]^+$ $C_6H_5O_2N^{35}Cl^+$ calc. 158.0003, found 158.0003.

2-(Hydroxymethyl)-4-methylpyridine 1-oxide (S13) and 2-formyl-4-methylpyridine 1-oxide (1g)

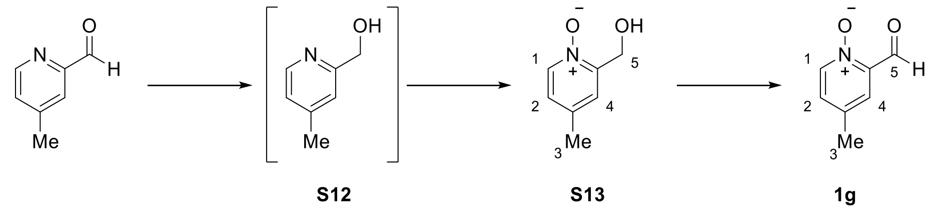

2-(Hydroxymethyl)-4-methylpyridine 1-oxide (S13): Prepared according to general procedures **A** and **C**. Procedure **A** used 4-methylpicolinaldehyde (500 mg, 4.13 mmol, 1.00 equiv.) and $NaBH_4$ (172 mg, 4.54 mmol, 1.10 equiv.) with stirring for 1 h. Procedure **C** used crude alcohol **S12** obtained from procedure **A** and *m*-CPBA (60% w/w, 1.43 g, 4.96 mmol, 1.20 equiv.) with stirring for 12 h. Purification by column chromatography (SiO_2, EtOAc to 1:4 MeOH:EtOAc) yielded 2-(hydroxymethyl)-4-methylpyridine 1-oxide **S13** (356 mg, 2.56 mmol, 62% over two steps) as an off-white solid.

m.p 109-112 °C (CH_2Cl_2); **δ_H** (400 MHz, $CDCl_3$) 8.11 (1H, d, *J* = 6.5 Hz, *Ar*), 7.19 (1H, d, *J* = 2.5 Hz, *Ar*), 7.04 (1H, dd, *J* = 6.6, 2.5 Hz, *Ar*), 5.28 (1H, bs, O*H*), 4.76 (2H, s, C(5)H_2), 2.35 (3H, s, C(3)H_3); **δ_C** (101 MHz, $CDCl_3$) 149.7, 139.7, 138.8, 125.4, 125.3, 61.0, 20.6; **ν_{max}**/cm^{-1} ($CHCl_3$) 3227, 1480, 1456, 1213, 1066, 820, 732; **m/z HRMS** (ESI^+) $[M + H]^+$ $C_7H_{10}O_2N^+$ calc. 140.0706, found 140.0706.

2-Formyl-4-methylpyridine 1-oxide (1g): Prepared according to general procedure **D**, using 2-(hydroxymethyl)-4-methylpyridine 1-oxide **S13** (356 mg, 2.56 mmol, 1.00 equiv.) and MnO_2 (1.89 g, 21.76 mmol, 8.50 equiv.), with stirring for 2 h. Purification by column chromatography (SiO_2, 1:1 CH_2Cl_2:Acetone) yielded 2-formyl-4-methylpyridine 1-oxide **1g** (195 mg, 1.43 mmol, 56%) as a pale-yellow solid.

m.p 101-103 °C (CH_2Cl_2); **δ_H** (400 MHz, $CDCl_3$) 10.61 (1H, s, C(5)*H*), 8.09 (1H, d, J = 6.7 Hz, *Ar*), 7.60 (1H, d, J = 2.7 Hz, *Ar*), 7.28-7.21 (1H, m, *Ar*), 2.37 (3H, s, C(3)H_3); **δ_C** (101 MHz, $CDCl_3$) 186.1, 142.9, 139.8, 137.1, 130.8, 125.9, 20.4; **ν_{max}**/cm^{-1} ($CHCl_3$) 1695, 1475, 1200, 1066, 1037, 826; **m/z HRMS** (ESI^+) $[M + H]^+$ $C_7H_8O_2N^+$ calc. 138.0550, found 138.0548.

2-(Hydroxymethyl)-3-methylpyridine 1-oxide (S15) and 2-formyl-3-methylpyridine 1-oxide (1h)

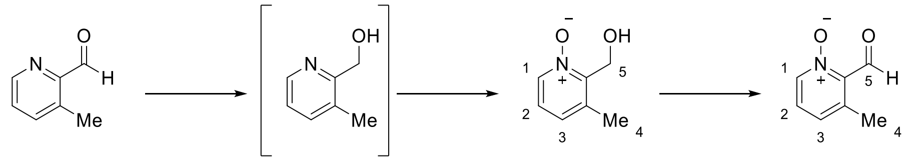

S14 **S15** **1h**

2-(Hydroxymethyl)-3-methylpyridine 1-oxide (S15): Prepared according to general procedures **A** and **C**. Procedure **A** used 3-methylpicolinaldehyde (463 mg, 4.13 mmol, 1.00 equiv.) and $NaBH_4$ (172 mg, 4.54 mmol, 1.10 equiv.), with stirring for 1.5 h. Procedure **C** used crude alcohol **S14** obtained from procedure **A** and *m*-CPBA (60% w/w, 1.43 g, 4.96 mmol, 1.20 equiv.), with stirring for 2 h. Purification by column chromatography (SiO_2, 1:1 EtOAc:Petrol to 1:9 MeOH:EtOAc) yielded 2-(hydroxymethyl)-3-methylpyridine 1-oxide **S15** (333 mg, 2.40 mmol, 58% over two steps) as an off-white solid.

m.p 131-135 °C (CH_2Cl_2); **δ_H** (400 MHz, $CDCl_3$) 8.10 (1H, dd, J = 5.7, 2.0 Hz, *Ar*), 7.18-7.08 (2H, m, *Ar*), 5.74 (1H, bs, O*H*), 4.82 (2H, s, C(5)H_2), 2.37 (3H, s, C(4)H_3); **δ_C** (101 MHz,

$CDCl_3$) 148.7, 137.5, 134.6, 129.4, 124.1, 58.0, 18.5; **ν$_{max}$**/cm^{-1} ($CHCl_3$) 3144, 1251, 1224, 1182, 1093, 1058, 1042, 1026, 804; **m/z HRMS** (ESI$^+$) [M + H]$^+$ $C_7H_{10}O_2N^+$ calc. 140.0706, found 140.0705.

2-Formyl-3-methylpyridine 1-oxide (1h): Prepared according to general procedure **D**, using 2-(hydroxymethyl)-3-methylpyridine 1-oxide **S15** (270 mg, 1.94 mmol, 1.00 equiv.) and MnO_2 (1.43 g, 16.5 mmol, 8.50 equiv.), with stirring for 3 h. Purification by column chromatography (SiO_2, 1:1 CH_2Cl_2:Acetone) yielded 2-formyl-3-methylpyridine 1-oxide **1h** (147 mg, 1.07 mmol, 55%) as an off-white solid.

m.p 105-108 °C (CH_2Cl_2); **δ$_H$** (400 MHz, $CDCl_3$) 10.63 (1H, d, *J* = 0.6 Hz, C(5)*H*), 8.10 (1H, d, *J* = 6.4 Hz, *Ar*), 7.30 (1H, dd, *J* = 7.9, 6.4 Hz, *Ar*), 7.11 (1H, dq, *J* = 8.0, 0.9 Hz, *Ar*), 2.53 (3H, s, C(4)*H$_3$*); **δ$_C$** (101 MHz, $CDCl_3$) 188.6, 142.7, 139.7, 137.9, 128.8, 128.4, 19.2; **ν$_{max}$**/cm^{-1} ($CHCl_3$) 2981, 1699, 1587, 1476, 1445, 1418, 1382, 1265, 1207, 1177, 1092, 1070, 1042, 960, 834, 752; **m/z HRMS** (ESI$^+$) [M + H]$^+$ $C_7H_8O_2N^+$ calc. 138.0550, found 138.0548.

3-Bromo-2-(hydroxymethyl)pyridine 1-oxide (S17) and 3-bromo-2-formylpyridine 1-oxide (1i)

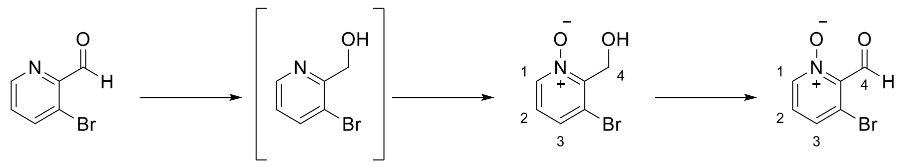

S16 **S17** **1i**

3-Bromo-2-(hydroxymethyl)pyridine 1-oxide (S17): Prepared according to the general procedures **A** and **C**. Procedure **A** used 3-bromopicolinaldehyde (1.00 g, 5.43 mmol, 1.00 equiv.) and $NaBH_4$ (227 mg, 5.98 mmol, 1.10 equiv.), with stirring for 1 h. Procedure **C** used crude alcohol **S16** obtained from procedure **A** and *m*-CPBA (60% w/w, 1.86 g, 6.52 mmol,

1.20 equiv.), with stirring for 12 h. After completion, Et_2O trituration gave pure 3-bromo-2-(hydroxymethyl)pyridine 1-oxide **S17** (760 mg, 3.76 mmol, 69% yield over two steps).

m.p 136-140 °C (Et_2O); **δ_H** (400 MHz, DMSO-d_6) 8.31 (1H, dd, *J* = 6.5, 1.0 Hz, *Ar*), 7.64 (1H, dd, *J* = 8.3, 1.0 Hz, *Ar*), 7.31 (1H, dd, *J* = 8.3, 6.5 Hz, *Ar*), 5.41 (1H, s, O*H*), 4.79 (2H, s, C(4)H_2); **δ_C** (101 MHz, DMSO-d_6) 149.2, 138.8, 129.5, 125.7, 121.5, 57.6; **ν_{max}**/cm^{-1} ($CHCl_3$) 3258, 3104, 3010, 2875, 1412, 1229, 1164, 1036, 969, 862; **m/z HRMS** (ESI$^+$) [M + H]$^+$ $C_6H_7O_2N^{79}Br^+$ calc. 203.9655, found 203.9657.

3-Bromo-2-formylpyridine 1-oxide (1i): Prepared according to general procedure **D**, using 3-bromo-2-(hydroxymethyl)pyridine 1-oxide **S17** (400 mg, 1.96 mmol, 1.00 equiv.) and MnO_2 (2.56 g, 29.4 mmol, 15.0 equiv.). Purification by column chromatography (SiO_2, from 1:1 EtOAc:Petrol to 3:1 EtOAc: Petrol) yielded 3-bromo-2-formylpyridine 1-oxide **1i** (180 mg, 0.90 mmol, 46%) as an off-white solid. ***Note: a higher loading of MnO_2 was used here, to achieve full conversion.***

m.p 122-125 °C (CH_2Cl_2); **δ_H** (400 MHz, $CDCl_3$) 10.28 (1H, d, *J* = 0.7 Hz, C(4)*H*), 8.18 (1H, dd, *J* = 6.5, 1.0 Hz, *Ar*), 7.53 (1H, app. dt, *J* = 8.4, 0.8 Hz, *Ar*), 7.26 (1H, dd, *J* = 8.4, 6.4 Hz, *Ar*); **δ_C** (101 MHz, $CDCl_3$) 184.3, 142.8, 139.3, 131.1, 128.2, 120.2; **ν_{max}**/cm^{-1} ($CHCl_3$) 3102, 1707, 1455, 1421, 1228, 1180, 1064, 1028, 987, 902, 792, 726; **m/z HRMS** (ESI$^+$) [M + H]$^+$ $C_6H_5O_2N^{79}Br^+$ calc. 201.9498, found 201.9501.

2,6-Bis(hydroxymethyl)pyridine 1-oxide (S18) and 2,6-Diformylpyridine 1-oxide (1j)

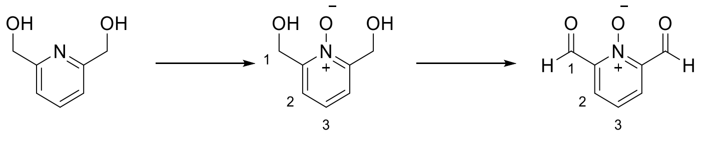

S18 **1j**

For 2,6-Bis(hydroxymethyl)pyridine 1-oxide (S18): Prepared according to general procedure **C**, using pyridine-2,6-diyldimethanol (2.00 g, 14.4 mmol, 1.00 equiv.), with *m*-CPBA (60% w/w, 4.96 g, 17.3 mmol, 1.20 equiv.) ***as a more diluted solution in $CHCl_3$ (0.075 M) instead of CH_2Cl_2.***[5] The reaction was stirred for 2 h. After completion, Et_2O trituration gave 2,6-bis(hydroxymethyl)pyridine 1-oxide **S18** (2.17 g, 14.0 mmol, 97%) as a white powdered solid.

m.p 135-138 °C (Et_2O); **δ_H** (400 MHz, DMSO-d$_6$) 7.51-7.39 (3H, m, *Ar*), 5.57 (2H, t, *J* = 5.7 Hz, O*H*), 4.60 (4H, d, *J* = 5.7 Hz, C(1)H_2); **δ_C** (101 MHz, MeOD) 153.5, 130.2, 122.4, 59.6; **ν_{max}**/cm^{-1} ($CHCl_3$) 3455, 3264, 2972, 2890, 1412, 1250, 1207, 1155, 1091, 954, 840, 776, 669; **m/z HRMS** (ESI$^+$) [M + H]$^+$ $C_7H_{10}O_3N^+$ calc. 156.0655, found 156.0654.

2,6-Diformylpyridine 1-oxide (1j): Prepared according to general procedure **D**, using 2,6-bis(hydroxymethyl)pyridine 1-oxide **S18** (490 mg, 3.16 mmol, 1.00 equiv.) and MnO_2 (4.67 g, 53.72 mmol, 17.0 equiv.), with stirring for 6 h. Purification by column chromatography (SiO_2, 1:1 to 3:1 EtOAc:Pentane) yielded 2,6-diformylpyridine 1-oxide **1j** (373 mg, 2.48 mmol, 78%) as an off-white powdered solid. ***Note: double the loading of MnO_2 was used here, to permit double alcohol oxidation.***

m.p 182-184 °C (CH_2Cl_2); **δ_H** (400 MHz, DMSO-d$_6$) 10.39 (2H, d, *J* = 0.8 Hz, C(1)*H*), 8.00 (2H, d, *J* = 7.8 Hz, *Ar*), 7.53 (1H, t, *J* = 7.8 Hz, *Ar*); **δ_C** (101 MHz, DMSO-d$_6$) 185.3, 144.0, 129.1, 125.2; **ν_{max}**/cm^{-1} ($CHCl_3$) 3046, 2981, 1702, 1677, 1549, 1399, 1379, 1269, 1249,

1205, 906, 850, 809, 708; **m/z HRMS** (ESI^+) $[M + H]^+$ $C_7H_6O_3N^+$ calc. 152.0342, found 152.0342.

Isoquinolin-1-ylmethanol (S19), 1-(Hydroxymethyl)isoquinoline 2-oxide (S20) and 1-Formylisoquinoline 2-oxide (1k)

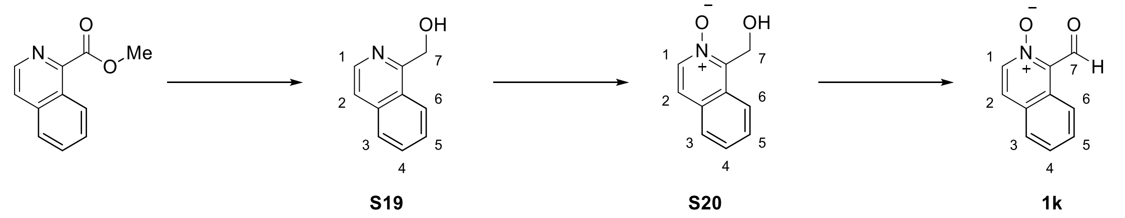

For Isoquinolin-1-ylmethanol (S19): Prepared according to general procedure **B**, using methyl isoquinoline-1-carboxylate (1.46 g, 7.79 mmol, 1.00 equiv.), $CaCl_2$ (3.46 g, 31.2 mmol, 4.00 equiv.) and $NaBH_4$ (738 mg, 19.5 mmol, 2.50 equiv.) with stirring for 5 h. Work-up yielded isoquinolin-1-ylmethanol **S19** (1.24 g, 7.78 mmol, >99%), as an off-white solid.

m.p 60-62 °C (CH_2Cl_2); **δ_H** (400 MHz, $CDCl_3$) 8.43 (1H, d, J = 5.8 Hz, *Ar*), 7.95-7.87 (1H, m, *Ar*), 7.87-7.81 (1H, m, J = 9.2, 1.0 Hz, *Ar*), 7.70 (1H, ddd, J = 8.2, 6.9, 1.2 Hz, *Ar*), 7.65-7.54 (2H, m, *Ar*), 5.22 (2H, s, C(7)H_2), 4.71 (1H, s, O*H*); **δ_C** (101 MHz, $CDCl_3$) 157.6, 140.5, 136.0, 130.6, 127.6, 127.5, 125.1, 123.3, 120.4, 61.6; **m/z LRMS** (ESI^+) 160.0 $[M + H]^+$. *Data is consistent with literature*[6]

***For 1-(Hydroxymethyl)isoquinoline 2-oxide (S20)*:** Prepared according to procedure **C**, using isoquinolin-1-ylmethanol **S19** (500 mg, 3.14 mmol, 1.00 equiv.), with *m*-CPBA (60% w/w, 1.08 g, 3.77 mmol, 1.20 equiv.) and stirring for 36 h. Purification by column chromatography (SiO_2, 3:1 to 1:3 CH_2Cl_2:Acetone) yielded 1-(hydroxymethyl)isoquinoline 2-oxide **S20** (296 mg, 1.69 mmol, 54%) as an off-white solid.

m.p 124-126 °C (CH_2Cl_2); **δ_H** (400 MHz, $CDCl_3$) 8.16 (1H, d, J = 7.1 Hz, *Ar*), 8.06-7.99 (1H, m, *Ar*), 7.82 (1H, d, J = 7.2 Hz, *Ar*), 7.73-7.59 (3H, m, *Ar*), 6.04-5.41 (1H, bs, O*H*), 5.27 (2H, s, C(7)H_2); **δ_C** (101 MHz, $CDCl_3$) 146.2, 136.3, 129.8, 129.7, 129.3, 127.6, 127.4, 123.7, 123.2, 57.1; **ν_{max}**/cm^{-1} ($CHCl_3$) 3221, 2981, 1333, 1278, 1215, 1179, 1143, 1066, 1012, 817, 750; **m/z HRMS** (ESI$^+$) [M + H]$^+$ $C_{10}H_{10}O_2N^+$ calc. 176.0706, found 176.0706.

For 1-Formylisoquinoline 2-oxide (1k): Prepared according to general procedure **D**, using 1-(hydroxymethyl)isoquinoline 2-oxide **S20** (270 mg, 1.54 mmol, 1.00 equiv.) and MnO_2 (1.14 g, 13.1 mmol, 8.50 equiv.), with stirring for 1 h. Purification by column chromatography (SiO_2, 4:1 CH_2Cl_2:Acetone) yielded 1-formylisoquinoline 2-oxide **1k** (185 mg, 1.07 mmol, 69%) as a yellow powdered solid.

m.p 134-136 °C (CH_2Cl_2); **δ_H** (400 MHz, $CDCl_3$) 10.81 (1H, s, C(7)*H*), 8.90 (1H, d, J = 8.7 Hz, *Ar*), 8.11 (1H, d, J = 7.1 Hz, *Ar*), 7.82 (1H, d, J = 7.1 Hz, *Ar*), 7.78 (1H, d, J = 8.1 Hz, *Ar*), 7.76-7.68 (1H, m, *Ar*), 7.66-7.57 (1H, m, *Ar*); **δ_C** (101 MHz, $CDCl_3$) 188.6, 138.8, 137.1, 131.9, 129.2, 128.7, 128.5, 127.9, 127.3, 123.8; **ν_{max}**/cm^{-1} ($CHCl_3$) 1686, 1623, 1498, 1432, 1317, 1230, 1141, 823, 751; **m/z HRMS** (ESI$^+$) [M + H]$^+$ $C_{10}H_8O_2N^+$ calc. 174.0550, found 174.0550. *Data is consistent with literature*[2]

3-(Hydroxymethyl)isoquinoline 2-oxide (S22) and 3-Formylisoquinoline-2-oxide (1l)

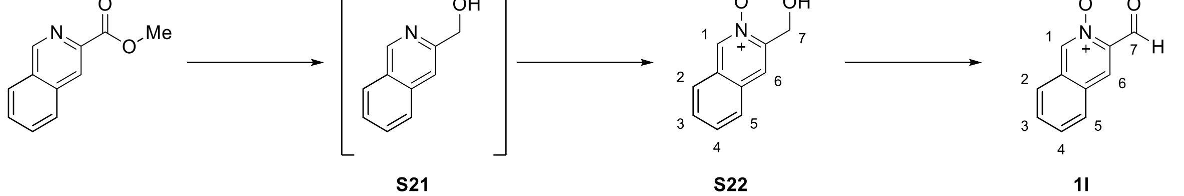

For 3-(Hydroxymethyl)isoquinoline 2-oxide (S22): Prepared according to general procedures **B** and **C**. Procedure **B** used methyl isoquinoline-3-carboxylate (500 mg, 2.67 mmol, 1.00

equiv.), $CaCl_2$ (1.19 g, 10.69 mmol, 4.00 equiv.) and $NaBH_4$ (253 mg, 6.68 mmol, 2.50 equiv.) with stirring for 1.5 h. Procedure **C** used crude alcohol **S21** obtained from procedure **B**, with *m*-CPBA (60% w/w, 920 mg, 3.20 mmol, 1.20 equiv.) and stirring for 20 h. After completion, Et_2O trituration gave 3-(hydroxymethyl)isoquinoline 2-oxide **S22** (397 mg, 2.27 mmol, 85% over two steps) as an off-white solid.

m.p 204-208 °C (Et_2O); **δ_H** (400 MHz, DMSO-d$_6$) 9.01 (1H, s, *Ar*), 8.02-7.96 (2H, m, *Ar*), 7.93-7.84 (1H, m, *Ar*), 7.66-7.55 (2H, m, *Ar*), 5.67 (1H, t, *J* = 5.7 Hz, O*H*), 4.71 (2H, dd, *J* = 5.6, 1.4 Hz, C(7)H_2); **δ_C** (101 MHz, DMSO-d$_6$) 148.5, 135.3, 128.4, 128.1, 128.1, 126.5, 124.5, 120.1, 58.1; **ν_{max}**/cm^{-1} ($CHCl_3$) 3247, 2972, 1320, 1207, 1170, 1103, 751; **m/z HRMS** (ESI$^+$) $[M + H]^+$ $C_{10}H_{10}O_2N^+$ calc. 176.0706, found 176.0707.

For 3-Formylisoquinoline 2-oxide (1l): Prepared according to general procedure **D**, using 3-(hydroxymethyl)isoquinoline 2-oxide **S22** (300 mg, 1.71 mmol, 1.00 equiv.) and MnO_2 (1.27 g, 14.57 mmol, 8.50 equiv.), with stirring for 2.5 h. ***After this time, starting material could still be observed by TLC, and so more MnO_2 (374 mg, 4.23 mmol, 2.5 equiv.) was added and the temperature was increased to 100 °C. TLC showed completion after 1 h at this temperature.*** Purification by column chromatography (SiO_2, 1:1 CH_2Cl_2:Acetone) yielded 3-formylisoquinoline 2-oxide **1l** (137 mg, 0.792 mmol, 46%) as a yellow solid.

m.p Decomp. at 220-224 °C (CH_2Cl_2); **δ_H** (400 MHz, $CDCl_3$) 10.80 (1H, s, C(7)*H*), 8.77 (1H, s, *Ar*), 8.30 (1H, s, *Ar*), 7.93 (1H, d, *J* = 8.1 Hz, *Ar*), 7.81-7.69 (2H, m, *Ar*), 7.69-7.61 (1H, m, *Ar*); **δ_C** (101 MHz, $CDCl_3$) 186.4, 140.2, 137.2, 132.0, 131.6, 130.0, 129.2, 128.2, 125.9, 125.1; **ν_{max}**/cm^{-1} ($CHCl_3$) 1692, 1623, 1599, 1317, 1179; **m/z HRMS** (ESI$^+$) $[M + H]^+$ $C_{10}H_8O_2N^+$ calc. 174.0550, found 174.0551. *Data is consistent with literature*[2]

2-(Hydroxymethyl)pyrazine 1-oxide (S23) and 2-Formylpyrazine 1-oxide (1m)

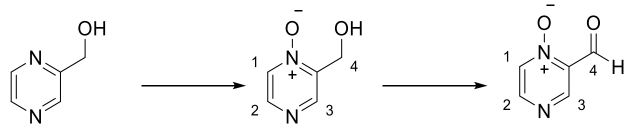

S23 **1m**

For 2-(Hydroxymethyl)pyrazine 1-oxide (S23): Prepared according to general procedure **C**, using 2-pyrazinylmethanol (404 mg, 3.67 mmol, 1.00 equiv.), with *m*-CPBA (60% w/w, 950 mg, 3.30 mmol, 0.900 equiv.) and stirring for 20 h. Purification by column chromatography (SiO_2, 1:1 to 1:3 CH_2Cl_2:Acetone) yielded pure 2-(hydroxymethyl)pyrazine 1-oxide **S23** (108 mg, 0.857 mmol, 23%) as an off-white solid.

m.p 89-91 °C (Et_2O); δ_H (400 MHz, Acetone-d_6) 8.67 (1H, s, *Ar*), 8.46 (1H, d, $J = 4.1$ Hz, *Ar*), 8.21 (1H, dd, $J = 4.1, 0.7$ Hz, *Ar*), 4.78 (3H, app. s, C(4)H_2 and O*H*); δ_C (101 MHz, Acetone-d_6) 147.3, 146.9, 146.5, 134.2, 58.3; ν_{max}/cm^{-1} ($CHCl_3$) 3252, 3108, 1592, 1456, 1437, 1402, 1248, 1197, 1174, 1072, 1049, 864, 847, 838, 694; **m/z HRMS** (ESI$^+$) [M + H]$^+$ $C_5H_7O_2N_2^+$ calc. 127.0502, found 127.0503.

2-Formylpyrazine 1-oxide (1m): Prepared according to general procedure **D**, using 2-(hydroxymethyl)pyrazine 1-oxide **S23** (108 mg, 0.857 mmol, 1.00 equiv.) and MnO_2 (633 mg, 7.28 mmol, 8.50 equiv.), with stirring for 3 h. Purification by column chromatography (SiO_2, 3:1 CH_2Cl_2:Acetone) yielded 2-formylpyrazine 1-oxide **1m** (42.3 mg, 0.341 mmol, 40%) as an off-white powdered solid.

m.p 116-118 °C (CH_2Cl_2); δ_H (400 MHz, Acetone-d_6) 10.44 (1H, s, C(4)*H*), 8.81 (1H, s, *Ar*), 8.69 (1H, d, $J = 4.3$ Hz, *Ar*), 8.32 (1H, dd, $J = 4.2, 0.8$ Hz, *Ar*); δ_C (101 MHz, Acetone-d_6) 185.8, 152.0, 148.0, 140.1, 135.9; ν_{max}/cm^{-1} ($CHCl_3$) 2924, 1676, 1586, 1445, 1412, 1381,

1306, 1275, 1195, 1056, 871, 809, 661; **m/z HRMS** (ESI^+) $[M + H]^+$ $C_5H_5O_2N_2^+$ calc. 125.0346, found 125.0347.

Pyridazine 1-oxide (S24), 6-(Prop-1-en-1-yl)pyridazine 1-oxide (S25) and 6-Formylpyridazine 1-oxide (1n)

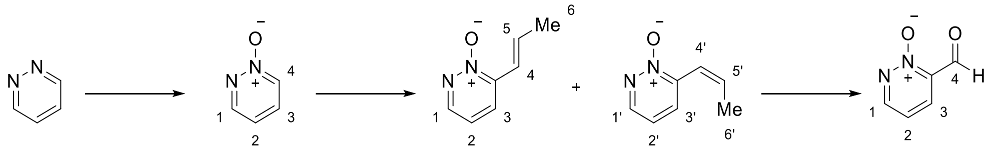

For Pyridazine 1-oxide (S24): According to a modified procedure,[7] a solution of pyridazine (0.72 mL, 10.0 mmol, 1.00 equiv.), in CH_2Cl_2 (50 mL) was cooled to 0 °C. *m*-CPBA (60% w/w, 2.88 g, 10.0 mmol, 1.00 equiv.) was added portion-wise before the resulting mixture was warmed to room temperature and stirred overnight. Once TLC showed completion (18 h), PPh_3 (1.31 g, 5.00 mmol, 0.500 equiv.) was added slowly, and the resulting mixture was stirred for a further 4 h. After this time, celite was added to the mixture and the solvent was removed *in vacuo*. The resulting celite-bound residue was loaded directly onto a flash column (SiO_2, neat EtOAc to 9:1 EtOAc:MeOH) to yield pyridazine 1-oxide **S24** (935 mg, 9.74 mmol, 97%) as a pink solid.

m.p 39-40 °C (CH_2Cl_2); {*Lit:* 39-40 °C}[8]; **δ_H** (400 MHz, $CDCl_3$) 8.46-8.40 (1H, m, *Ar*), 8.13 (1H, d, J = 6.5 Hz, *Ar*), 7.66-7.57 (1H, m, *Ar*), 7.05 (1H, ddd, J = 7.7, 5.4, 0.9 Hz, *Ar*); **δ_C** (101 MHz, $CDCl_3$) 150.6, 134.4, 134.2, 116.0. *Data is consistent with literature*[7]

For 6-(Prop-1-en-1-yl)pyridazine-1-oxide (S25): According to a modified procedure,[7] in an oven-dried microwave vial was added pyridazine 1-oxide **S24** (96.1 mg, 1.00 mmol, 2.00 equiv.), $Pd(OAc)_2$ (11.2 mg, 0.050 mmol, 0.100 equiv.), $P(t\text{-}Bu)_3{\cdot}HBF_4$ (43.5 mg, 0.150 mmol, 0.300 equiv.) and KF (58 mg, 1.00 mmol, 2.00 equiv.). The vial was sealed with a microwave

cap and vac-cycled under Ar (×3). Dry and degassed THF (2 mL, 0.25 M) was then added, followed by allyl acetate (54 µL, 0.500 mmol, 1.00 equiv.) and the resulting mixture was heated to 100 °C for 20 h. Once complete, the mixture was cooled to room temperature and the solvent was removed *in vacuo*. Purification of the crude residue by column chromatography (SiO_2, 1:1 EtOAc:Petrol to neat EtOAc) yielded a 9.4:1 inseparable *E*/*Z* mixture of 6-(prop-1-en-1-yl)pyridazine-1-oxide **S25** (42.2 mg, 0.310 mmol, 62%) as a pale-red oil.

δ_H (400 MHz, $CDCl_3$) 8.40-8.35 (1H, m, *Ar'*), 8.31 (1H, dd, *J* = 5.2, 2.4 Hz, *Ar*), 7.71 (1H, dd, *J* = 8.1, 2.4 Hz, *Ar*), 7.65 (1H, dd, *J* = 7.9, 2.5 Hz, *Ar'*), 7.09-7.02 (1H, m, *Ar'*), 7.01 (1H, ddd, *J* = 8.1, 5.2, 0.6 Hz, *Ar*), 6.91 (1H, dq, *J* = 16.1, 1.5 Hz, C(4)*H*), 6.73-6.61 (1H, m, C(4')*H*), 6.69 (1H, dq, *J* = 16.1, 6.7 Hz, C(5)*H*), 6.22 (1H, dq, *J* = 11.8, 7.3 Hz, C(5')*H*), 2.00 (3H, dd, *J* = 6.7, 1.7 Hz, C(6)H_3), 1.90 (3H, dd, *J* = 7.3, 1.9 Hz, C(6')H_3); **δ_C** (101 MHz, $CDCl_3$) 148.4*, 148.0, 142.9 (*E* and *Z*), 136.0, 134.1*, 133.7*, 130.6, 121.6, 120.9*, 116.3, 115.6*, 19.2, 14.9* (* denotes the minor *Z* isomer ***Z*-S25**); **m/z LRMS** (ESI^+) 137.0 $[M + H]^+$. *Data is consistent with literature*[7]

For 6-Formylpyridazine 1-oxide (1n): According to a modified procedure,[7] in a dry two-neck flask, a 9.4:1 E/*Z* mixture of 6-(prop-1-en-1-yl)pyridazine 1-oxide **S25** (42.2 mg, 0.310 mmol, 1.00 equiv.) was dissolved in a 1:1 CH_2Cl_2:MeOH solvent mixture (2.50 mL, 0.125 M). The solution was then cooled to -78 °C and ozone was bubbled through for 30 min with stirring. After this time, dimethyl sulfide (114 µL, 1.55 mmol, 5.00 equiv.) was added and the solution was warmed to room temperature with stirring for 2 h. The resulting mixture was then concentrated *in vacuo*. Purification of the crude residue by column chromatography (SiO_2, 1:4 EtOAc:Pentane to neat EtOAc) yielded 6-formylpyridazine 1-oxide **1n** (25.9 mg, 0.208 mmol, 67%) as a white powdered solid.

m.p 126-129 °C (CH_2Cl_2); **δ_H** (400 MHz, $CDCl_3$) 10.42 (1H, d, *J* = 0.8 Hz, C(4)*H*), 8.61 (1H, dd, *J* = 5.6, 2.5 Hz, *Ar*), 8.08 (1H, dd, *J* = 7.9, 2.6 Hz, *Ar*), 7.17 (1H, ddd, *J* = 8.0, 5.3, 0.9 Hz, *Ar*); **δ_C** (101 MHz, $CDCl_3$) 184.8, 153.7, 138.8, 133.2, 116.2; **ν_{max}**/cm^{-1} ($CHCl_3$) 2923, 1697, 1683, 1590, 1536, 1384, 1300, 1261, 1023, 1013, 881, 807, 742, 733, 645; **m/z HRMS** (ESI$^+$) [M + H]$^+$ $C_5H_5O_2N_2^+$ calc. 125.0346, found 125.0347.

2-(Hydroxymethyl)quinoline 1-oxide (S27) and 2-Formylquinoline 1-oxide (1q)

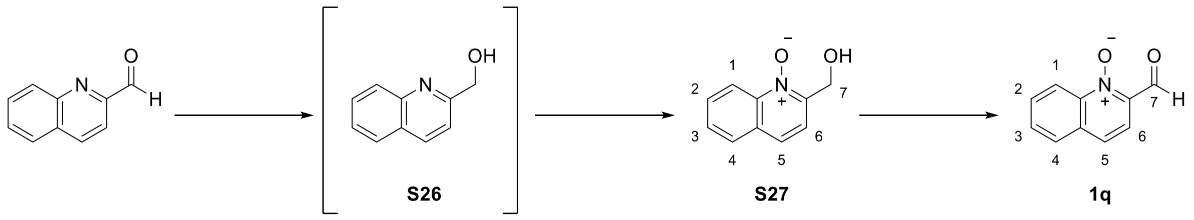

For 2-(Hydroxymethyl)quinoline 1-oxide (S27): Prepared according to general procedures **A** and **C**. Procedure **A** used quinoline carboxaldehyde (800 mg, 5.10 mmol, 1.00 equiv.) and $NaBH_4$ (213 mg, 5.61 mmol, 1.10 equiv.) with stirring for 1 h. Procedure **C** used crude alcohol **S26** obtained from procedure **A**, with *m*-CPBA (60% w/w, 1.75 g, 6.12 mmol, 1.20 equiv.) and stirring for 12 h. After completion, Et_2O trituration gave pure 2-(hydroxymethyl)quinoline 1-oxide **S27** (420 mg, 2.40 mmol, 47% yield over two steps) as an off white solid.

m.p 109-113 °C (Et_2O); **δ_H** (400 MHz, $CDCl_3$) 8.70 (1H, d, *J* = 8.8 Hz, *Ar*), 7.88-7.82 (1H, m, *Ar*), 7.82-7.73 (2H, m, *Ar*), 7.67-7.59 (1H, m, *Ar*), 7.43 (1H, d, *J* = 8.5 Hz, *Ar*), 5.18 (1H, bs, O*H*), 5.01 (2H, s, C(7)*H*$_2$); **δ_C** (101 MHz, $CDCl_3$) 147.3, 141.3, 131.0, 129.6, 128.6, 128.3, 127.1, 120.5, 119.1, 62.0; **ν_{max}**/cm^{-1} ($CHCl_3$) 3222, 2981, 2886, 1340, 1229, 1211, 1185, 1094, 1066, 813, 768, 736; **m/z HRMS** (ESI$^+$) [M + H]$^+$ $C_{10}H_{10}O_2N^+$ calc. 176.0706, found 176.0707.

For 2-Formylquinoline 1-oxide (1q): Prepared according to general procedure **D**, using 2-(hydroxymethyl)quinoline 1-oxide **S27** (400 mg, 2.28 mmol) and MnO_2 (1.69 g, 19.2 mmol, 8.50 equiv.) with stirring for 2 h. Purification by column chromatography (SiO_2, from 1:1 to 3:1 EtOAc: Petrol) yielded 2-formylquinoline 1-oxide **1q** (200 mg, 1.15 mmol, 50% yield) as a yellow/orange solid.

m.p 103-105 °C (CH_2Cl_2); $\boldsymbol{\delta_H}$ (400 MHz, $CDCl_3$) 10.83 (1H, d, J = 0.8 Hz, C(7)*H*), 8.78-8.73 (1H, m, *Ar*), 7.88 (1H, dd, J = 8.0, 1.5 Hz, *Ar*), 7.82-7.68 (4H, m, *Ar*); $\boldsymbol{\delta_C}$ (101 MHz, $CDCl_3$) 186.5, 142.3, 140.4, 132.2, 131.1, 131.1, 128.6, 124.8, 120.1, 119.1; $\boldsymbol{\nu_{max}}$/cm^{-1} ($CHCl_3$) 3104, 3060, 1696, 1676, 1448, 1371, 1227, 1208, 824, 770; **m/z HRMS** (ESI^+) $[M + H]^+$ $C_{10}H_8O_2N^+$ calc. 174.0550, found 174.0550. *Data is consistent with literature*[2]

Quinolin-8-ylmethanol (S28), 8-(Hydroxymethyl)quinoline 1-oxide (S29) and 8-Formylquinoline 1-oxide (1r)

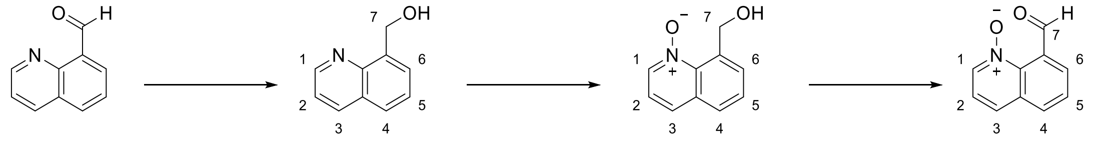

For Quinolin-8-ylmethanol (S28): Prepared according to general procedure **A**, using quinoline-8-carbaldehyde (1.00 g, 6.37 mmol, 1.00 equiv.), $NaBH_4$ (265 mg, 7.00 mmol, 1.10 equiv.) with stirring for 1.5 h. Purification by column chromatography (SiO_2, 1:9 to 1:1 EtOAc:Petrol) yielded quinolin-8-ylmethanol **S28** (622 mg, 3.91 mmol, 61%), as an off-white solid.

m.p 73-75 °C ($CHCl_3$); $\boldsymbol{\delta_H}$ (400 MHz, $CDCl_3$) 8.87 (1H, dd, J = 4.2, 1.8 Hz, *Ar*), 8.20 (1H, dd, J = 8.3, 1.8 Hz, *Ar*), 7.76 (1H, dd, J = 8.2, 1.5 Hz, *Ar*), 7.61-7.57 (1H, m, *Ar*), 7.49 (1H, dd, J

= 8.2, 7.0 Hz, *Ar*), 7.44 (1H, dd, *J* = 8.3, 4.2 Hz, *Ar*), 5.21 (2H, s, C(7)H_2), 5.03 (1H, s, O*H*); **δ$_C$** (101 MHz, $CDCl_3$) 149.1, 147.2, 138.3, 137.0, 128.6, 127.9, 127.6, 126.6, 121.3, 64.9.

For 8-(Hydroxymethyl)quinoline 1-oxide (S29): Prepared according to procedure **C**, using quinolin-8-ylmethanol **S28** (622 mg, 3.91 mmol, 1.00 equiv.), with *m*-CPBA (60% w/w, 1.35 g, 4.69 mmol, 1.20 equiv.) and stirring for 20 h. Purification by column chromatography (SiO_2, 1:1 EtOAc:Pentane to 9:1 EtOAc:Methanol) yielded 8-(hydroxymethyl)quinoline 1-oxide **S29** (518 mg, 2.96 mmol, 76%) as an off-white solid.

m.p Decomp. at 108-110 °C (CH_2Cl_2); **δ$_H$** (400 MHz, $CDCl_3$) 8.58 (1H, dd, *J* = 6.1, 1.2 Hz, *Ar*), 7.82 (1H, ddd, *J* = 7.5, 5.9, 1.4 Hz, *Ar*), 7.61 (1H, dd, *J* = 7.1, 1.6 Hz, *Ar*), 7.54 (1H, t, *J* = 7.6 Hz, *Ar*), 7.33 (1H, dd, *J* = 8.4, 6.1 Hz, *Ar*), 5.88 (1H, bs, O*H*), 5.10 (2H, s, C(7)H_2); **δ$_C$** (101 MHz, $CDCl_3$) 141.0, 138.3, 135.1, 133.4, 132.7, 129.3, 128.9, 128.7, 121.0, 66.4; **ν$_{max}$**/cm^{-1} ($CHCl_3$) 3364, 1577, 1422, 1387, 1301, 1206, 1162, 1006, 814, 757; **m/z HRMS** (ESI$^+$) $[M + H]^+$ $C_{10}H_{10}O_2N^+$ calc. 176.0706, found 176.0708.

For 8-Formylquinoline 1-oxide (1r): Prepared according to general procedure **D**, using 8-(Hydroxymethyl)quinoline 1-oxide **S29** (300 mg, 1.71 mmol, 1.00 equiv.) and MnO_2 (1.27 g, 14.6 mmol, 8.50 equiv.), ***with initial stirring at 55 °C for 1.5 h. However, conversion was incomplete after this time, and so the temperature was increased to 90 °C for 1 h.*** Purification by column chromatography (SiO_2, 9:1 to 7:3 CH_2Cl_2:Acetone) yielded 8-formylquinoline 1-oxide **1r** (191 mg, 1.10 mmol, 65%) as a yellow powdered solid.

m.p 140-142 °C (CH_2Cl_2); **δ$_H$** (400 MHz, $CDCl_3$) 11.21 (s, 1H), 8.51 (dd, *J* = 6.0, 1.1 Hz, 1H), 8.00 (dd, *J* = 8.2, 1.4 Hz, 1H), 7.88 (dd, *J* = 7.2, 1.4 Hz, 1H), 7.82 (dd, *J* = 8.4, 1.0 Hz, 1H), 7.73 – 7.65 (m, 1H), 7.37 (dd, *J* = 8.4, 6.0 Hz, 1H); **δ$_C$** (101 MHz, $CDCl_3$) 191.2, 139.8, 136.5, 133.7, 131.8, 131.3, 131.1, 128.8, 126.7, 122.0; **ν$_{max}$**/cm^{-1} ($CHCl_3$) 2885, 1679, 1311, 1226,

1159, 1124, 845, 812, 760; **m/z HRMS** (ESI$^+$) $[M + H]^+$ $C_{10}H_8O_2N^+$ calc. 174.0550, found 174.0552.

2.3 Alkyne Substrates

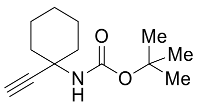

S34

Synthesised according to a standard literature procedure[10]

3. Reaction Optimisation

Table 1: Ligand Evaluation (0.2 mmol scale)

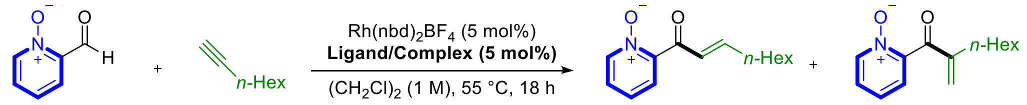

1a (1.0 equiv.) (1.2 equiv.) **2a** (l) **3a** (b)

Entry	Ligand/ Complex	Aldehyde consumption (%)^c	l:b	2a + 3a (%)^{d,e}
1	dcpe	>99	5.6:1	22
2	dppe	>99	>20:1	25
3	dcpm	86	-	0
4[a]	PPh_3	64	-	0
5	DPEPhos	>99	2.8:1	53(54)
6	*rac*-BINAP	>99	1:1	40
7	dppbenz	>99	>20:1	17
8	dppf	>99	1.9:1	61(67)
9[b]	dppf	94	1.3:1	43
10	DCEPhos	33	5.1:1	2
11	Xantphos	>99	>20:1	11
12	*t*-Bu-Xantphos	35	-	0
13	dppb	82	14:1	15
14	dppp	>99	>20:1	17

[a] 10 mol% PPh_3 used; [b] 2.5 mol% $[Rh(COD)Cl]_2$ pre-catalyst was used instead of $Rh(nbd)_2BF_4$; [c] Aldehyde consumption was determined by crude ^{1}H NMR spectroscopy; [d] ^{1}H NMR yields were based on ^{1}H NMR standard (methyl-3,5-dinitrobenzoate); [e] Isolated yields in parentheses.

Table 2: Shorter Reaction Times for dppf and DPEPhos (0.2 mmol scale)

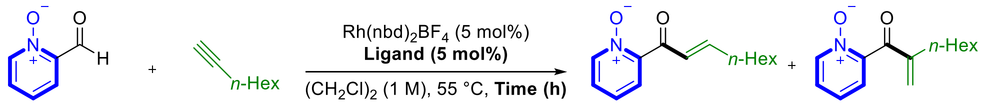

1a 1.0 equiv. 1.2 equiv. **2a** (l) **3a** (b)

Entry	Ligand	Time (h)	Aldehyde consumption (%)^a	l:b	2 + 3 (%)^{b,c}
1	dppf	4	>99	2.1:1	74(66)
2	DPEPhos	6	55	2.5:1	27

[a] Aldehyde consumption was determined by crude ^{1}H NMR spectroscopy, [b] ^{1}H NMR yields were based on ^{1}H NMR standard (methyl-3,5-dinitrobenzoate), [c] Isolated yields in parentheses.

Table 3: Solvent Alteration with dppf in an Attempt to Improve Regioselectivity (0.2 mmol scale)

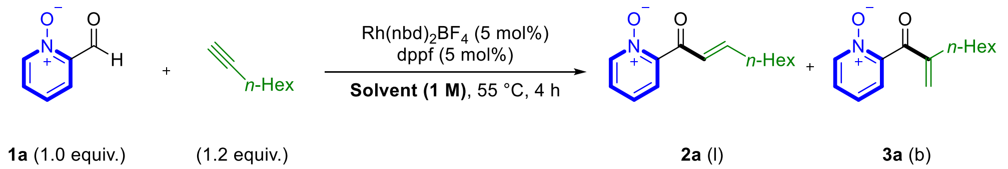

Entry	Solvent	Aldehyde consumption (%)[a]	l:b	l + b (%)[b,c]
1	MeCN	97	1.8:1	65
2	CH_2Cl_2	49	2:1	28
3	*t*-BuCN	73	1.8:1	40

[a] Aldehyde consumption was determined by crude ^{1}H NMR spectroscopy, [b] ^{1}H NMR yields were based on ^{1}H NMR standard (methyl-3,5-dinitrobenzoate), [c] Isolated yields in parentheses.

Table 4: Higher Alkyne Loading with DPEPhos Ligand (0.2 mmol scale)

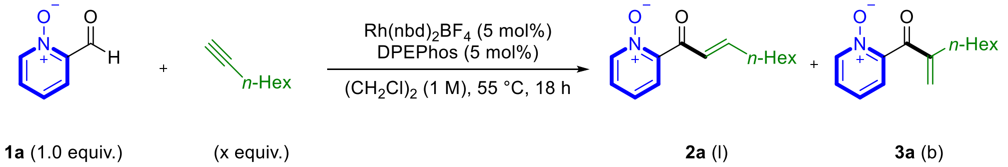

Entry	Alkyne (equiv.)	Aldehyde consumption (%)[a]	l:b	l + b (%)[b,c]
1	1.2	>99	2.8:1	53(54)
2	1.5	>99	2.8:1	64
3	2.0	>99	2.8:1	67
4	3.0	>99	2.4:1	59

[a] Aldehyde consumption was determined by crude ^{1}H NMR spectroscopy; [b] ^{1}H NMR yields were based on ^{1}H NMR standard (methyl-3,5-dinitrobenzoate); [c] Isolated yields in parentheses.

Table 5: Lower Temperature Reactions (0.2 mmol scale)

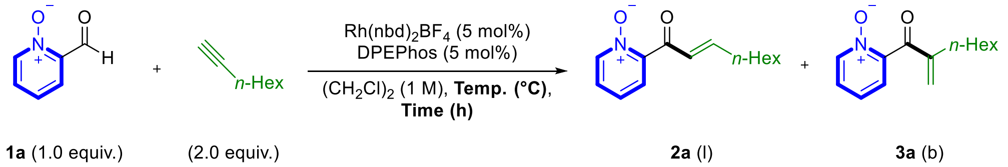

Entry	Time (h)	Temp. (°C)	Aldehyde consumption (%)[a]	l:b	l + b (%)[b,c]
1	18	35	57	3.7:1	34
2	18	rt	45	4.6:1	30
3	48	rt	47	6.7:1	34

[a] Aldehyde consumption determined by crude ^{1}H NMR spectroscopy; [b] ^{1}H NMR yields based on ^{1}H NMR standard (methyl-3,5-dinitrobenzoate); [c] Isolated yields in parentheses.

Table 6: Stoichiometry-switch Whilst Maintaining 1 M Aldehyde Concentration (0.2 mmol scale):

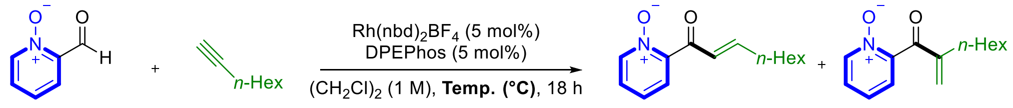

1a (2.0 equiv.) (1.0 equiv.) **2a** (l) **3a** (b)

Entry	Temp (°C)	l:b	l + b (%)[a,b]
1	55	2.1:1	26
2	rt	5.9:1	17

[a] ^{1}H NMR yields were based on ^{1}H NMR standard (methyl-3,5-dinitrobenzoate), [b] Isolated yields in parentheses.

Table 7: Higher Scale (0.3 mmol) Reactions

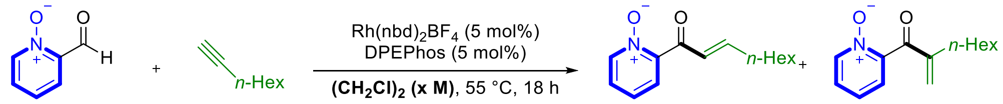

1a 1.0 equiv. 2.0 equiv. **2a** (l) 3a (b)

Entry	Aldehyde conc. (M)	Aldehyde consumption (%)[a]	l:b	l + b (%)[b,c]
1	1.0	>99	2.9:1	57
2	1.25	>99	3.1:1	73(76)
3	1.5	>99	2.8:1	68

[a] Reaction conversion was determined by crude ^{1}H NMR spectroscopy; [b] ^{1}H NMR yields were based on ^{1}H NMR standard (methyl-3,5-dinitrobenzoate); [c] Isolated yields in parentheses.

Table 8: Shorter Reaction Times at 1.25 M conc. with DPEPhos Ligand (0.3 mmol scale):

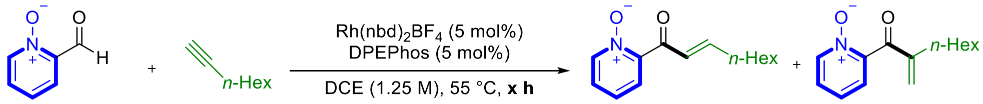

1a (1.0 equiv.) (2.0 equiv.) **2a** (l) **3a** (b)

Entry	Time (h)	Aldehyde Consumption (%)[a]	l:b	l + b (%)[b,c]
1	4	82	2.9:1	66
2	5	98	2.8:1	76
3	6	>99	2.9:1	79(73)

[a] Reaction conversion was determined by crude ^{1}H NMR spectroscopy; [b] ^{1}H NMR yields were based on ^{1}H NMR standard (methyl-3,5-dinitrobenzoate); [c] Isolated yields in parentheses.

Table 9: Varying the Reaction Solvent with DPEPhos Ligand (0.3 mmol scale):

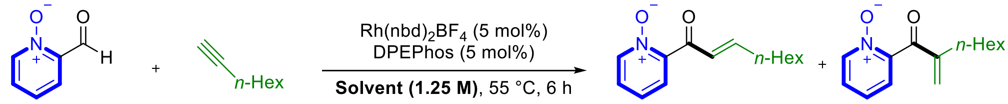

1a (1.0 equiv.) (2.0 equiv.) **2a** (l) **3a** (b)

Entry	Solvent	Aldehyde consumption (%)[a]	l:b	l + b (%)[b,c]
1	Acetone	40	1.2:1	29
2	CH_2Cl_2	75	2.7:1	45
3	MeCN	>99	1.8:1	72
4	1:1 DCE:Dioxane	53	2.1:1	25
5	1:1 DCE:THF	54	2:1	25
6	1:1 DCE:Toluene	53	2.3:1	27
7	1:1 DCE:*o*-Xylene	65	2.3:1	27

[a] Reaction conversion was determined by crude ^1H NMR spectroscopy; [b] ^1H NMR yields were based on ^1H NMR standard (methyl-3,5-dinitrobenzoate); [c] Isolated yields in parentheses.

4. Rhodium-catalysed Hydroacylation

4.1 General procedure E - Optimised Conditions for *N*-Oxide-Directed Intramolecular Alkyne Hydroacylation

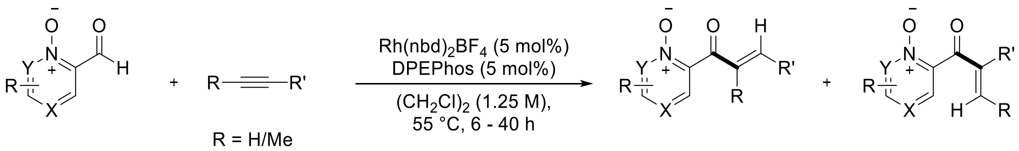

1 (1.00 equiv.) (2.00 equiv.) **2** **3**

[Rh(nbd)$_2$]BF$_4$ (0.015 mmol, 5 mol%) was weighed into a tapered, 10 mL microwave vial. Next, DPEPhos (0.015 mmol, 5 mol%) was introduced and the vial sealed with a microwave cap. The vial was placed under vacuum on a Schlenck line and back-filled with argon three times. After this, 1.0 mL of distilled $(CH_2Cl)_2$ was introduced, and the solution sonicated for 30 s. Hydrogen gas was bubbled through the solution for 1-3 min. The solvent was then removed using a stream of argon and the catalyst system re-dissolved in $(CH_2Cl)_2$ (1.25 M). Aldehyde (0.300 mmol, 1.00 equiv) was weighed into a separate concave, 10 mL microwave vial, alongside alkyne (0.600 mmol, 2.00 equiv), if a solid. The vial was sealed with a suba-seal/microwave cap and para-filmed (if suba-sealed). It was then placed under vacuum on a Schlenck line and back-filled with argon three times. ***If the alkyne was a liquid, it was then syringed into the vial post vac-cycle.*** The catalyst solution was then added to the reactant mixture. The resulting solution was then stirred at 55 °C for 6-40 h. Once complete by TLC, the reaction mixture was cooled to room temperature and concentrated *in vacuo*. Purification by column chromatography (SiO_2) yielded the enone products.

4.2 Alkyne Scope

(*E*)-2-(Non-2-enoyl)pyridine 1-oxide (2a) and 2-(2-Methyleneoctanoyl)pyridine 1-oxide (3a)

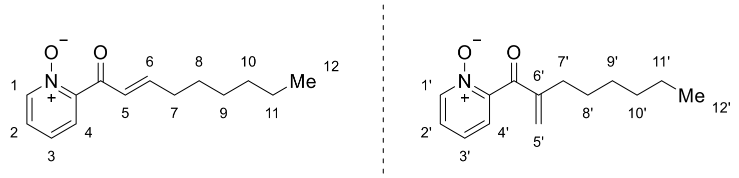

2a **3a**

Prepared according to general procedure **E**, using 2-formylpyridine 1-oxide **1a** (37 mg, 0.300 mmol, 1.00 equiv.), 1-octyne (87 μL, 0.600 mmol, 2.00 equiv.), $Rh(nbd)_2BF_4$ (5.6 mg, 0.015 mmol, 0.050 equiv.), DPEPhos (8.1 mg, 0.015 mmol, 0.050 equiv.) and $(CH_2Cl)_2$ (0.24 mL), with stirring for 6 h. Purification of the crude residue (2.9:1 l:b) by column chromatography (SiO_2, 1:1 EtOAc:Petrol to neat EtOAc) yielded (*E*)-2-(non-2-enoyl)pyridine 1-oxide **2a** (37.3 mg, 0.160 mmol, 53%) and 2-(2-methyleneoctanoyl)pyridine 1-oxide **3a** (14 mg, 0.06 mmol, 20%) both as yellow viscous oils.

(E)-2-(Non-2-enoyl)pyridine 1-oxide (2a)**:** δ_H (400 MHz, $CDCl_3$) 8.22–8.15 (1H, m, *Ar*), 7.58–7.47 (1H, m, *Ar*), 7.38–7.27 (2H, m, *Ar*), 7.04 (1H, dt, *J* = 15.6, 6.6 Hz, C(6)*H*), 6.93 (1H, dt, *J* = 15.5, 1.2 Hz, C(5)*H*), 2.27 (2H, app. dtd, *J* = 7.8, 6.7, 1.3 Hz, C(7)H_2), 1.53-1.41 (2H, m, C(8)H_2), 1.41–1.14 (6H, m, C(9 to 11)H_2), 0.93–0.75 (3H, m, C(12)H_3); δ_C (101 MHz, $CDCl_3$) 187.0, 150.7, 147.4, 140.4, 128.1, 127.5, 126.9, 125.7, 32.8, 31.7, 29.0, 28.0, 22.6, 14.1; ν_{max}/cm^{-1} ($CHCl_3$) 2955, 2926, 1667, 1619, 1426, 1297, 1247, 1197, 1031, 969, 859; **m/z HRMS** (ESI$^+$) $[M + H]^+$ $C_{14}H_{20}O_2N^+$ calc. 234.1489, found 234.1490.

2-(2-Methyleneoctanoyl)pyridine 1-oxide (3a)**:** δ_H (400 MHz, $CDCl_3$) 8.25–8.16 (1H, m, *Ar'*), 7.38–7.31 (2H, m, *Ar'*), 7.29–7.25 (1H, m, *Ar'*), 5.93 (1H, app. t, *J* = 1.4 Hz, C(5')*H*H), 5.66 (1H, s, C(5')H*H*), 2.49–2.41 (2H, m, C(7')H_2), 1.60–1.49 (2H, m, C(8')H_2), 1.41–1.26 (6H, m, C(9' to 11')H_2), 0.9–0.85 (3H, m, C(12')H_3); δ_C (101 MHz, $CDCl_3$) 191.4, 148.1,

147.4, 140.2, 127.8, 126.7, 125.5, 125.1, 31.8, 30.0, 29.1, 28.0, 22.7, 14.2; **ν_{max}**/cm^{-1} ($CHCl_3$) 2954, 2927, 2856, 1627, 1426, 1247, 987, 847, 769; **m/z HRMS** (ESI^+) $[M + H]^+$ $C_{14}H_{20}O_2N^+$ calc. 234.1489, found 234.1489.

(*E*)-2-(3-Cyclohexylacryloyl)pyridine 1-oxide (2b)

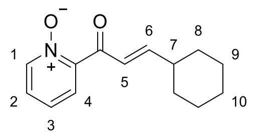

2b

Prepared according to general procedure **E**, using 2-formylpyridine 1-oxide **1a** (37 mg, 0.300 mmol, 1.00 equiv.), ethynylcyclohexane (78 µL, 0.600 mmol, 2.00 equiv.), $Rh(nbd)_2BF_4$ (5.6 mg, 0.015 mmol, 0.050 equiv.), DPEPhos (8.1 mg, 0.015 mmol, 0.050 equiv.) and $(CH_2Cl)_2$ (0.24 mL), with stirring for 18 h. Purification of the crude residue (>20:1 l:b) by column chromatography (SiO_2, 1:1 to 4:1 EtOAc:Petrol) yielded single isomer (*E*)-2-(3-cyclohexylacryloyl)pyridine 1-oxide **2b** (51.1 mg, 0.221 mmol, 74%) as a yellow viscous oil.

δ_H (400 MHz, $CDCl_3$) 8.16 (1H, dd, *J* = 6.1, 1.4 Hz, *Ar*), 7.51 (1H, dd, *J* = 7.5, 2.4 Hz, *Ar*), 7.36-7.26 (2H, m, *Ar*), 6.97 (1H, dd, *J* = 15.7, 6.2 Hz, C(6)*H*), 6.89 (1H, d, *J* = 15.9 Hz, C(5)*H*), 2.26-2.13 (1H, m, C(7)*H*), 1.84-1.67 (4H, m, C(8)*H₂*), 1.67-1.58 (1H, m, C(10)*H*H), 1.34-1.06 (5H, m, C(9)H_2 and C(10)H*H*); **δ_C** (101 MHz, $CDCl_3$) 187.3, 155.0, 147.4, 140.4, 127.5, 126.9, 125.8, 125.6, 40.9, 31.6, 26.0, 25.7; **ν_{max}**/cm^{-1} ($CHCl_3$) 2925, 2851, 1668, 1618, 1427, 1332, 1317, 1297, 1248, 770; **m/z HRMS** (ESI^+) $[M + H]^+$ $C_{14}H_{19}O_2N^+$ calc. 232.1332, found 232.1332. *Data is consistent with literature*[11]

(*E*)-2-(3-Cyclopentylacryloyl)pyridine 1-oxide (2c) and 2-(2-Cyclopentylacryloyl)-pyridine 1-oxide (3c)

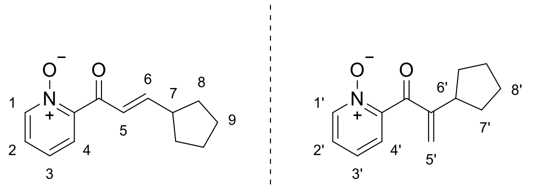

2c **3c**

Prepared according to general procedure **E**, using 2-formylpyridine 1-oxide **1a** (37 mg, 0.300 mmol, 1.00 equiv.), ethynylcyclopentane (70 μL, 0.600 mmol, 2.00 equiv.), $Rh(nbd)_2BF_4$ (5.6 mg, 0.015 mmol, 0.050 equiv.), DPEPhos (8.1 mg, 0.015 mmol, 0.050 equiv.) and $(CH_2Cl)_2$ (0.24 mL), with stirring for 20 h. Purification of the crude residue (18:1 l:b) by column chromatography (SiO_2, 1:1 to 7:3 EtOAc:Pentane) yielded (*E*)-2-(3-cyclopentylacryloyl)pyridine 1-oxide **2c** (33.6 mg, 0.155 mmol, 51%) and 2-(2-cyclopentylacryloyl)-pyridine 1-oxide **3c** (3.00 mg, 0.014 mmol, 5%) both as yellow viscous oils.

(E)-2-(3-Cyclopentylacryloyl)pyridine 1-oxide (2c)**:** δ_H (400 MHz, $CDCl_3$) 8.20-8.13 (1H, m, *Ar*), 7.52 (1H, dd, *J* = 7.5, 2.4 Hz, *Ar*), 7.31 (2H, ddd, *J* = 9.0, 6.7, 1.8 Hz, *Ar*), 7.02 (1H, dd, *J* = 15.5, 7.6 Hz, C(6)*H*), 6.93 (1H, d, *J* = 15.6 Hz, C(5)*H*), 2.67 (1H, app. hex, *J* = 7.9 Hz, C(7)*H*), 1.92-1.77 (2H, m, C(8 and 9)H_2), 1.74-1.50 (4H, m, C(8 and 9)H_2), 1.50-1.35 (2H, m, C(8 and 9)H_2); δ_C (101 MHz, $CDCl_3$) 187.1, 154.6, 147.4, 140.4, 127.5, 126.9, 126.2, 125.6, 43.3, 32.5, 25.5; ν_{max}/cm^{-1} ($CHCl_3$) 2951, 2867, 1665, 1615, 1426, 1314, 1294, 1247, 1029, 981, 771; **m/z HRMS** (ESI$^+$) [M + H]$^+$ $C_{13}H_{16}O_2N^+$ calc. 218.1176, found 218.1177.

2-(2-Cyclopentylacryloyl)-pyridine 1-oxide (3c)**:** δ_H (500 MHz, $CDCl_3$) 8.18 (1H, dd, *J* = 4.9, 2.7 Hz, *Ar'*), 7.37-7.32 (2H, m, *Ar'*), 7.32-7.27 (1H, m, *Ar'*), 5.90 (1H, d, *J* = 1.5 Hz, C(5')*H*H), 5.61 (1H, app. s, C(5')H*H*), 3.14-3.04 (1H, m, C(6')*H*), 2.04-1.96 (2H, m, C(7' and 8')H_2), 1.77-1.70 (2H, m, C(7' and 8')H_2), 1.69-1.61 (2H, m, C(7' and 8')H_2), 1.59-1.46 (2H, m, C(7'

and 8')H_2); **δC** (126 MHz, $CDCl_3$) 191.8, 151.8, 147.6, 140.2, 126.7, 125.5, 125.2, 125.1, 39.9, 31.8, 25.1; **νmax**/cm^{-1} ($CHCl_3$) 2953, 2924, 2868, 1674, 1427, 1248, 990, 850, 770; **m/z HRMS** (ESI^+) $[M + H]^+$ $C_{13}H_{16}O_2N^+$ calc. 218.1176, found 218.1176.

(*E*)-2-(4,4-Dimethylpent-2-enoyl)pyridine 1-oxide (2d)

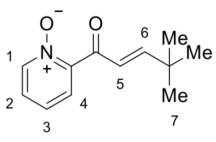

2d

Prepared according to general procedure **E**, using 2-formylpyridine 1-oxide **1a** (37 mg, 0.300 mmol, 1.00 equiv.), 3,3-dimethylbut-1-yne (74 μL, 0.600 mmol, 2.00 equiv.), $Rh(nbd)_2BF_4$ (5.6 mg, 0.015 mmol, 0.050 equiv.), DPEPhos (8.1 mg, 0.015 mmol, 0.050 equiv.) and $(CH_2Cl)_2$ (0.24 mL), with stirring for 18 h. Purification of the crude residue (>20:1 l:b) by column chromatography (SiO_2, 1:1 to 4:1 EtOAc:Pentane) yielded single isomer (*E*)-2-(4,4-dimethylpent-2-enoyl)pyridine 1-oxide **2d** (47.1 mg, 0.230 mmol, 77%) as a pale-brown solid.

1.00 mmol scale reaction: Prepared according to general procedure **E**, using 2-formylpyridine 1-oxide **1a** (123 mg, 1.00 mmol, 1.00 equiv.), 3,3-dimethylbut-1-yne (246 μL, 2.00 mmol, 2.00 equiv.), $Rh(nbd)_2BF_4$ (18.7 mg, 0.05 mmol, 0.050 equiv.), DPEPhos (27 mg, 0.05 mmol, 0.050 equiv.) and $(CH_2Cl)_2$ (0.8 mL), with stirring for 18 h. Purification of the crude residue (>20:1 l:b) by column chromatography (SiO_2, 1:1 EtOAc:Petrol to neat EtOAc) yielded single isomer (*E*)-2-(4,4-dimethylpent-2-enoyl)pyridine 1-oxide **2d** (170 mg, 0.828 mmol, 83%) as a pale-brown solid.

m.p 85-88 °C (CH_2Cl_2), {*Lit:* 85-88 °C}[12]; **δH** (400 MHz, $CDCl_3$) 8.25-8.18 (1H, m, *Ar*), 7.61-7.54 (1H, m, *Ar*), 7.41-7.29 (2H, m, *Ar*), 7.07 (1H, d, J = 15.9 Hz, C(6)*H*), 6.93 (1H, d, J = 15.8 Hz, C(5)*H*), 1.13 (9H, s, C(7)H_3); **δC** (101 MHz, $CDCl_3$) 187.5, 159.6, 147.4, 140.4, 127.5,

126.9, 125.6, 123.5, 34.3, 28.7; $\mathbf{\nu_{max}}$/cm^{-1} ($CHCl_3$) 2961, 1667, 1615, 1427, 1317, 1029, 863, 772, 654; **m/z HRMS** (ESI^+) $[M + H]^+$ $C_{12}H_{16}O_2N^+$ calc. 206.1176, found 206.1778. *Data is consistent with literature*[12]

(*E*)-2-(3-(Trimethylsilyl)acryloyl)pyridine 1-oxide (2e)

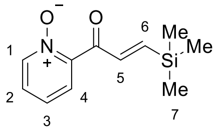

2e

Prepared according to general procedure **E**, using 2-formylpyridine 1-oxide **1a** (37 mg, 0.300 mmol, 1.00 equiv.), ethynyltrimethylsilane (83 µL, 0.600 mmol, 2.00 equiv.), $Rh(nbd)_2BF_4$ (5.6 mg, 0.015 mmol, 0.050 equiv.), DPEPhos (8.1 mg, 0.015 mmol, 0.050 equiv.) and $(CH_2Cl)_2$ (0.24 mL), with stirring for 18 h. Purification of the crude residue (>20:1 l:b) by column chromatography (SiO_2, 7:3 EtOAc:Pentane) yielded single isomer (*E*)-2-(3-(trimethylsilyl)acryloyl)pyridine 1-oxide **2e** (49 mg, 0.222 mmol, 74%) as an off-white solid.

m.p 53-56 °C (CH_2Cl_2); $\mathbf{\delta_H}$ (400 MHz, $CDCl_3$) 8.26-8.20 (1H, m, *Ar*), 7.60-7.53 (1H, m, *Ar*), 7.43-7.33 (2H, m, *Ar*), 7.31 (2H, app. s, C(5 and 6)*H*), 0.17 (9H, s, C(7)*H₃*); $\mathbf{\delta_C}$ (101 MHz, $CDCl_3$) 186.5, 149.0, 147.1, 140.3, 139.4, 127.7, 127.0, 125.6, -1.74; $\mathbf{\nu_{max}}$/cm^{-1} ($CHCl_3$) 2957, 1669, 1427, 1295, 1248, 1020, 987, 875, 844, 756; **m/z HRMS** (ESI^+) $[M + H]^+$ $C_{11}H_{16}O_2NSi^+$ calc. 222.0946, found 222.0945.

(*E*)-2-(3-(Cyclohex-1-en-1-yl)acryloyl)pyridine 1-oxide (2f)

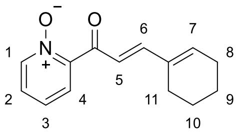

2f

Prepared according to general procedure **E**, using 2-formylpyridine 1-oxide **1a** (37 mg, 0.300 mmol, 1.00 equiv.), 1-ethynylcyclohex-1-ene (71 μL, 0.600 mmol, 2.00 equiv.), $Rh(nbd)_2BF_4$ (5.6 mg, 0.015 mmol, 0.050 equiv.), DPEPhos (8.1 mg, 0.015 mmol, 0.050 equiv.) and $(CH_2Cl)_2$ (0.24 mL), with stirring for 18 h. Purification of the crude residue (>20:1 l:b) by column chromatography (SiO_2, 1:1 to 9:1 EtOAc:Pentane) yielded single isomer (*E*)-2-(3-(cyclohex-1-en-1-yl)acryloyl)pyridine 1-oxide **2f** (55.6 mg, 0.243 mmol, 81%) as a pale-brown solid.

m.p 68-70 °C (CH_2Cl_2); **δ_H** (400 MHz, $CDCl_3$) 8.18-8.13 (1H, m, *Ar*), 7.56-7.50 (1H, m, *Ar*), 7.35-7.27 (3H, m, *Ar* and C(5 or 6)*H*), 6.92 (1H, d, *J* = 15.6 Hz, C(5 or 6)*H*), 6.25 (1H, bs, C(7)*H*), 2.22-2.14 (4H, m, C(8 to 11)H_2), 1.69-1.52 (4H, m, C(8 to 11)H_2); **δ_C** (101 MHz, $CDCl_3$) 187.1, 148.5, 147.6, 142.0, 140.4, 135.9, 127.4, 126.9, 125.6, 121.5, 26.9, 24.3, 22.0, 22.0; **ν_{max}**/cm^{-1} ($CHCl_3$) 2929, 1657, 1577, 1426, 1322, 1244, 1190, 1031, 768; **m/z HRMS** (ESI^+) $[M + H]^+$ $C_{14}H_{16}O_2N^+$ calc. 230.1176, found 230.1178.

2-Cinnamoylpyridine 1-oxide (2g)

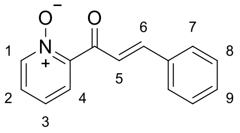

2g

Prepared according to general procedure **E**, using 2-formylpyridine 1-oxide **1a** (37 mg, 0.300 mmol, 1.00 equiv.), Phenylacetylene (66 µL, 0.600 mmol, 2.00 equiv.), $Rh(nbd)_2BF_4$ (5.6 mg, 0.015 mmol, 0.050 equiv.), DPEPhos (8.1 mg, 0.015 mmol, 0.050 equiv.) and $(CH_2Cl)_2$ (0.24 mL), with stirring for 18 h. Purification of the crude residue (9:1 l:b) by column chromatography (SiO_2, 1:1 EtOAc:Petrol to neat EtOAc) yielded single isomer 2-cinnamoylpyridine 1-oxide **2g** (34 mg, 0.160 mmol, 53%) as a pale-brown solid. A pure fraction of the branched isomer could not be isolated.

m.p 100-104 °C (CH_2Cl_2), {*Lit:* 104-107 °C}[12]; **δ_H** (400 MHz, $CDCl_3$) 8.26-8.22 (1H, m, *Ar*), 7.82 (1H, d, *J* = 15.9 Hz, C(5 or 6)*H*), 7.73 (1H, d, *J* = 15.9 Hz, C(5 or 6)*H*), 7.73-7.66 (1H, m, *Ar*), 7.67-7.61 (2H, m, *Ar*), 7.42-7.31 (5H, m, *Ar*); **δ_C** (101 MHz, $CDCl_3$) 186.5, 147.4, 144.4, 140.6, 134.8, 130.9, 129.0, 129.0, 127.8, 127.4, 125.8, 124.4; **ν_{max}**/cm^{-1} ($CHCl_3$) 2919, 1666, 1610, 1428, 1336, 765; **m/z HRMS** (ESI^+) $[M + Na]^+$ $C_{14}H_{11}O_2N^{23}Na^+$ calc. 248.0682, found 248.0682. *Data is consistent with literature*[12]

(*E*)-2-(3-(4-Methoxyphenyl)acryloyl)pyridine 1-oxide (2h)

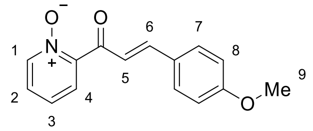

2h

Prepared according to general procedure **E**, using 2-formylpyridine 1-oxide **1a** (37 mg, 0.300 mmol, 1.00 equiv.), 1-ethynyl-4-methoxybenzene (79 mg, 0.600 mmol, 2.00 equiv.), $Rh(nbd)_2BF_4$ (5.6 mg, 0.015 mmol, 0.050 equiv.), DPEPhos (8.1 mg, 0.015 mmol, 0.050 equiv.) and $(CH_2Cl)_2$ (0.24 mL), with stirring for 18 h. Purification of the crude residue (>20:1 l:b) by column chromatography (SiO_2, 1:1 EtOAc:Petrol to neat EtOAc) yielded single isomer (*E*)-2-(3-(4-methoxyphenyl)acryloyl)pyridine 1-oxide **2h** (45.6 mg, 0.180 mmol, 60%) as a pale-brown solid.

m.p 95-98 °C (CH_2Cl_2), {*Lit:* 113-116 °C}[12]; **δ_H** (400 MHz, $CDCl_3$) 8.24-8.18 (1H, m, *Ar*), 7.74 (1H, d, *J* = 15.8 Hz, C(5 or 6)*H*), 7.63 (1H, dd, *J* = 7.4, 2.6 Hz, *Ar*), 7.61-7.54 (3H, m, C(5 or 6)*H* and *Ar*), 7.39-7.28 (2H, m, *Ar*), 6.92-6.84 (2H, m, *Ar*), 3.81 (3H, s, OC(9)H_3); **δ_C** (101 MHz, $CDCl_3$) 186.3, 162.1, 147.6, 144.6, 140.5, 130.8, 127.6, 127.4, 127.2, 125.7, 122.2, 114.5, 55.5; **ν_{max}**/cm^{-1} ($CHCl_3$) 2839, 1738, 1657, 1597, 1570, 1512, 1427, 1254, 1174, 1033; **m/z HRMS** (ESI^+) $[M + H]^+$ $C_{15}H_{14}O_3N^+$ calc. 256.0968, found 256.0970. *Data is consistent with literature*[12]

(*E*)-2-(3-(4-(Trifluoromethyl)phenyl)acryloyl)pyridine 1-oxide (2i)

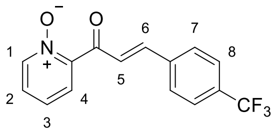

2i

Prepared according to general procedure **E**, using 2-formylpyridine 1-oxide **1a** (37 mg, 0.300 mmol, 1.00 equiv.), 1-ethynyl-4-(trifluoromethyl)benzene (98 μL, 0.600 mmol, 2.00 equiv.), $Rh(nbd)_2BF_4$ (5.6 mg, 0.015 mmol, 0.050 equiv.), DPEPhos (8.1 mg, 0.015 mmol, 0.050 equiv.) and $(CH_2Cl)_2$ (0.24 mL), with stirring for 40 h. Purification of the crude residue (>20:1 l:b) by column chromatography (SiO_2, 9:1 EtOAc:Petrol to neat EtOAc) yielded single isomer (*E*)-2-(3-(4-(trifluoromethyl)phenyl)acryloyl)pyridine 1-oxide **2i** (35.2 mg, 0.120 mmol, 40%) as a pale-brown solid.

m.p 112-116 °C (CH_2Cl_2); {*Lit:* 123-126 °C}[13]; **δ_F** (376 MHz, $CDCl_3$) -62.86 (C*F₃*); **δ_H** (400 MHz, $CDCl_3$) 8.27-8.21 (1H, m, *Ar*), 7.81 (2H, app. d, *J* = 1.5 Hz, C(5 and 6)*H*), 7.75-7.70 (3H, m, *Ar*), 7.63 (2H, d, *J* = 8.2 Hz, *Ar*), 7.46-7.34 (2H, m, *Ar*); **δ_C** (127 MHz, $CDCl_3$) 186.1, 147.0, 141.8, 140.6, 138.2, 132.1 (q, *J* = 32.6 Hz), 129.0, 128.2, 127.7, 126.6, 125.9, 125.9 (q, *J* = 3.8 Hz), 123.9 (q, *J* = 272.3 Hz); **ν_{max}**/cm^{-1} ($CHCl_3$) 1668, 1612, 1430, 1322, 1167, 1068, 830, 769; **m/z HRMS** (ESI^+) $[M + H]^+$ $C_{15}H_{11}O_2NF_3^+$ calc. 294.0736, found 294.0735. *Data is consistent with literature*[13]

(*E*)-2-(3-(4-(Methoxycarbonyl)phenyl)acryloyl)pyridine 1-oxide (2j)

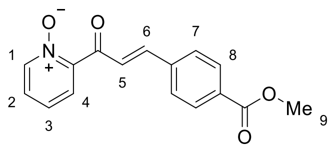

2j

Prepared according to general procedure **E**, using 2-formylpyridine 1-oxide **1a** (37 mg, 0.300 mmol, 1.00 equiv.), methyl 4-ethynylbenzoate (96.1 mg, 0.600 mmol, 2.00 equiv.), $Rh(nbd)_2BF_4$ (5.6 mg, 0.015 mmol, 0.050 equiv.), DPEPhos (8.1 mg, 0.015 mmol, 0.050 equiv.) and $(CH_2Cl)_2$ (0.24 mL), with stirring for 40 h. Purification of the crude residue (>20:1 l:b) by column chromatography (SiO_2, 9:1 EtOAc:Petrol to neat EtOAc) yielded single isomer (*E*)-2-(3-(4-(methoxycarbonyl)phenyl)acryloyl)pyridine 1-oxide **2j** (32.5 mg, 0.115 mmol, 38%) as a pale-brown solid.

m.p 148-150 °C (CH_2Cl_2); **δ_H** (400 MHz, $CDCl_3$) 8.28-8.22 (1H, m, *Ar*), 8.04 (2H, d, *J* = 8.5 Hz, *Ar*), 7.81 (2H, app. s, C(5 and 6)*H*), 7.77-7.65 (3H, m, *Ar*), 7.46-7.33 (2H, m, *Ar*), 3.93 (3H, s, C(9)H_3); **δ_C** 186.1, 166.6, 147.1, 142.5, 140.7, 139.0, 131.8, 130.2, 128.8, 128.2, 127.7, 126.4, 126.1, 52.4; **ν_{max}**/cm^{-1} ($CHCl_3$) 1719, 1664, 1614, 1432, 1284, 1112, 764, 743; **m/z HRMS** (ESI^+) $[M + H]^+$ $C_{16}H_{14}O_4N^+$ calc. 284.0917, found 284.0918. *Data is consistent with literature*[14]

(*E*)-2-(7-(1,3-Dioxoisoindolin-2-yl)hept-2-enoyl)pyridine 1-oxide (2k) and 2-(6-(1,3-Dioxoisoindolin-2-yl)-2-methylenehexanoyl)pyridine 1-oxide (3k)

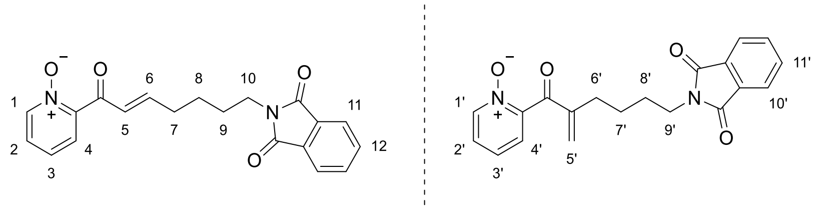

2k **3k**

Prepared according to general procedure **E**, using 2-formylpyridine 1-oxide **1a** (37 mg, 0.300 mmol, 1.00 equiv.), 2-(hex-5-yn-1-yl)isoindoline-1,3-dione (136 mg, 0.600 mmol, 2.00 equiv.), $Rh(nbd)_2BF_4$ (5.6 mg, 0.015 mmol, 0.050 equiv.), DPEPhos (8.1 mg, 0.015 mmol, 0.050 equiv.) and $(CH_2Cl)_2$ (0.24 mL), with stirring for 6 h. Purification of the crude residue (3.3:1 l:b) by column chromatography (SiO_2, 99:1 CH_2Cl_2:MeOH to 97:3 CH_2Cl_2:MeOH) yielded (*E*)-2-(7-(1,3-dioxoisoindolin-2-yl)hept-2-enoyl)pyridine 1-oxide **2k** (45.9 mg, 0.131 mmol, 43%) and 2-(6-(1,3-dioxoisoindolin-2-yl)-2-methylenehexanoyl)pyridine 1-oxide **3k** (19.4 mg, 0.055 mmol, 18%) both as yellow viscous oils.

(E)-2-(7-(1,3-Dioxoisoindolin-2-yl)hept-2-enoyl)pyridine 1-oxide (2k)**:** **δ_H** (400 MHz, $CDCl_3$) 8.16 (1H, dd, *J* = 6.2, 1.4 Hz, *Ar*), 7.80 (2H, dd, *J* = 5.4, 3.1 Hz, *Ar*), 7.68 (2H, dd, *J* = 5.4, 3.0 Hz, *Ar*), 7.52 (1H, dd, *J* = 7.5, 2.5 Hz, *Ar*), 7.37-7.27 (2H, m, *Ar*), 7.03-6.95 (1H, m, C(6)*H*), 6.93 (1H, d, *J* = 15.7 Hz, C(5)*H*), 3.67 (2H, app. t, *J* = 7.1 Hz, C(10)H_2), 2.38-2.27 (2H, m, C(7)H_2), 1.70 (2H, app. p, *J* = 7.7 Hz, C(9)H_2), 1.53 (2H, app. p, *J* = 7.6 Hz, C(8)H_2); **δ_C** (101 MHz, $CDCl_3$) 186.8, 168.5, 149.2, 147.2, 140.4, 134.0, 132.2, 128.5, 127.6, 126.9, 125.7, 123.3, 37.6, 32.2, 28.3, 25.3; **ν_{max}**/cm^{-1} ($CHCl_3$) 2939, 1706, 1619, 1429, 1397, 721; **m/z HRMS** (ESI^+) $[M + H]^+$ $C_{20}H_{19}O_4N_2^+$ calc. 351.1339, found 351.1340.

2-(6-(1,3-Dioxoisoindolin-2-yl)-2-methylenehexanoyl)pyridine 1-oxide (3k)**:** **δ_H** (400 MHz, $CDCl_3$) 8.28-8.22 (1H, m, *Ar'*), 7.83 (2H, dd, *J* = 5.5, 3.0 Hz, *Ar'*), 7.70 (2H, dd, *J* = 5.5, 3.0

Hz, *Ar'*), 7.44-7.33 (2H, m, *Ar'*), 7.37-7.27 (1H, m, *Ar'*), 5.97 (1H, s, C(5')*H*H), 5.67 (1H, s, C(5')H*H*), 3.72 (2H, t, *J* = 7.2 Hz, C(9')*H*$_2$), 2.56-2.47 (2H, m, C(6')*H*$_2$), 1.83-1.71 (2H, m, C(8')*H*$_2$), 1.68-1.54 (2H, m, C(7')*H*$_2$); **δC** (151 MHz, $CDCl_3$) 190.8, 168.6, 147.3, 147.2, 140.2, 134.0, 132.3, 128.8, 126.9, 126.7, 125.3, 123.3, 37.8, 29.5, 28.2, 25.3; **νmax**/cm^{-1} ($CHCl_3$) 2940, 1704, 1672, 1427, 1397, 771, 721; **m/z HRMS** (ESI$^+$) $[M + H]^+$ $C_{20}H_{19}O_4N_2^+$ calc. 351.1339, found 351.1339.

(*E*)-2-(3-(1-((*Tert*-butoxycarbonyl)amino)cyclohexyl)acryloyl)pyridine 1-oxide (2l)

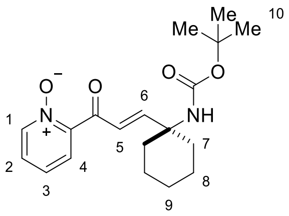

2l

Prepared according to general procedure **E**, using 2-formylpyridine 1-oxide **1a** (37 mg, 0.300 mmol, 1.00 equiv.), *tert*-butyl (1-ethynylcyclohexyl)carbamate (223 mg, 0.600 mmol, 2.00 equiv.), $Rh(nbd)_2BF_4$ (5.6 mg, 0.015 mmol, 0.050 equiv.), DPEPhos (8.1 mg, 0.015 mmol, 0.050 equiv.) and $(CH_2Cl)_2$ (0.24 mL), with stirring for 18 h. Purification of the crude residue (>20:1 l:b) by column chromatography (SiO_2, 7:3 EtOAc:Pentane) yielded single isomer (*E*)-2-(3-(1-((*Tert*-butoxycarbonyl)amino)cyclohexyl)acryloyl)pyridine 1-oxide **2l** (75 mg, 0.216 mmol, 72%) as an off-white solid.

m.p (CH_2Cl_2) 50-55 °C; **δH** (400 MHz, $CDCl_3$) 8.20-8.11 (1H, m, *Ar*), 7.60-7.51 (1H, m, *Ar*), 7.37-7.26 (2H, m, *Ar*), 7.06 (1H, d, *J* = 15.7 Hz, C(5 or 6)*H*), 7.02 (1H, d, *J* = 15.7 Hz, C(5 or 6)*H*), 4.63 (1H, bs, N*H*), 2.07-1.94 (2H, m, C(7 to 9)*H*$_2$), 1.65-1.42 (7H, m, C(7 to 9)*H*$_2$), 1.39 (9H, s, C(10)*H*$_3$), 1.33-1.21 (1H, m, C(7 to 9)*H*); **δC** (101 MHz, $CDCl_3$) 186.9, 153.9 (2C), 147.2, 140.3, 127.5, 127.1, 125.4, 125.1, 79.4, 55.5, 34.6, 28.4, 25.2, 21.3; **νmax**/cm

(Chloroform) 3247, 2978, 2931, 2859, 1699, 1619, 1520, 1428, 1356, 1317, 1249, 1167, 759; **m/z HRMS** (ESI^+) $[M + H]^+$ $C_{19}H_{26}O_4N_2{}^{23}Na^+$ calc. 369.1785; found 369.1783.

(*E*)-2-(2-methyl-3-phenylacryloyl)pyridine 1-oxide (2m)

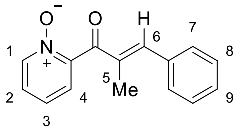

2m

Prepared according to general procedure **E**, using 2-formylpyridine 1-oxide **1a** (37 mg, 0.300 mmol, 1.00 equiv.), 1-phenyl-1-propyne (75 µL, 0.600 mmol, 2.00 equiv.), $Rh(nbd)_2BF_4$ (5.6 mg, 0.015 mmol, 0.050 equiv.), DPEPhos (8.1 mg, 0.015 mmol, 0.050 equiv.) and $(CH_2Cl)_2$ (0.24 mL), ***with stirring for 18 h at 80 °C***. Purification of the crude residue (15:1 rr) by column chromatography (SiO_2, neat EtOAc to 19:1 EtOAc:MeOH) yielded single isomer (*E*)-2-(2-methyl-3-phenylacryloyl)pyridine 1-oxide **2m** (27.7 mg, 0.116 mmol, 39%) as an off-white solid.

m.p (CH_2Cl_2) 106-108 °C; $\mathbf{\delta_H}$ (400 MHz, Acetone-d6) 8.24-8.18 (1H, m, *Ar*), 7.56-7.35 (8H, m, *Ar*), 7.23 (1H, s, C(6)*H*), 2.19 (3H, d, J = 1.4 Hz, C(5)H_3); $\mathbf{\delta_C}$ (101 MHz, Acetone-d6) 192.9, 148.2, 143.1, 140.6, 136.8, 136.4, 130.9, 130.0, 129.4, 127.8, 126.1, 125.5, 12.8; $\mathbf{\nu_{max}}$/cm (Chloroform) 3110, 3054, 2963, 1657, 1620, 1448, 1303, 1241, 1157, 1019, 927, 845, 770, 695; **m/z HRMS** (ESI^+) $[M + H]^+$ $C_{15}H_{14}O_2N^+$ calc. 240.1019, found 240.1019.

A NOESY Spectrum of product **2m** is shown below:

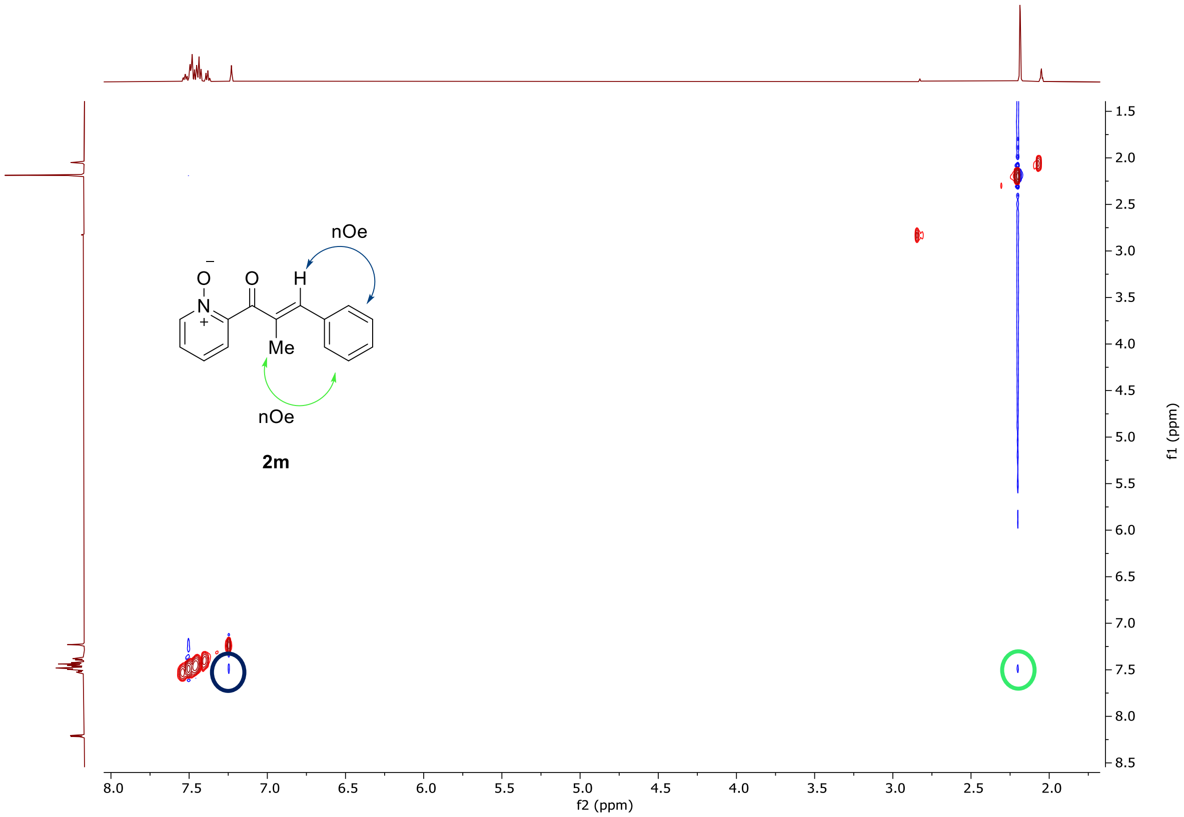

4.3 Aldehyde Scope

(*E*)-5-Bromo-2-(3-cyclohexylacryloyl)pyridine-1-oxide (2n)

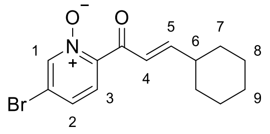

2n

Prepared according to general procedure **E**, using 5-bromo-2-formylpyridine 1-oxide **1b** (60 mg, 0.300 mmol, 1.00 equiv.), ethynylcyclohexane (78 μL, 0.600 mmol, 2.00 equiv.), $Rh(nbd)_2BF_4$ (5.6 mg, 0.015 mmol, 0.050 equiv.), DPEPhos (8.1 mg, 0.015 mmol, 0.050 equiv.) and $(CH_2Cl)_2$ (0.24 mL), with stirring for 18 h. Purification of the crude residue (>20:1 l:b) by column chromatography (SiO_2, 1:9 to 15:85 EtOAc:Petrol) yielded single isomer (*E*)-5-bromo-2-(3-cyclohexylacryloyl)pyridine-1-oxide **2n** (69.6 mg, 0.225 mmol, 75%) as an off-white solid.

m.p 70-74 °C (CH_2Cl_2); **δ_H** (400 MHz, $CDCl_3$) 8.34 (1H, dd, *J* = 1.5, 0.6 Hz, *Ar*), 7.48 (1H, dd, *J* = 8.6, 0.6 Hz, *Ar*), 7.44 (1H, dd, *J* = 8.5, 1.6 Hz, *Ar*), 7.04 (1H, dd, *J* = 15.7, 6.4 Hz, C(5)*H*), 6.94 (1H, dd, *J* = 15.7, 1.0 Hz, C(4)*H*), 2.30-2.17 (1H, m, C(6)*H*), 1.86-1.72 (4H, m, C(7 to 9)H_2), 1.72-1.61 (1H, m, C(7 to 9)H_2), 1.38-1.11 (5H, m, C(7 to 9)H_2); **δ_C** (101 MHz, $CDCl_3$) 186.2, 155.6, 146.3, 141.8, 128.7, 127.3, 125.5, 122.7, 41.0, 31.7, 26.0, 25.8; **ν_{max}**/cm^{-1} ($CHCl_3$) 3106, 2932, 2855, 1717, 1699, 1376, 910; **m/z HRMS** (ESI^+) $[M + H]^+$ $C_{14}H_{17}O_2N^{79}Br^+$ calc. 310.0437, found 310.0437, $C_{14}H_{17}O_2N^{81}Br^+$ calc. 312.0417, found 312.0416.

(*E*)-2-(3-Cyclohexylacryloyl)-5-methoxypyridine 1-oxide (2o)

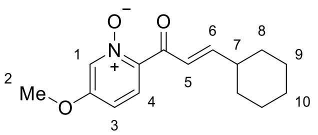

2o

Prepared according to general procedure **E**, using 2-formyl-5-methoxypyridine 1-oxide **1c** (46 mg, 0.300 mmol, 1.00 equiv.), ethynylcyclohexane (78 μL, 0.600 mmol, 2.00 equiv.), $Rh(nbd)_2BF_4$ (5.6 mg, 0.015 mmol, 0.050 equiv.), DPEPhos (8.1 mg, 0.015 mmol, 0.050 equiv.) and $(CH_2Cl)_2$ (0.24 mL), with stirring for 18 h. Purification of the crude residue (>20:1 l:b) by column chromatography (SiO_2, 1:1 to 7:3 EtOAc:Petrol) yielded single isomer (*E*)-2-(3-cyclohexylacryloyl)-5-methoxypyridine 1-oxide **2o** (51.7 mg, 0.198 mmol, 66%) as a yellow/orange powdered solid.

m.p 80-89 °C (CH_2Cl_2); δ_H (400 MHz, $CDCl_3$) 7.91 (1H, d, *J* = 2.3 Hz, *Ar*), 7.60 (1H, d, *J* = 9.0 Hz, *Ar*), 7.11 (1H, dd, *J* = 15.7, 1.0 Hz, C(5)*H*), 7.02 (1H, dd, *J* = 15.7, 6.4 Hz, C(6)*H*), 6.90 (1H, dd, *J* = 9.0, 2.3 Hz, *Ar*), 3.88 (3H, s, C(2)H_3), 2.29-2.16 (1H, m, C(7)*H*), 1.87-1.78 (2H, m, C(8 to 10)H_2), 1.75 (2H, app dt, *J* = 11.7, 3.7 Hz, C(8 to 10)H_2), 1.71-1.61 (1H, m, C(8 to 10)H_2), 1.37-1.12 (5H, m, C(8 to 10)H_2); δ_C (101 MHz, $CDCl_3$) 186.2, 159.6, 154.2, 141.3, 128.2, 127.8, 126.1, 113.2, 56.5, 40.9, 31.8, 26.1, 25.9; ν_{max}/cm^{-1} ($CHCl_3$) 2925, 2851, 1664, 1616, 1396, 1173, 1018, 971; **m/z HRMS** (ESI$^+$) [M + H]$^+$ $C_{15}H_{20}O_3N^+$ calc. 262.1438, found 262.1437.

(*E*)-2-(3-Cyclohexylacryloyl)-5-(methoxycarbonyl)pyridine 1-oxide (2p)

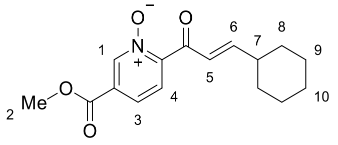

2p

Prepared according to general procedure **E**, using 2-formyl-5-(methoxycarbonyl)pyridine 1-oxide **1d** (54.3 mg, 0.300 mmol, 1.00 equiv.), ethynylcyclohexane (78 μL, 0.600 mmol, 2.00 equiv.), $Rh(nbd)_2BF_4$ (5.6 mg, 0.015 mmol, 0.050 equiv.), DPEPhos (8.1 mg, 0.015 mmol, 0.050 equiv.) and $(CH_2Cl)_2$ (0.24 mL), with stirring for 18 h. Purification of the crude residue (>20:1 l:b) by column chromatography (SiO_2, 1:4 to 1:1 EtOAc:Petrol) yielded single isomer (*E*)-2-(3-cyclohexylacryloyl)-5-(methoxycarbonyl)pyridine 1-oxide **2p** (51 mg, 0.176 mmol, 59%) as an off-white solid.

m.p 66-70 °C (CH_2Cl_2); **δ_H** (400 MHz, $CDCl_3$) 8.75 (1H, d, *J* = 1.4 Hz, *Ar*), 7.86 (1H, dd, *J* = 8.2, 1.2 Hz, *Ar*), 7.59 (1H, d, *J* = 8.2 Hz, *Ar*), 7.01 (1H, dd, *J* = 15.8, 6.6 Hz, C(6)*H*), 6.87 (1H, dd, *J* = 15.7, 1.2 Hz, C(5)*H*), 3.98 (3H, s, C(2)H_3), 2.29-2.18 (1H, m, C(7)*H*), 1.86-1.71 (4H, m, C(8 to 10)H_2), 1.71-1.63 (1H, m, C(8 to 10)H_2), 1.37-1.11 (5H, m, C(8 to 10)H_2); **δ_C** (101 MHz, $CDCl_3$) 186.7, 163.1, 156.1, 150.0, 141.6, 130.8, 126.7, 125.9, 125.5, 53.4, 41.0, 31.7, 26.0, 25.8; **ν_{max}**/cm^{-1} ($CHCl_3$) 2926, 2852, 1733, 1618, 1438, 1387, 1301, 1233, 1113; **m/z HRMS** (ESI^+) $[M + H]^+$ $C_{16}H_{20}O_4N^+$ calc. 290.1387, found 290.1387.

(*E*)-2-Chloro-6-(3-cyclohexylacryloyl)pyridine 1-oxide (2q)

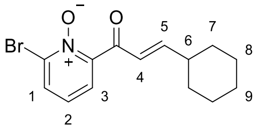

2q

Prepared according to general procedure **E**, using 2-bromo-6-formylpyridine-1-oxide **1e** (60 mg, 0.300 mmol, 1.00 equiv.), ethynylcyclohexane (78 μL, 0.600 mmol, 2.00 equiv.), $Rh(nbd)_2BF_4$ (5.6 mg, 0.015 mmol, 0.050 equiv.), DPEPhos (8.1 mg, 0.015 mmol, 0.050 equiv.) and $(CH_2Cl)_2$ (0.24 mL), with stirring for 18 h. Purification of the crude residue (>20:1 l:b) by column chromatography (SiO_2, 1:9 to 1:4 EtOAc:Petrol) yielded single isomer (*E*)-2-chloro-6-(3-cyclohexylacryloyl)pyridine 1-oxide **2q** (53.8 mg, 0.174 mmol, 58%) as a white solid.

m.p 101-102 °C (CH_2Cl_2); **δ_H** (400 MHz, Acetone-d6) 7.98 (1H, dd, *J* = 8.1, 2.0 Hz, *Ar*), 7.54 (1H, dd, *J* = 7.8, 2.0 Hz, *Ar*), 7.34 (1H, t, *J* = 7.9 Hz, *Ar*), 6.87 (1H, dd, *J* = 15.8, 6.7 Hz, C(5)*H*), 6.70 (1H, dd, *J* = 15.8, 1.3 Hz, C(4)*H*), 2.31-2.17 (1H, m, C(7)*H*), 1.83-1.70 (4H, m, C(7 to 9)H_2), 1.71-1.61 (1H, m, C(7 to 9)H_2), 1.41-1.27 (2H, m, C(7 to 9)H_2), 1.27-1.12 (3H, m, C(7 to 9)H_2); **δ_C** (101 MHz, Acetone-d6) 187.6, 154.6, 149.2, 133.9, 133.2, 126.7, 126.0, 126.0, 41.4, 32.3, 26.6, 26.3; **ν_{max}**/cm^{-1} ($CHCl_3$) 2923, 2850, 1668, 1615, 1374; **m/z HRMS** (ESI$^+$) $[M + H]^+$ $C_{14}H_{17}O_2N^{79}Br^+$ calc. 310.0437, found 310.0438.

(*E*)-2-Chloro-6-(3-cyclohexylacryloyl)pyridine 1-oxide (2r)

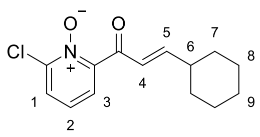

2r

Prepared according to general procedure **E**, using 2-chloro-6-formylpyridine-1-oxide **1f** (47 mg, 0.300 mmol, 1.00 equiv.), ethynylcyclohexane (78 µL, 0.600 mmol, 2.00 equiv.), $Rh(nbd)_2BF_4$ (5.6 mg, 0.015 mmol, 0.050 equiv.), DPEPhos (8.1 mg, 0.015 mmol, 0.050 equiv.) and $(CH_2Cl)_2$ (0.24 mL), with stirring for 18 h. Purification of the crude residue (>20:1 l:b) by column chromatography (SiO_2, 1:9 to 1:4 EtOAc:Petrol) yielded single isomer (*E*)-2-chloro-6-(3-cyclohexylacryloyl)pyridine 1-oxide **2r** (45.3 mg, 0.171 mmol, 57%) as an off-white solid.

m.p 84-86 °C (CH_2Cl_2); **δ_H** (400 MHz, $CDCl_3$) 7.59 (1H, dd, *J* = 8.1, 2.1 Hz, *Ar*), 7.49 (1H, dd, *J* = 7.9, 2.1 Hz, *Ar*), 7.24 (1H, app t, *J* = 7.9 Hz, *Ar*), 7.02 (1H, dd, *J* = 15.7, 6.6 Hz, C(5)*H*), 6.90 (1H, dd, *J* = 15.7, 1.2 Hz, C(4)*H*), 2.29-2.16 (1H, m, C(6)*H*), 1.85-1.71 (3H, m, C(7 to 9)H_2), 1.71-1.62 (1H, m, C(7 to 9)H_2), 1.36-1.11 (6H, m, C(7 to 9)H_2); **δ_C** (101 MHz, $CDCl_3$) 186.4, 155.4, 148.8, 143.0, 128.5, 125.5, 125.3, 125.0, 41.0, 31.7, 26.0, 25.8; **ν_{max}**/cm^{-1} ($CHCl_3$) 2924, 2851, 1670, 1616, 1380; **m/z HRMS** (ESI^+) $[M + H]^+$ $C_{14}H_{17}O_2N^{35}Cl^+$ calc. 266.0942, found 266.0941.

(*E*)-2-(3-Cyclohexylacryloyl)-4-methylpyridine 1-oxide (2s)

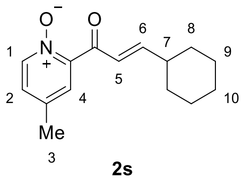

2s

Prepared according to general procedure **E**, using 2-formyl-4-methylpyridine 1-oxide **1g** (41.1 mg, 0.300 mmol, 1.00 equiv.), ethynylcyclohexane (78 μL, 0.600 mmol, 2.00 equiv.), $Rh(nbd)_2BF_4$ (5.6 mg, 0.015 mmol, 0.050 equiv.), DPEPhos (8.1 mg, 0.015 mmol, 0.050 equiv.) and $(CH_2Cl)_2$ (0.24 mL), with stirring for 18 h. Purification of the crude residue (>20:1 l:b) by column chromatography (SiO_2, 1:1 EtOAc:Petrol to 3:97 MeOH:EtOAc) yielded single isomer (*E*)-2-(3-cyclohexylacryloyl)-4-methylpyridine 1-oxide **2s** (42.6 mg, 0.174 mmol, 58%) as a pale-brown viscous oil.

δ_H (400 MHz, $CDCl_3$) 8.05 (1H, d, *J* = 6.6 Hz, *Ar*), 7.31 (1H, d, *J* = 2.6 Hz, *Ar*), 7.12 (1H, dd, *J* = 6.7, 2.7 Hz, *Ar*), 6.97 (1H, dd, *J* = 15.8, 5.6 Hz, C(6)*H*), 6.91 (1H, d, *J* = 15.8 Hz, C(5)*H*), 2.33 (3H, s, C(3)H_3), 2.25-2.14 (1H, m, C(7)*H*), 1.83-1.67 (4H, m, C(8 to 10)H_2), 1.68-1.58 (1H, m, C(8 to 10)H_2), 1.33-1.08 (5H, m, C(8 to 10)H_2); **δ_C** (101 MHz, $CDCl_3$) 187.5, 154.9, 146.6, 139.7, 137.4, 128.3, 127.2, 125.9, 40.9, 31.7, 26.0, 25.8, 20.4; **ν_{max}**/cm^{-1} ($CHCl_3$) 2924, 2851, 1668, 1624, 1608, 1449, 1421, 1241, 1200, 848, 826; **m/z HRMS** (ESI^+) $[M + H]^+$ $C_{15}H_{20}O_2N^+$ calc. 246.1489, found 246.1488.

(*E*)-2-(3-Cyclohexylacryloyl)-3-methylpyridine 1-oxide (2t)

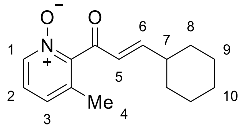

2t

Prepared according to general procedure **E**, using 2-formyl-3-methylpyridine 1-oxide **1g** (41.1 mg, 0.300 mmol, 1.00 equiv.), ethynylcyclohexane (78 μL, 0.600 mmol, 2.00 equiv.), $Rh(nbd)_2BF_4$ (5.6 mg, 0.015 mmol, 0.050 equiv.), DPEPhos (8.1 mg, 0.015 mmol, 0.050 equiv.) and $(CH_2Cl)_2$ (0.24 mL), with stirring for 18 h. Purification of the crude residue (14:1 l:b) by column chromatography (SiO_2, 1:1 EtOAc:Petrol to 19:1 EtOAc:MeOH) yielded single isomer (*E*)-2-(3-cyclohexylacryloyl)-3-methylpyridine 1-oxide **2t** (46.3 mg, 0.189 mmol, 63%) as an off-white solid.

m.p 99-102 °C (CH_2Cl_2); **δ_H** (400 MHz, $CDCl_3$) 8.09 (1H, d, *J* = 6.2 Hz, *Ar*), 7.24 -7.14 (2H, m, *Ar*), 6.71 (1H, dd, *J* = 16.1, 6.4 Hz, C(6)*H*), 6.38 (1H, dd, *J* = 16.0, 1.4 Hz, C(5)*H*), 2.27-2.16 (1H, m, C(7)*H*), 1.84-1.69 (4H, m, C(8 to 10)H_2), 1.70-1.61 (1H, m, C(8 to 10)H_2), 1.35-1.20 (2H, m, C(8 to 10)H_2), 1.21-1.08 (3H, m, C(8 to 10)H_2); **δ_C** (101 MHz, $CDCl_3$) 189.6, 156.6, 146.5, 137.3, 134.8, 128.3, 127.1, 125.4, 40.8, 31.5, 26.0, 25.8, 17.6; **ν_{max}**/cm^{-1} ($CHCl_3$) 2925, 2851, 1664, 1619, 1448, 1419, 1289, 1241, 966, 789; **m/z HRMS** (ESI^+) $[M + H]^+$ $C_{15}H_{20}O_2N^+$ calc. 246.1489, found 246.1486.

(*E*)-3-Bromo-2-(3-cyclohexylacryloyl)pyridine 1-oxide (2u)

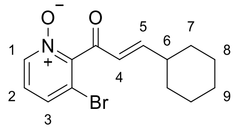

2u

Prepared according to general procedure **E**, using 3-bromo-2-formylpyridine 1-oxide **1i** (60 mg, 0.300 mmol, 1.00 equiv.), ethynylcyclohexane (78 μL, 0.600 mmol, 2.00 equiv.), $Rh(nbd)_2BF_4$ (5.6 mg, 0.015 mmol, 0.050 equiv.), DPEPhos (8.1 mg, 0.015 mmol, 0.050 equiv.) and $(CH_2Cl)_2$ (0.24 mL), with stirring for 18 h. Purification of the crude residue (>20:1 l:b) by column chromatography (SiO_2, 1:1 EtOAc:Petrol to neat EtOAc) yielded single isomer (*E*)-3-bromo-2-(3-cyclohexylacryloyl)pyridine 1-oxide **2u** (84.6 mg, 0.274 mmol, 91%) as an off-white solid.

m.p 80-82 °C (CH_2Cl_2); $\boldsymbol{\delta_H}$ (400 MHz, $CDCl_3$) 8.15 (1H, dd, *J* = 6.5, 0.9 Hz, *Ar*), 7.49 (1H, dd, *J* = 8.3, 0.9 Hz, *Ar*), 7.18 (1H, dd, *J* = 8.3, 6.5 Hz, *Ar*), 6.69 (1H, dd, *J* = 16.2, 6.4 Hz, C(5)*H*), 6.31 (1H, dd, *J* = 16.2, 1.5 Hz, C(4)*H*), 2.29-2.15 (1H, m, C(6)*H*), 1.85-1.73 (4H, m, C(7 to 9)H_2), 1.70-1.59 (1H, m, C(7 to 9)H_2), 1.35-1.22 (2H, m, C(7 to 9)H_2), 1.22-1.08 (3H, m, C(7 to 9)H_2); $\boldsymbol{\delta_C}$ (101 MHz, $CDCl_3$) 187.5, 158.2, 146.9, 138.8, 129.6, 126.2, 125.9, 118.1, 40.9, 31.4, 25.9, 25.7; $\boldsymbol{\nu_{max}}$/cm^{-1} ($CHCl_3$) 2924, 2851, 1692, 1411, 1283, 1249, 907; **m/z HRMS** (ESI$^+$) $[M + H]^+$ $C_{14}H_{17}O_2N^{79}Br^+$ calc. 310.0437, found 310.0438.

2,6-Bis((*E*)-3-cyclohexylacryloyl)pyridine 1-oxide (2v), 2,6-bis(2-cyclohexylacryloyl)pyridine 1-oxide (3v)

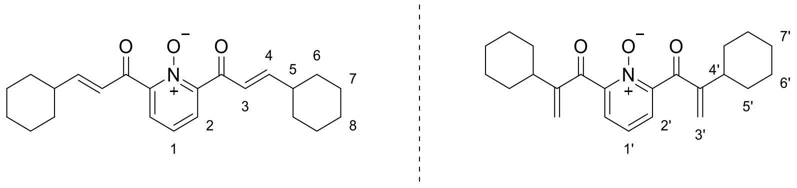

Prepared according to general procedure **E**, using 2,6-diformylpyridine 1-oxide **1j** (30 mg, 0.200 mmol, 1.00 equiv.), ethynylcyclohexane (105 μL, 0.800 mmol, 4.00 equiv.), $Rh(nbd)_2BF_4$ (3.7 mg, 0.010 mmol, 0.050 equiv.), DPEPhos (5.4 mg, 0.010 mmol, 0.050 equiv.) and $(CH_2Cl)_2$ (0.16 mL), with stirring for 18 h. Purification of the crude residue (>20:1 l:b) by column chromatography (SiO_2, 1:19 to 1:4 EtOAc:Petrol) yielded a >20:1 total inseparable mixture of regioisomers **2v/3v** (31.6 mg, 0.086 mmol, 43%) as an off-white solid.

m.p 135-136 °C (CH_2Cl_2); δ_H (400 MHz, $CDCl_3$) 7.59 (2H, d, *J* = 7.8 Hz, *Ar*), 7.35 (1H, dd, *J* = 8.1, 7.5 Hz, *Ar*), 6.99 (2H, dd, *J* = 15.7, 6.7 Hz, C(4)*H*), 6.83 (2H, dd, *J* = 15.7, 1.3 Hz, C(3)*H*), 5.89 (2H, d, *J* = 1.3 Hz, C(3')*H*H), 5.63 (2H, app s, C(3')H*H*), 2.29-2.15 (2H, m, C(5)*H*), 1.85-1.70 (8H, m, C(6 to 8)H_2), 1.70-1.56 (2H, m, C(6 to 8)H_2), 1.36-1.09 (10H, m, C(6 to 8)H_2); δ_C (101 MHz, $CDCl_3$) 187.1, 155.5, 148.1, 128.2, 125.7, 125.5, 41.0, 32.5*, 31.7, 26.7*, 26.0, 25.8 (* denotes the minor branched regioisomer **3v**); ν_{max}/cm^{-1} ($CHCl_3$) 2924, 2851, 1674, 1621, 1448, 1386, 978, 968, 754; **m/z HRMS** (ESI^+) $[M + H]^+$ $C_{23}H_{30}O_3N^+$ calc. 368.2220, found 368.2221.

(*E*)-1-(3-Cyclohexylacryloyl)isoquinoline 2-oxide (2w) and 1-(2-cyclohexylacryloyl)iso - quinoline 2-oxide (3w)

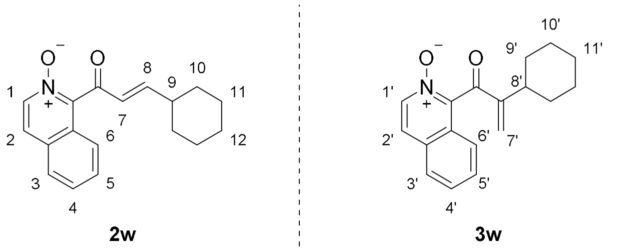

Prepared according to general procedure **E**, using 1-formylisoquinoline 2-oxide **1k** (52 mg, 0.300 mmol, 1.00 equiv.), ethynylcyclohexane (78 μL, 0.600 mmol, 2.00 equiv.), $Rh(nbd)_2BF_4$ (5.6 mg, 0.015 mmol, 0.050 equiv.), DPEPhos (8.1 mg, 0.015 mmol, 0.050 equiv.) and $(CH_2Cl)_2$ (0.24 mL), with stirring for 18 h. Purification of the crude residue (15:1 l:b) by column chromatography (SiO_2, 1:1 19:1 EtOAc:Petrol) yielded a 15:1 total inseparable mixture of regioisomers **2w/3w** (59.4 mg, 0.211 mmol, 70%) as a pale-brown solid.

m.p 82-86 °C (CH_2Cl_2); **δ_H** (400 MHz, $CDCl_3$) 8.07 (1H, d, *J* = 7.1 Hz, *Ar*), 7.84-7.75 (1H, m, *Ar*), 7.69 (1H, d, *J* = 7.1 Hz, *Ar*), 7.61-7.51 (3H, m, *Ar*), 6.74 (1H, dd, *J* = 16.0, 6.3 Hz, C(8)*H*), 6.52 (1H, dd, *J* = 16.0, 1.4 Hz, C(7)*H*), 5.89 (1H, d, *J* = 1.2 Hz, C(7')*H*H), 5.54 (1H, app s, C(7')H*H*), 2.89-2.80 (1H, m, C(8')*H*), 2.25-2.11 (1H, m, C(9)*H*), 1.99 (2H, d, *J* = 9.6 Hz, C(10' to 12')H_2), 1.81-1.65 (4H, m, C(10 to 12)H_2), 1.66-1.56 (1H, m, C(10 to 12)H_2), 1.49-1.35 (4H, m, C(10' to 12')H_2), 1.33-1.04 (5H, m, C(10 to 12)H_2); **δ_C** (101 MHz, $CDCl_3$) 191.6*, 189.3, 157.2, 153.5*, 142.9, 136.8, 130.1, 129.0, 128.8, 127.3, 127.2, 127.1*, 124.8, 124.6, 123.5, 123.2*, 40.8, 37.3*, 32.4*, 31.4, 26.6*, 26.3*, 25.9, 25.7 (* denotes the minor branched regioisomer **3w**); **ν_{max}**/cm^{-1} ($CHCl_3$) 2926, 2852, 1664, 1618, 1426, 1331, 1229, 1146, 979, 965, 819, 750; **m/z HRMS** (ESI^+) $[M + H]^+$ $C_{18}H_{20}O_2N^+$ calc. 282.1489, found 282.1484.

(*E*)-3-(3-Cyclohexylacryloyl)isoquinoline 2-oxide (2x)

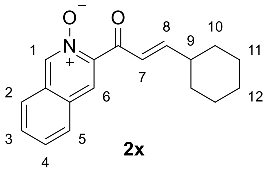

2x

Prepared according to general procedure **E**, using 3-formylisoquinoline 2-oxide **1l** (52 mg, 0.300 mmol, 1.00 equiv.), ethynylcyclohexane (78 µL, 0.600 mmol, 2.00 equiv.), $Rh(nbd)_2BF_4$ (5.6 mg, 0.015 mmol, 0.050 equiv.), DPEPhos (8.1 mg, 0.015 mmol, 0.050 equiv.) and $(CH_2Cl)_2$ (0.24 mL), with stirring for 18 h. Purification of the crude residue (>20:1 l:b) by column chromatography (SiO_2, 1:1 to 7:3 EtOAc:Petrol) yielded single isomer (*E*)-3-(3-cyclohexylacryloyl)isoquinoline 2-oxide **2x** (53.8 mg, 0.191 mmol, 64%) as an yellow viscous oil.

δ_H (600 MHz, Acetone-d6) 8.86 (1H, s, *Ar*), 8.04 (1H, s, *Ar*), 8.03 (1H, d, *J* = 8.3 Hz, *Ar*), 7.92 (1H, d, *J* = 1.1 Hz, *Ar*), 7.73 (1H, ddd, *J* = 8.3, 6.9, 1.2 Hz, *Ar*), 7.66 (1H, ddd, *J* = 8.2, 7.0, 1.2 Hz, *Ar*), 6.87 (1H, dd, *J* = 15.8, 6.4 Hz, C(8)*H*), 6.81 (1H, dd, *J* = 15.8, 1.0 Hz, C(7)*H*), 2.28-2.21 (1H, m, C(9)*H*), 1.82-1.78 (2H, m, C(10 to 12)H_2), 1.75 (2H, ddd, *J* = 12.5, 5.5, 2.4 Hz, C(10 to 12)H_2), 1.70-1.62 (1H, m, C(10 to 12)H_2), 1.38-1.28 (2H, m, C(10 to 12)H_2), 1.25-1.16 (3H, m, C(10 to 12)H_2); **δ_C** (151 MHz, Acetone-d6) 188.3, 153.7, 146.1, 137.1, 131.6, 131.2, 129.6, 128.9, 128.6, 127.6, 125.5, 125.4, 41.4, 32.4, 26.6, 26.4; **ν_{max}**/cm^{-1} ($CHCl_3$) 2925, 2851, 1669, 1625, 1438, 1317, 1210, 1179, 1125; **m/z HRMS** (ESI^+) $[M + H]^+$ $C_{18}H_{20}O_2N^+$ calc. 282.1489, found 282.1489.

(*E*)-2-(3-Cyclohexylacryloyl)pyrazine 1-oxide (2y)

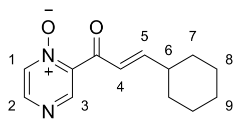

2y

Prepared according to general procedure **E**, using 2-formylpyrazine 1-oxide **1m** (37 mg, 0.300 mmol, 1.00 equiv.), ethynylcyclohexane (78 μL, 0.600 mmol, 2.00 equiv.), $Rh(nbd)_2BF_4$ (5.6 mg, 0.015 mmol, 0.050 equiv.), DPEPhos (8.1 mg, 0.015 mmol, 0.050 equiv.) and $(CH_2Cl)_2$ (0.24 mL), with stirring for 18 h. Purification of the crude residue (>20:1 l:b) by column chromatography (SiO_2, 1:4 to 1:1 EtOAc:Petrol) yielded single isomer (*E*)-2-(3-cyclohexylacryloyl)pyrazine 1-oxide **2y** (58.6 mg, 0.254 mmol, 85%) as a pale-yellow viscous oil.

δ_H (400 MHz, $CDCl_3$) 8.72 (1H, s, *Ar*), 8.48 (1H, d, *J* = 4.2 Hz, *Ar*), 8.06 (1H, d, *J* = 4.1 Hz, *Ar*), 7.05 (1H, dd, *J* = 15.7, 6.7 Hz, C(5)*H*), 6.87 (1H, dd, *J* = 15.7, 1.3 Hz, C(4)*H*), 2.29-2.16 (1H, m, C(6)*H*), 1.85-1.69 (3H, m, C(7 to 9)H_2), 1.69-1.60 (1H, m, C(7 to 9)H_2), 1.37-1.11 (6H, m, C(7 to 9)H_2); **δ_C** (101 MHz, $CDCl_3$) 185.2, 156.6, 149.3, 148.5, 142.3, 134.7, 125.5, 41.1, 31.6, 26.0, 25.8; **ν_{max}**/cm^{-1} ($CHCl_3$) 3098, 2924, 2851, 1665, 1613, 1583, 1507, 1446, 1410, 1324, 1019, 979, 967; **m/z HRMS** (ESI^+) $[M + H]^+$ $C_{13}H_{17}O_2N_2^+$ calc. 233.1285, found 233.1284.

(*E*)-6-(3-Cyclohexylacryloyl)pyridazine 1-oxide (2z)

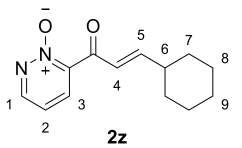

2z

Prepared according to general procedure **E**, using 6-formylpyridazine 1-oxide **1n** (24.8 mg, 0.200 mmol, 1.00 equiv.), ethynylcyclohexane (52 µL, 0.400 mmol, 2.00 equiv.), $Rh(nbd)_2BF_4$ (3.7 mg, 0.010 mmol, 0.050 equiv.), DPEPhos (5.4 mg, 0.010 mmol, 0.050 equiv.) and $(CH_2Cl)_2$ (0.16 mL), with stirring for 18 h. Purification of the crude residue (>20:1 l:b) by column chromatography (SiO_2, 1:4 to 2:3 EtOAc:Petrol) yielded single isomer (*E*)-6-(3-cyclohexylacryloyl)pyridazine 1-oxide **2z** (34.5 mg, 0.149 mmol, 75%) as a beige powdered solid.

m.p 82-83 °C (CH_2Cl_2); **δ_H** (400 MHz, $CDCl_3$) 8.52 (1H, dd, *J* = 5.3, 2.6 Hz, *Ar*), 7.94 (1H, dd, *J* = 7.9, 2.5 Hz, *Ar*), 7.13 (1H, dd, *J* = 7.9, 5.3 Hz, *Ar*), 7.07 (1H, dd, *J* = 15.6, 6.6 Hz, C(5)*H*), 6.94 (1H, dd, *J* = 15.7, 1.3 Hz, C(6)*H*), 2.28-2.18 (1H, m, C(6)*H*), 1.85-1.70 (4H, m, C(7 to 9)H_2), 1.71-1.61 (1H, m, C(7 to 9)H_2), 1.37-1.10 (5H, m, C(7 to 9)H_2); **δ_C** (101 MHz, $CDCl_3$) 185.6, 156.3, 151.9, 142.1, 135.4, 124.7, 116.3, 41.0, 31.6, 26.0, 25.7; **ν_{max}**/cm^{-1} ($CHCl_3$) 2974, 2923, 2851, 1666, 1614, 1538, 1449, 1381; **m/z HRMS** (ESI^+) $[M + Na]^+$ $C_{13}H_{16}O_2N_2{}^{23}Na^+$ calc. 255.1104, found 255.1105.

5. Product Derivatisations and Deoxygenations

(*E*)-3-Cyclohexyl-1-(pyridin-2-yl)prop-2-en-1-one (4)

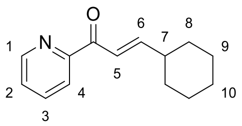

4

Prepared according to a modified literature procedure.[15] To a stirred solution of (*E*)-2-(3-cyclohexylacryloyl)pyridine 1-oxide **2b** (23 mg, 0.100 mmol, 1.00 equiv.) in dry toluene (0.5 mL, 0.2 M) was added PCl_3 (10.5 μL, 0.120 mmol, 1.20 equiv.) dropwise at room temperature and the resulting mixture was stirred for 15 min. Once complete by TLC, the mixture was quenched with sat. aq. $NaHCO_3$ (2.5 mL) and the mixture was stirred for an additional 5 min. The mixture was then extracted in CH_2Cl_2 (3 × 10 mL), the combined organic layers were dried over Na_2SO_4 and concentrated *in vacuo*. Purification of the crude residue by column chromatography (SiO_2, 9:1 to 4:1 Petrol:Et_2O) yielded (*E*)-3-cyclohexyl-1-(pyridin-2-yl)prop-2-en-1-one **4** (15.4 mg, 0.072 mmol, 72%) as a colourless oil.

Or alternatively prepared via the following two-step one-pot method:

The first hydroacylation step was according to general procedure **E**, using 2-formylpyridine 1-oxide **1a** (37 mg, 0.300 mmol, 1.00 equiv.), ethynylcyclohexane (78 μL, 0.600 mmol, 2.00 equiv.), $Rh(nbd)_2BF_4$ (5.6 mg, 0.015 mmol, 0.050 equiv.), DPEPhos (8.1 mg, 0.015 mmol, 0.050 equiv.) and $(CH_2Cl)_2$ (0.24 mL), with stirring for 18 h. After this time, the reaction mixture was cooled to room temperature and diluted with dry $(CH_2Cl)_2$ (1.26 mL, 0.2 M total conc.). PCl_3 (31.4 μL, 0.360 mmol, 1.20 equiv.) was then added dropwise to this solution, and the resulting mixture was stirred for 15 min. Once complete by TLC, the mixture was quenched with sat. aq. $NaHCO_3$ (2.5 mL) and the mixture was stirred for an additional 5 min. The mixture was then extracted in CH_2Cl_2 (3 × 10 mL), the combined organic layers were dried over Na_2SO_4

and concentrated *in vacuo*. Purification of the crude residue (>20:1 l:b) by column chromatography (SiO_2, 9:1 to 4:1 Petrol:Et_2O) yielded single isomer (*E*)-3-cyclohexyl-1-(pyridin-2-yl)prop-2-en-1-one **4** (29.7 mg, 0.138 mmol, 46%) as a colourless oil.

δ_H (400 MHz, $CDCl_3$) 8.69 (1H, ddd, *J* = 4.8, 1.8, 0.9 Hz, *Ar*), 8.11 (1H, app dt, *J* = 7.9, 1.2 Hz, *Ar*), 7.83 (1H, app td, *J* = 7.7, 1.7 Hz, *Ar*), 7.56 (1H, dd, *J* = 15.8, 1.4 Hz, C(5)*H*), 7.45 (1H, ddd, *J* = 7.6, 4.8, 1.3 Hz, *Ar*), 7.18 (1H, dd, *J* = 15.8, 6.9 Hz, C(6)*H*), 2.36-2.22 (1H, m, C(7)*H*), 1.90-1.82 (2H, m, C(8 to 10)H_2), 1.82-1.74 (2H, m, C(8 to 10)H_2), 1.73-1.64 (1H, m, C(8 to 10)H_2), 1.39-1.17 (5H, m, C(8 to 10)H_2); **δ_C** (101 MHz, $CDCl_3$) 190.0, 155.4, 154.4, 148.9, 137.1, 126.8, 123.0, 122.1, 41.4, 32.0, 26.1, 25.9; **ν_{max}**/cm^{-1} ($CHCl_3$) 2925, 2852, 1677, 1620, 1583, 1333, 1320, 1020, 993, 964, 743; **m/z HRMS** (ESI^+) $[M + H]^+$ $C_{14}H_{18}ON^+$ calc. 216.1383, found 216.1384. *Data is consistent with literature*[16]

(*E*)-1-(6-(*Tert*-butylamino)pyridin-2-yl)-3-cyclohexylprop-2-en-1-one (5)

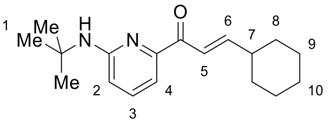

5

Reaction setup according to a modified literature procedure.[17] To a stirred solution of (*E*)-2-(3-cyclohexylacryloyl)pyridine 1-oxide **2b** (23 mg, 0.100 mmol, 1.00 equiv.) and *t*-butylamine (74 μL, 0.700 mmol, 7.00 equiv.) in 2.5:1 $PhCF_3$:CH_2Cl_2 (0.7 mL) was added Ts_2O (82 mg, 0.250 mmol, 2.50 equiv.) portion-wise, and the mixture was heated to 30 °C with stirring. After 30 min, starting material could still be observed by TLC, so more *t*-butylamine (11 μL, 0.100 mmol, 1.00 equiv.) and Ts_2O (32.8 mg, 0.100 mmol, 1.00 equiv.) were added and the reaction was stirred overnight (18 h). After this time, the mixture was cooled to room temperature and quenched with H_2O (1 mL) and the mixture was concentrated *in vacuo*. Purification by column

chromatography (SiO_2, 1:19 EtOAc:Petrol) yielded (*E*)-1-(6-(tert-butylamino)pyridin-2-yl)-3-cyclohexylprop-2-en-1-one **5** (16 mg, 0.056 mmol, 56%) as a yellow powdered solid.

m.p 84-87 °C (CH_2Cl_2); **δ_H** (400 MHz, $CDCl_3$) 7.55-7.42 (2H, m, *Ar* and C(5)*H*), 7.36 (1H, dd, *J* = 7.4, 0.9 Hz, *Ar*), 7.11 (1H, dd, *J* = 15.9, 6.4 Hz, C(6)*H*), 6.51 (1H, d, *J* = 8.3 Hz, *Ar*), 4.47 (1H, bs, N*H*), 2.31-2.19 (1H, m, C(7)*H*), 1.91-1.80 (2H, m, C(8 to 10)H_2), 1.78 (2H, app dt, *J* = 12.1, 3.4 Hz, C(8 to 10)H_2), 1.72-1.65 (1H, m, C(8 to 10)H_2), 1.50 (9H, s, C(1)H_3), 1.41-1.15 (5H, m, C(8 to 10)H_2); **δ_C** (101 MHz, $CDCl_3$) 190.9, 157.5, 153.8, 152.5, 137.5, 122.8, 113.0, 111.7, 51.3, 41.0, 31.9, 29.4, 26.2, 26.0; **ν_{max}**/cm^{-1} ($CHCl_3$) 3386, 2980, 2926, 2853, 1671, 1617, 1593, 1517, 1506, 1453, 1388, 1360, 1226, 989, 964, 804; **m/z HRMS** (ESI^+) $[M + H]^+$ $C_{18}H_{27}ON_2^+$ calc. 287.2118, found 287.2118.

2-(1-(*Tert*-butoxycarbonyl)-1H-pyrrol-2-yl)pyridine 1-oxide (6)

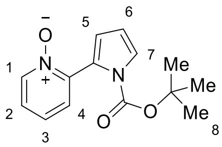

6

First step undertaken according to general procedure **E**, using 2-formylpyridine 1-oxide **1a** (37 mg, 0.300 mmol, 1.00 equiv.), *t*-butyl prop-2-yn-1-ylcarbamate (93.1 µL, 0.600 mmol, 2.00 equiv.), $Rh(nbd)_2BF_4$ (5.6 mg, 0.015 mmol, 0.050 equiv.), DPEPhos (8.1 mg, 0.015 mmol, 0.050 equiv.) and $(CH_2Cl)_2$ (0.24 mL), ***with stirring at room temperature for 21 h***. After this time, the reaction mixture was diluted with $(CH_2Cl)_2$ (3 mL, 0.09 M total conc.) and *p*-toluene sulfonic acid monohydrate (86 mg, 0.450 mmol, 1.50 equiv.) was added. The mixture was then stirred at room temperature for a further 3 h before addition of sat. aq. $NaHCO_3$ (10 mL). The layers were separated and the aqueous layer was extracted in CH_2Cl_2 (3 × 10 mL). The combined organic layers were dried over Na_2SO_4 and concentrated *in vacuo*. Purification of the crude residue by column chromatography (SiO_2, 4:1 CH_2Cl_2:Acetone) yielded 2-(1-(tert-

butoxycarbonyl)-1H-pyrrol-2-yl)pyridine 1-oxide **6** (30.5 mg, 0.117 mmol, 39%) as a yellow/orange solid. (The second step conditions were modified from a literature procedure)[18]

m.p 109-111 °C (CH_2Cl_2); **δ_H** (400 MHz, $CDCl_3$) 8.33 (1H, dd, *J* = 6.2, 1.6 Hz, *Ar*), 7.45 (1H, dd, *J* = 3.3, 1.7 Hz, *Ar*), 7.42-7.38 (1H, m, *Ar*), 7.35-7.22 (2H, m, *Ar*), 6.36 (1H, dd, *J* = 3.4, 1.8 Hz, *Ar*), 6.28 (1H, t, *J* = 3.3 Hz, *Ar*), 1.40 (9H, s, C(8)H_3); **δ_C** (101 MHz, $CDCl_3$) 148.7, 144.5, 139.6, 127.4, 125.0, 125.0, 124.8, 123.7, 116.6, 110.7, 83.9, 27.7; **ν_{max}**/cm^{-1} ($CHCl_3$) 2979, 2932, 1744, 1321, 1257, 1151, 849, 768; **m/z HRMS** (ESI^+) $[M + H]^+$ $C_{14}H_{17}O_3N_2^+$ calc. 261.1234, found 261.1235.

6. Additional Findings

Non-isolated Scope Products due to Incompatible Alkyne and Aldehyde Substrates:[a,b]

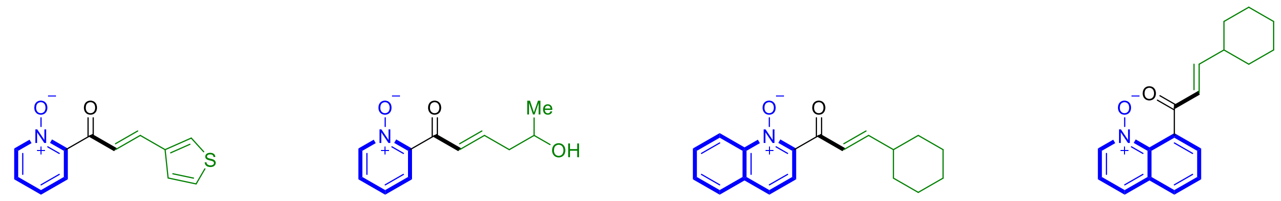

2aa
47% NMR yield, >20:1 l:b, 86% aldehyde consumption, 47% isolated impure - inseparable from aldehyde and other aliphatic impurities

2ab
>99% aldehyde consumption, 50% NMR yield, 8.2:1 l:b, 56% isolated with many impurities due to product degrdation during chromatography

2ac
0%, >99% aldehyde consumption, reaction yielded a complex mixture

2ad
0%, no conversion to product at 55 °C or 80 °C

Scheme S1: *A list of failed reaction substrates under the standard optimised conditions; [a] Aldehyde consumption, and ^{1}H NMR yields are relative to ^{1}H NMR standard methyl-3,5-dinitrobenzoate; [b] Crude regioselectivity determined by crude ^{1}H NMR analysis.*

Unsuccessful hydroacylations of terminal alkenes:[a,b]

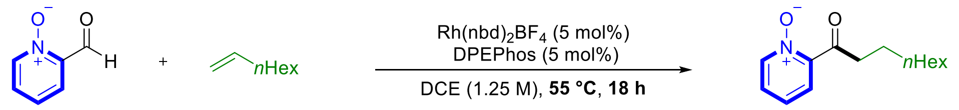

1a 1.0 equiv. 2.0 equiv.

2ae
23% aldehyde consumption[a]
- no detectable product

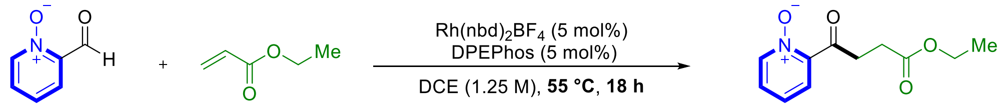

1a 1.0 equiv. 2.0 equiv.

2af
33% aldehyde consumption[a]
- no detectable product

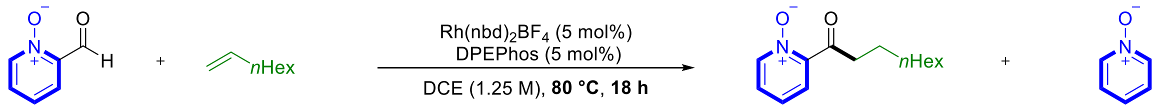

1a 1.0 equiv. 2.0 equiv.

2ae
0% NMR yield, >99% aldehyde consumption[a]

Py-*N*-Oxide
59% NMR yield[a,b]

Scheme S2: *Hydroacylation reaction conditions applied to terminal alkenes; [a] Aldehyde consumption and NMR yields are relative to NMR standard methyl-3,5-dinitrobenzoate; [b] NMR signals of crude pyridine-N-oxide compared to an authentic sample.*

Hydroacylations of internal alkynes:[a,b,c]

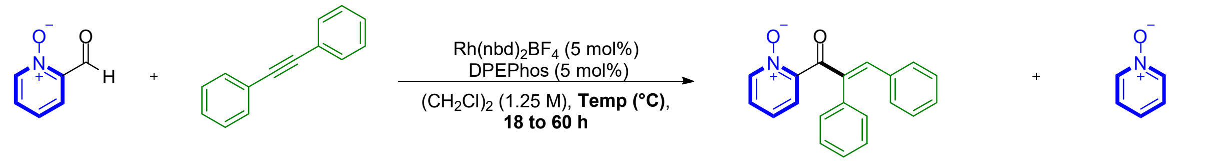

1a 1.0 equiv. 2.0 equiv. **2ag** **Py-*N*-Oxide**

**2ag**	**Py-*N*-Oxide**
**55 °C:** 1.4:1 **2ag**:**1a** in crude NMR, 18% isolated with starting material (60 h)[a]	**55 °C:** 0%[b,c]
**80 °C:** 0%, >99% aldehyde consumption (18 h)[b]	**80 °C:** 57%[b,c]
**100 °C:** 0%, >99% aldehyde consumption (18 h)[b]	**100 °C:** 56%[b,c]

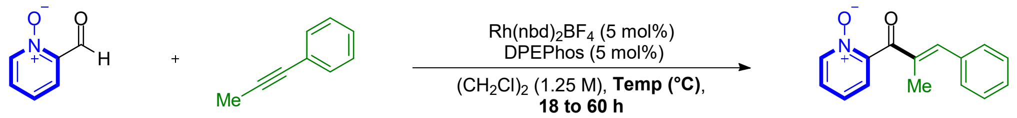

1a 1.0 equiv. 2.0 equiv. **2m**

55 °C: 18%,[d] 3.2:1 rr, 64% aldehyde consumption (72 h)[b]

80 °C: 39% isolated yield, 15:1 rr,[d] >99% aldehyde consumption (18 h)[b]

Scheme S3*: Hydroacylation attempts with internal alkynes 1,2-diphenylethyne and prop-1-yn-1-ylbenzene; [a]* ***2ag****:****1a*** *ratios were determined by crude ^{1}H NMR analysis; [b]Aldehyde consumption and NMR yields were based on ^{1}H NMR standard methyl-3,5-dinitrobenzoate; [c] ^{1}H NMR signals of crude pyridine-N-oxide compared to an authentic sample; [d] Crude regioselectivity was determined by crude ^{1}H NMR analysis.*

Control reactions testing for degradation *in-situ*:

A comparison of yield and aldehyde consumption between an overnight stir and 6 h:

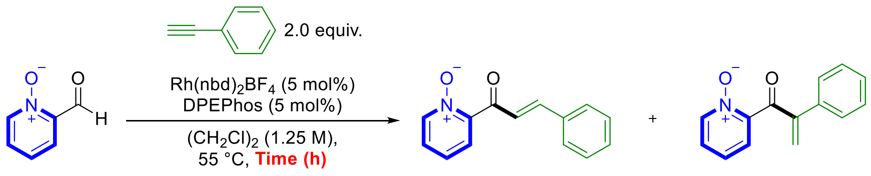

1a 1.0 equiv. **2g** **3g**

Entry	Time (h)	Aldehyde consumption (%)[a]	l:b[b]	Total Yield (%)[a]
1	6	64	6:1	58
2	18	>99	9:1	69(53)[c]

[a] Aldehyde consumption and total yield of both regioisomers calculated by crude ^{1}H NMR spectroscopy with respect to ^{1}H NMR standard methyl-3,5-dinitrobenoate; [b] l:b regioselectivity determined by crude ^{1}H NMR spectroscopy. [c] Isolated yield of the major linear isomer **2g** in the parenthesis

***Scheme S4**: A comparison of total product yield, selectivity and aldehyde consumption, for the hydroacylation reaction between aldehyde **1a** and phenylacetylene, at 6 h and 18 h.*

Subjecting 6-halo hydroacylation products 2q and 2r to Rh-DPEPhos - testing for product degradation from direct catalyst exposure:

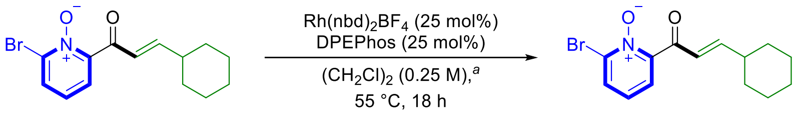

2q 1.0 equiv. (18.6 mg)

[a] Product concentration

2q 5.6 mg, 30% total product recovery

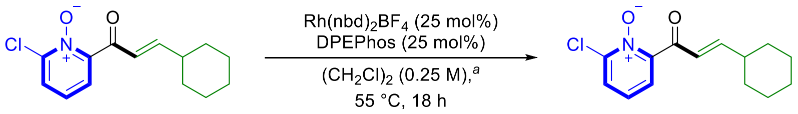

2r 1.0 equiv. (10.6 mg)

[a] Product concentration

2r 2.6 mg, 25% total product recovery

***Scheme S5:** Showing the direct degradation of hydroacylation products **2q** and **2r**, when directly subjected to Rh-DPEPhos at 55 °C.*

Testing for the degradation of hydroacylation product 2r during reaction progression:

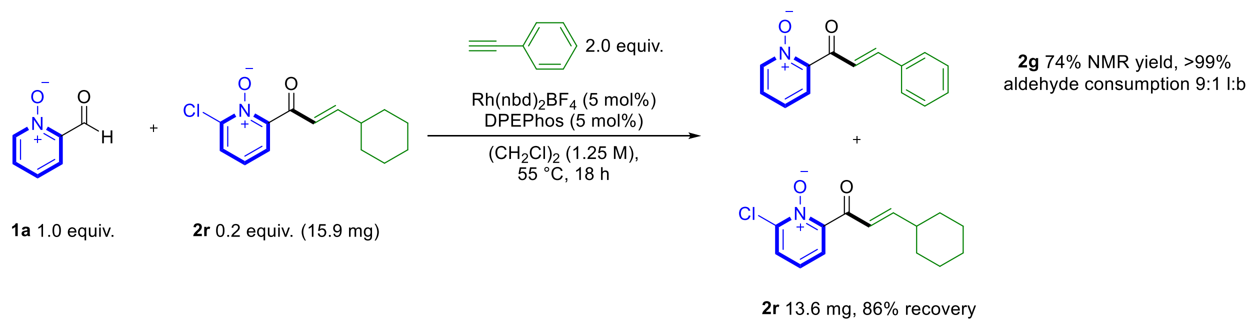

***Scheme S6**: Illustrating the degradation of hydroacylation product **2r**, in presence of a hydroacylation reaction between aldehyde **1a** and phenylacetylene.*

7. References

[1] L. Gu, K. Renault, A. Romieu, J. A. Richard, R. Srinivasan, *New J. Chem.* **2020**, *44*, 12208–12215.

[2] A. Landa, A. Minkkilä, G. Blay, K. A. Jergensen, *Chem. - A Eur. J.* **2006**, *12*, 3472–3483.

[3] D. Limnios, C. G. Kokotos, *Chem. - A Eur. J.* **2014**, *20*, 559–563.

[4] K. Pfleger, *Monatshefte fur Chemie* **1977**, *108*, 459–467.

[5] V. Piccialli, S. D'Errico, N. Borbone, G. Oliviero, R. Centore, S. Zaccaria, *European J. Org. Chem.* **2013**, *2*, 1781–1789.

[6] J. A. Weitgenant, J. D. Mortison, P. Helquist, *Org. Lett.* **2005**, *7*, 3609–3612.

[7] F. Roudesly, L. F. Veiros, J. Oble, G. Poli, *Org. Lett.* **2018**, *20*, 2346–2350.

[8] T. Kayima, Shozo;Masayuki, *Chem. Pharm. Bull.* **1980**, *28*, 529–534.

[9] Y. Mizuno, T. Endo, *J. Org. Chem.* **1978**, *43*, 684–688.

[10] V. Dhand, J. A. Draper, J. Moore, R. Britton, *Org. Lett.* **2013**, *15*, 1914–1917.

[11] P. K. Singh, V. K. Singh, *Org. Lett.* **2010**, *12*, 80–83.

[12] S. Barroso, G. Blay, J. R. Pedro, *Org. Lett.* **2007**, *9*, 1983–1986.

[13] L. Li, S. Zhang, Y. Hu, Y. Li, C. Li, Z. Zha, Z. Wang, *Chem. - A Eur. J.* **2015**, *21*, 12885–12888.

[14] S. Mukherjee, S. Mondal, A. Patra, R. G. Gonnade, A. T. Biju, *Chem. Commun.* **2015**, *51*, 9559–9562.

[15] H. C. Seung, J. H. Seung, S. Chang, *J. Am. Chem. Soc.* **2008**, *130*, 9254–9256.

[16] J. Bos, A. García-Herraiz, G. Roelfes, *Chem. Sci.* **2013**, *4*, 3578–3582.

[17] H. Sterckx, C. Sambiagio, V. Médran-Navarrete, B. U. W. Maes, *Adv. Synth. Catal.* **2017**, *359*, 3226–3236.

[18] M. K. Majhail, P. M. Ylioja, M. C. Willis, *Chem. - A Eur. J.* **2016**, *22*, 7879–7884.

8. NMR Spectra of Novel *N*-oxide Substrates and Scope Products

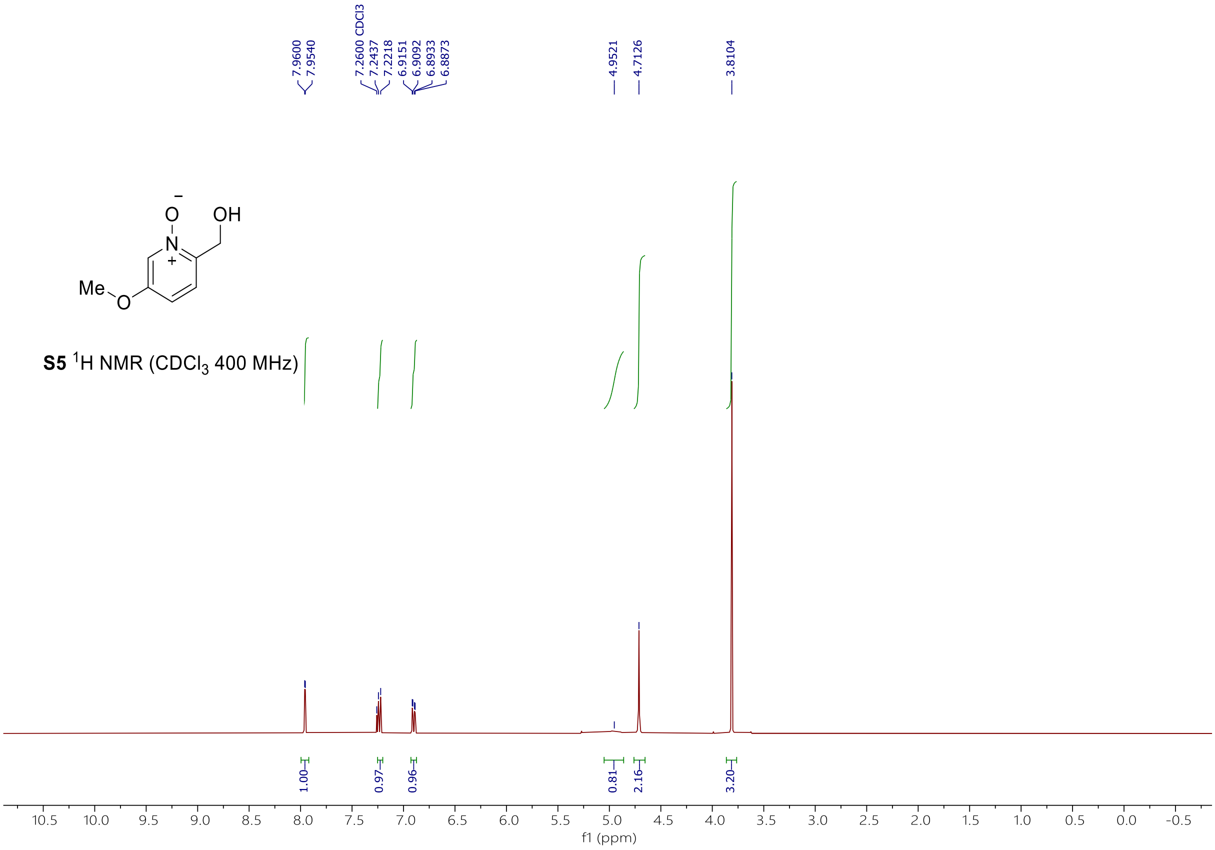

S5 ^{1}H NMR ($CDCl_3$ 400 MHz)

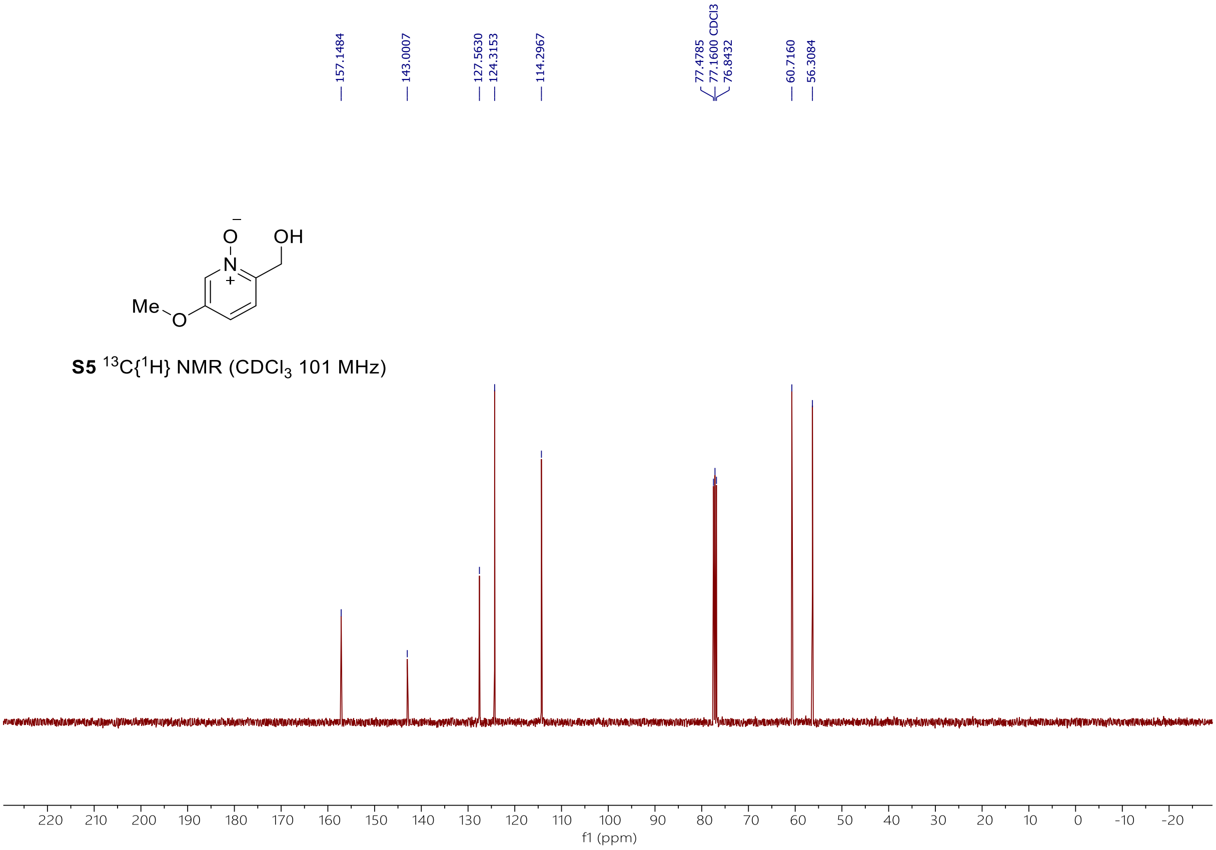

S5 ^{13}C{^{1}H} NMR ($CDCl_3$ 101 MHz)

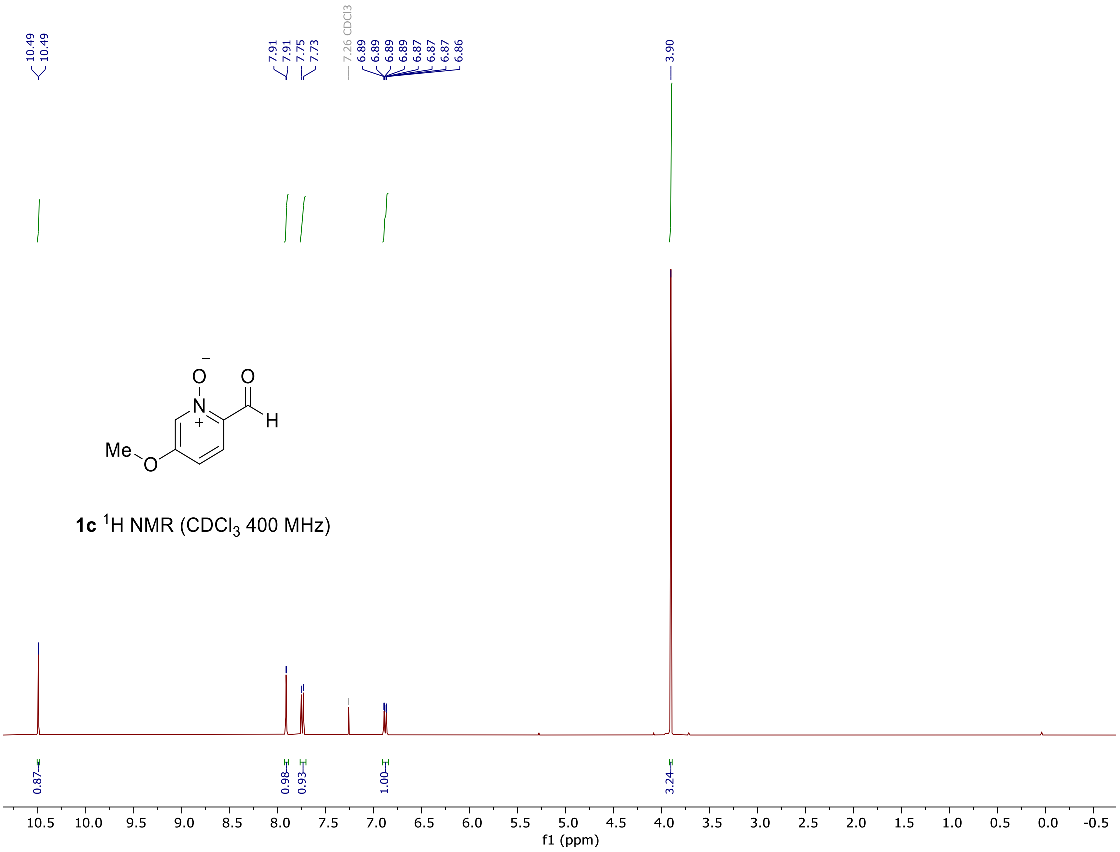

1c ^{1}H NMR ($CDCl_3$ 400 MHz)

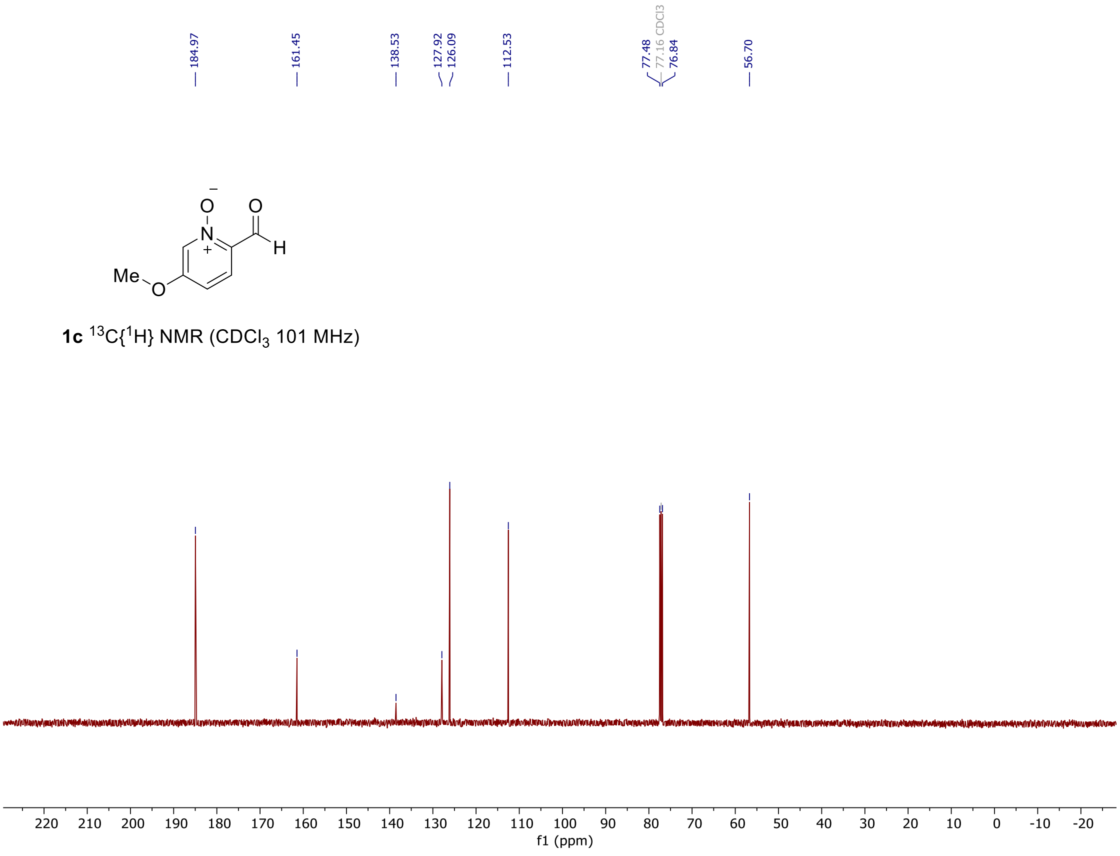

1c $^{13}C\{^{1}H\}$ NMR ($CDCl_3$ 101 MHz)

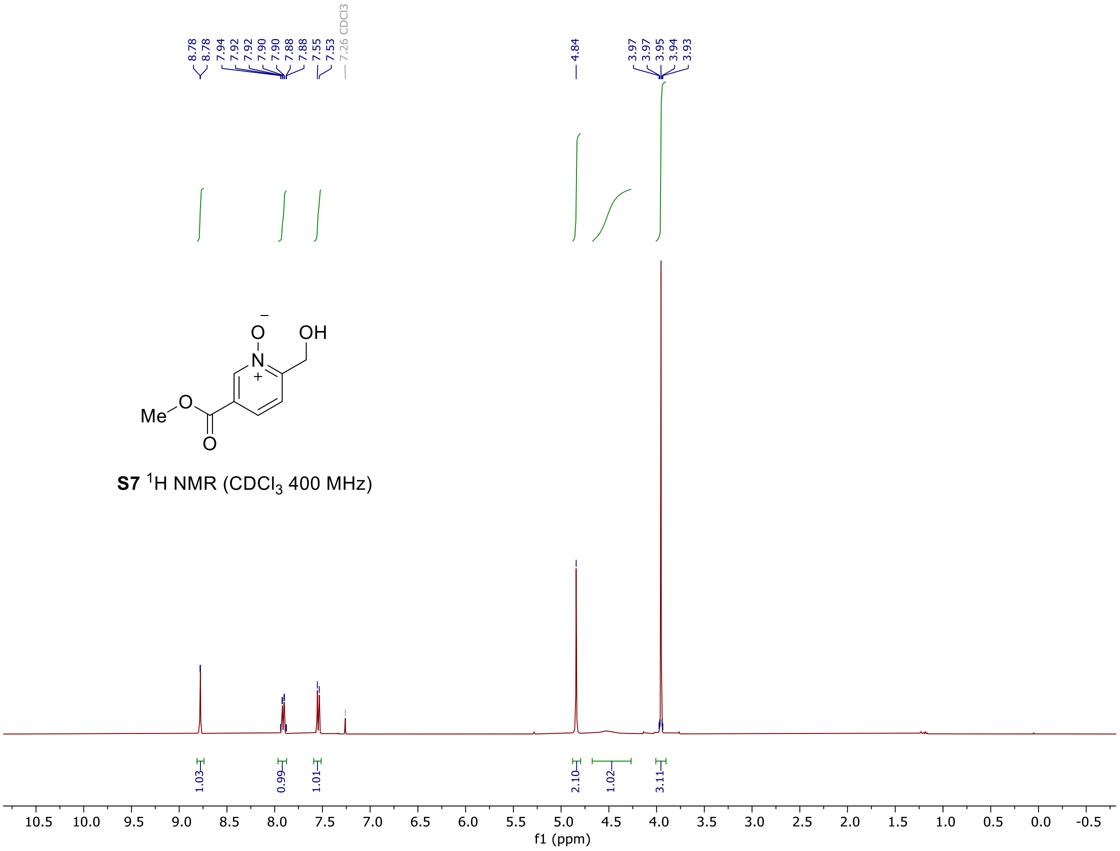

S7 ^{1}H NMR ($CDCl_3$ 400 MHz)

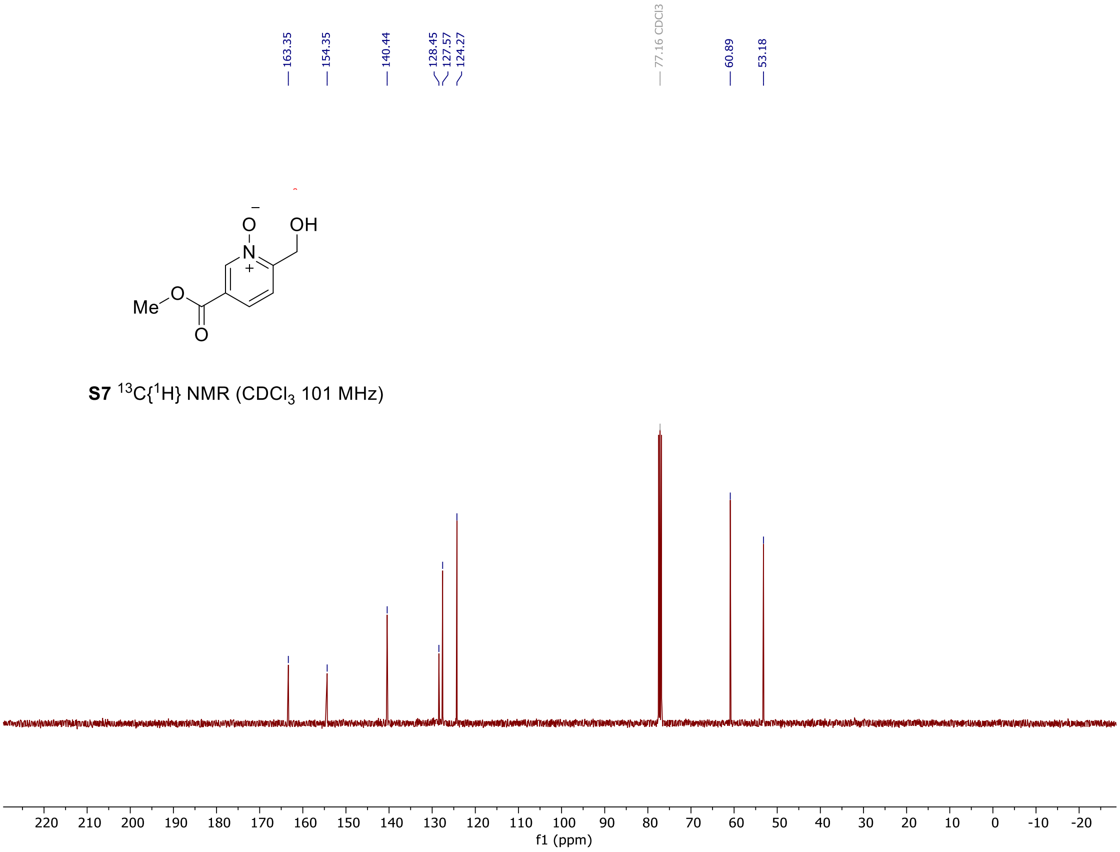

S7 $^{13}C\{^{1}H\}$ NMR ($CDCl_3$ 101 MHz)

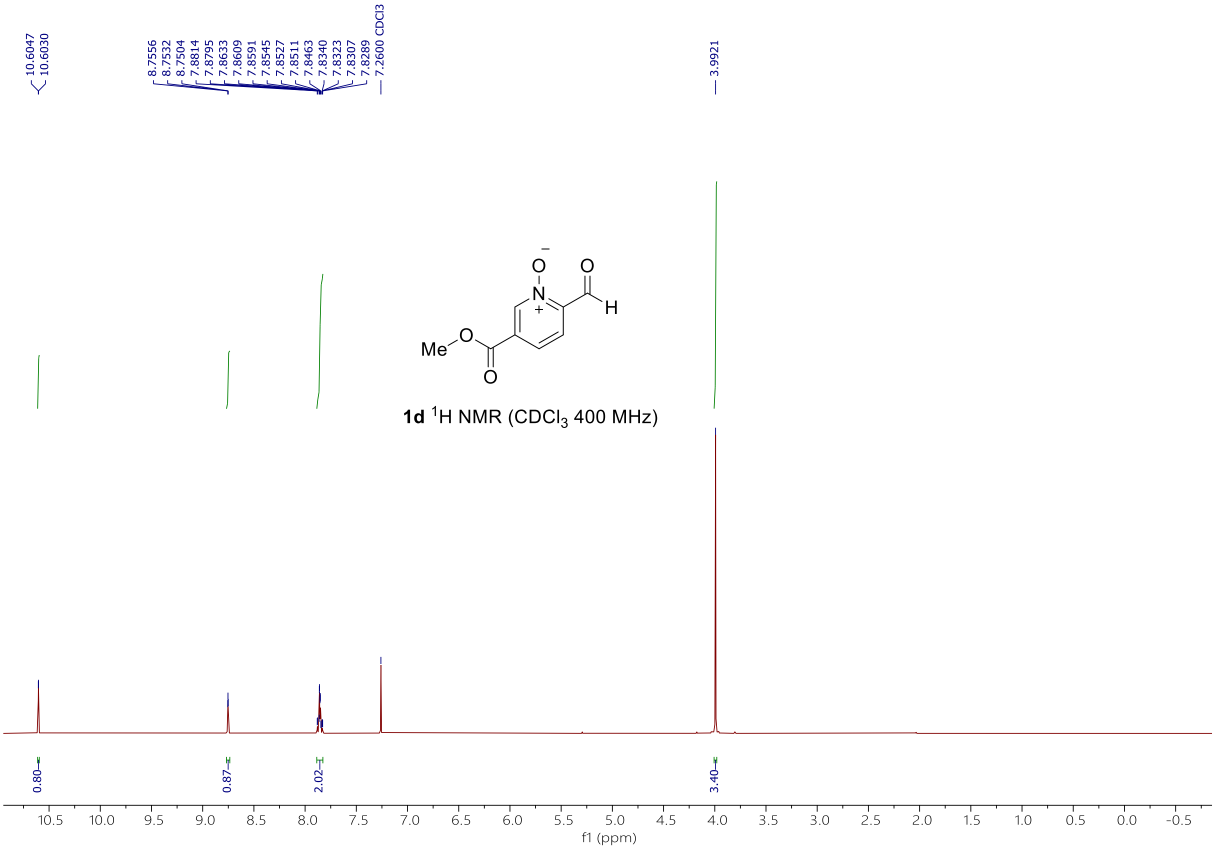

1d ^{1}H NMR ($CDCl_3$ 400 MHz)

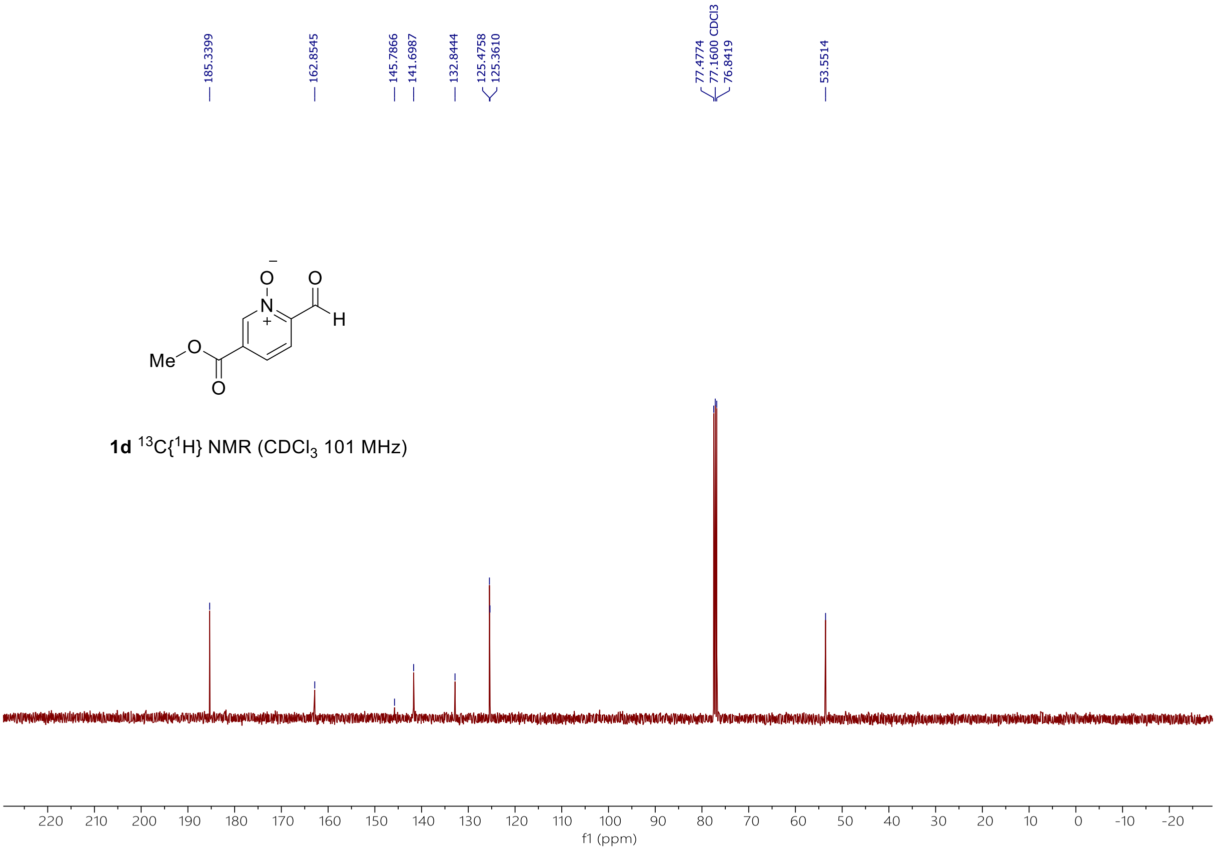

1d ^{13}C{^{1}H} NMR ($CDCl_3$ 101 MHz)

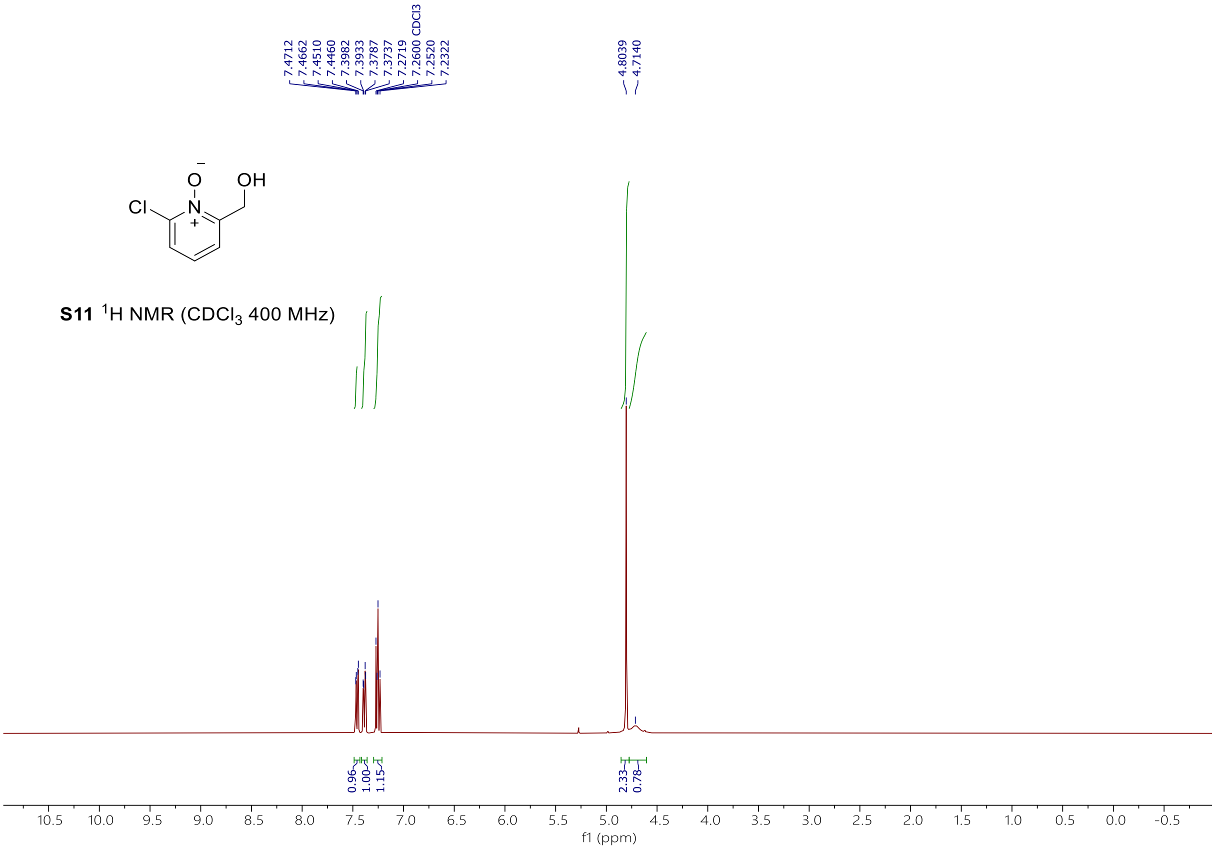

S11 ^{1}H NMR ($CDCl_3$ 400 MHz)

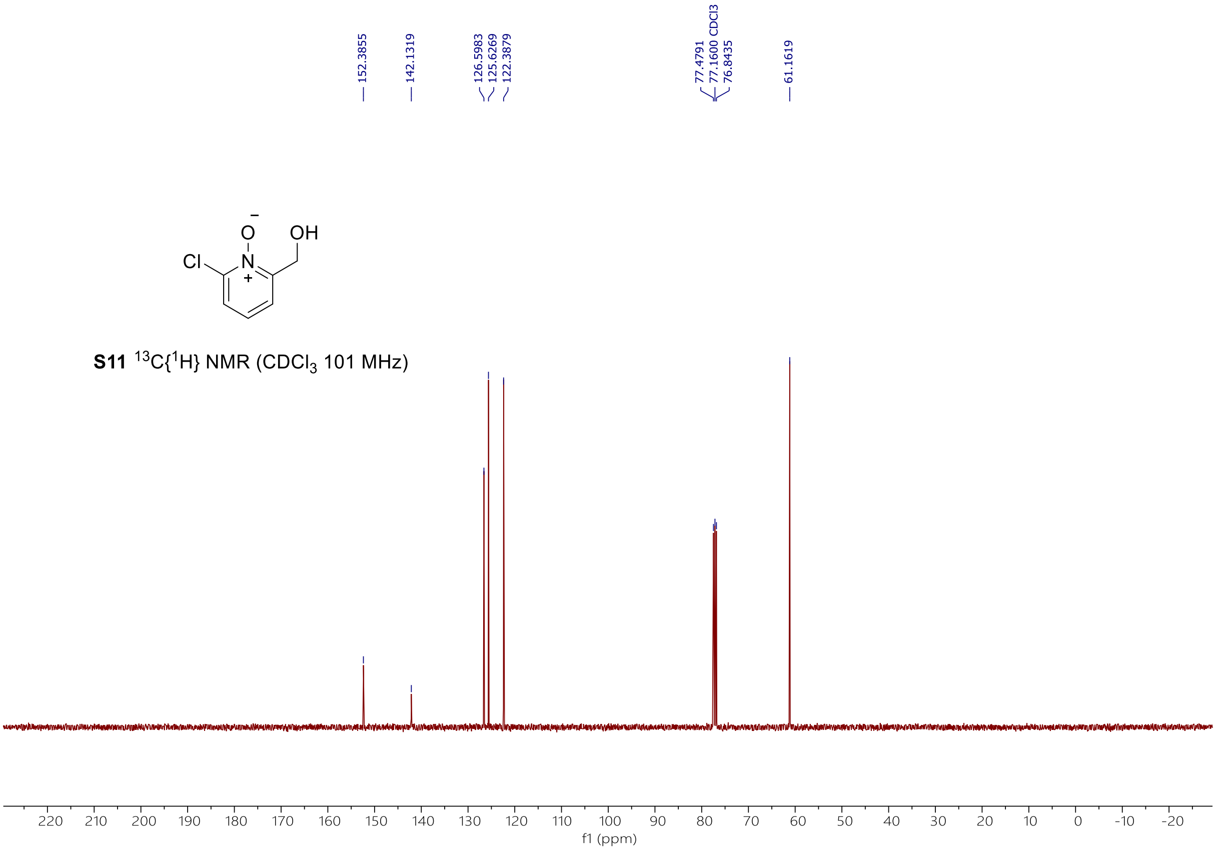

S11 $^{13}C\{^1H\}$ NMR ($CDCl_3$ 101 MHz)

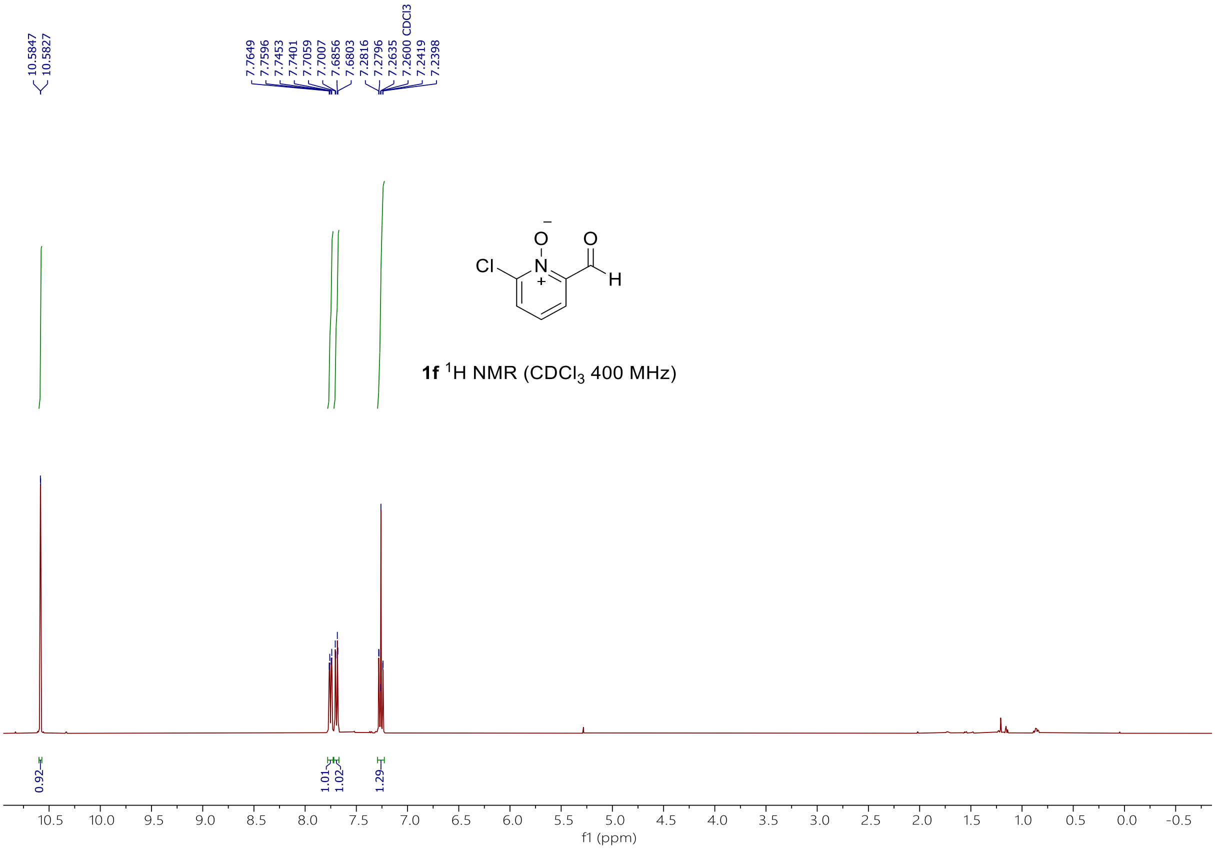
10.5847
10.5827
7.7649
7.7596
7.7453
7.7401
7.7059
7.7007
7.6856
7.6803
7.2816
7.2796
7.2635
7.2600 CDCl3
7.2419
7.2398
1f 1H NMR (CDCl3 400 MHz)
0.92
1.01
1.02
1.29
f1 (ppm)

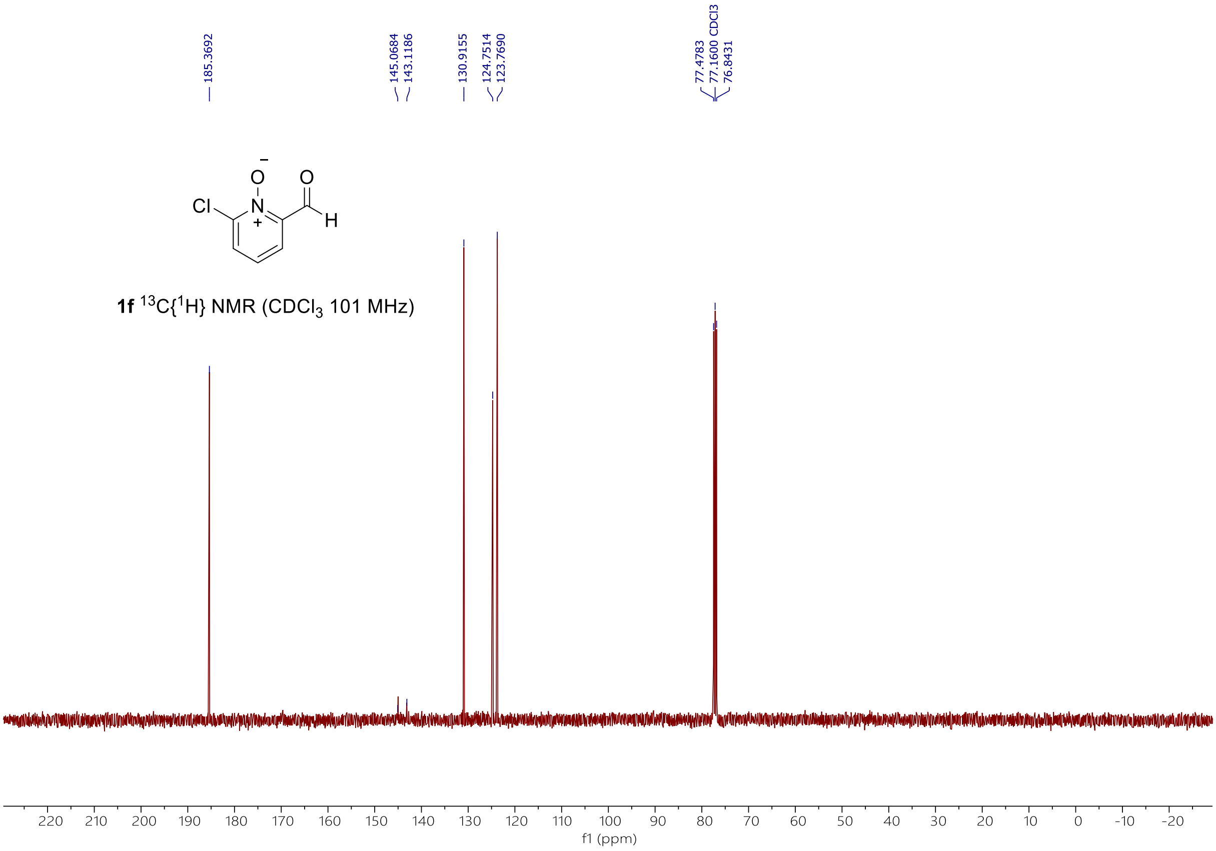
185.3692
145.0684
143.1186
130.9155
124.7514
123.7690
77.4783
77.1600 CDCl3
76.8431
1f 13C{1H} NMR (CDCl3 101 MHz)
f1 (ppm)

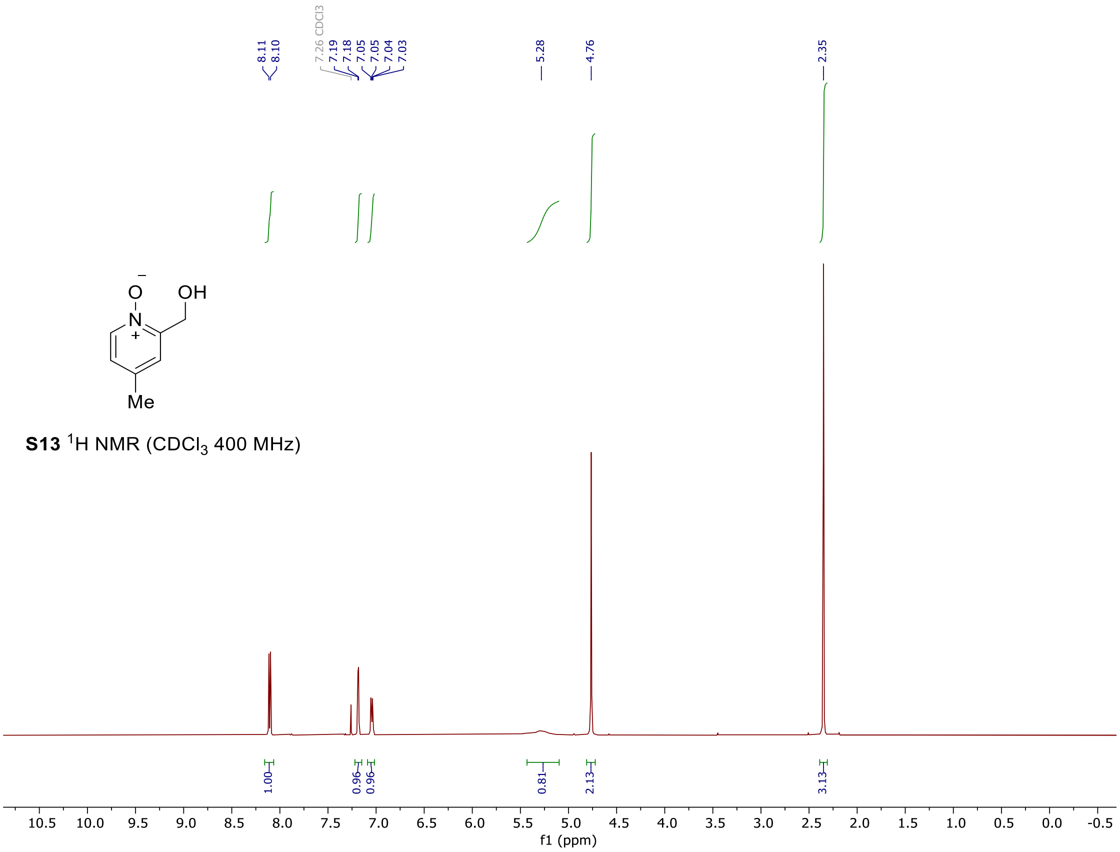

S13 ^{1}H NMR ($CDCl_3$ 400 MHz)

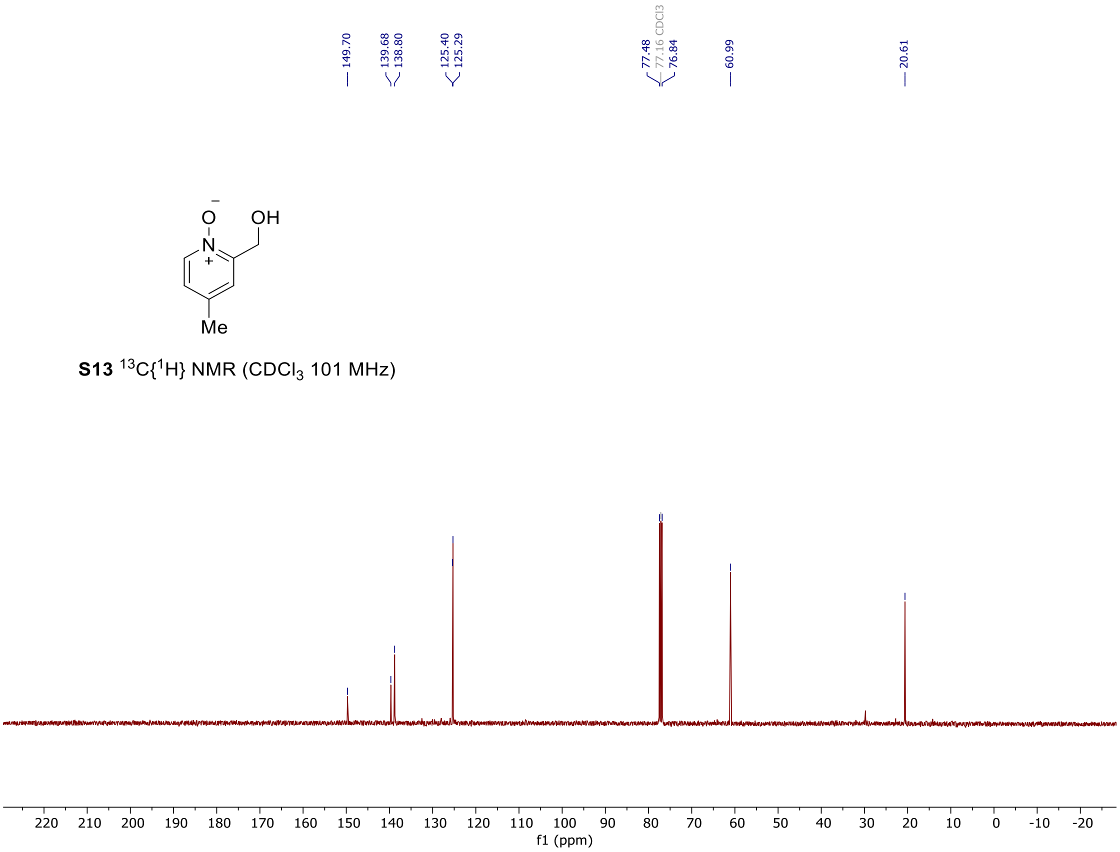

S13 $^{13}C\{^{1}H\}$ NMR ($CDCl_3$ 101 MHz)

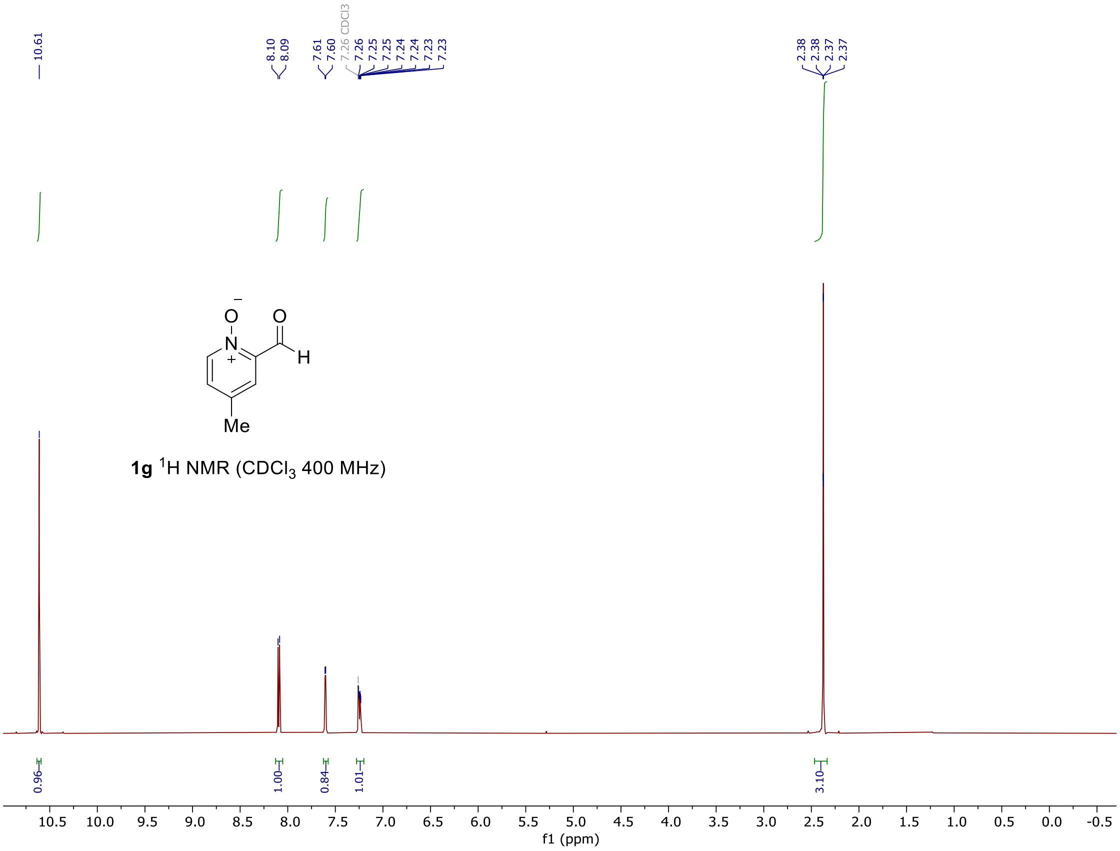

1g ^{1}H NMR ($CDCl_3$ 400 MHz)

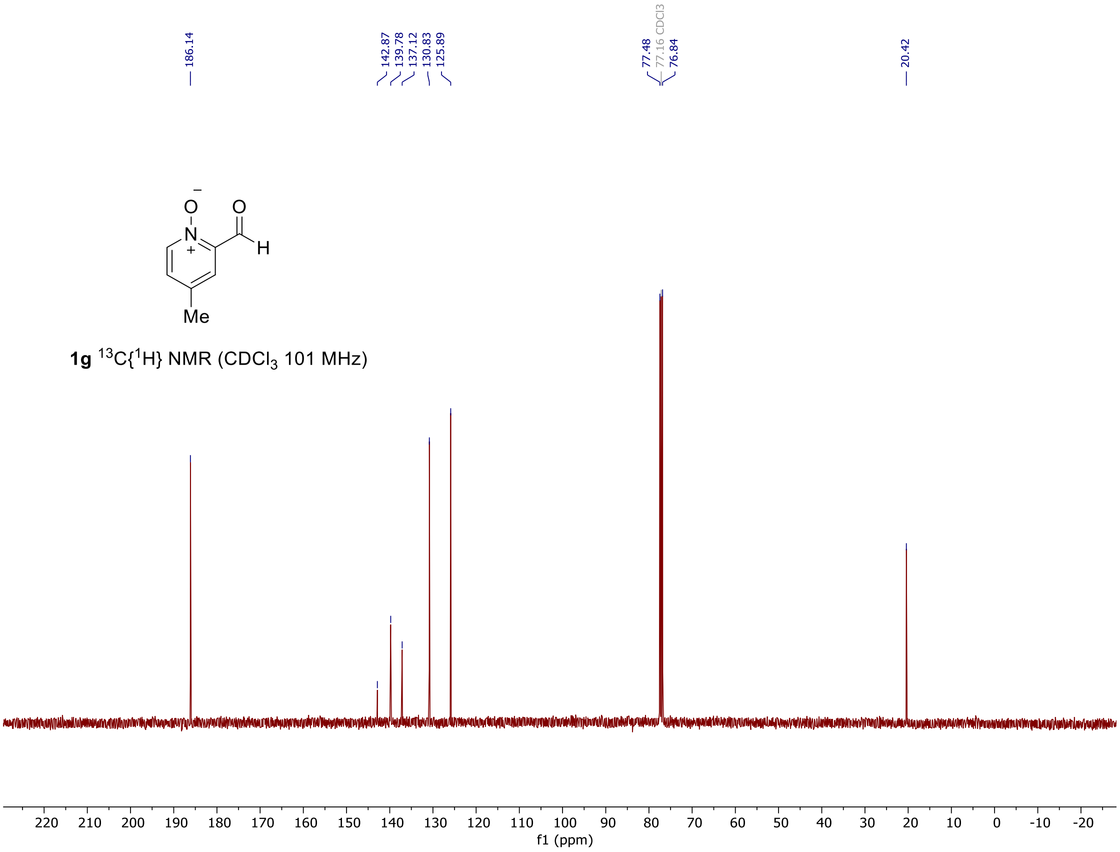

1g $^{13}C\{^{1}H\}$ NMR ($CDCl_3$ 101 MHz)

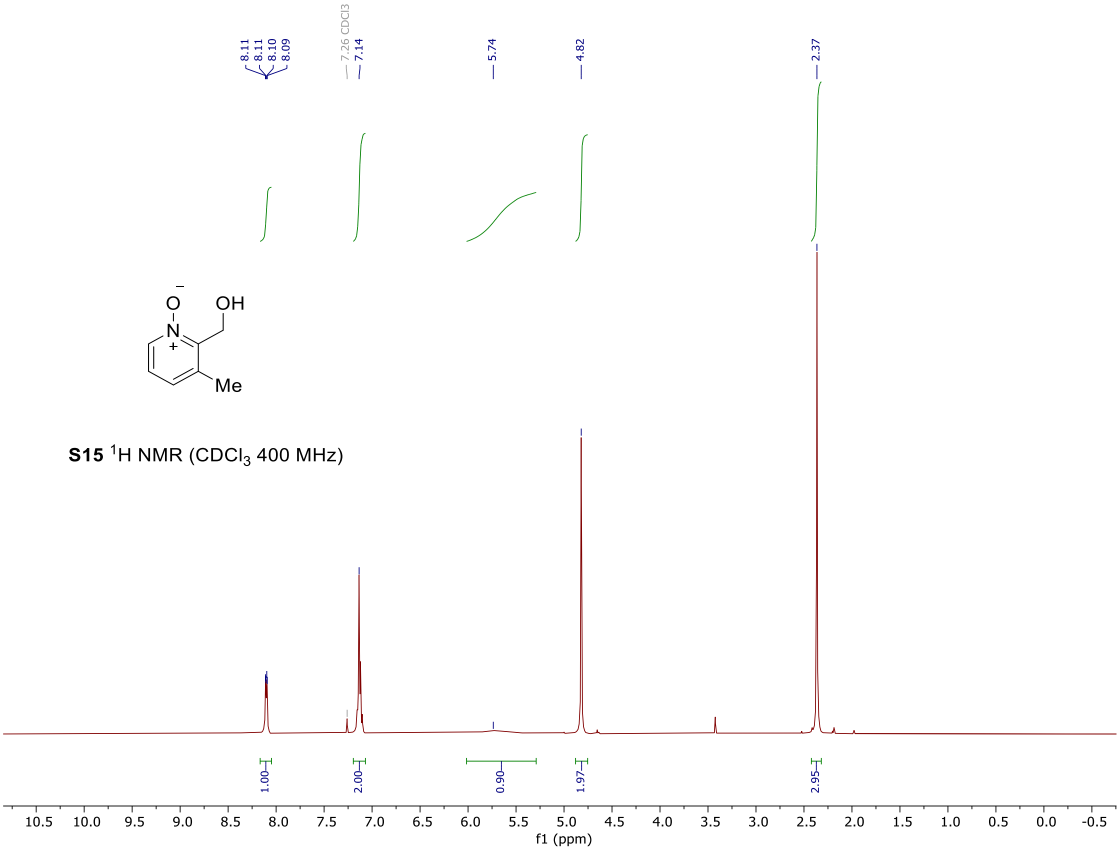

S15 ^{1}H NMR ($CDCl_3$ 400 MHz)

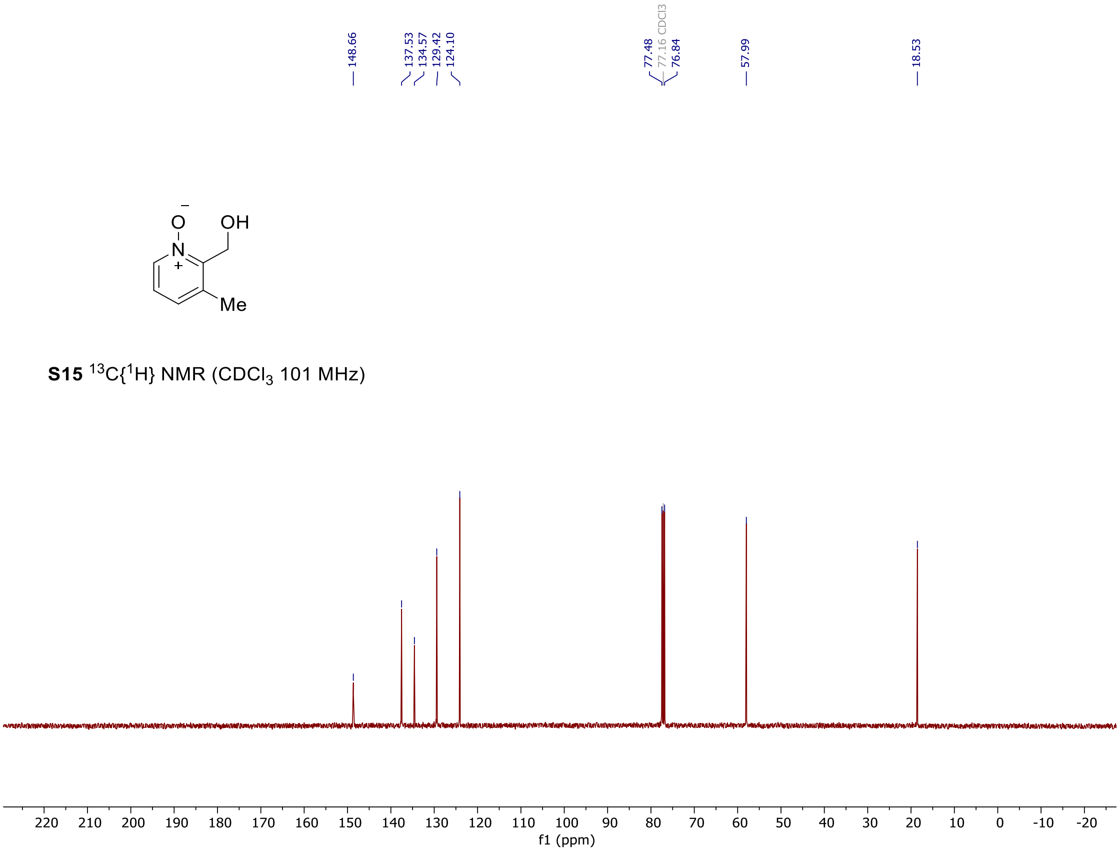

S15 $^{13}C\{^{1}H\}$ NMR ($CDCl_3$ 101 MHz)

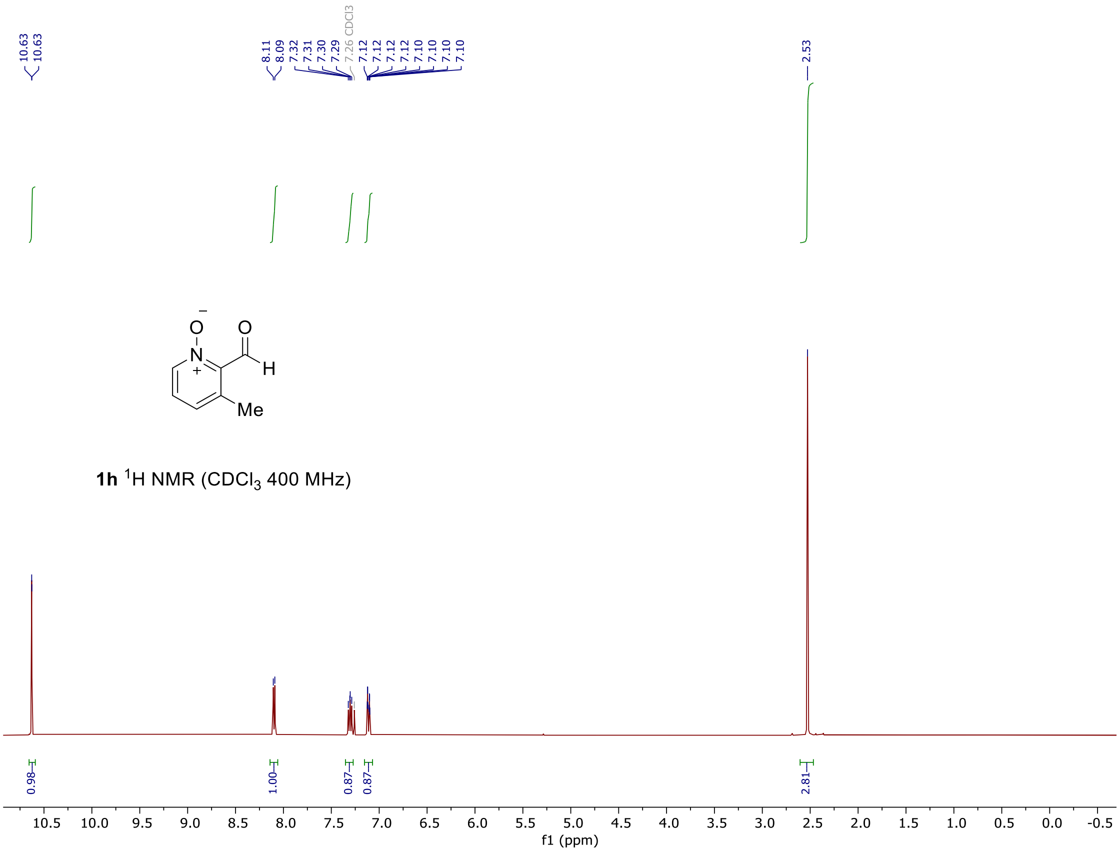
1h ¹H NMR (CDCl3 400 MHz)
10.63
10.63
8.11
8.09
7.32
7.31
7.30
7.29
7.26 CDCl3
7.12
7.12
7.12
7.12
7.10
7.10
7.10
7.10
2.53
0.98
1.00
0.87
0.87
2.81
f1 (ppm)

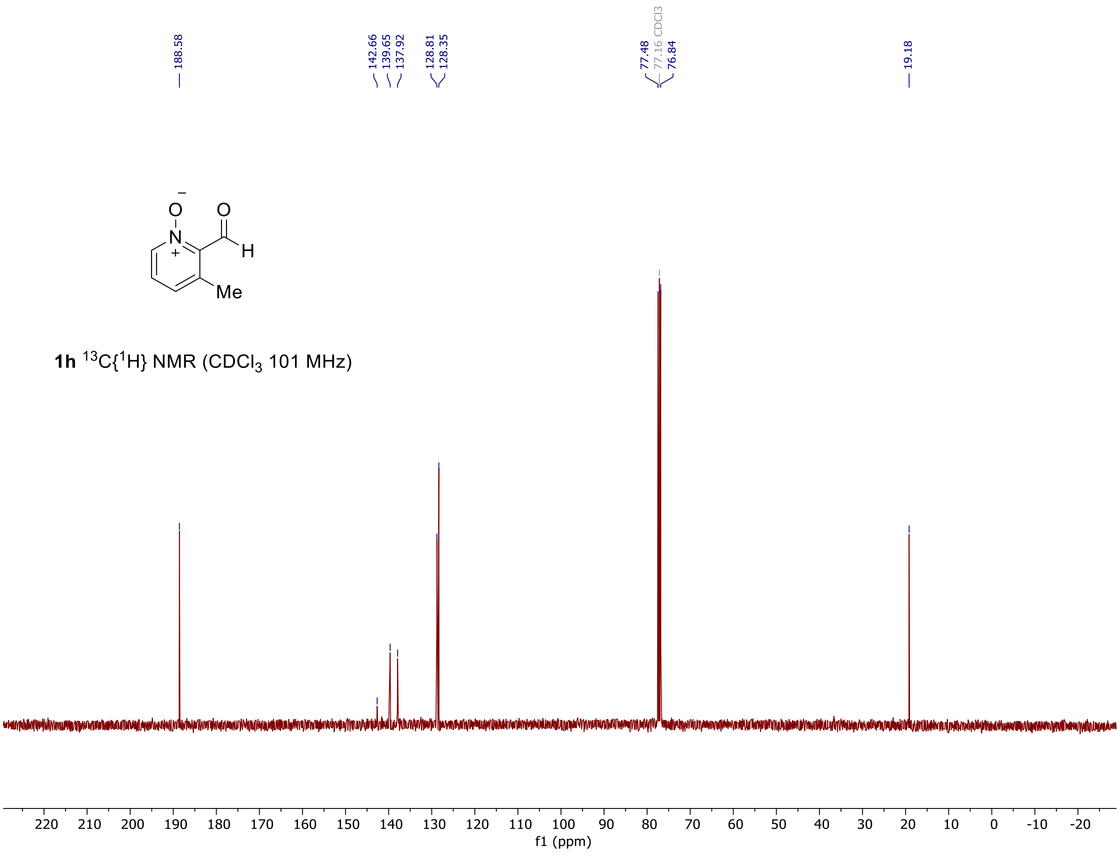
1h ¹³C{¹H} NMR (CDCl3 101 MHz)
188.58
142.66
139.65
137.92
128.81
128.35
77.48
77.16 CDCl3
76.84
19.18
f1 (ppm)

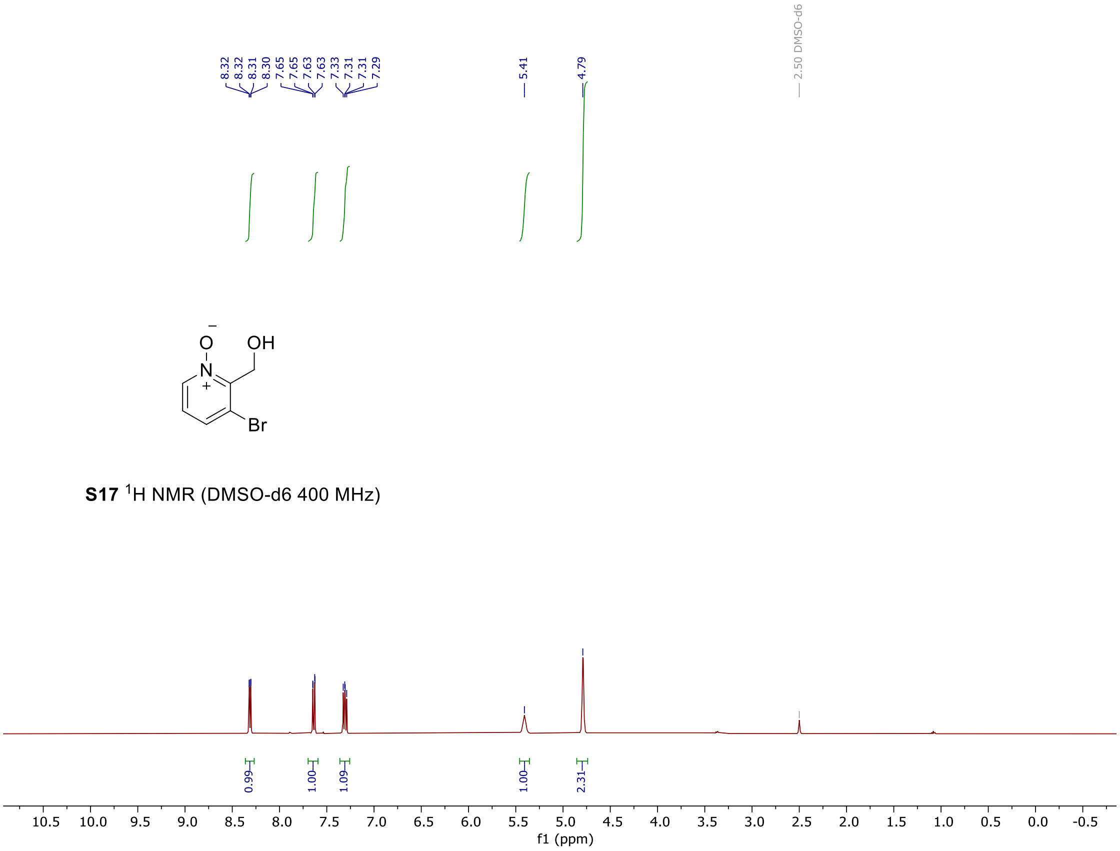

S17 ^{1}H NMR (DMSO-d6 400 MHz)

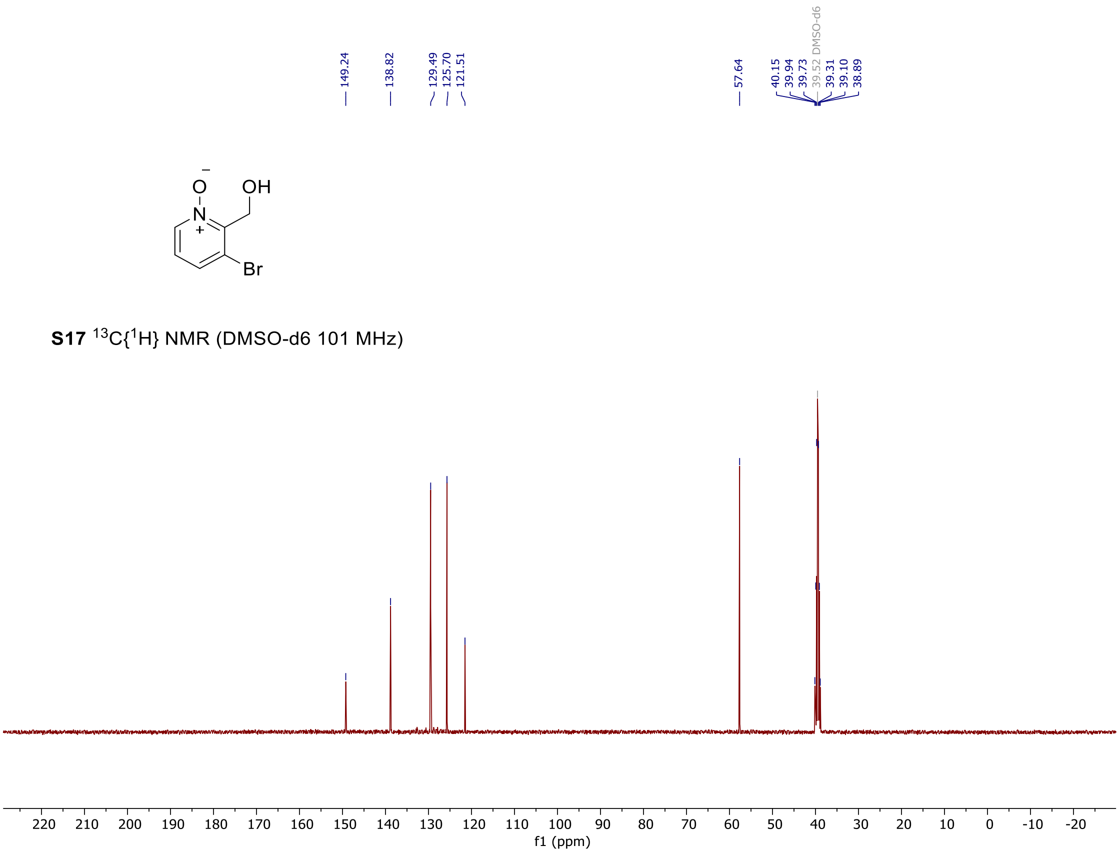

S17 ^{13}C{^{1}H} NMR (DMSO-d6 101 MHz)

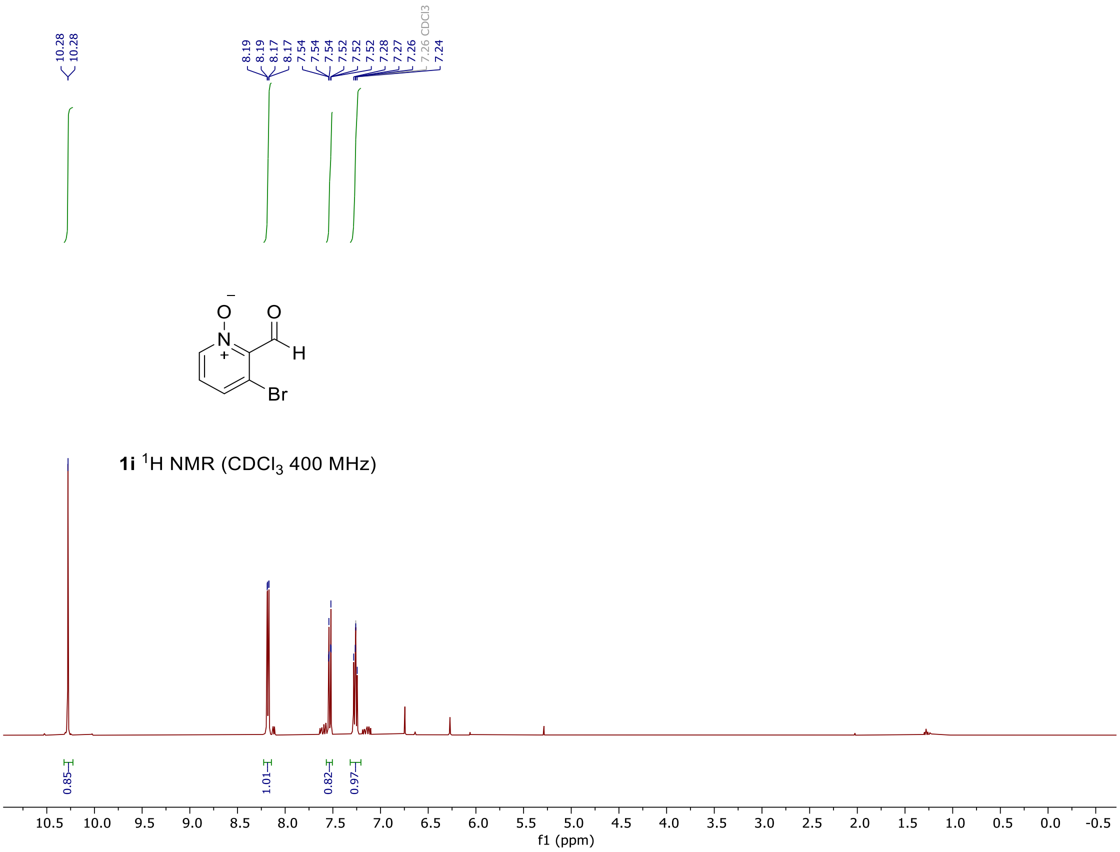
10.28
10.28
8.19
8.19
8.17
8.17
7.54
7.54
7.54
7.52
7.52
7.52
7.28
7.27
7.26 CDCl3
7.26
7.24
O
N
+
O
H
Br
1i 1H NMR (CDCl3 400 MHz)
0.85
1.01
0.82
0.97
10.5 10.0 9.5 9.0 8.5 8.0 7.5 7.0 6.5 6.0 5.5 5.0 4.5 4.0 3.5 3.0 2.5 2.0 1.5 1.0 0.5 0.0 -0.5
f1 (ppm)

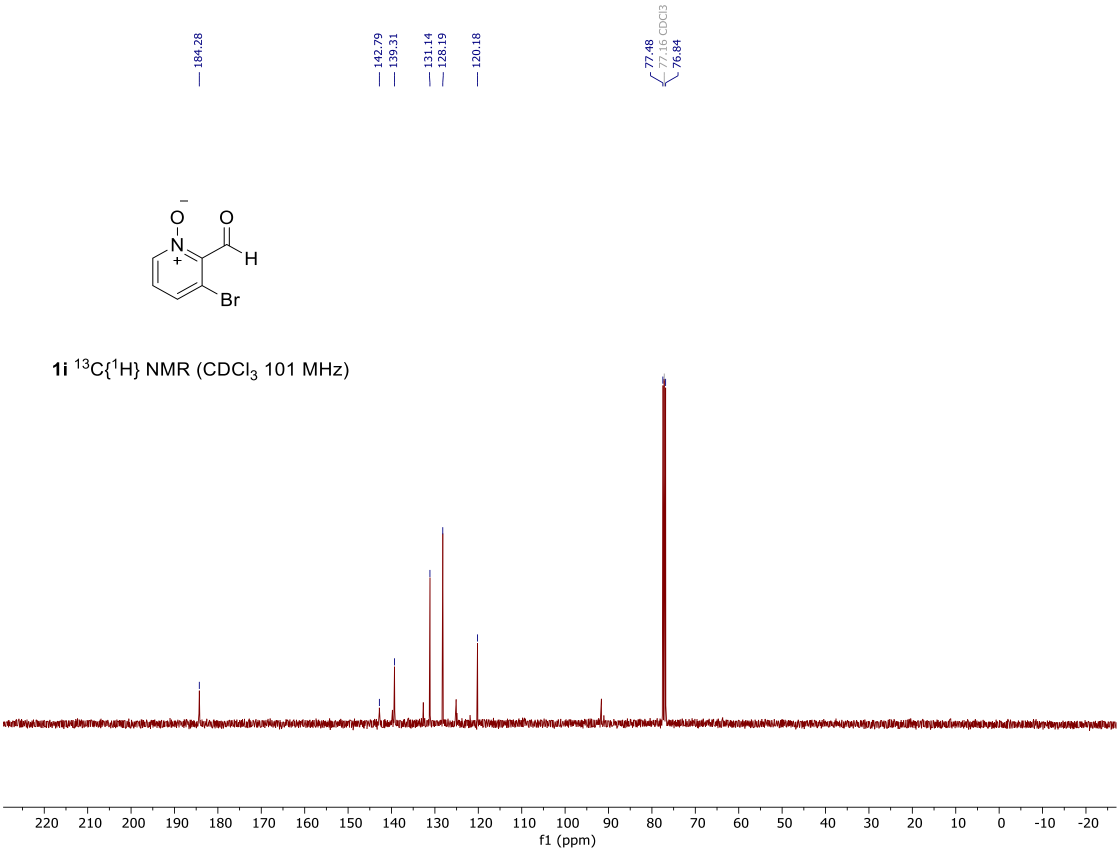
184.28
142.79
139.31
131.14
128.19
120.18
77.48
77.16 CDCl3
76.84
O
N
+
O
H
Br
1i 13C{1H} NMR (CDCl3 101 MHz)
220 210 200 190 180 170 160 150 140 130 120 110 100 90 80 70 60 50 40 30 20 10 0 -10 -20
f1 (ppm)

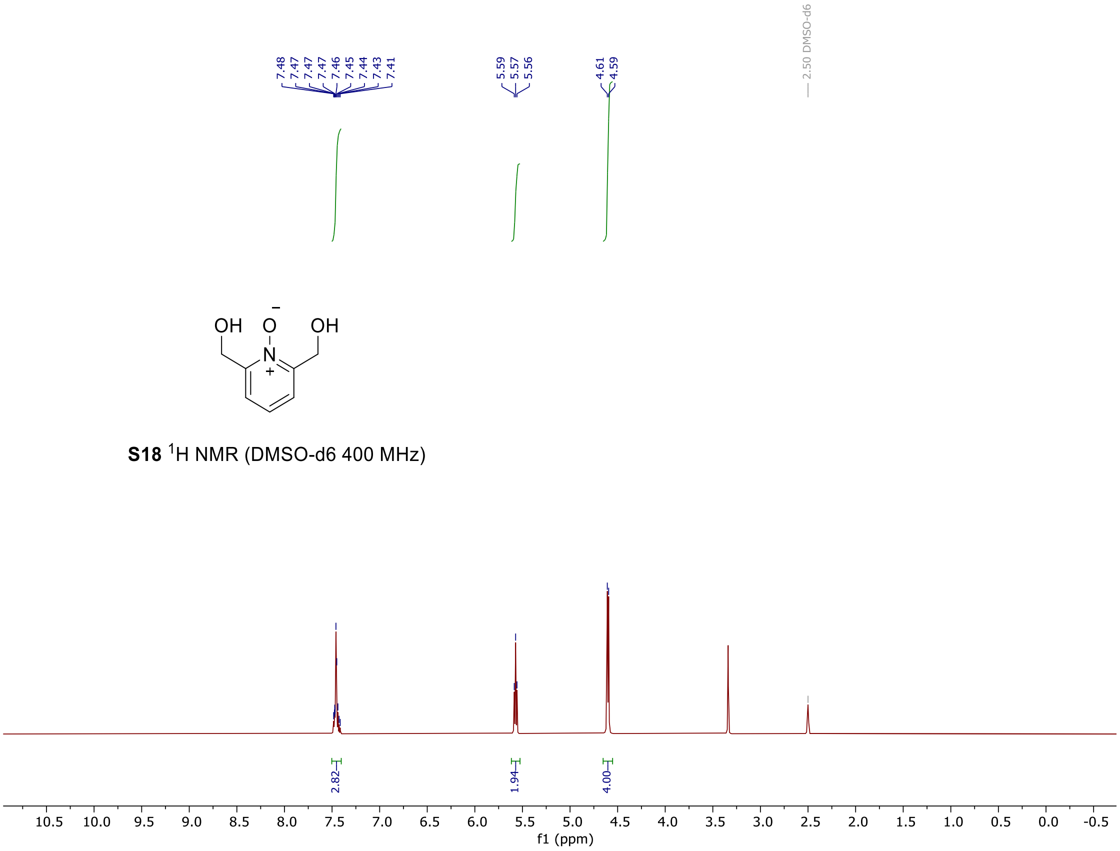

S18 ^{1}H NMR (DMSO-d6 400 MHz)

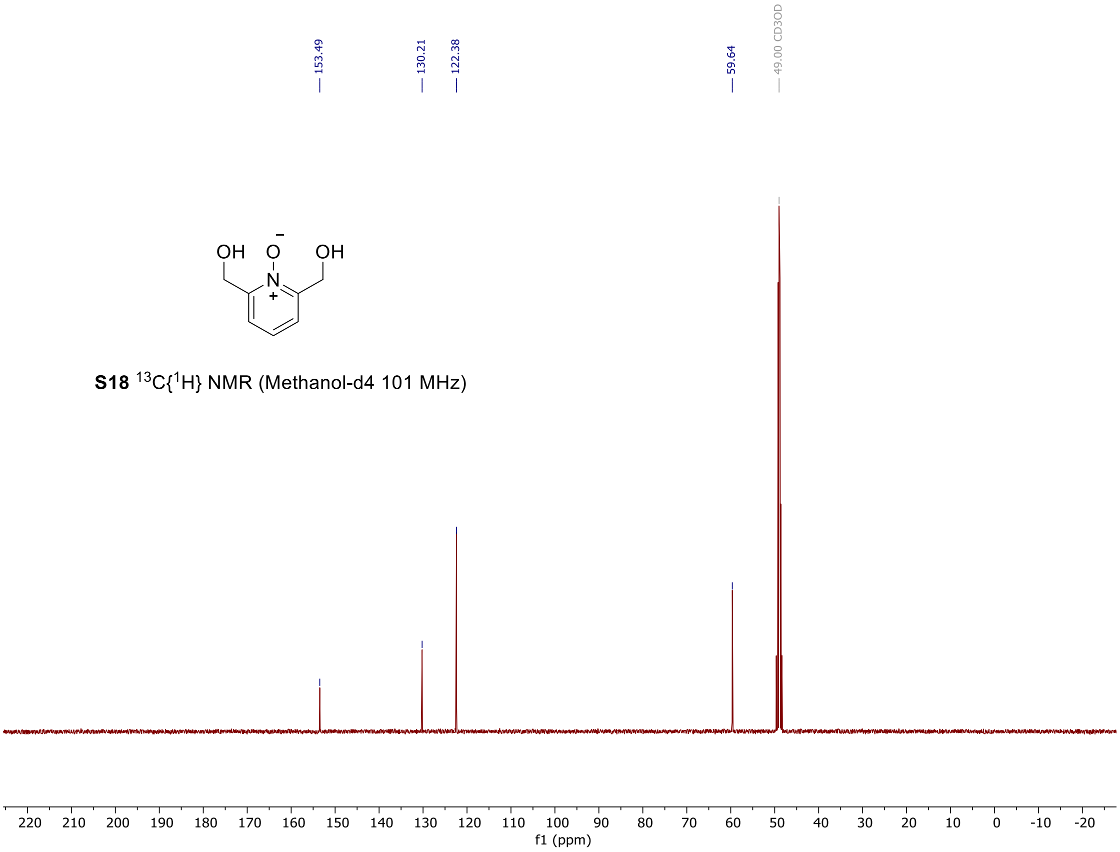

S18 $^{13}C\{^{1}H\}$ NMR (Methanol-d4 101 MHz)

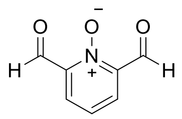

1j ^{1}H NMR (DMSO-d6 400 MHz)

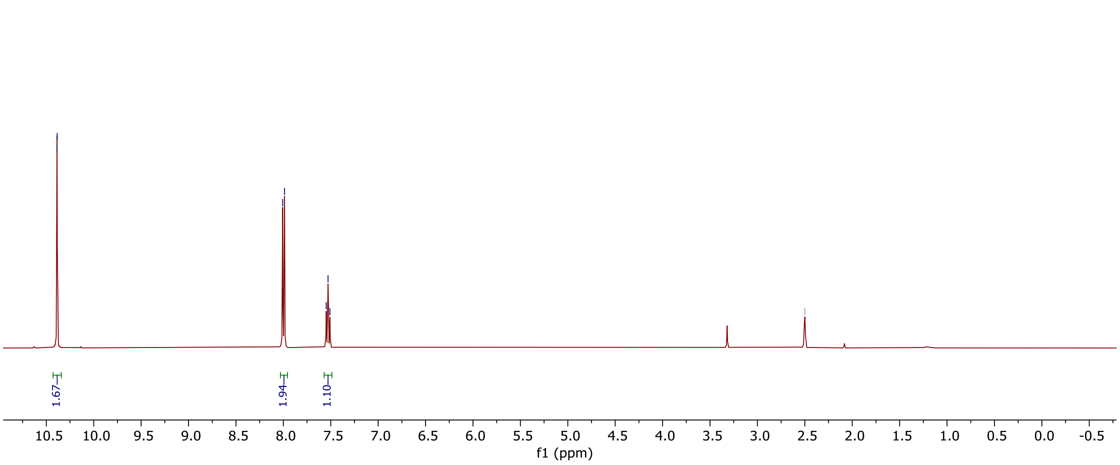

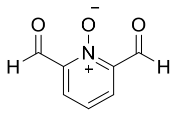

1j ^{13}C{^{1}H} NMR (DMSO-d6 101 MHz)

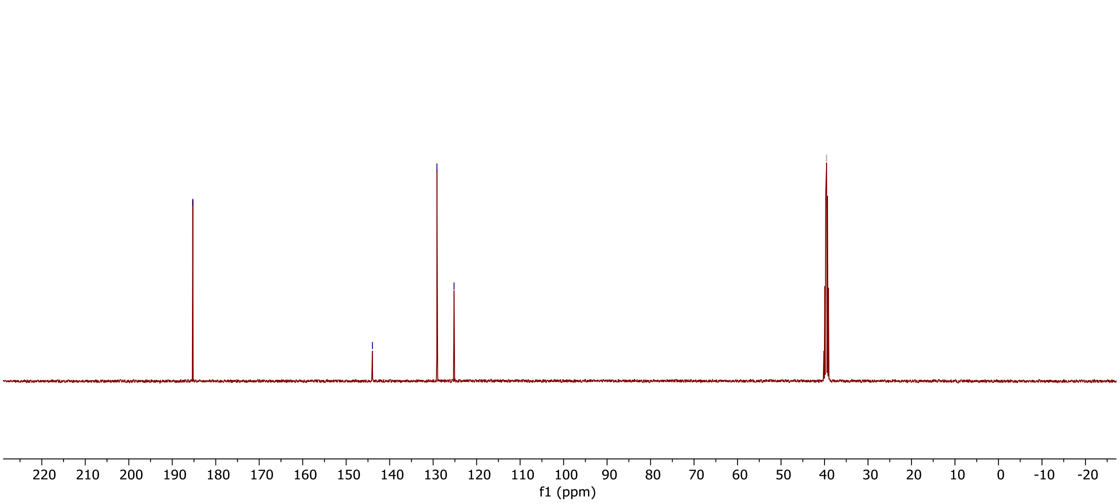

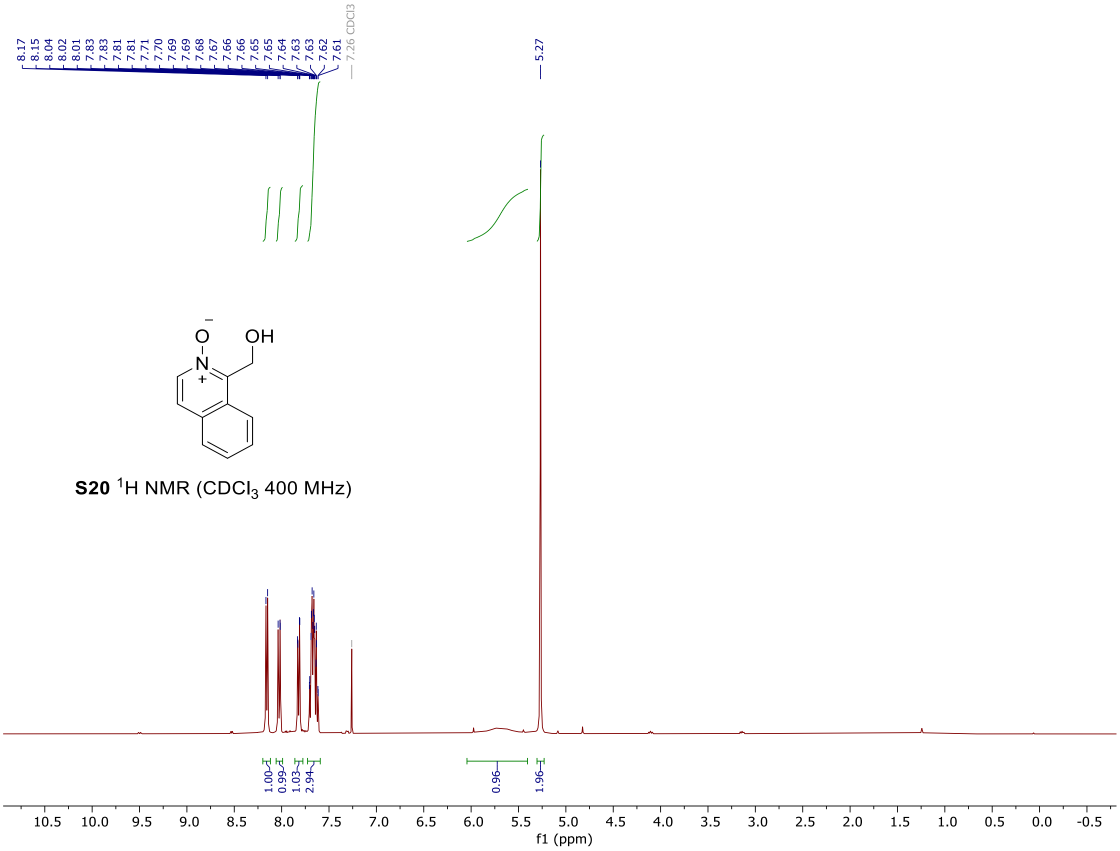

S20 ^{1}H NMR ($CDCl_3$ 400 MHz)

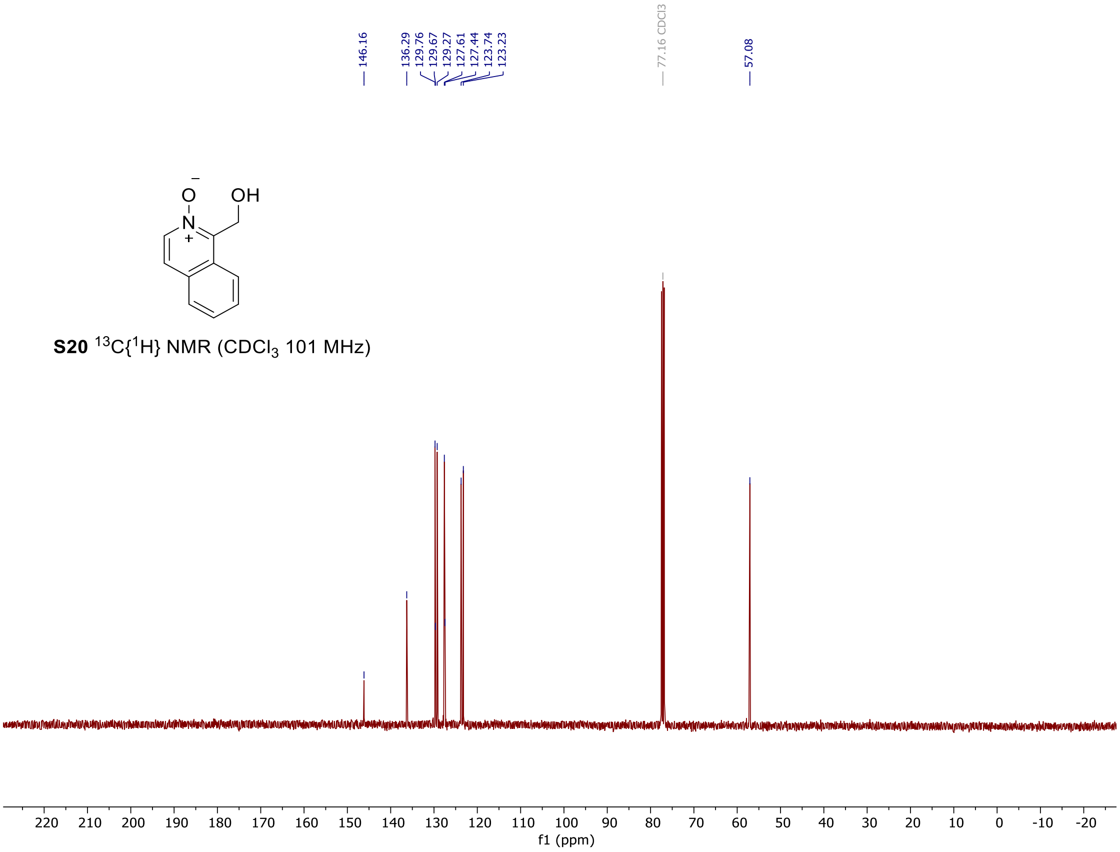

S20 ^{13}C{^{1}H} NMR ($CDCl_3$ 101 MHz)

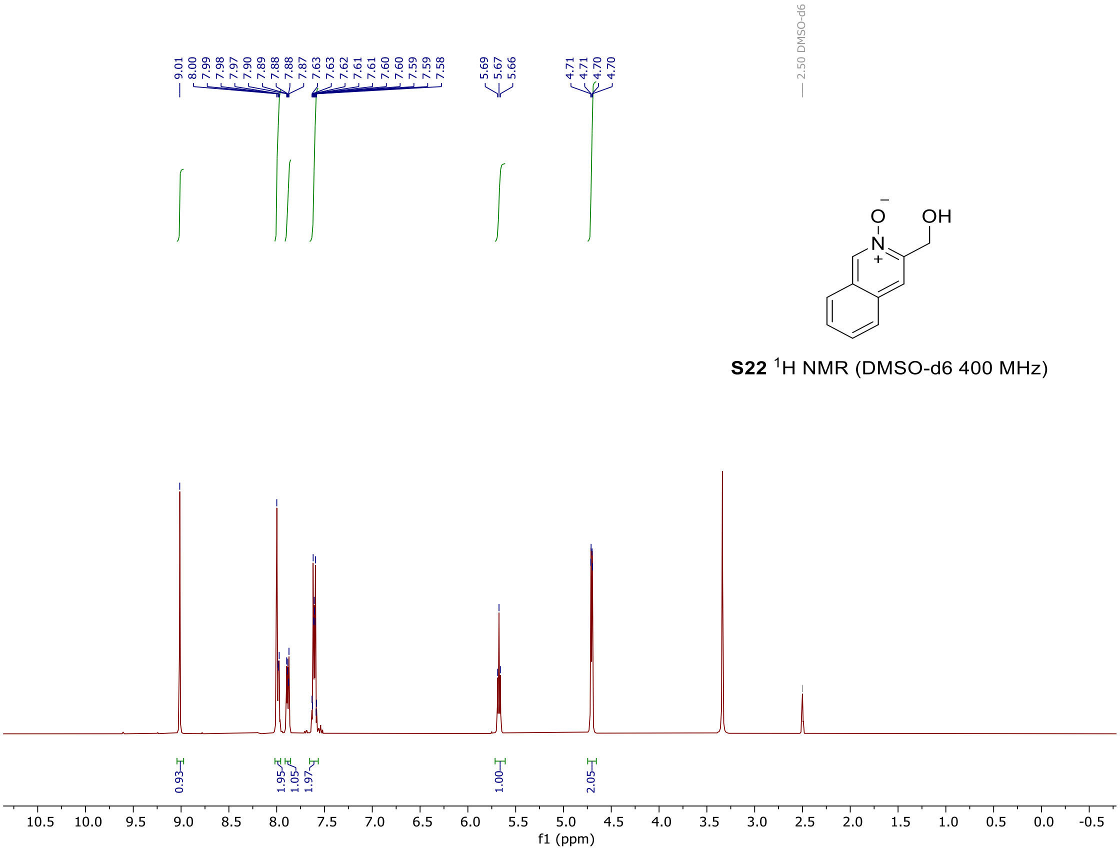

S22 ^{1}H NMR (DMSO-d6 400 MHz)

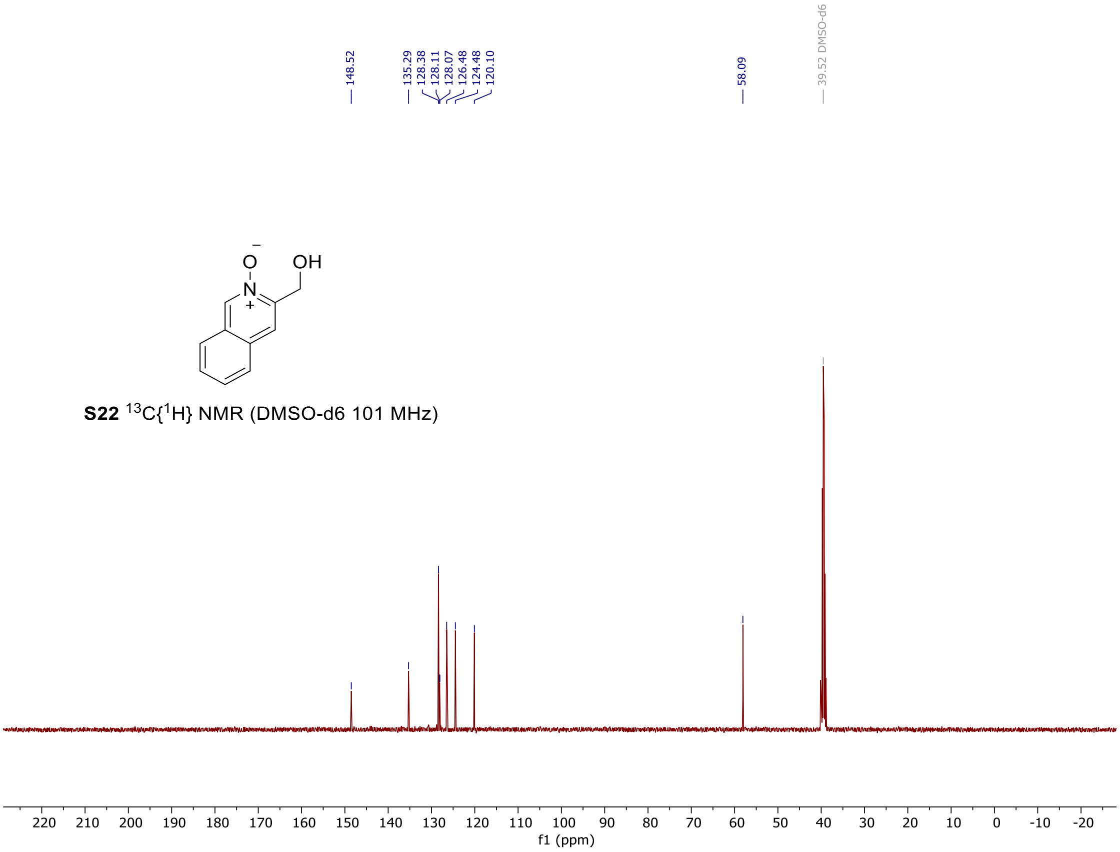

S22 $^{13}C\{^{1}H\}$ NMR (DMSO-d6 101 MHz)

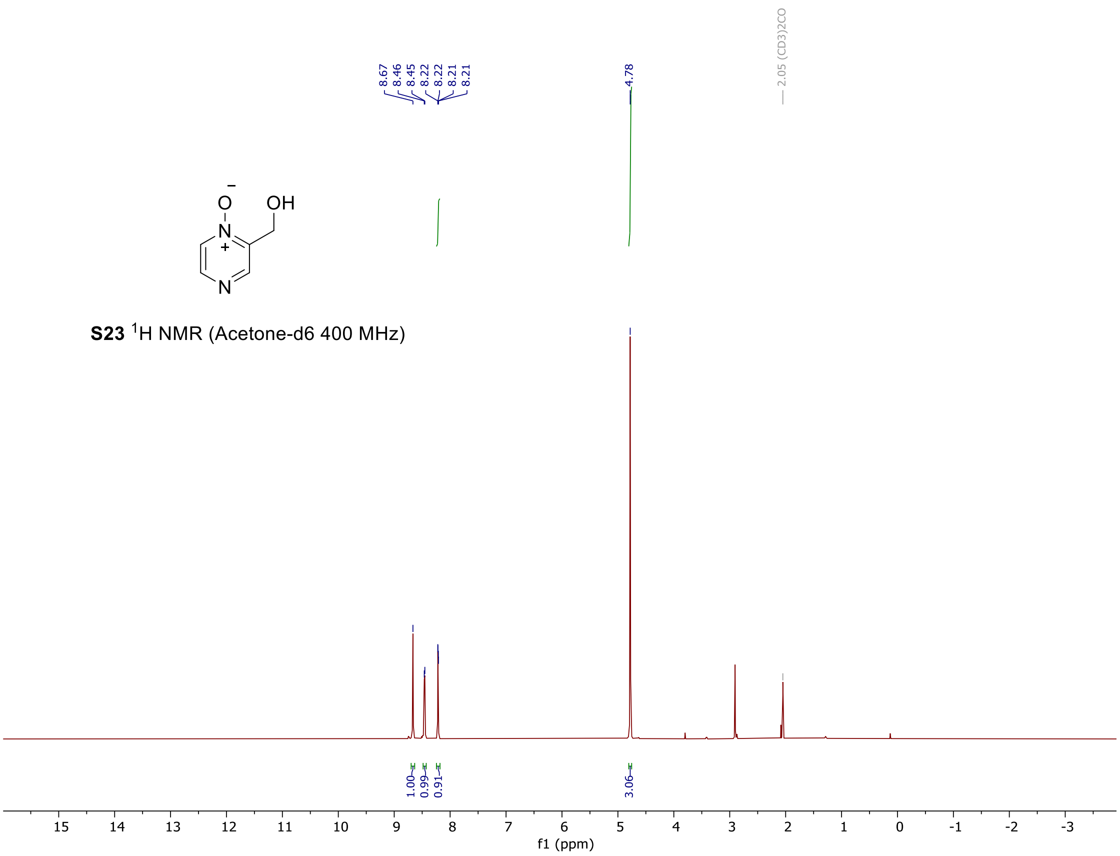

S23 ^{1}H NMR (Acetone-d6 400 MHz)

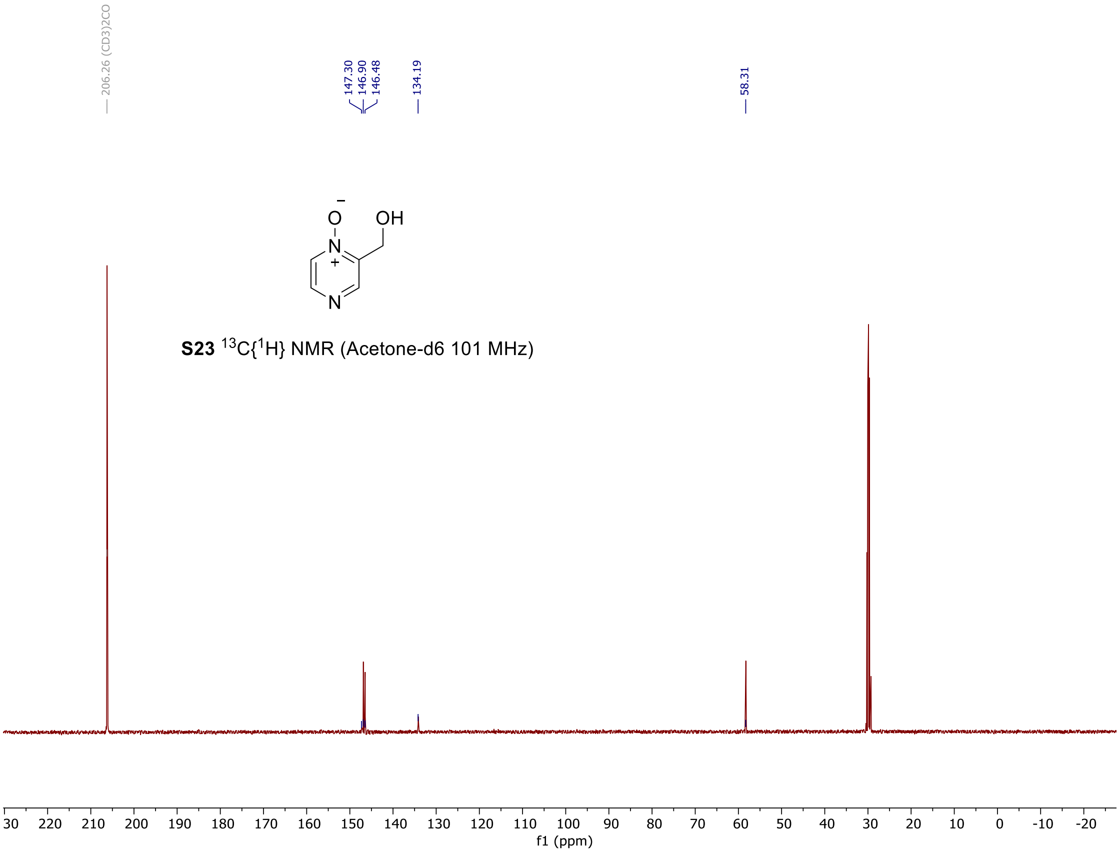

S23 $^{13}C\{^{1}H\}$ NMR (Acetone-d6 101 MHz)

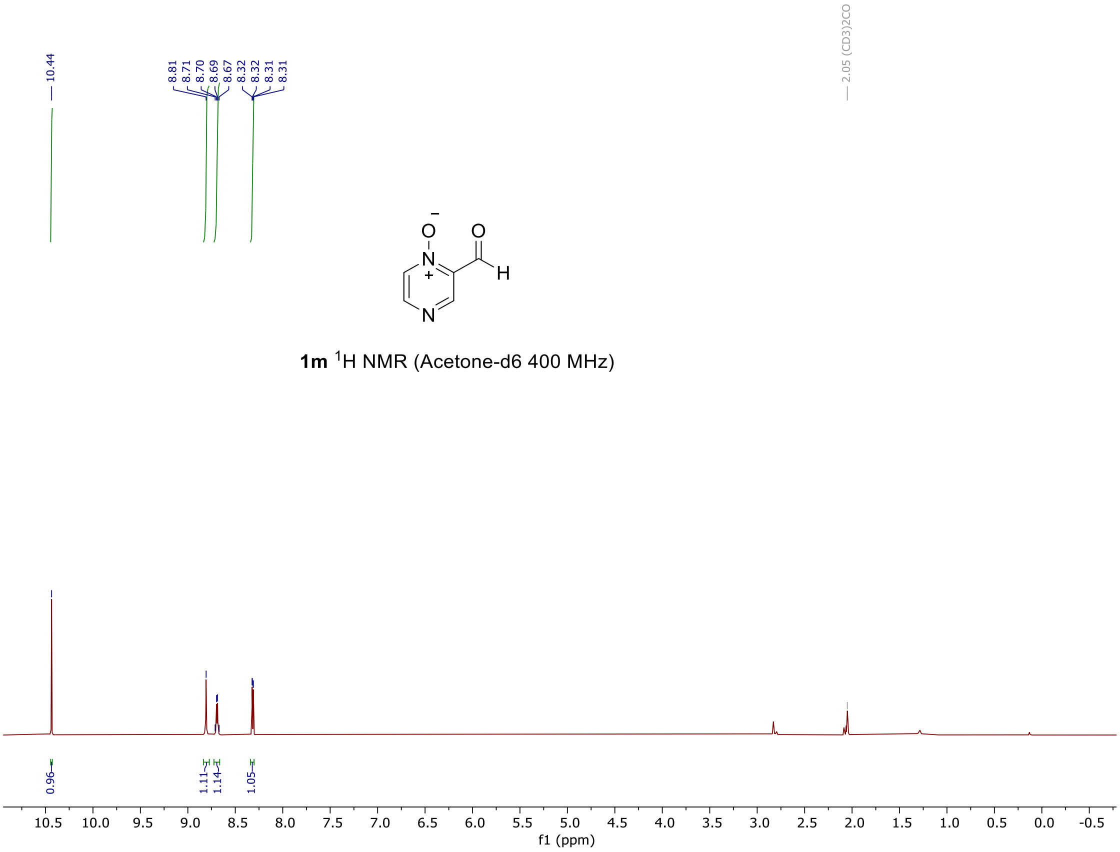

1m ^{1}H NMR (Acetone-d6 400 MHz)

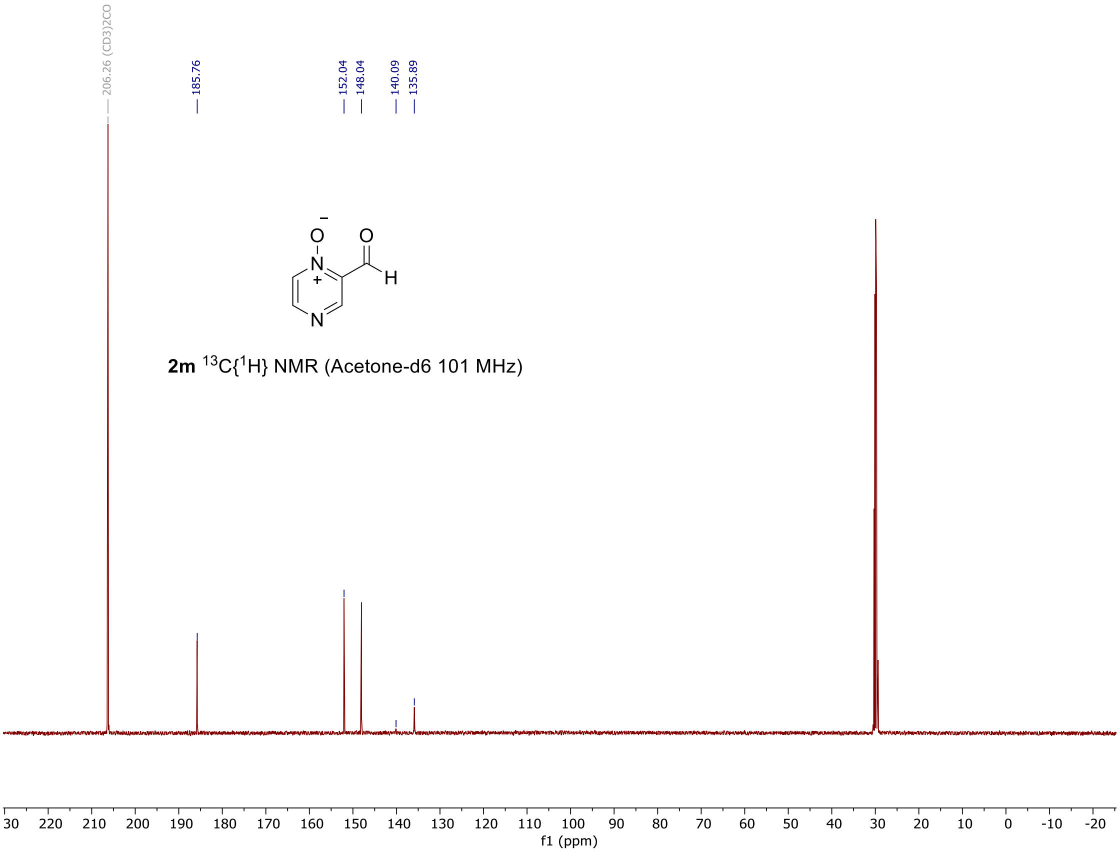

2m $^{13}C\{^{1}H\}$ NMR (Acetone-d6 101 MHz)

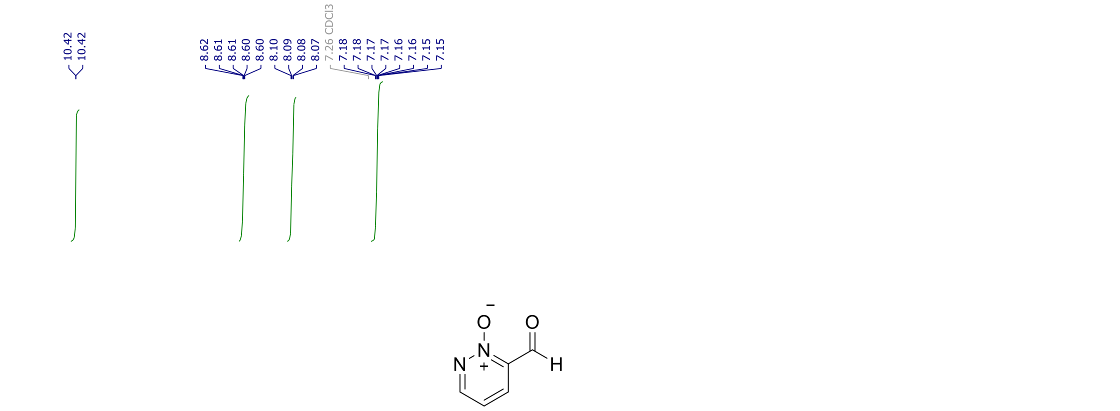

1n ^{1}H NMR ($CDCl_3$ 400 MHz)

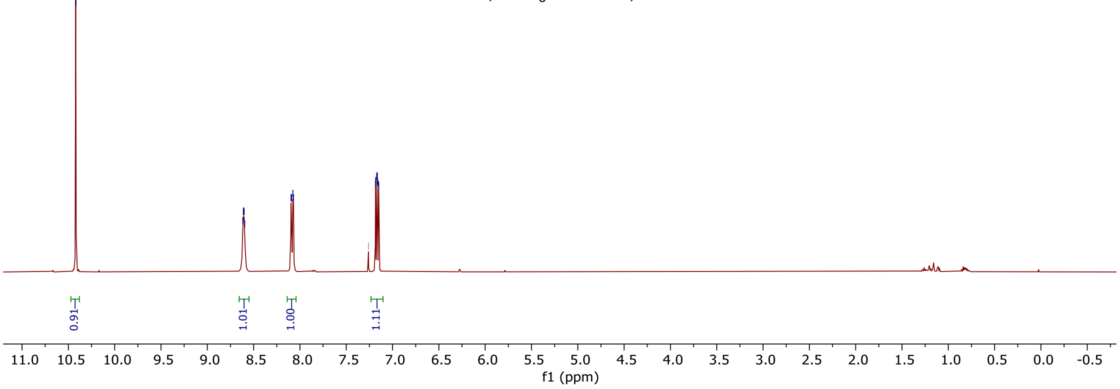

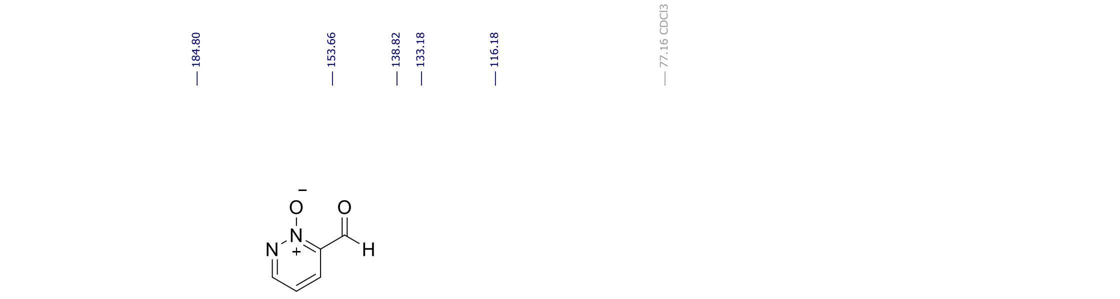

1n $^{13}C\{^{1}H\}$ NMR ($CDCl_3$ 101 MHz)

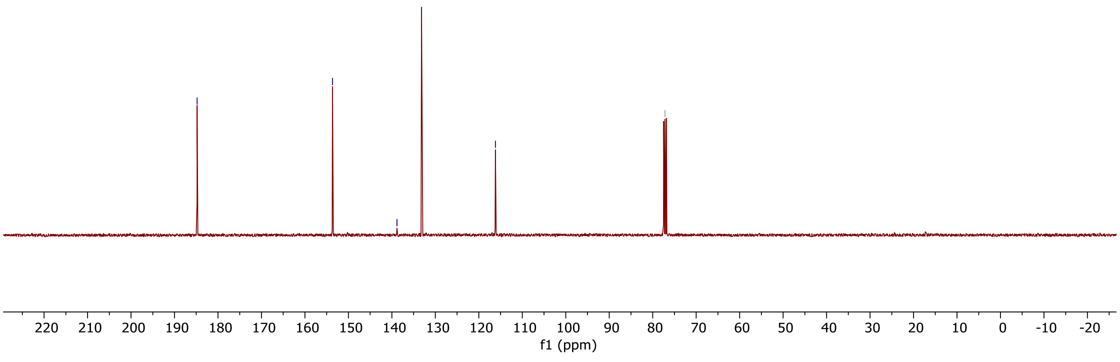

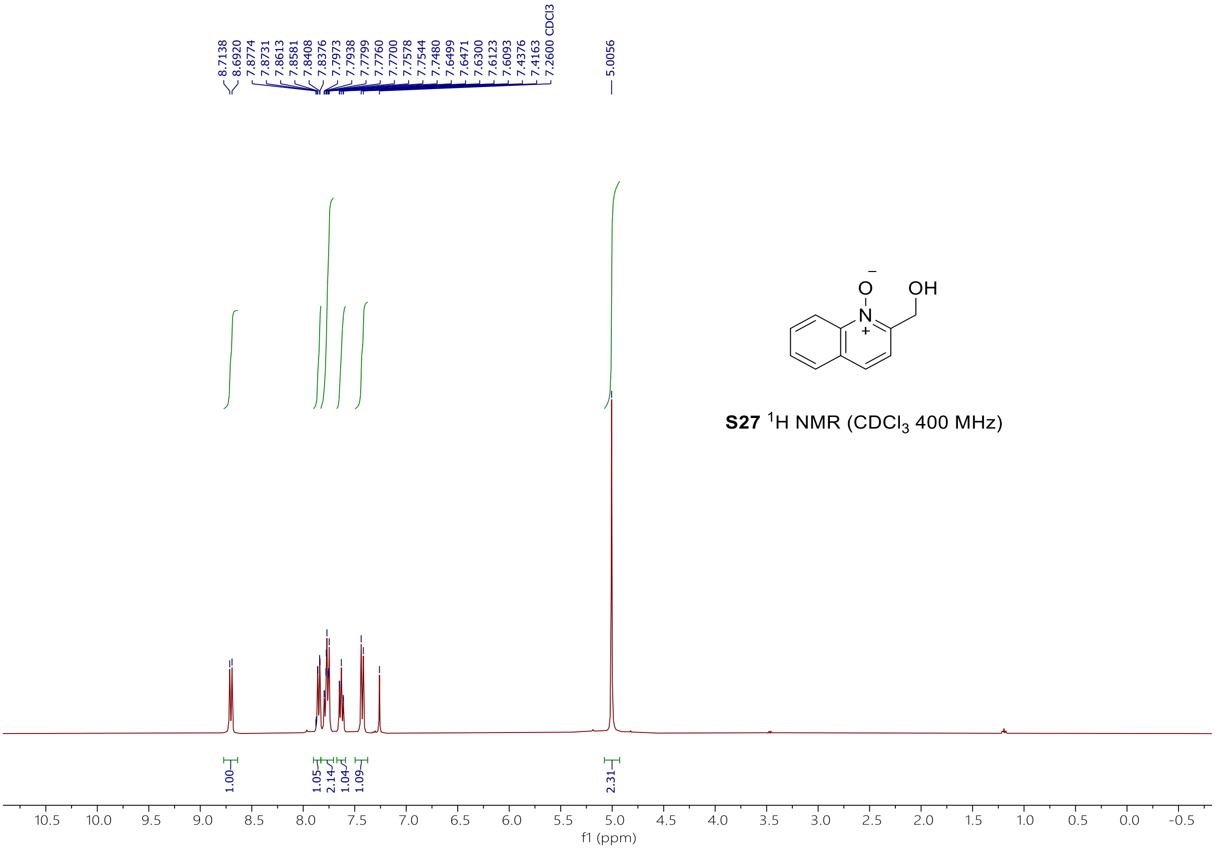

S27 ^{1}H NMR ($CDCl_3$ 400 MHz)

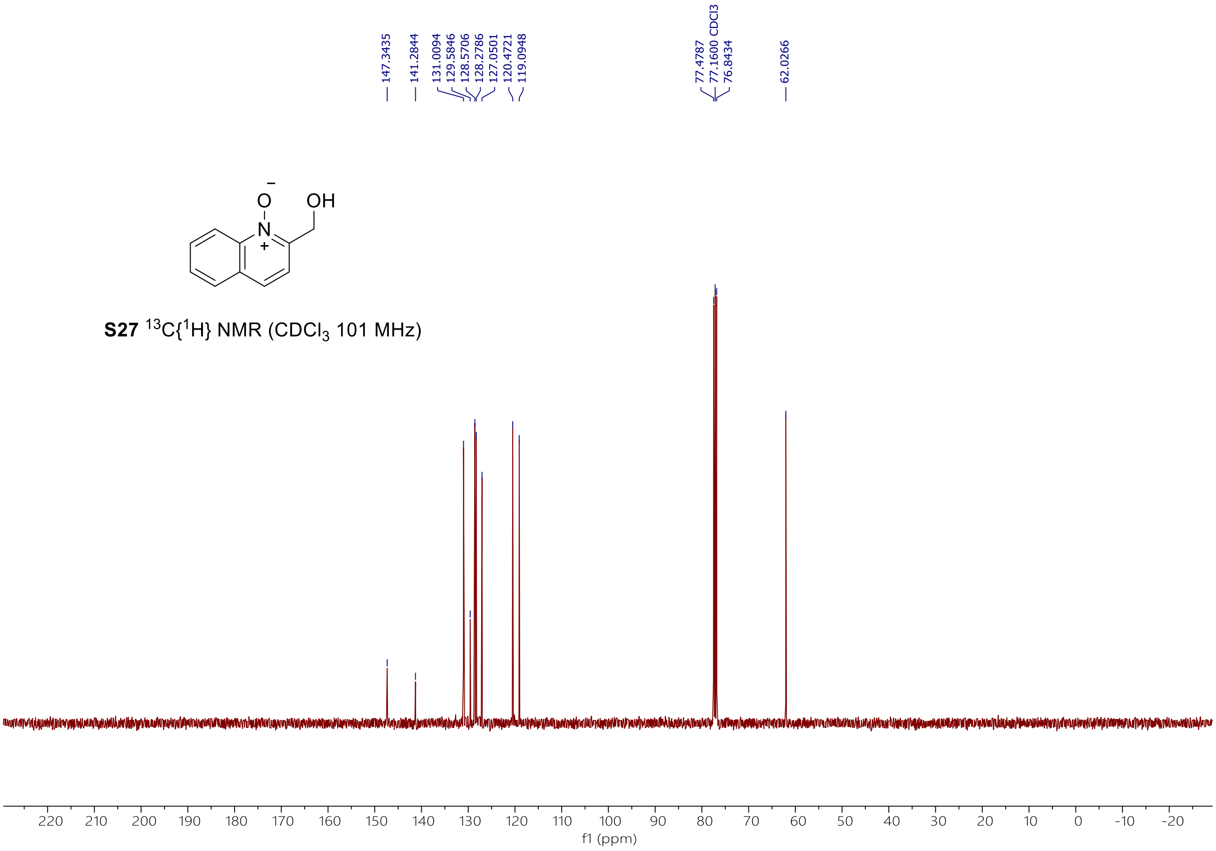

S27 ^{13}C{^{1}H} NMR ($CDCl_3$ 101 MHz)

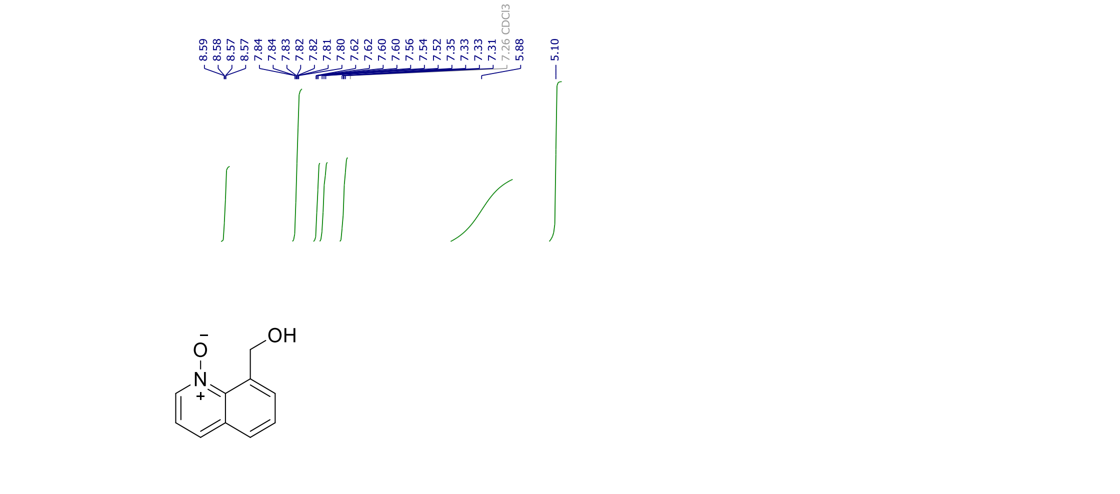

S29 ^{1}H NMR ($CDCl_3$ 400 MHz)

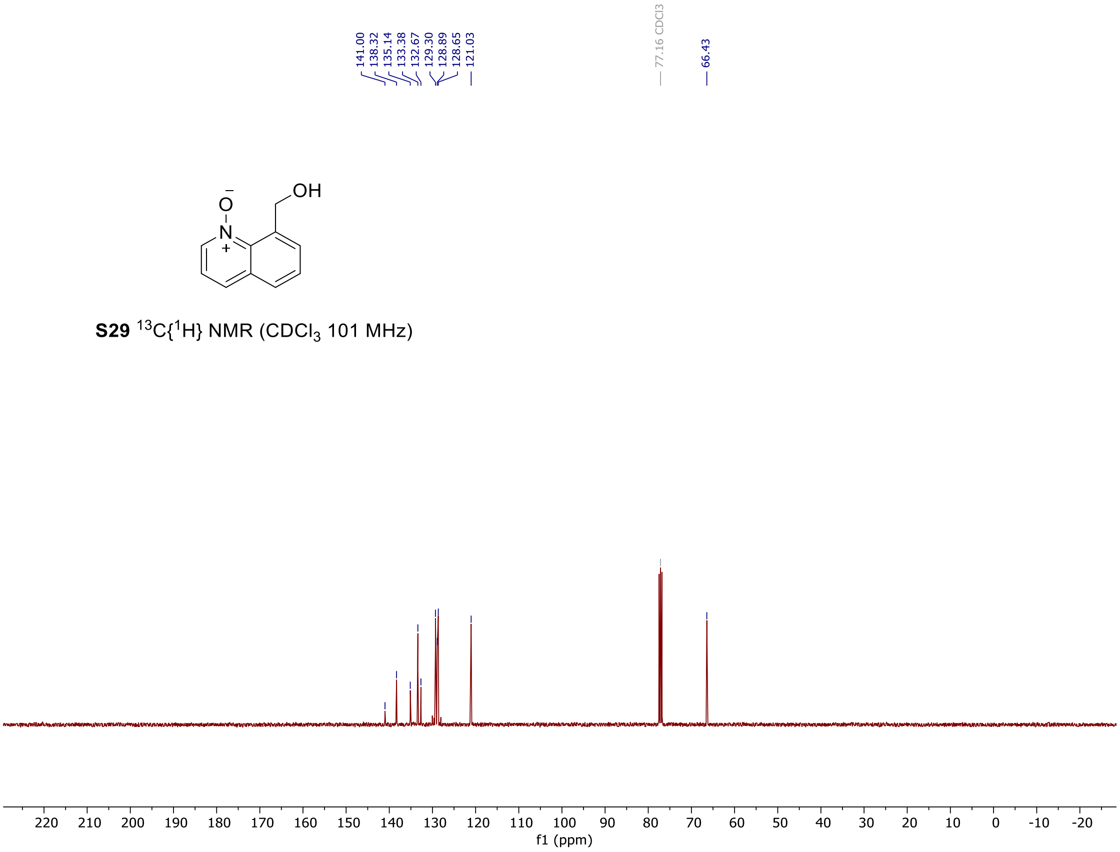

S29 ^{13}C{^{1}H} NMR ($CDCl_3$ 101 MHz)

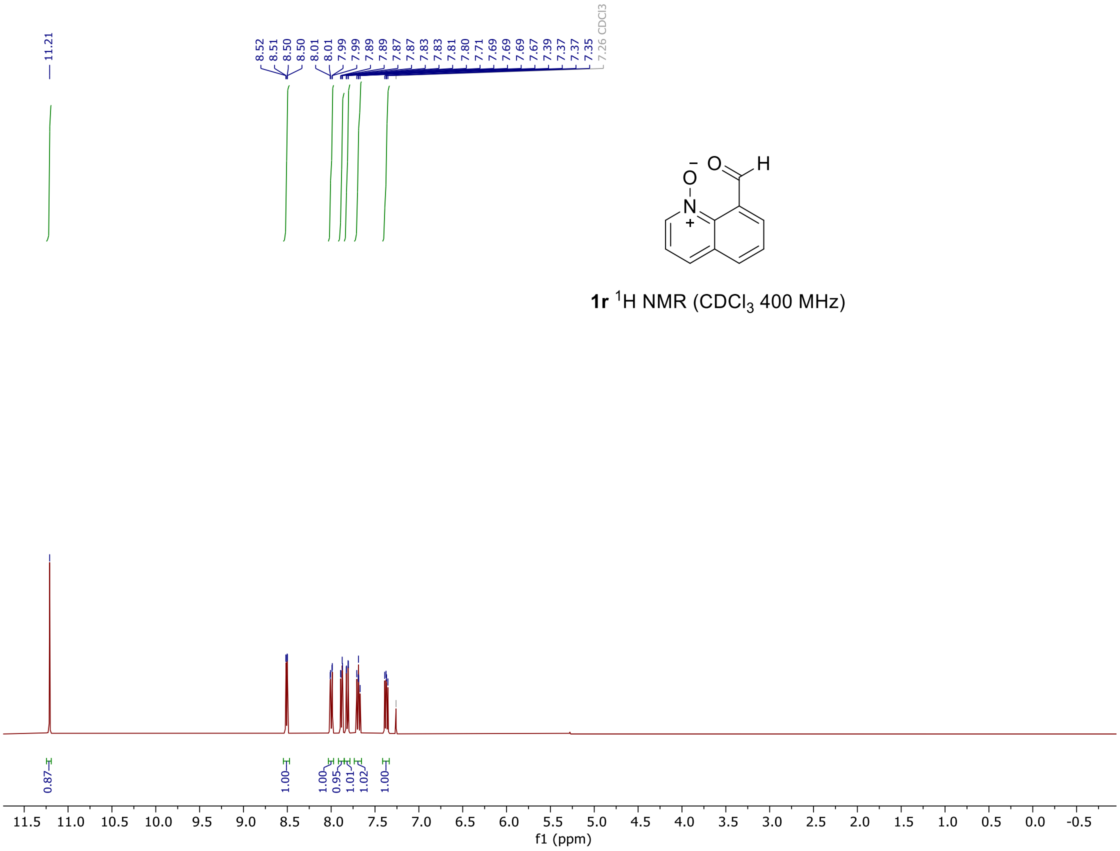

1r ^{1}H NMR ($CDCl_3$ 400 MHz)

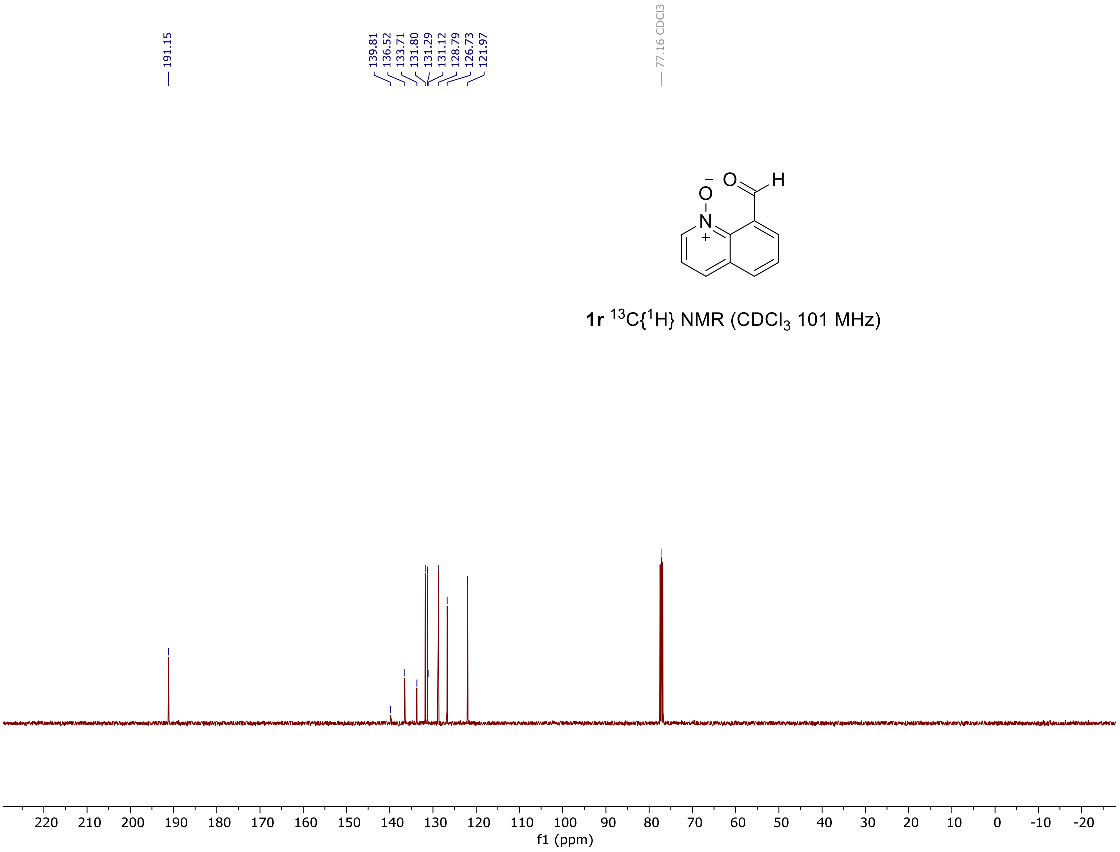

1r $^{13}C\{^{1}H\}$ NMR ($CDCl_3$ 101 MHz)

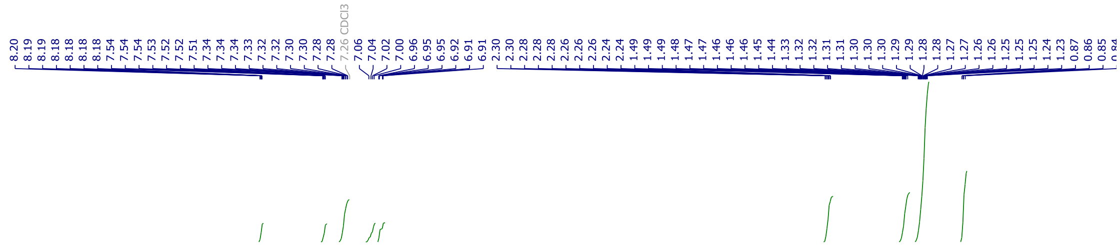

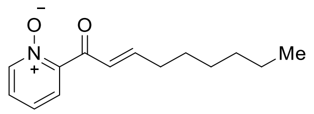

2a ^{1}H NMR ($CDCl_3$ 400 MHz)

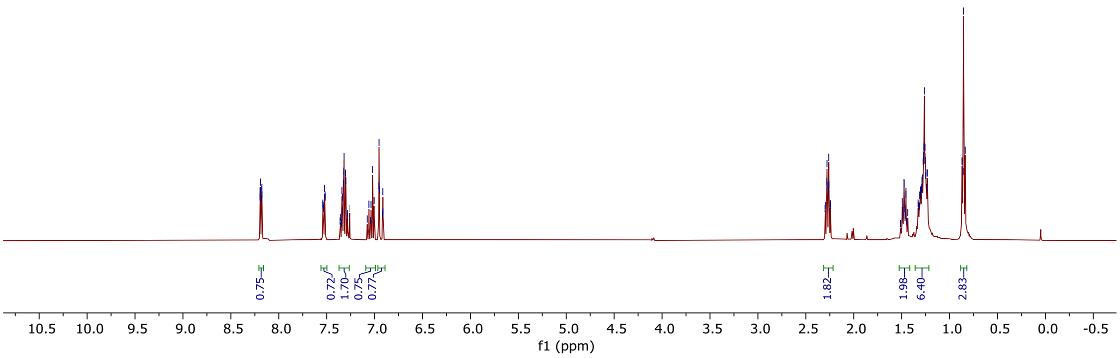

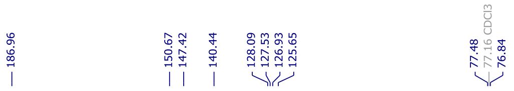

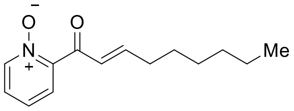

2a ^{13}C{^{1}H} NMR ($CDCl_3$ 101 MHz)

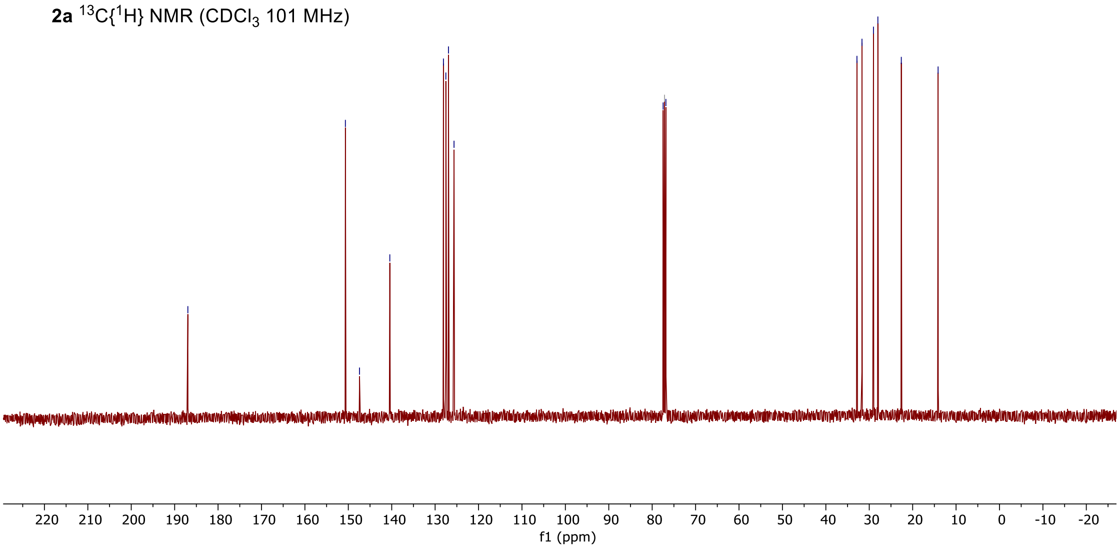

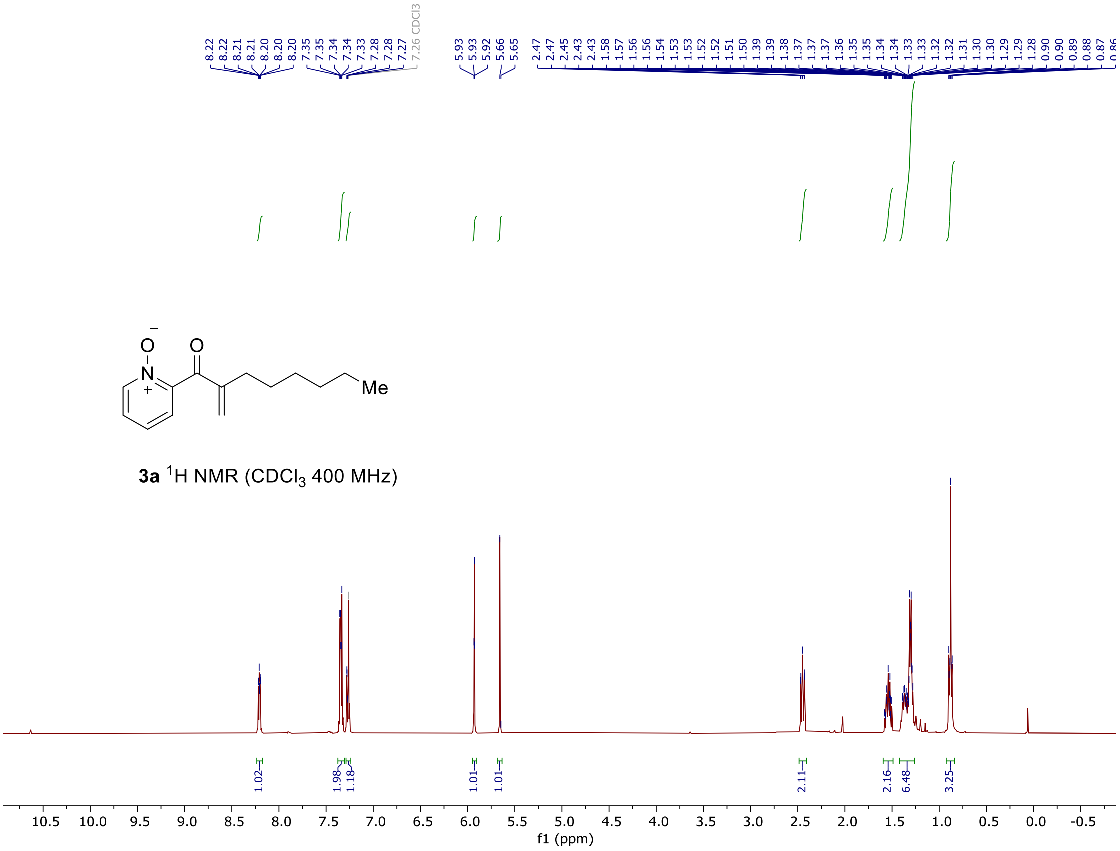
8.22 8.22 8.21 8.21 8.20 8.20 8.20 7.35 7.35 7.34 7.34 7.33 7.28 7.28 7.27 7.26 CDCl3
5.93 5.93 5.92 5.66 5.65
2.47 2.47 2.45 2.43 2.43 1.58 1.57 1.56 1.56 1.54 1.53 1.53 1.52 1.52 1.51 1.50 1.39 1.39 1.38 1.37 1.37 1.37 1.36 1.35 1.35 1.34 1.33 1.33 1.32 1.32 1.31 1.30 1.30 1.29 1.28 0.90 0.90 0.89 0.88 0.87
Me
3a ¹H NMR (CDCl₃ 400 MHz)
1.02 1.98 1.18 1.01 1.01 2.11 2.16 6.48 3.25
f1 (ppm)

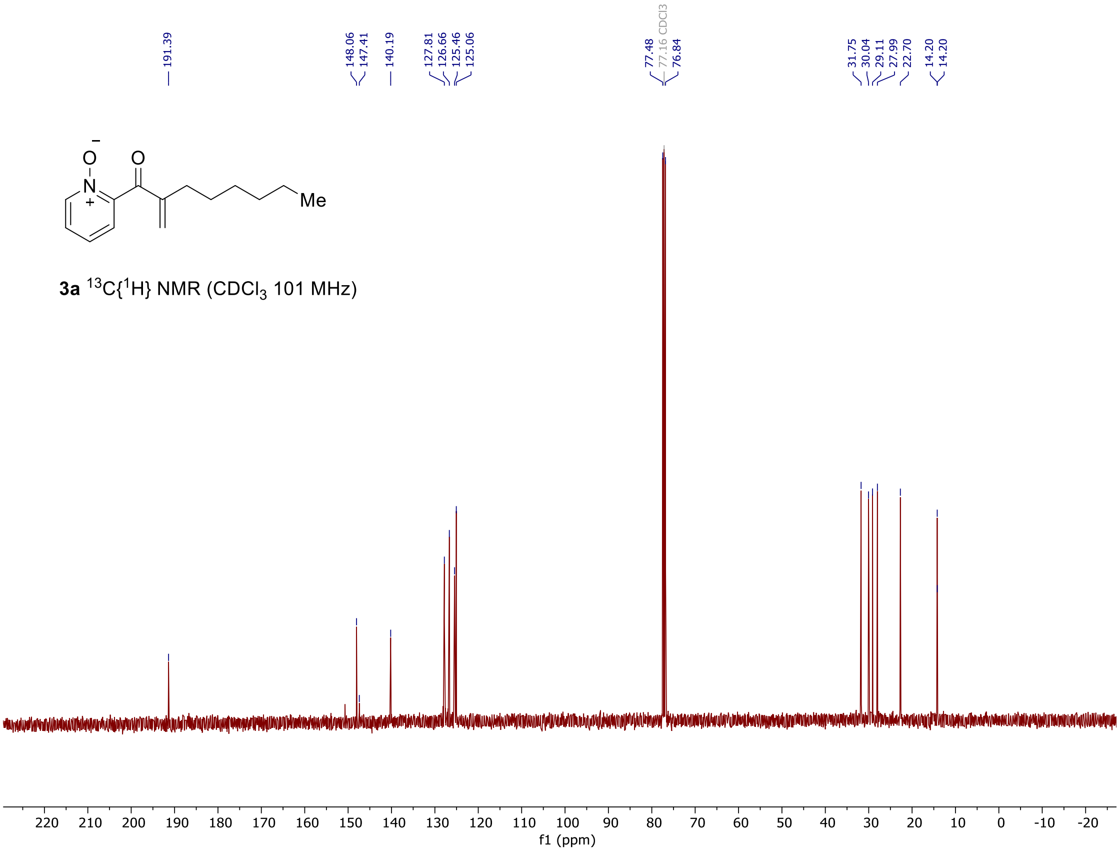
191.39
148.06 147.41 140.19 127.81 126.66 125.46 125.06
77.48 77.16 CDCl3 76.84
31.75 30.04 29.11 27.99 22.70 14.20 14.20
Me
3a ¹³C{¹H} NMR (CDCl₃ 101 MHz)
f1 (ppm)

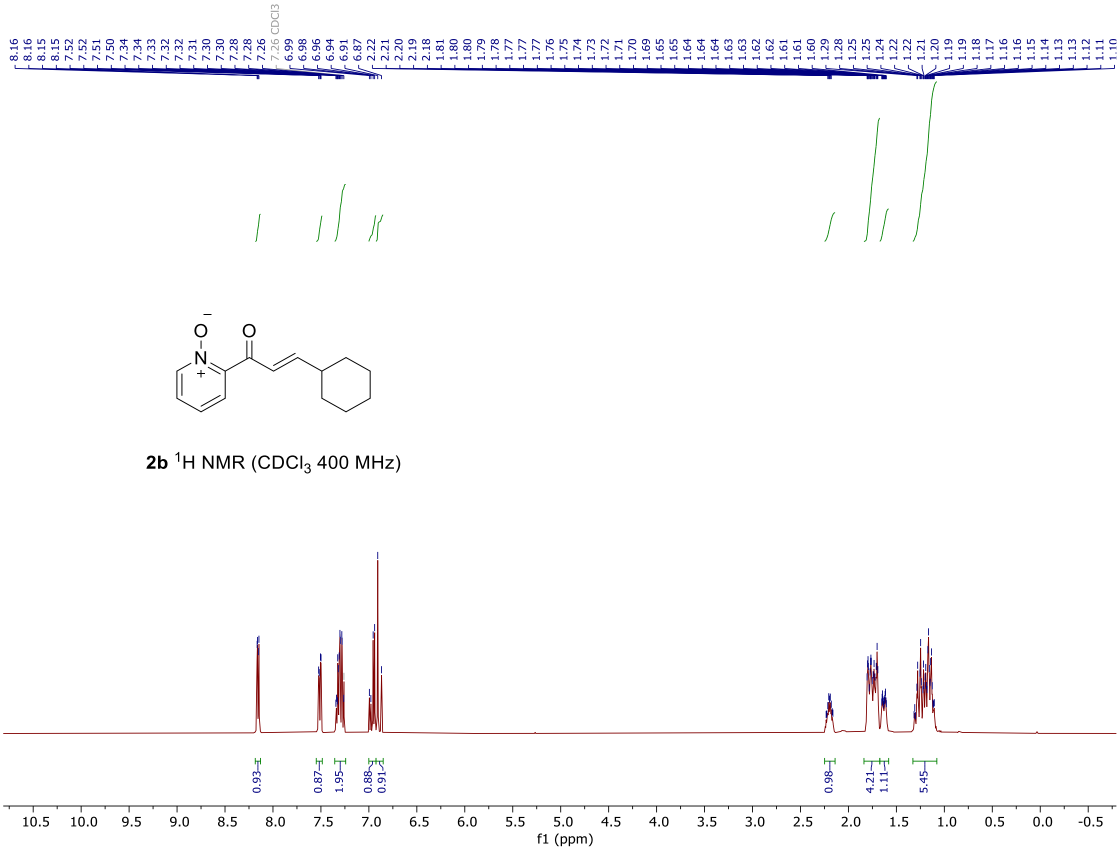

2b ^{1}H NMR ($CDCl_3$ 400 MHz)

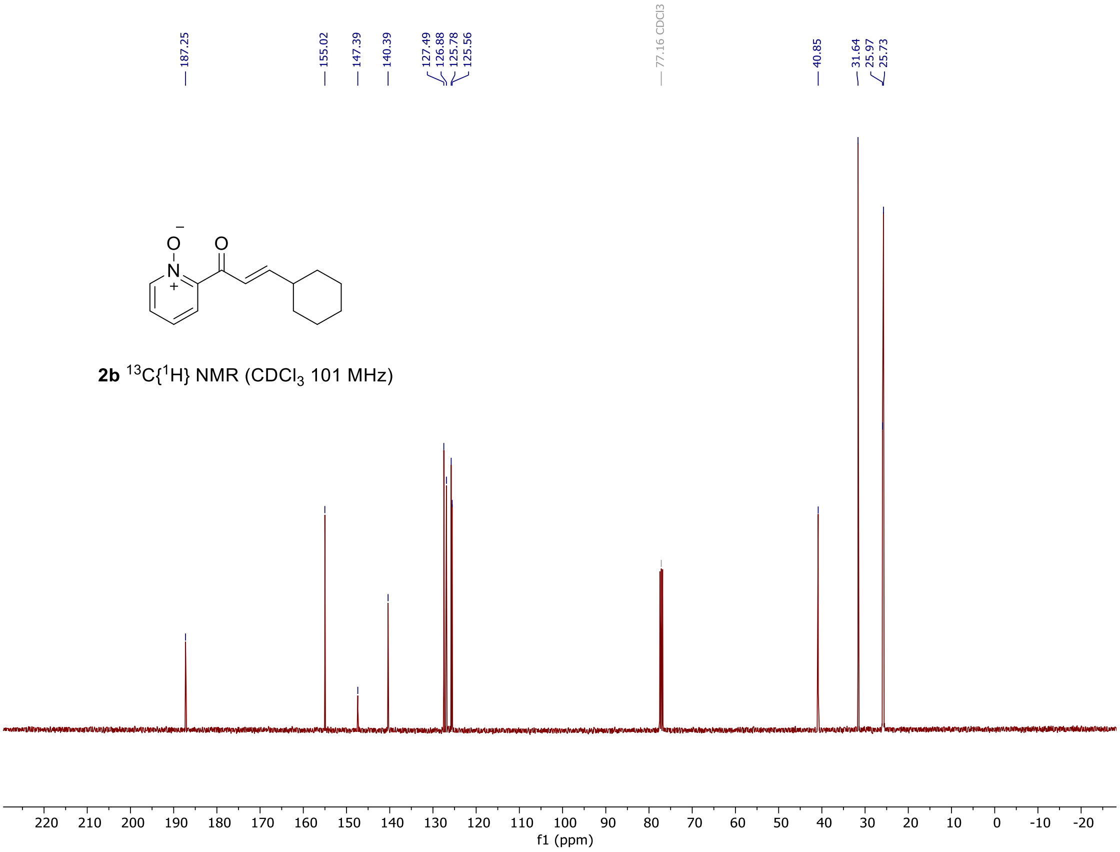

2b $^{13}C\{^{1}H\}$ NMR ($CDCl_3$ 101 MHz)

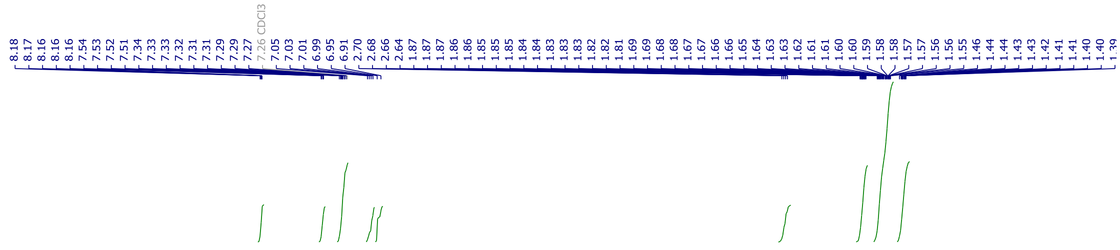

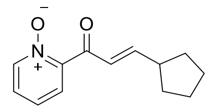

2c ^{1}H NMR ($CDCl_3$ 400 MHz)

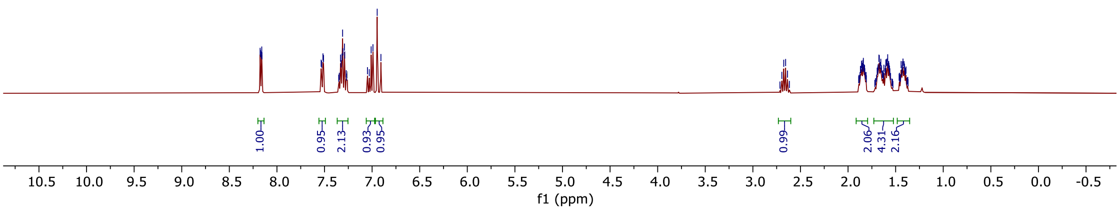

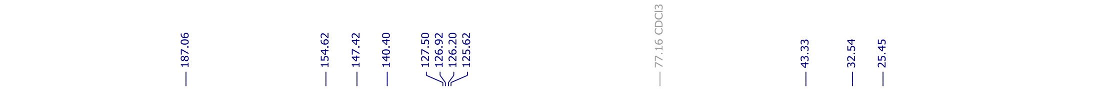

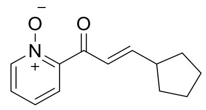

2c $^{13}C\{^{1}H\}$ NMR ($CDCl_3$ 101 MHz)

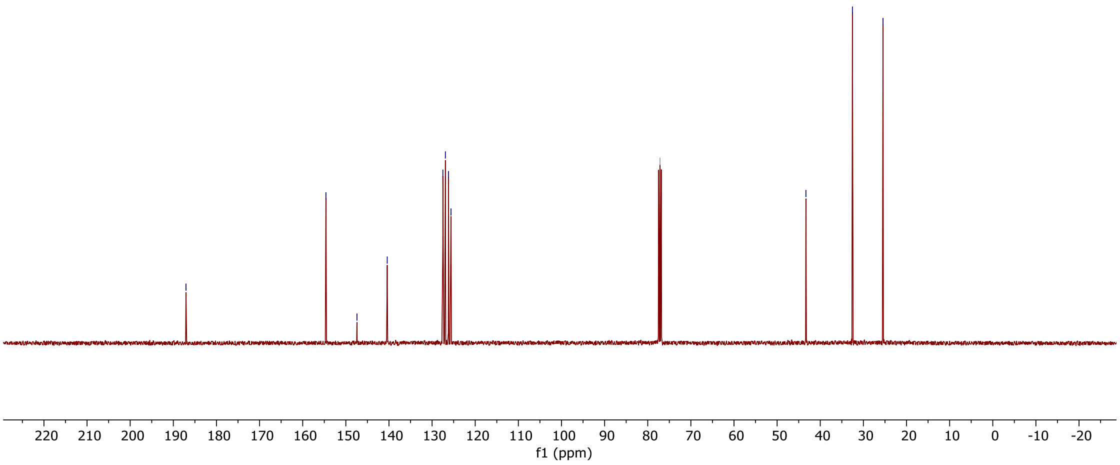

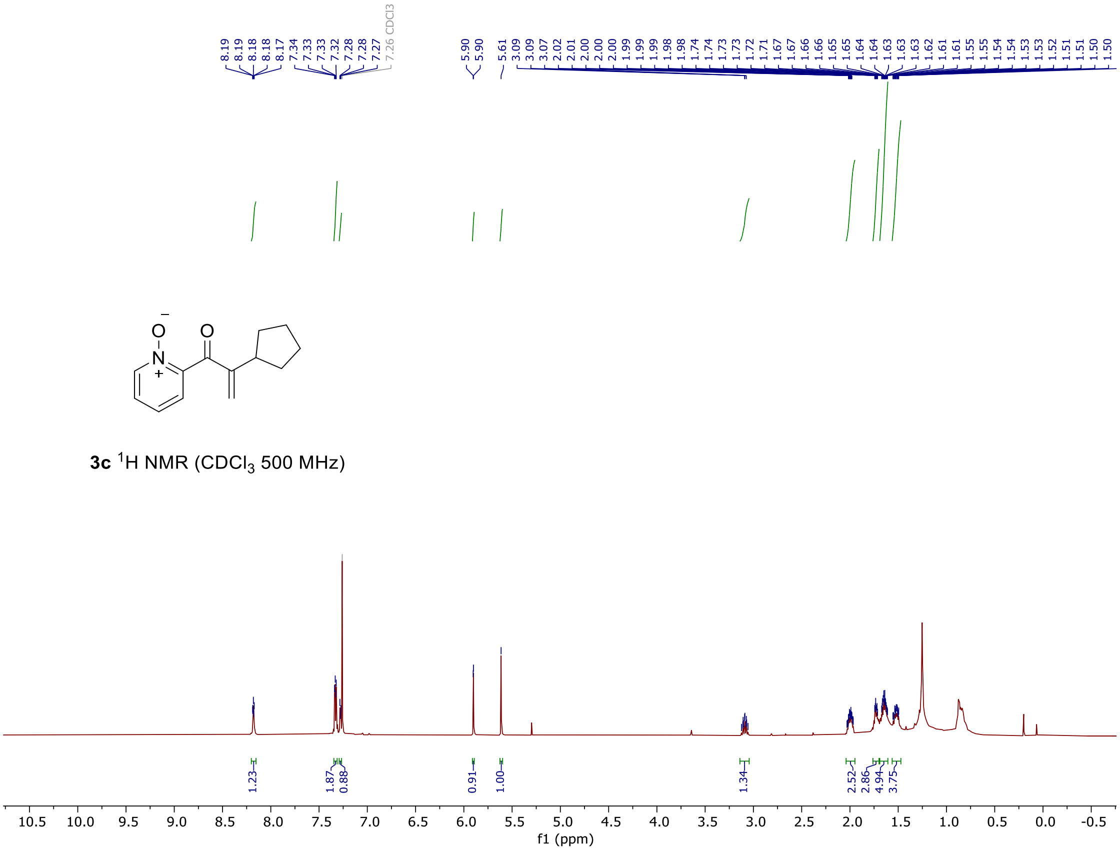

3c ^{1}H NMR ($CDCl_3$ 500 MHz)

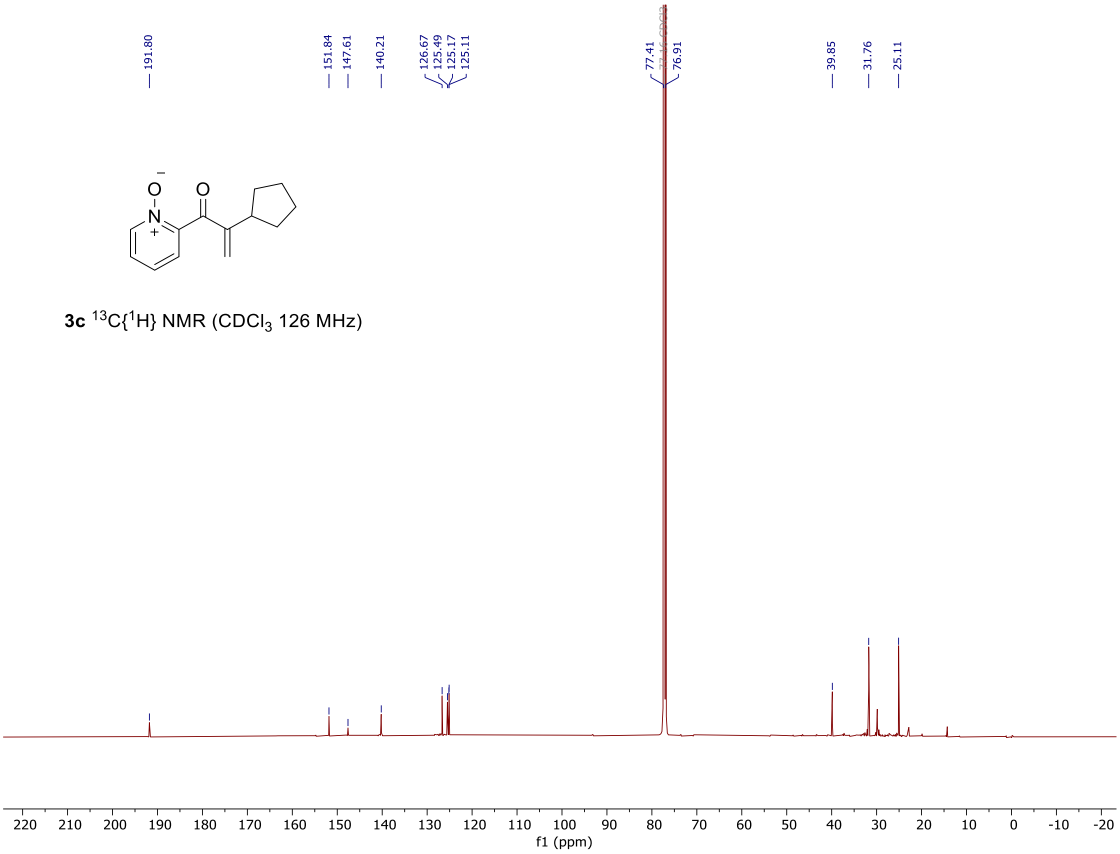

3c $^{13}C\{^{1}H\}$ NMR ($CDCl_3$ 126 MHz)

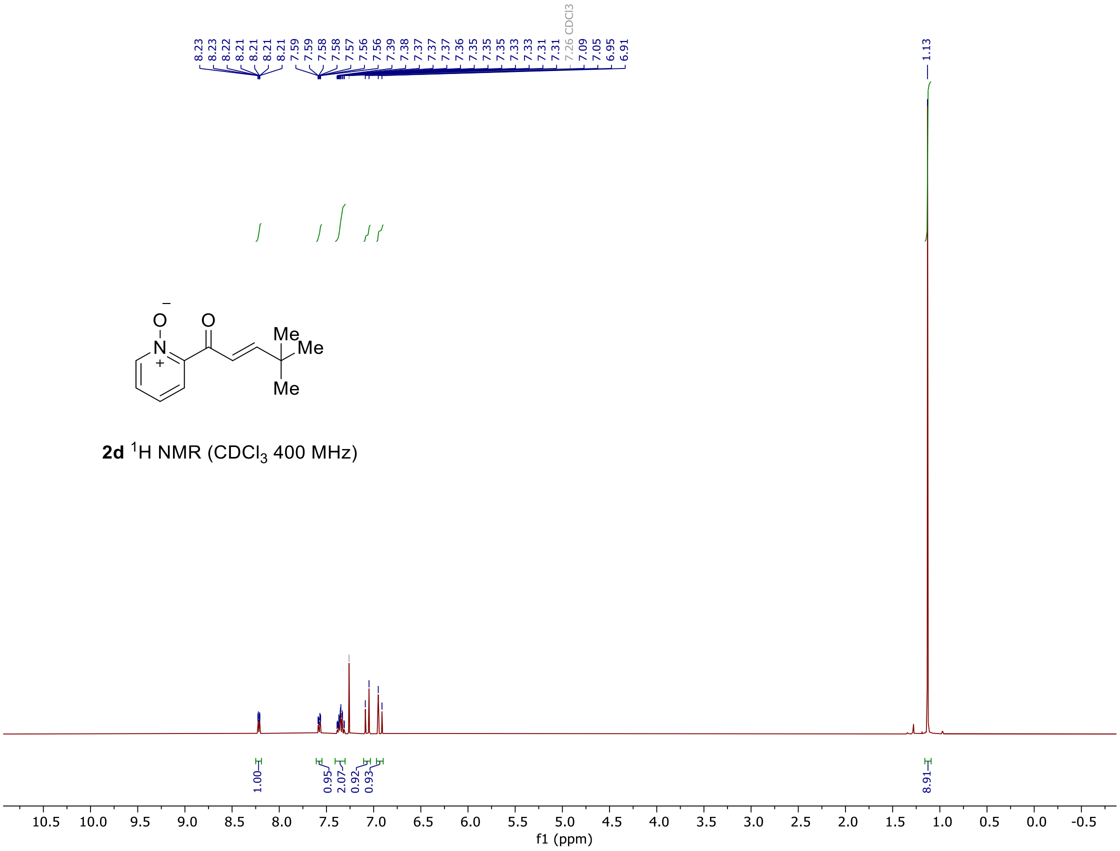

2d ^{1}H NMR ($CDCl_3$ 400 MHz)

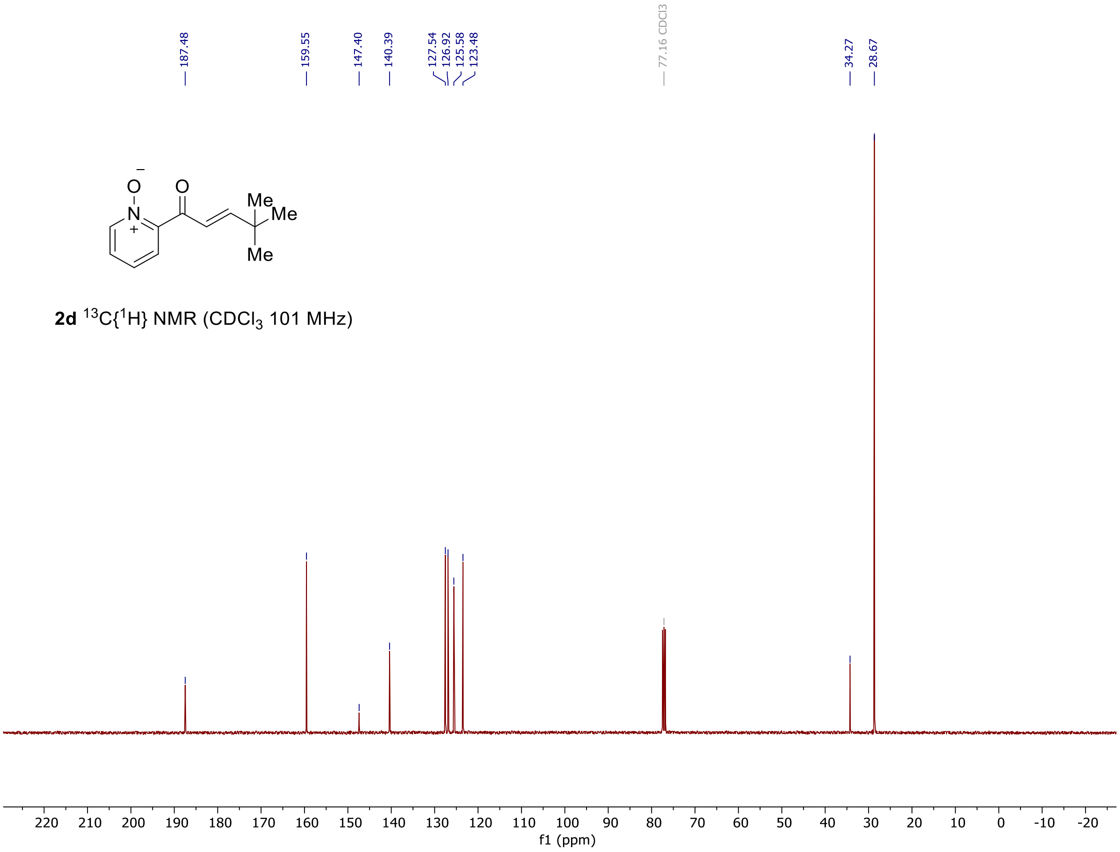

2d $^{13}C\{^1H\}$ NMR ($CDCl_3$ 101 MHz)

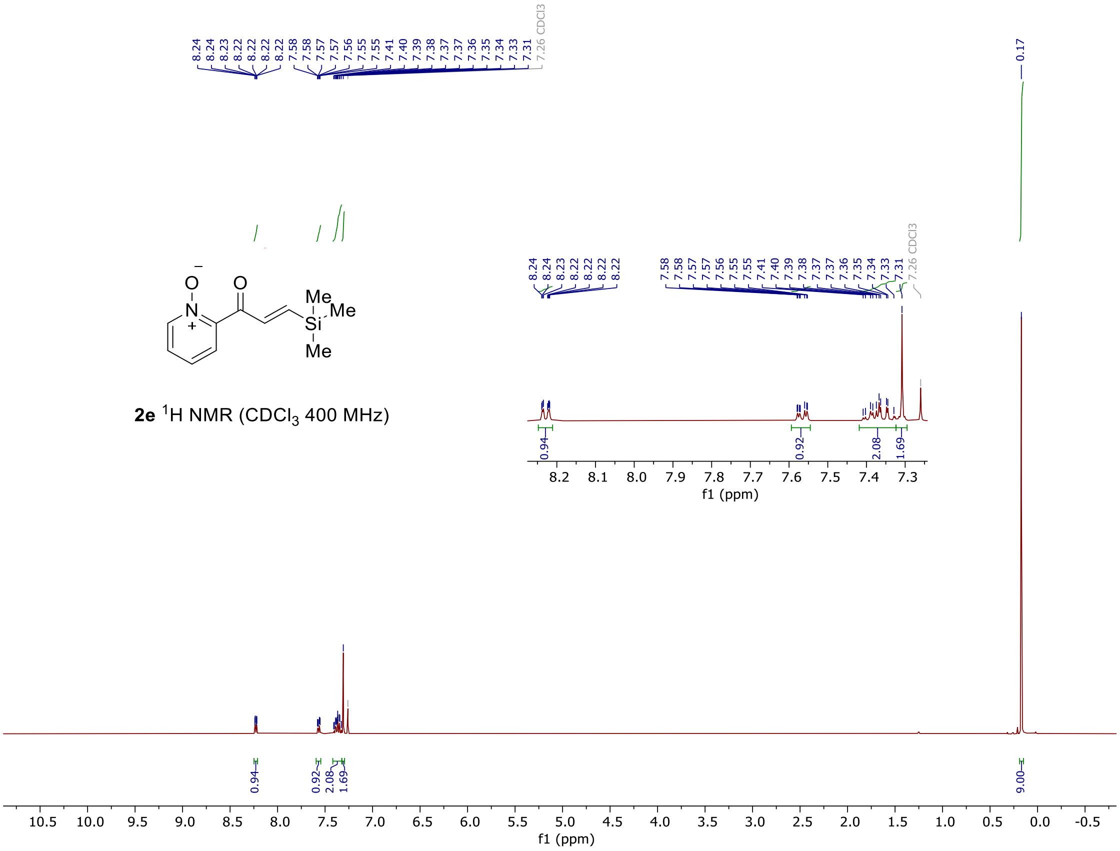

2e ^{1}H NMR ($CDCl_3$ 400 MHz)

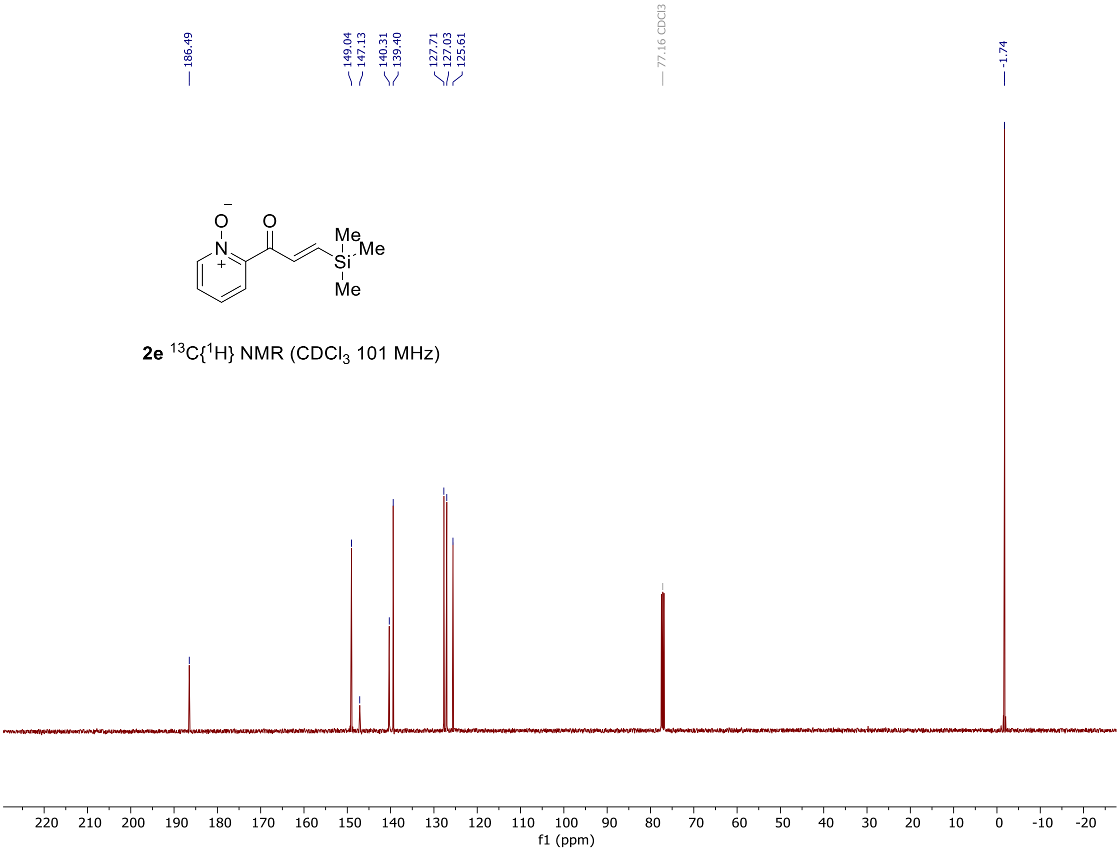

2e $^{13}C\{^{1}H\}$ NMR ($CDCl_3$ 101 MHz)

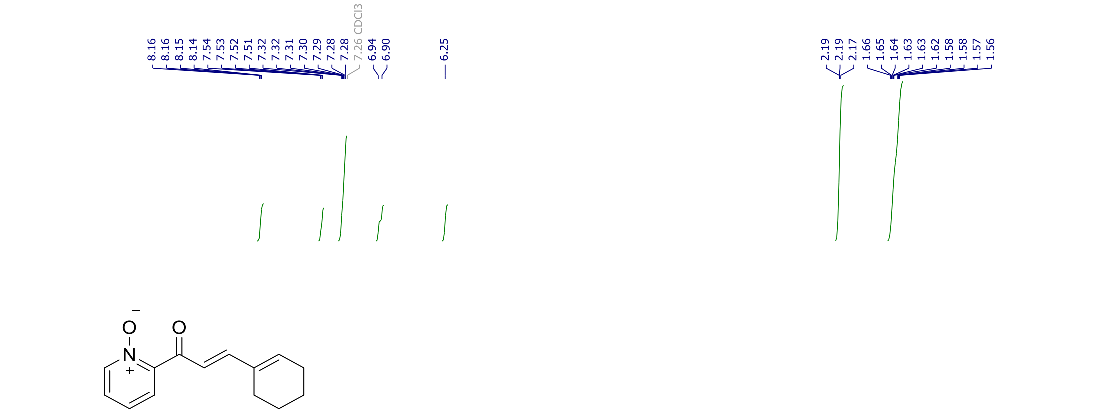

2f ^{1}H NMR ($CDCl_3$ 400 MHz)

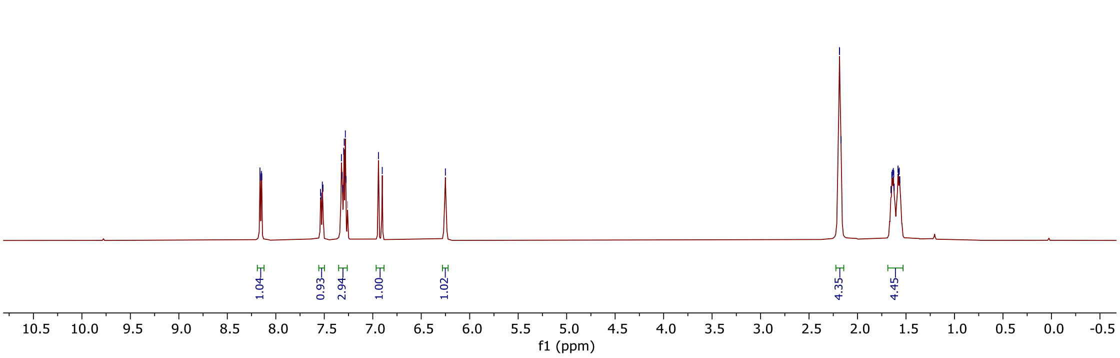

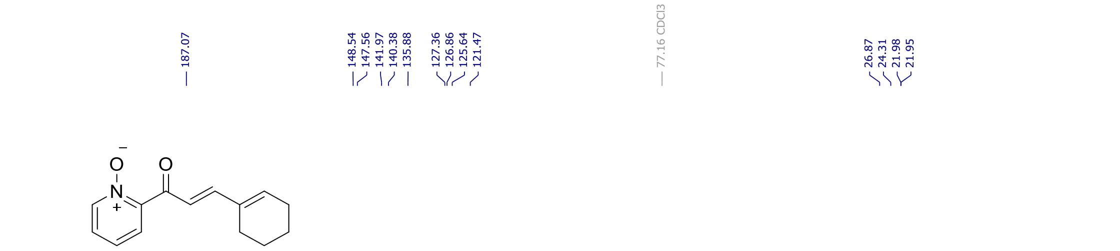

2f $^{13}C\{^{1}H\}$ NMR ($CDCl_3$ 101 MHz)

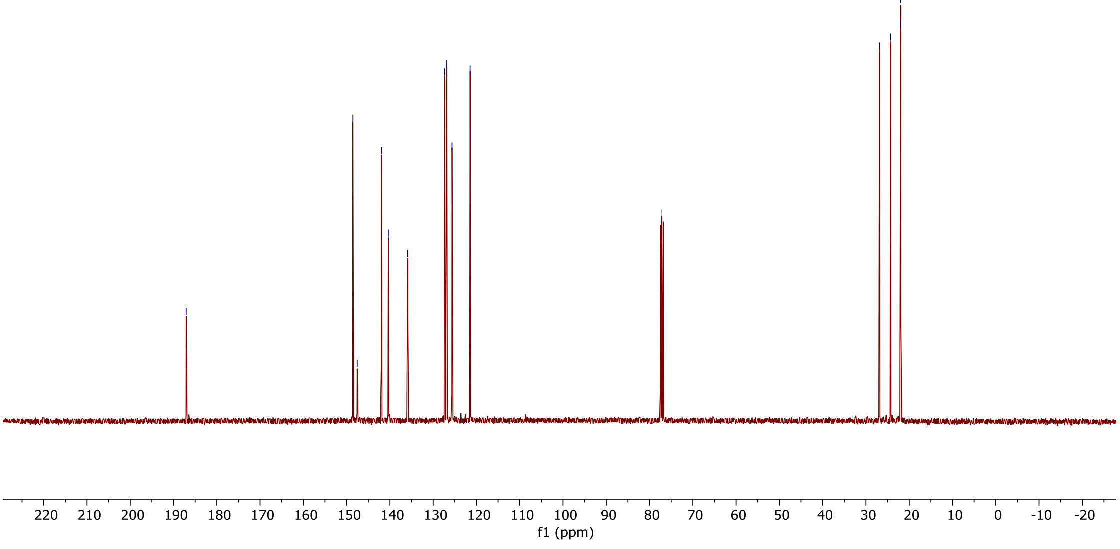

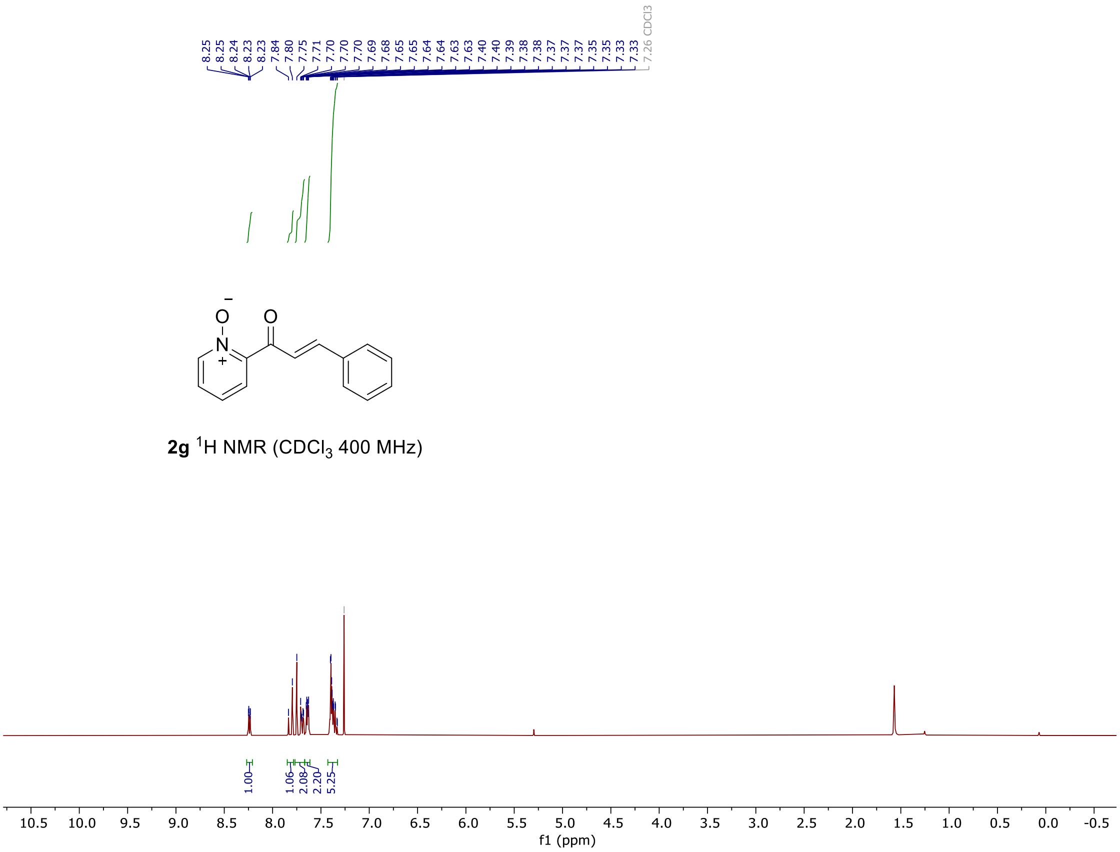

2g ^{1}H NMR (CDCl$_3$ 400 MHz)

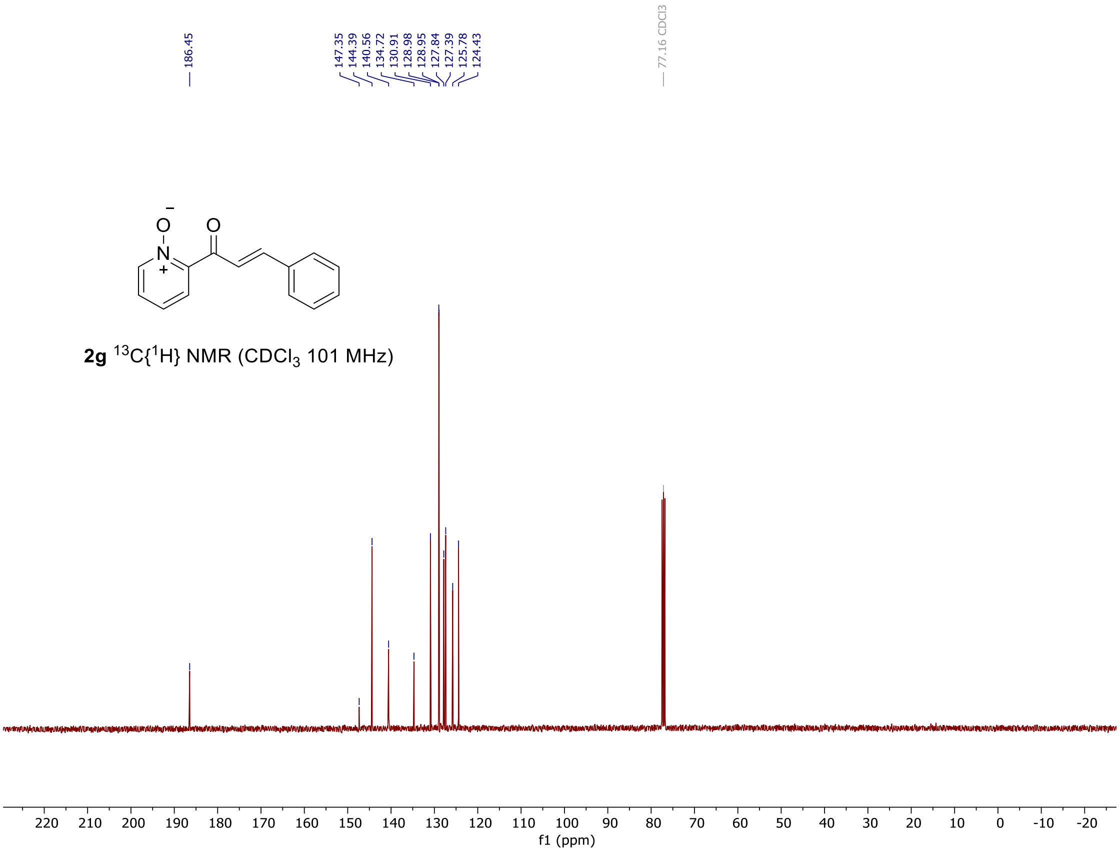

2g ^{13}C{^{1}H} NMR (CDCl$_3$ 101 MHz)

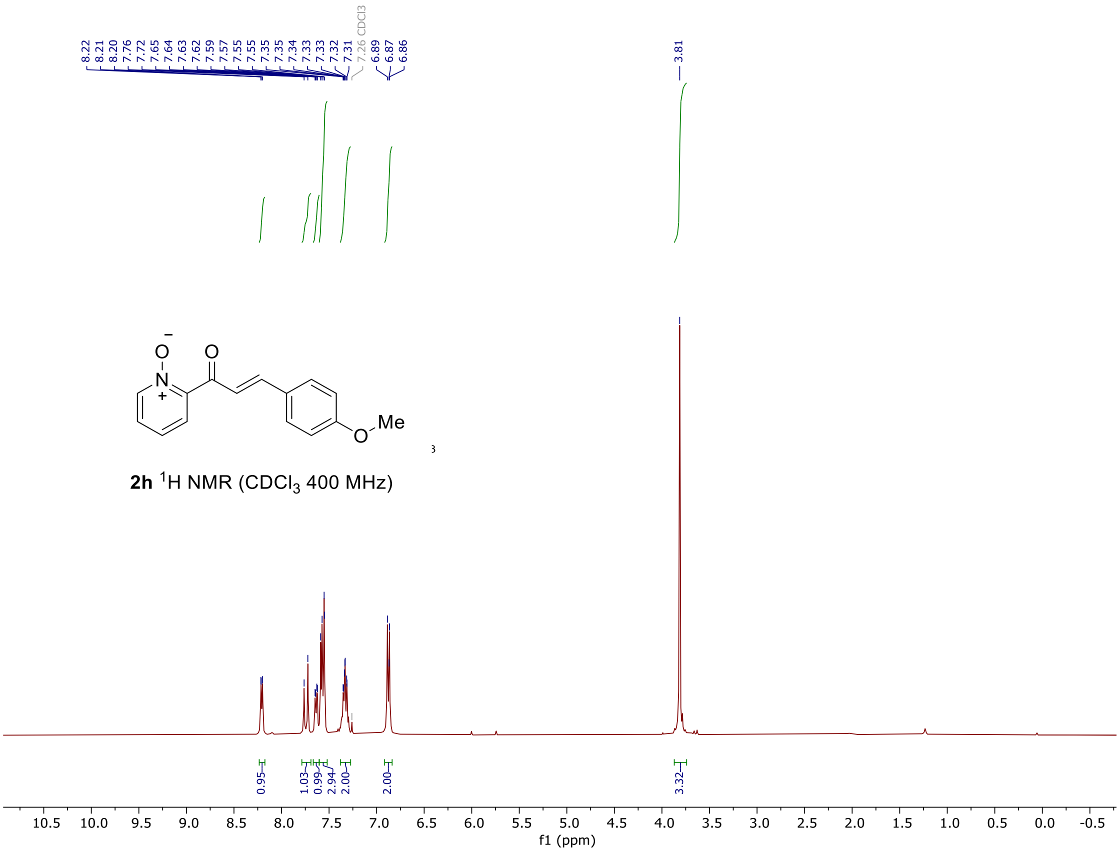

2h ^{1}H NMR ($CDCl_3$ 400 MHz)

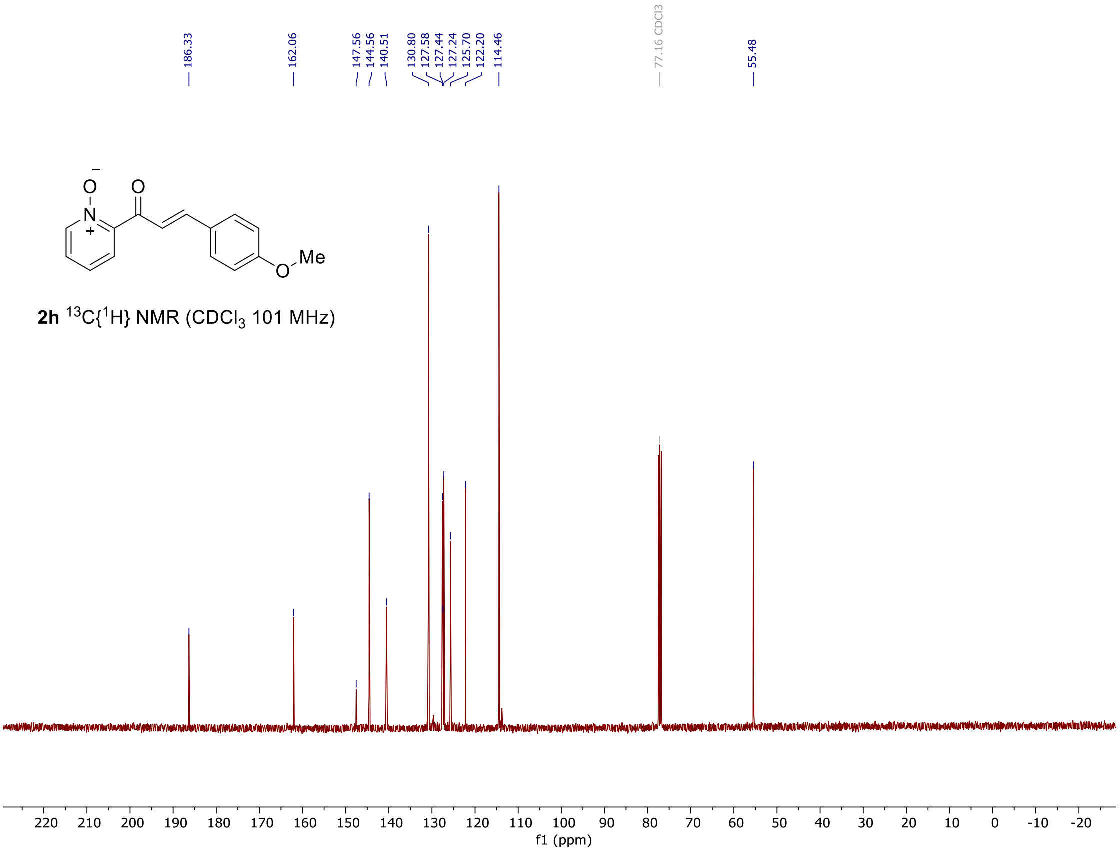

2h ^{13}C{^{1}H} NMR ($CDCl_3$ 101 MHz)

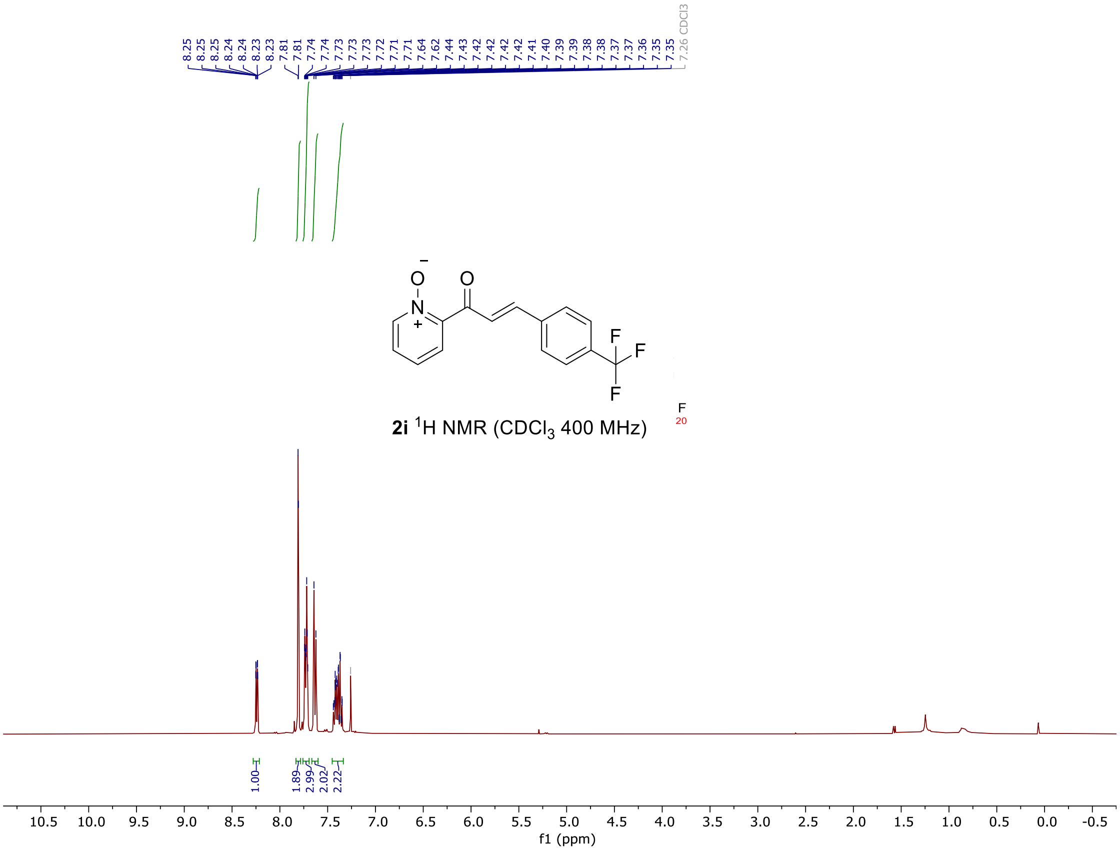

2i ^{1}H NMR ($CDCl_3$ 400 MHz)

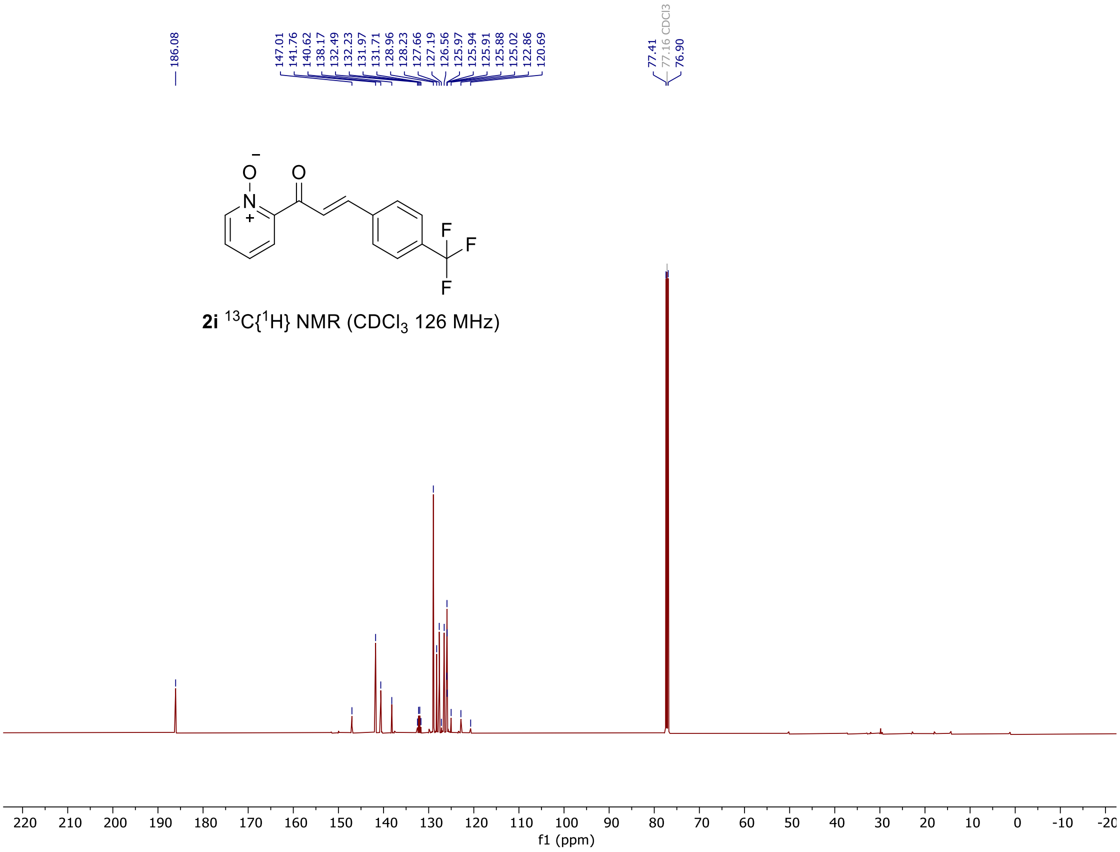

2i $^{13}C\{^{1}H\}$ NMR ($CDCl_3$ 126 MHz)

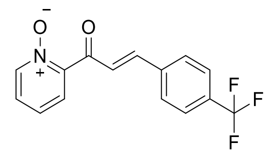

2i ^{19}F NMR ($CDCl_3$ 376 MHz)

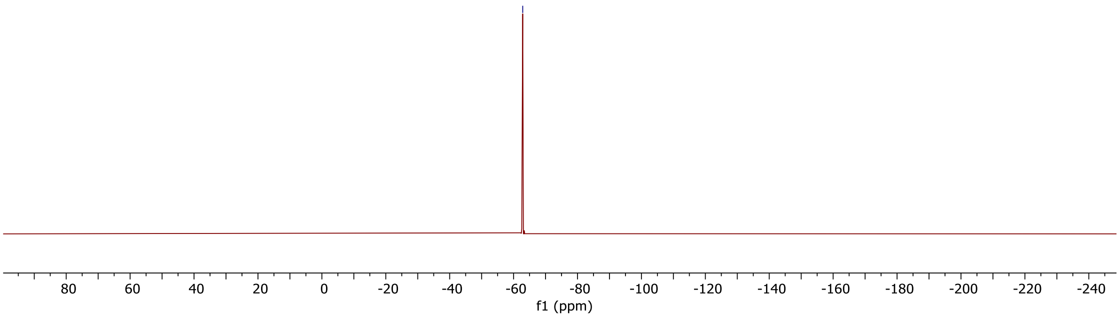

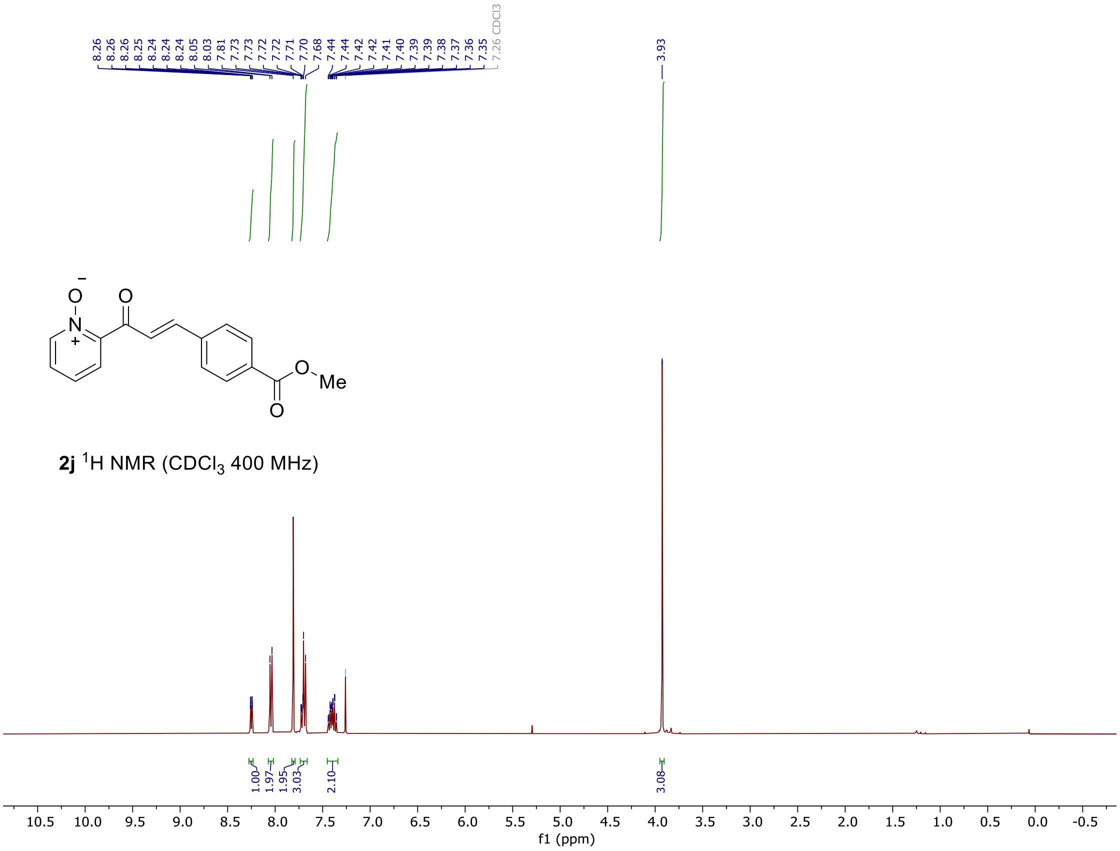

2j ^{1}H NMR ($CDCl_3$ 400 MHz)

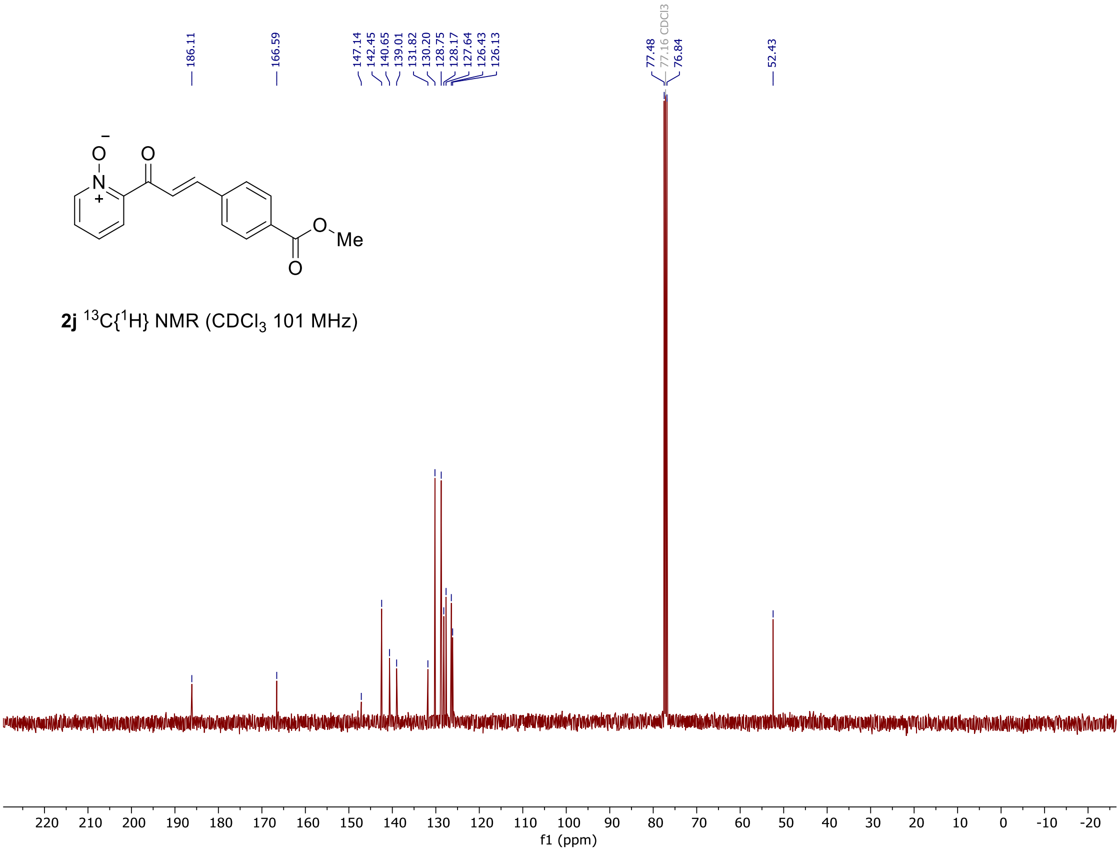

2j $^{13}C\{^{1}H\}$ NMR ($CDCl_3$ 101 MHz)

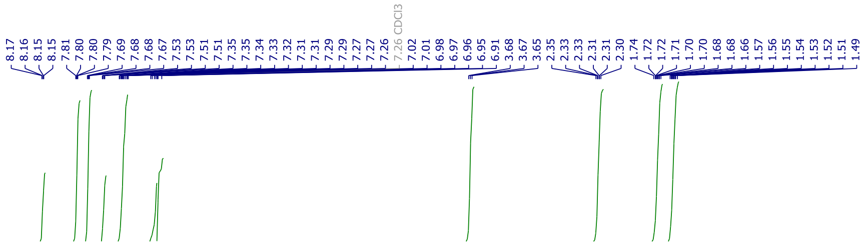

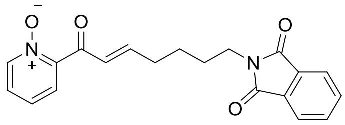

2k ^{1}H NMR (CDCl$_3$ 400 MHz)

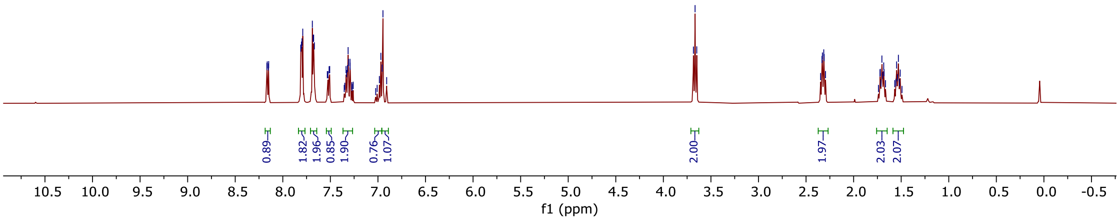

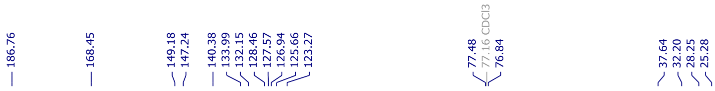

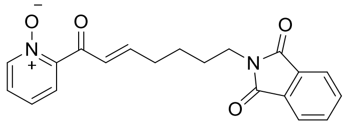

2k ^{13}C{^{1}H} NMR (CDCl$_3$ 101 MHz)

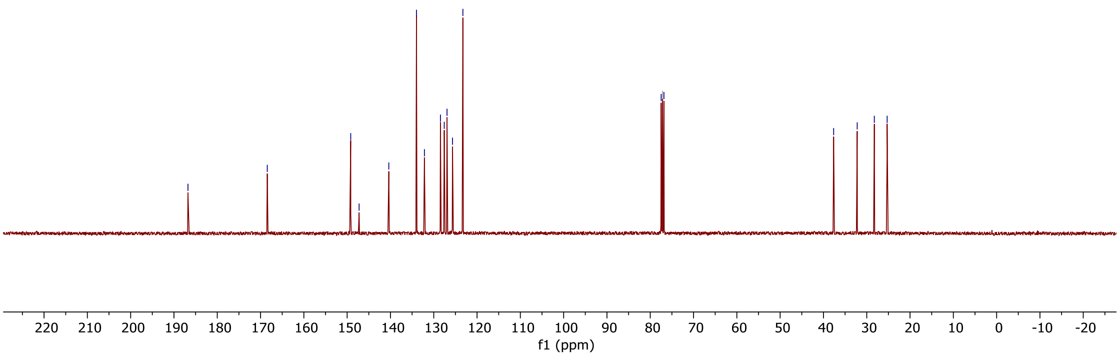

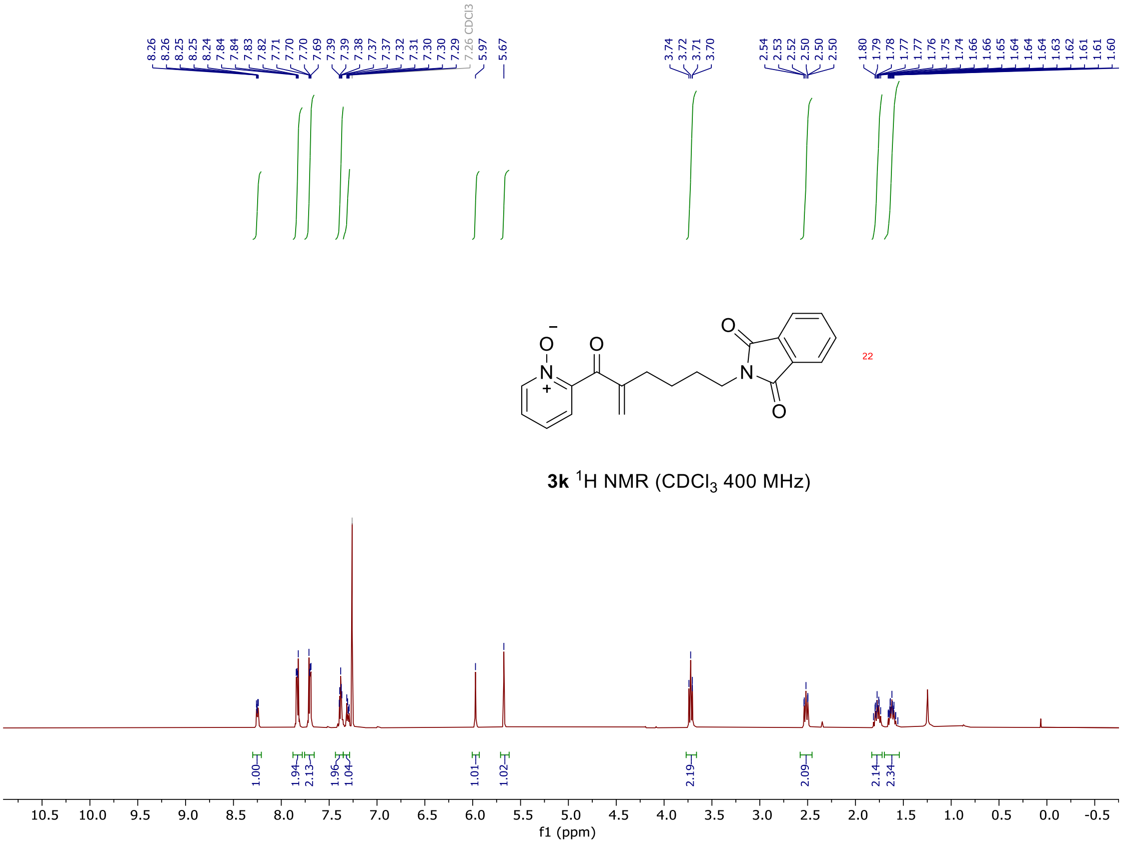

3k ^{1}H NMR ($CDCl_3$ 400 MHz)

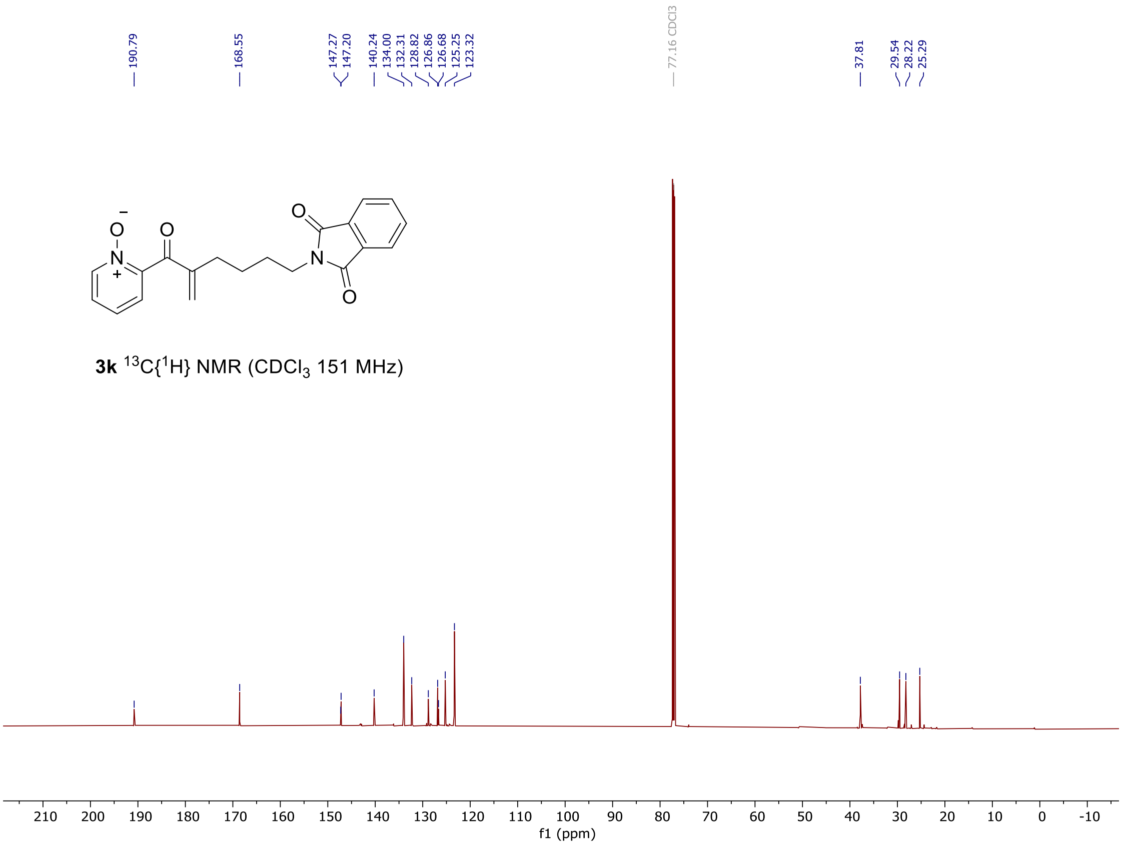

3k $^{13}C\{^{1}H\}$ NMR ($CDCl_3$ 151 MHz)

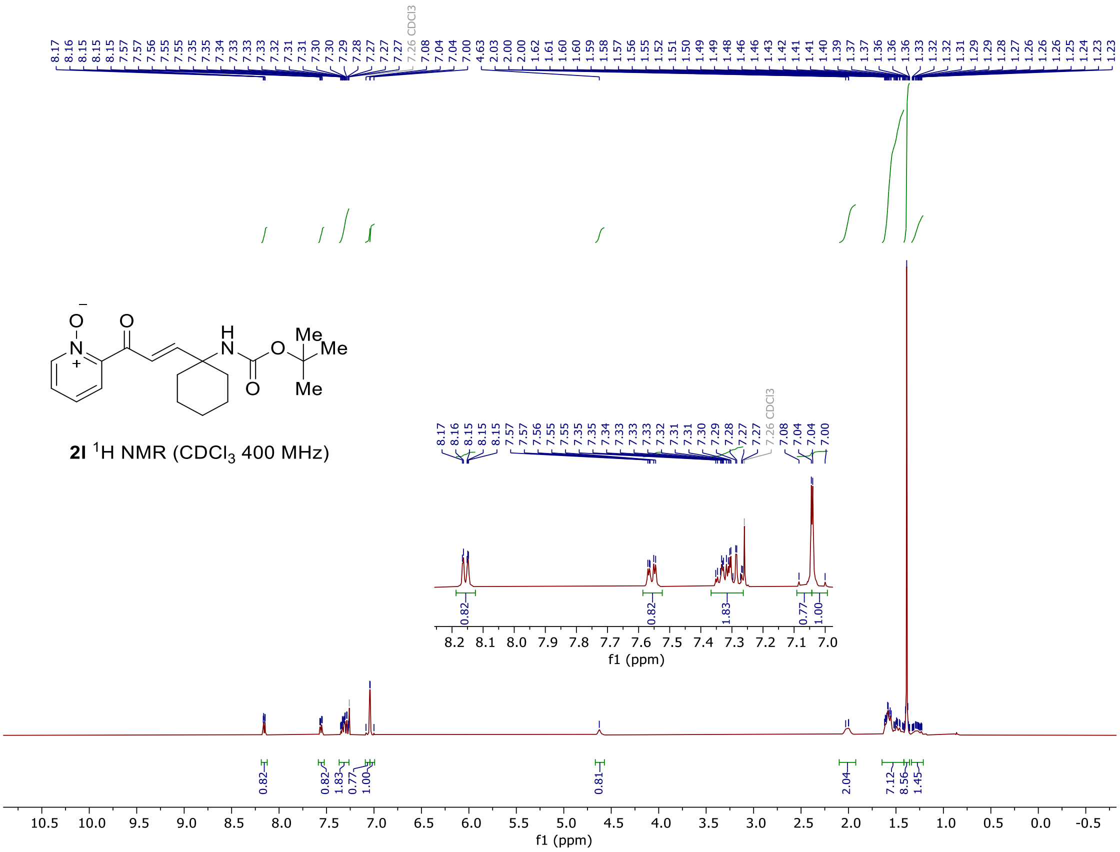

2l ^{1}H NMR ($CDCl_3$ 400 MHz)

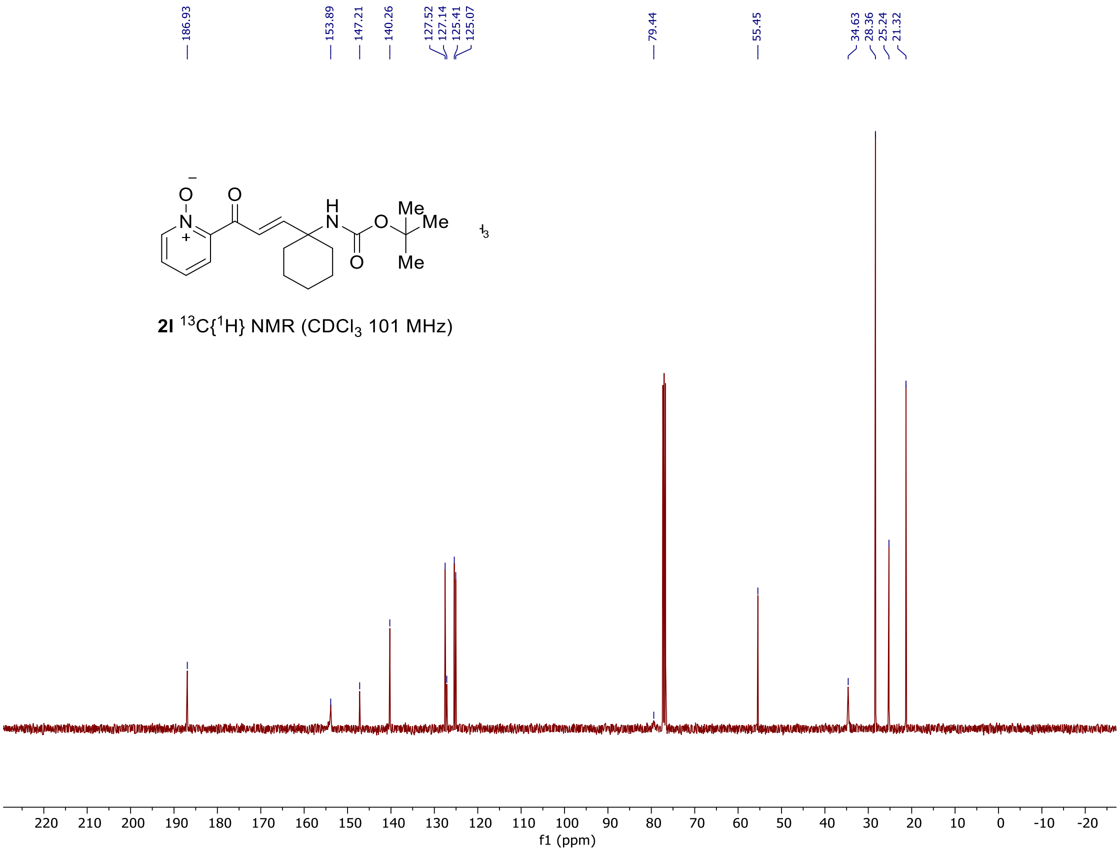

2l $^{13}C\{^{1}H\}$ NMR ($CDCl_3$ 101 MHz)

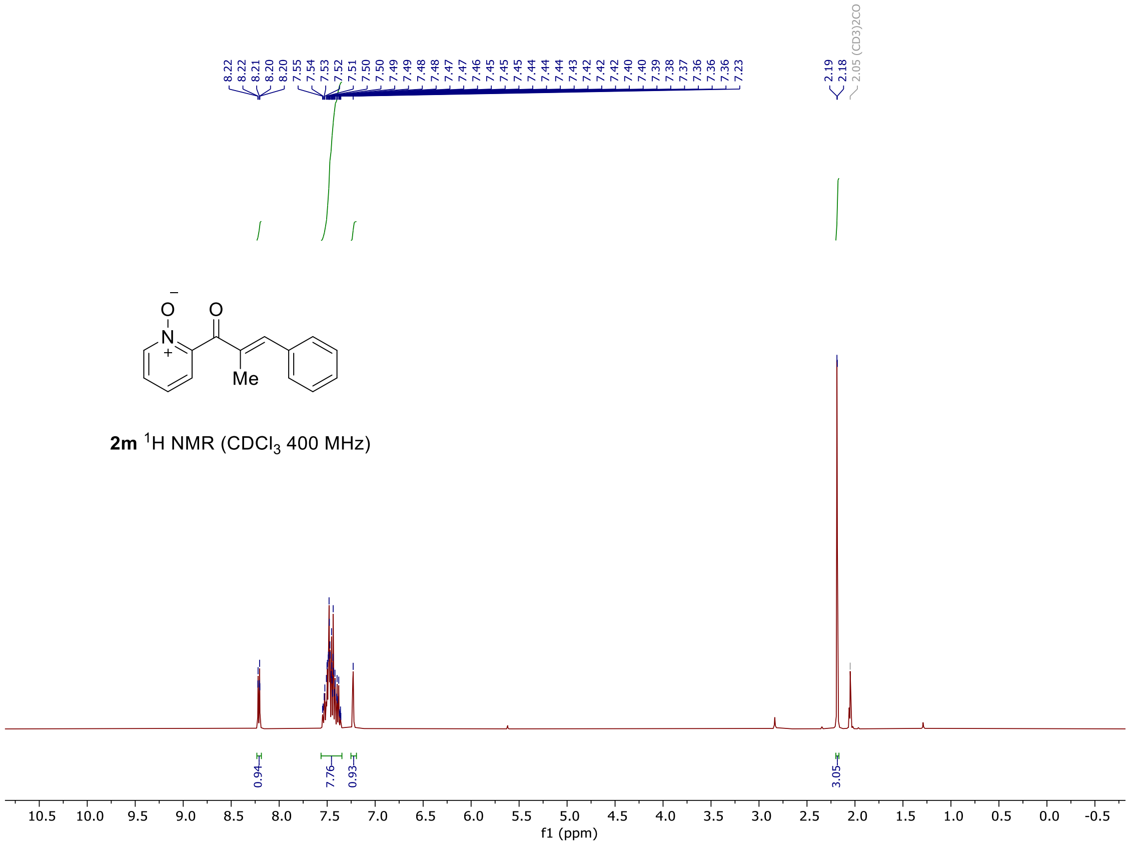

2m ^{1}H NMR ($CDCl_3$ 400 MHz)

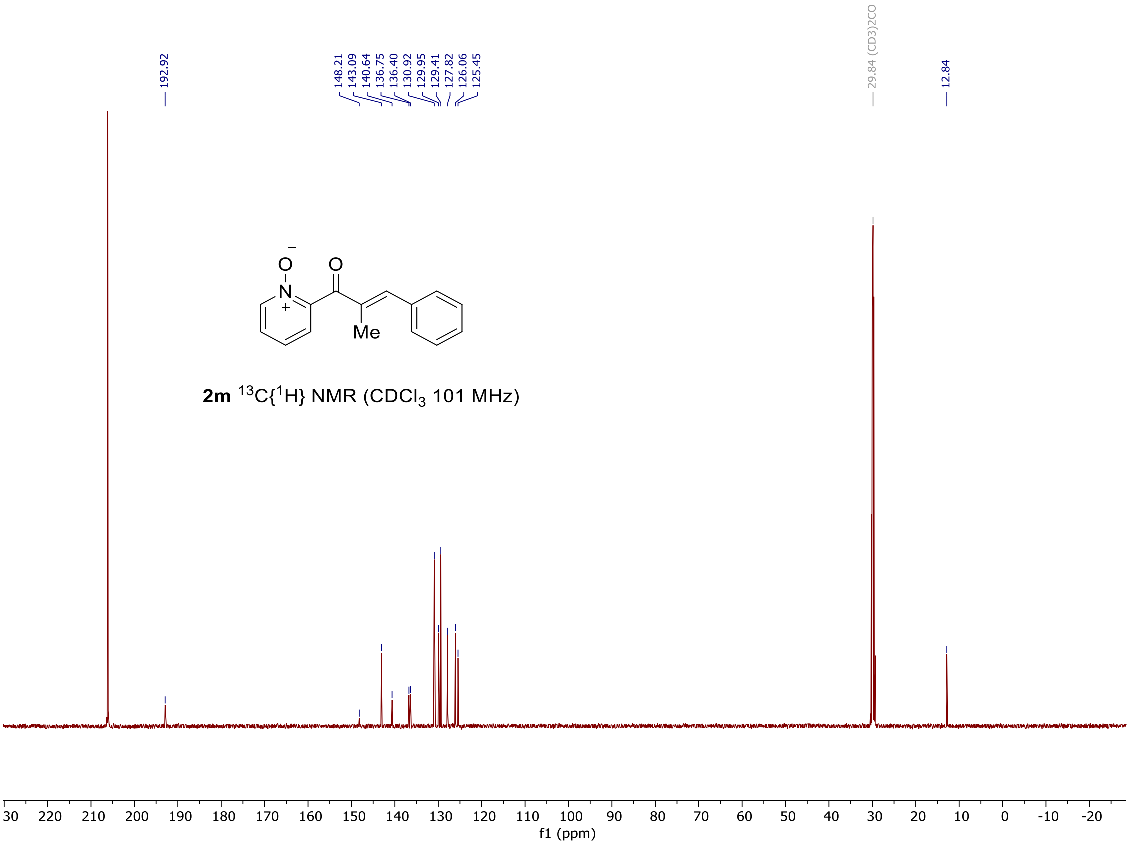

2m $^{13}C\{^{1}H\}$ NMR ($CDCl_3$ 101 MHz)

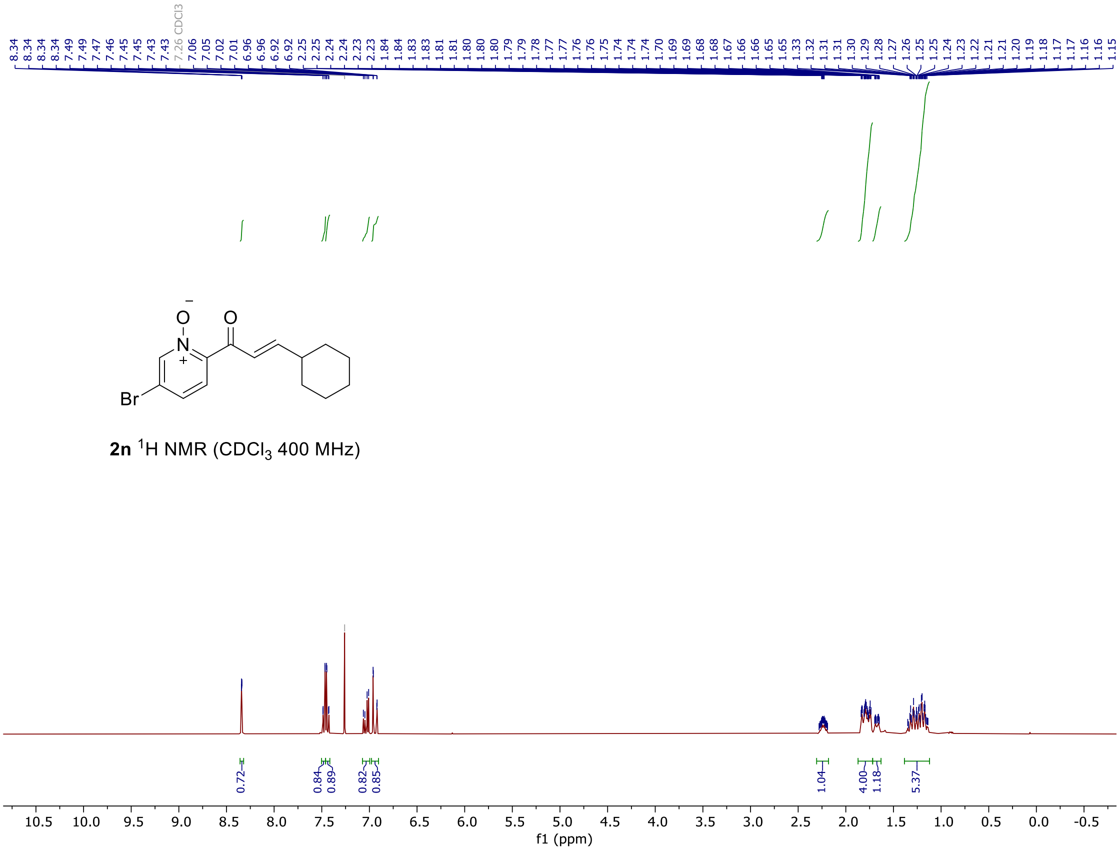

2n ^{1}H NMR ($CDCl_3$ 400 MHz)

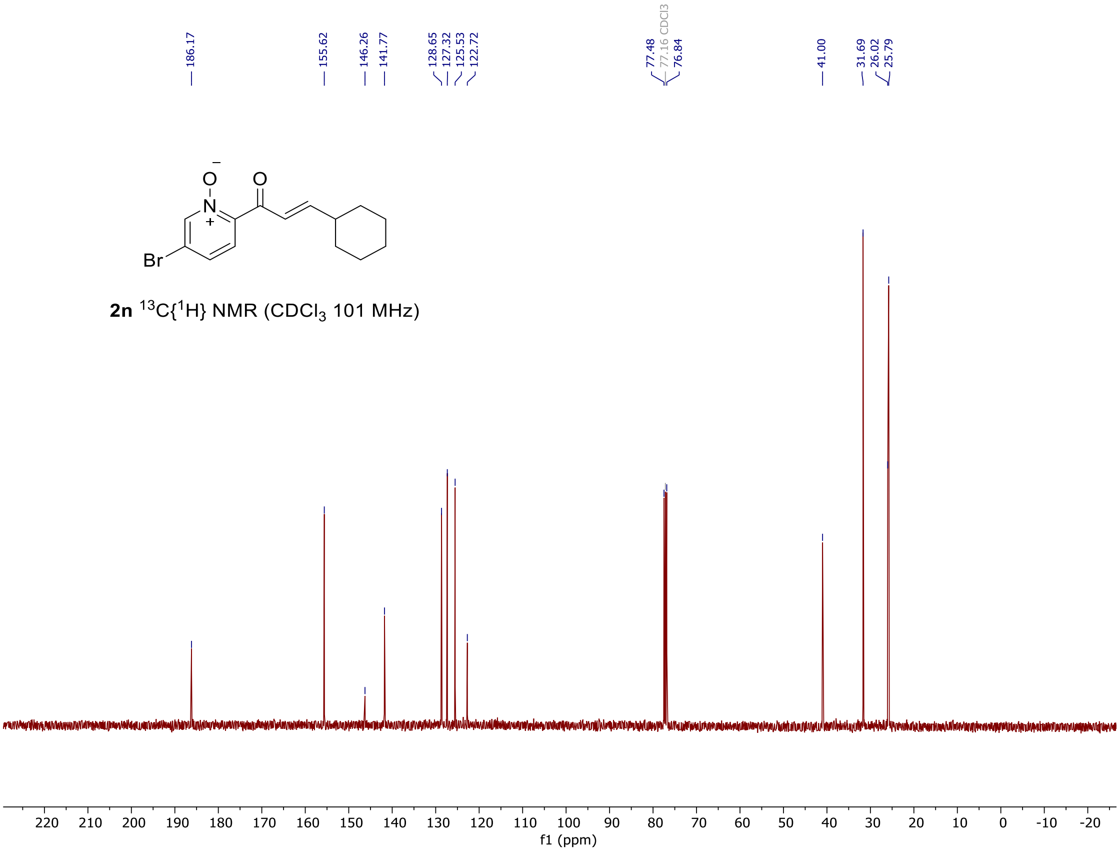

2n ^{13}C{^{1}H} NMR ($CDCl_3$ 101 MHz)

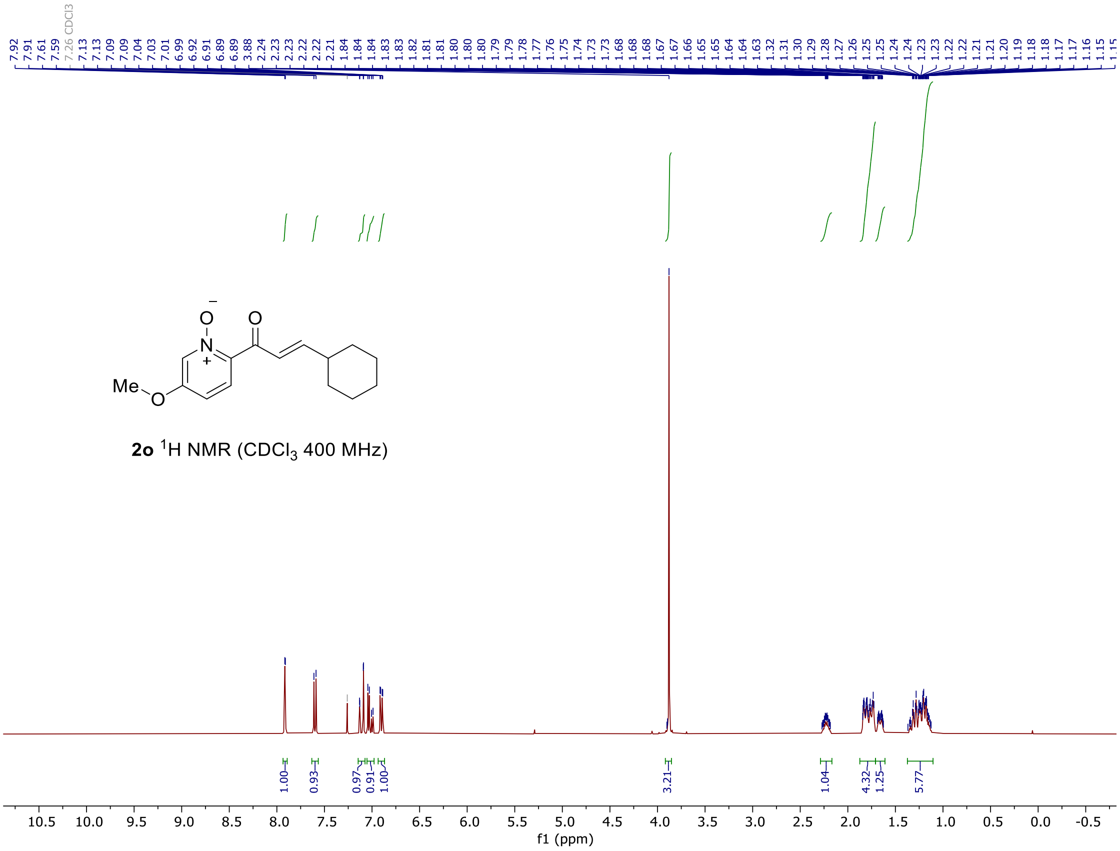
7.92 7.91 7.61 7.59 7.26 CDCl3 7.13 7.13 7.09 7.09 7.04 7.03 7.01 6.99 6.92 6.91 6.89 6.89 3.88 2.24 2.23 2.23 2.22 2.22 2.21 2.21 1.84 1.84 1.84 1.83 1.83 1.83 1.82 1.81 1.81 1.80 1.80 1.80 1.79 1.79 1.78 1.77 1.76 1.75 1.74 1.73 1.73 1.68 1.68 1.67 1.67 1.66 1.65 1.65 1.64 1.64 1.63 1.32 1.31 1.30 1.29 1.28 1.27 1.26 1.25 1.25 1.24 1.24 1.23 1.23 1.22 1.22 1.21 1.21 1.20 1.19 1.18 1.18 1.17 1.17 1.16 1.15 1.15
O
N
+
O
Me
O
2o ^{1}H NMR (CDCl$_3$ 400 MHz)
1.00
0.93
0.97
0.91
1.00
3.21
1.04
4.32
1.25
5.77
10.5 10.0 9.5 9.0 8.5 8.0 7.5 7.0 6.5 6.0 5.5 5.0 4.5 4.0 3.5 3.0 2.5 2.0 1.5 1.0 0.5 0.0 -0.5
f1 (ppm)

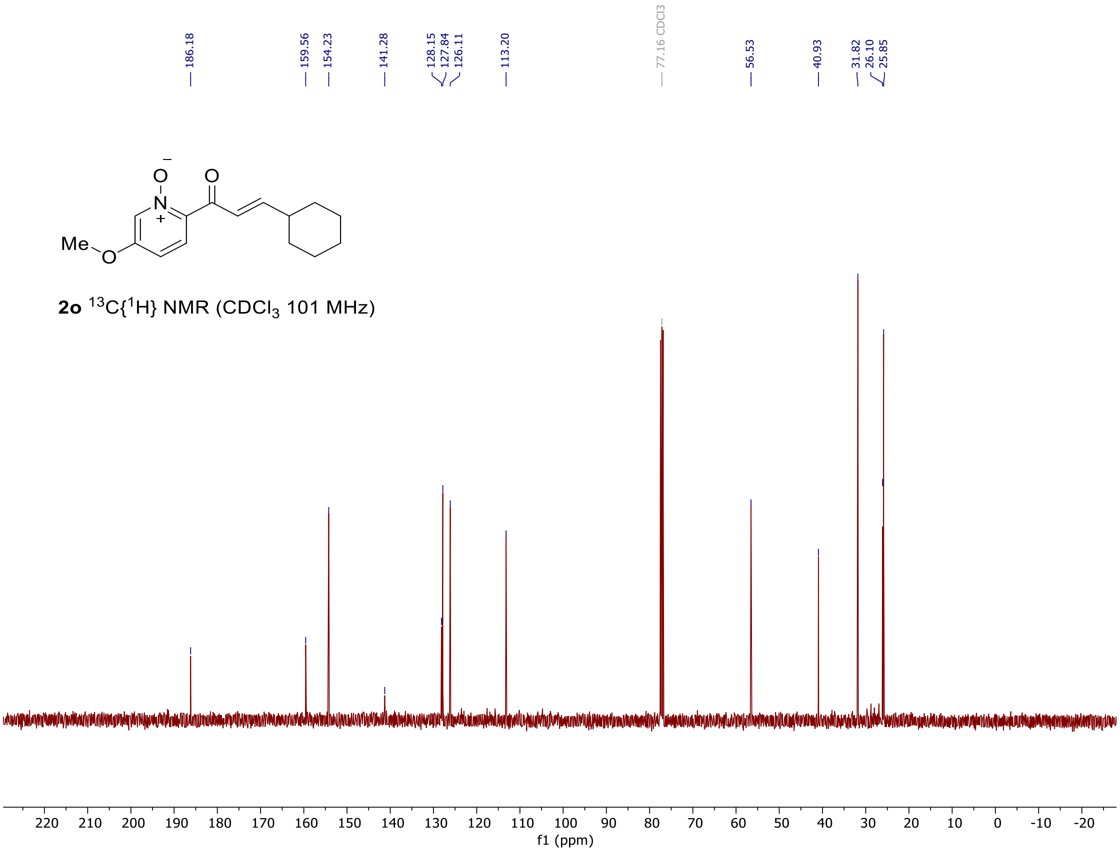
186.18
159.56
154.23
141.28
128.15
127.84
126.11
113.20
77.16 CDCl3
56.53
40.93
31.82
26.10
25.85
O
N
+
O
Me
O
2o ^{13}C{^{1}H} NMR (CDCl$_3$ 101 MHz)
220 210 200 190 180 170 160 150 140 130 120 110 100 90 80 70 60 50 40 30 20 10 0 -10 -20
f1 (ppm)

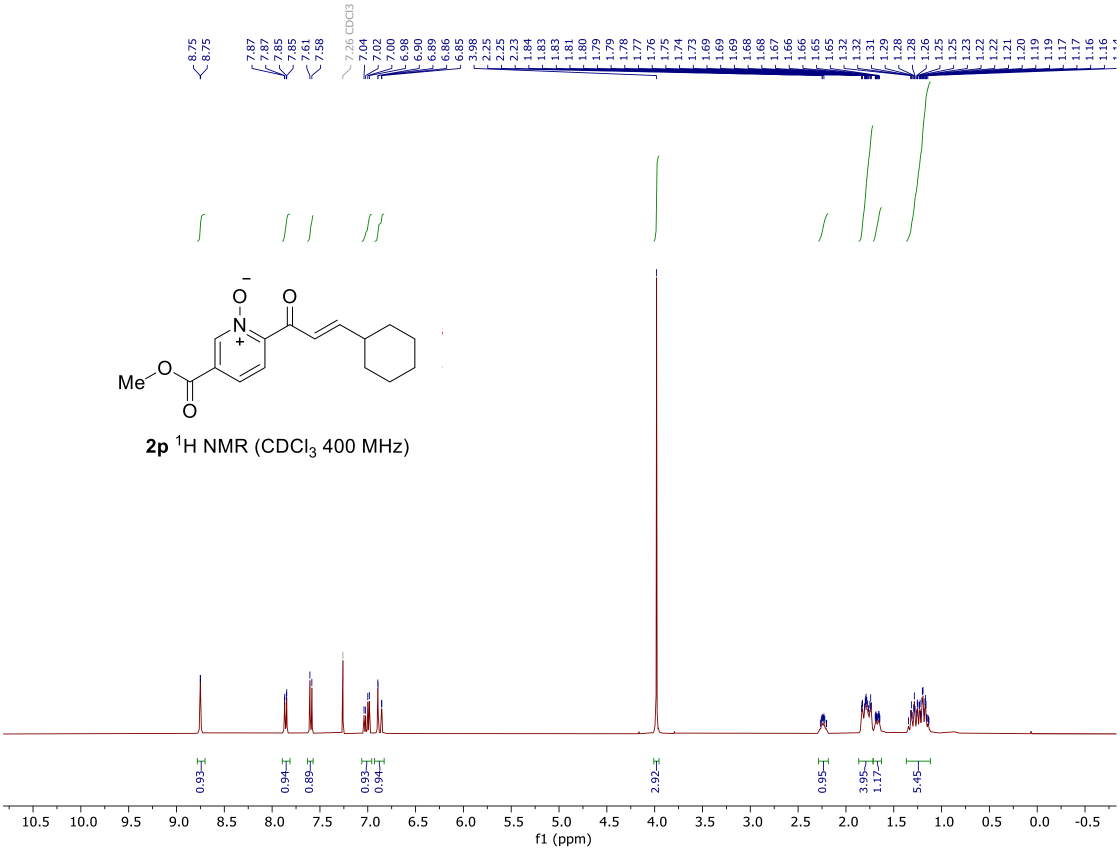
2p ^{1}H NMR ($CDCl_3$ 400 MHz)
0.93
0.94
0.89
0.93
0.94
2.92
0.95
3.95
1.17
5.45
f1 (ppm)

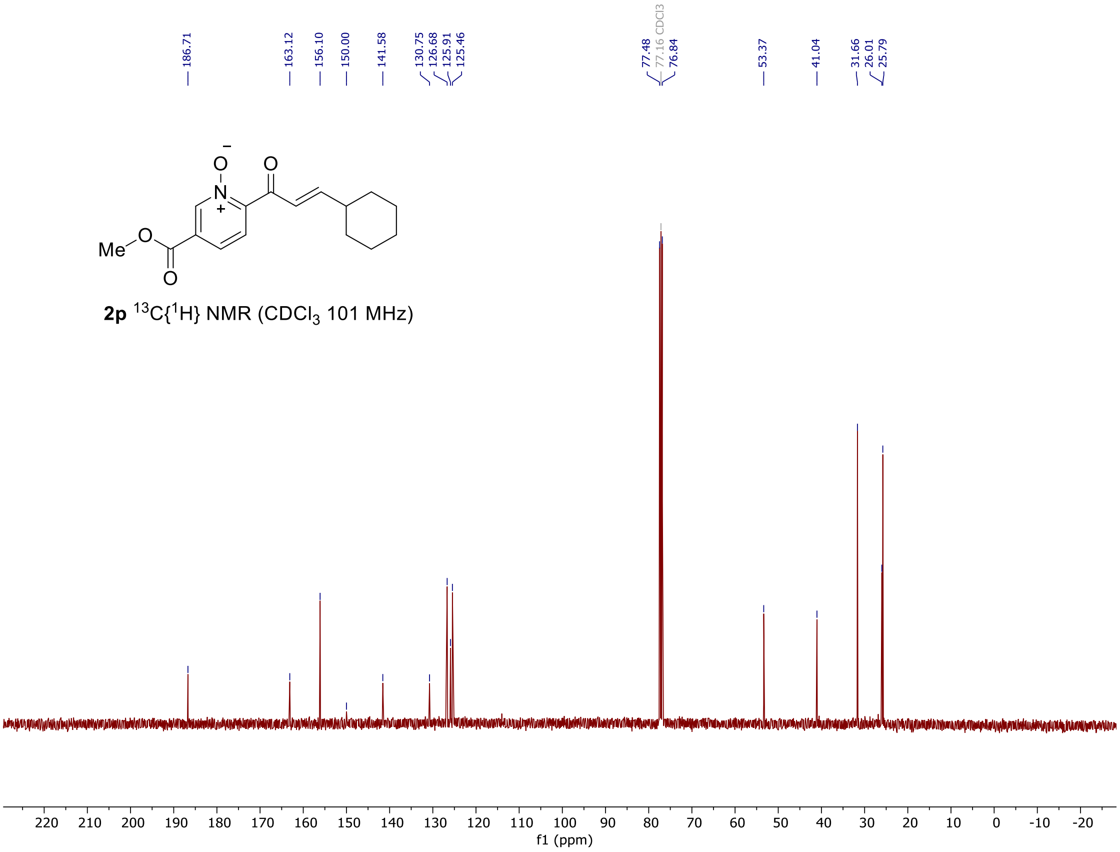
2p $^{13}C\{^1H\}$ NMR ($CDCl_3$ 101 MHz)
186.71
163.12
156.10
150.00
141.58
130.75
126.68
125.91
125.46
77.48
77.16 CDCl3
76.84
53.37
41.04
31.66
26.01
25.79
f1 (ppm)

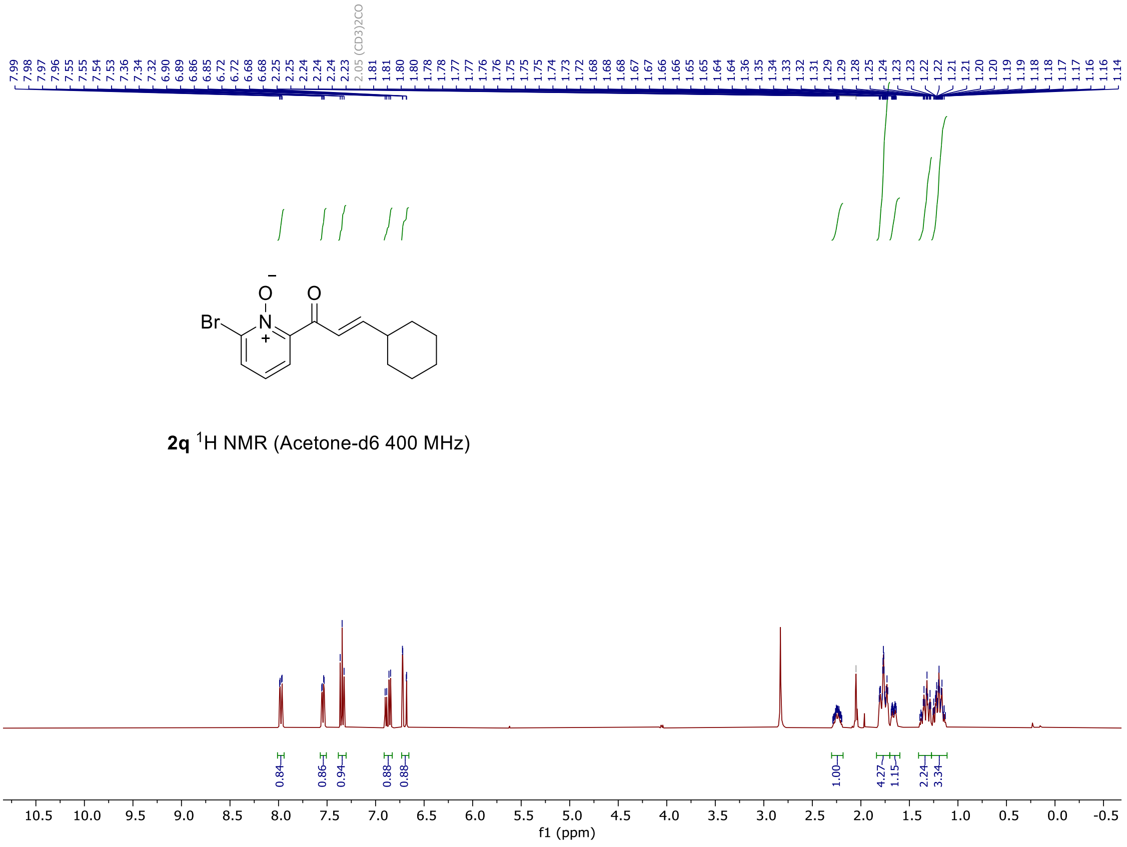

2q ^{1}H NMR (Acetone-d6 400 MHz)

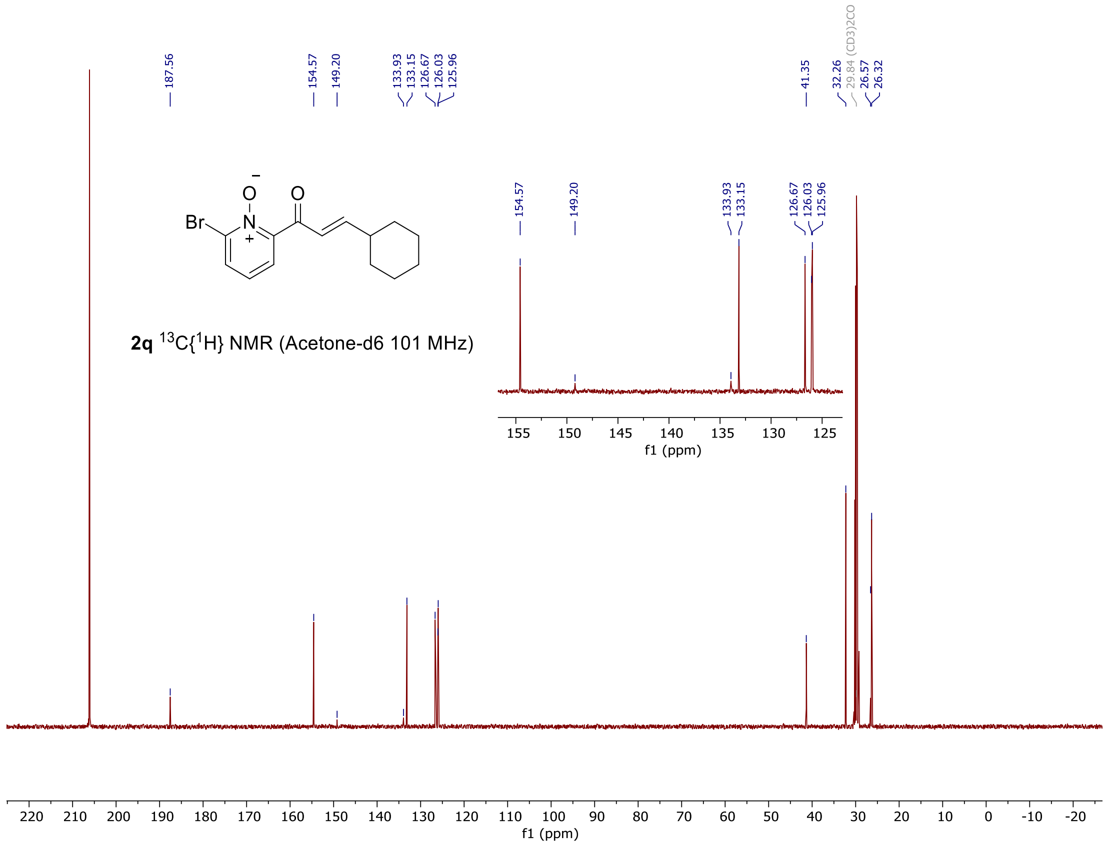

2q $^{13}C\{^{1}H\}$ NMR (Acetone-d6 101 MHz)

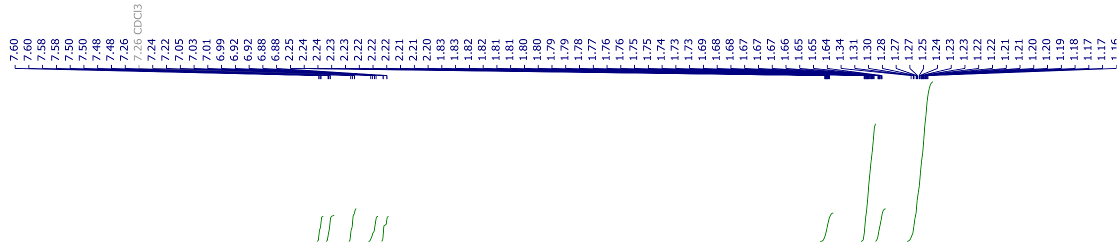

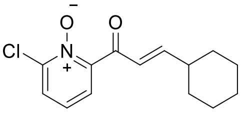

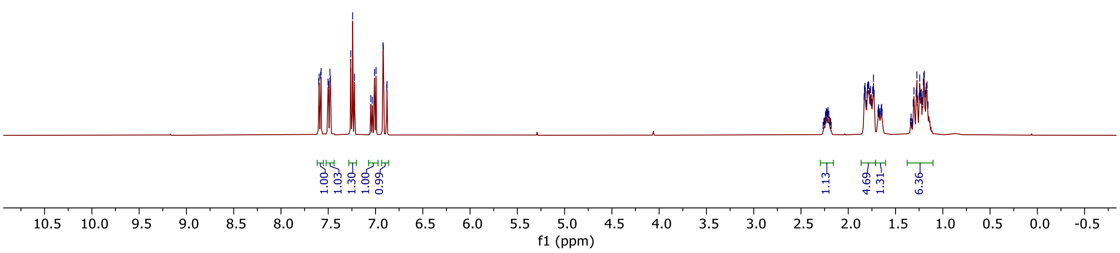

2r ^{1}H NMR ($CDCl_3$ 400 MHz)

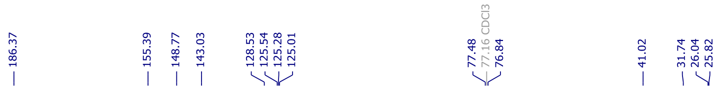

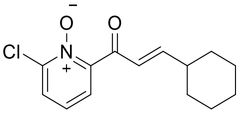

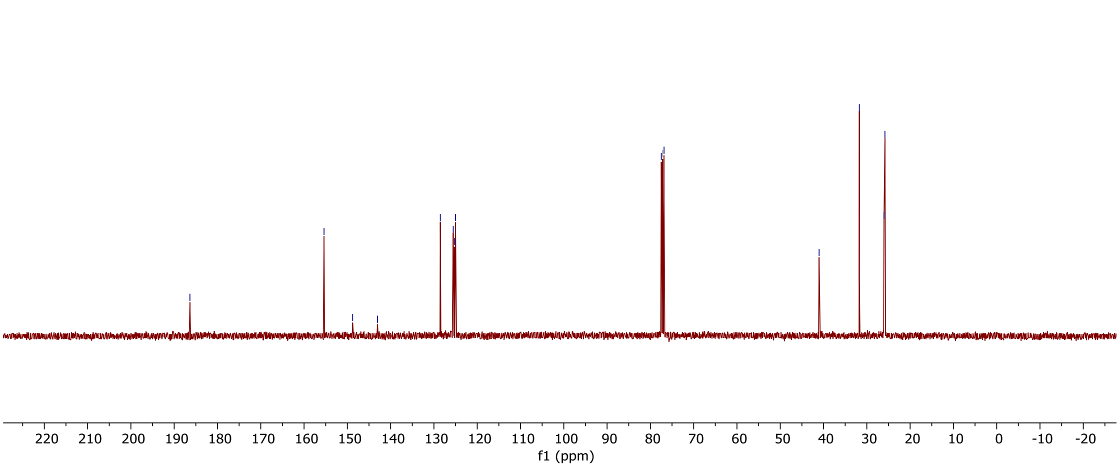

2r ^{13}C{^{1}H} NMR ($CDCl_3$ 101 MHz)

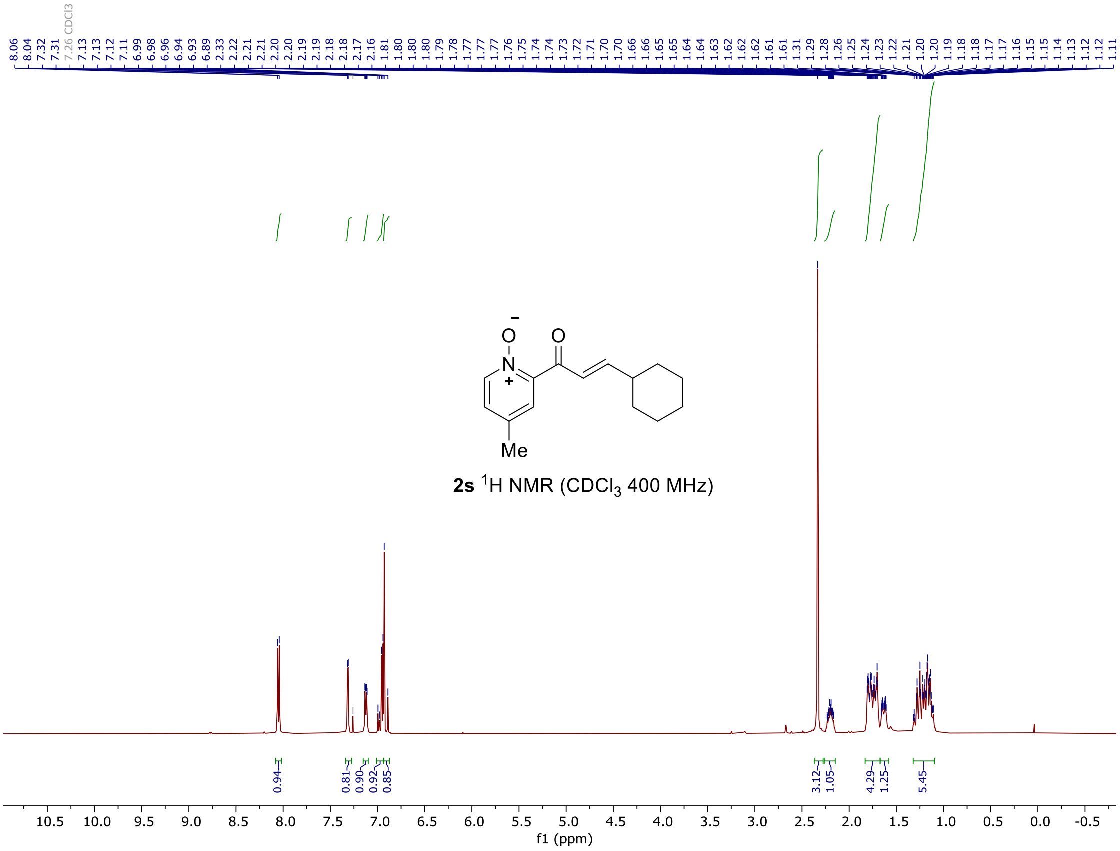
2s ^{1}H NMR ($CDCl_3$ 400 MHz)
f1 (ppm)

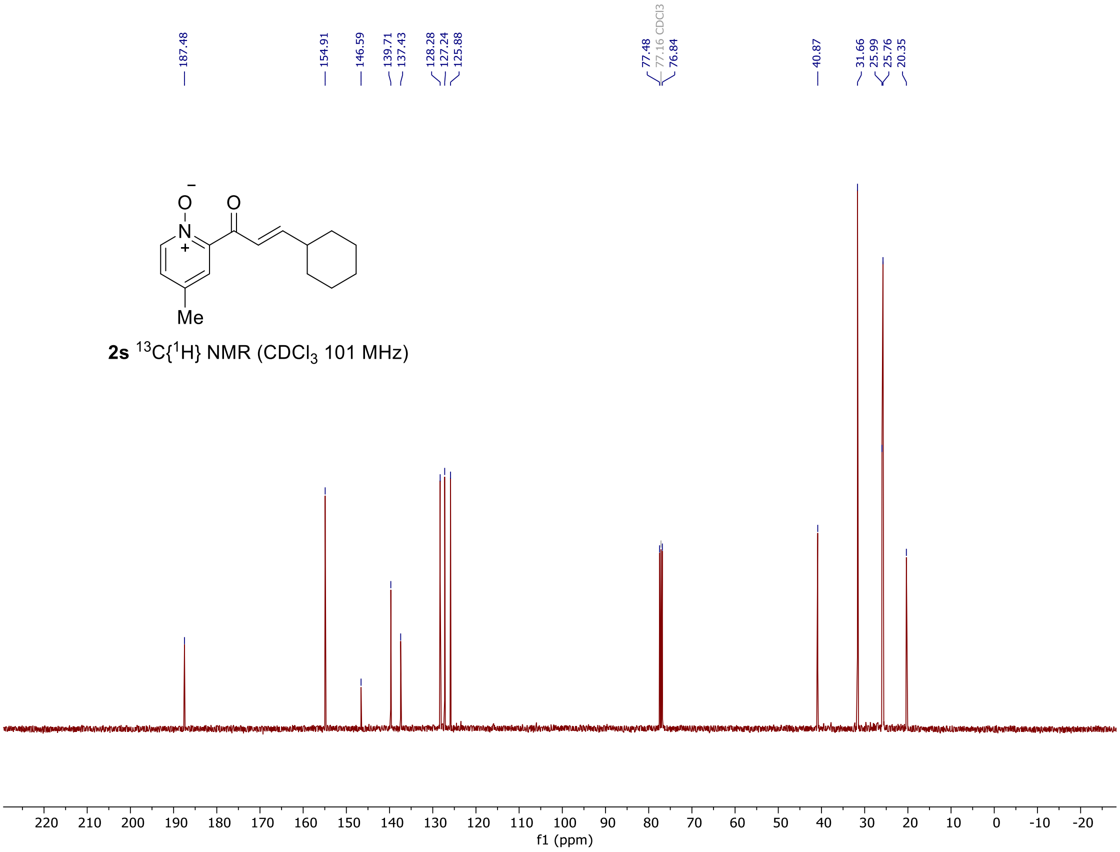
2s ^{13}C{^{1}H} NMR ($CDCl_3$ 101 MHz)
f1 (ppm)

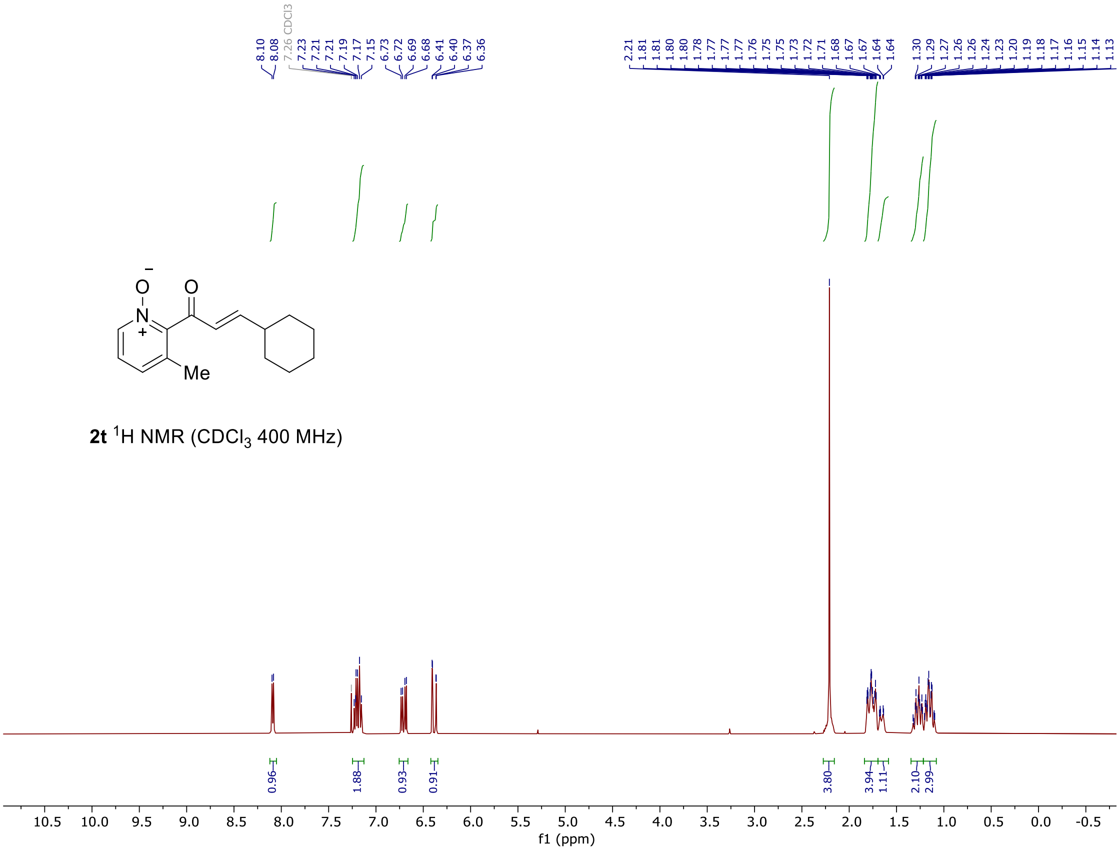

2t ^{1}H NMR (CDCl$_3$ 400 MHz)

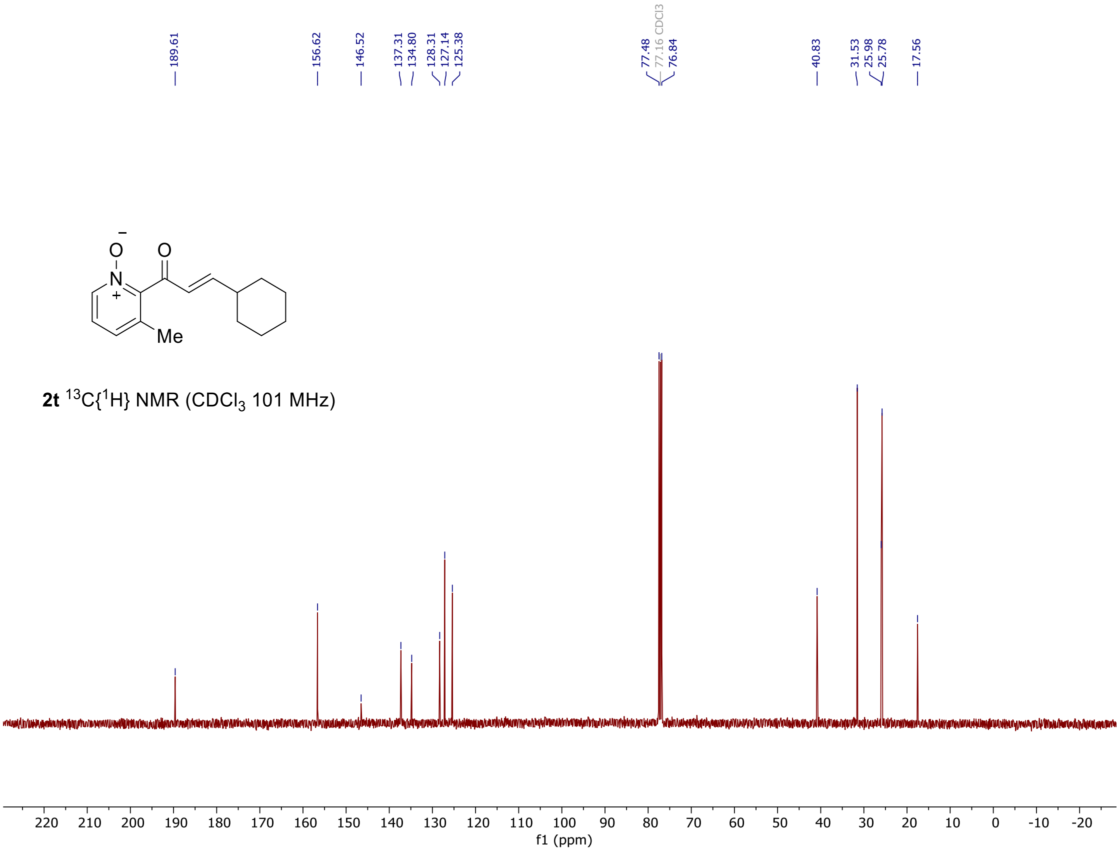

2t ^{13}C{^{1}H} NMR (CDCl$_3$ 101 MHz)

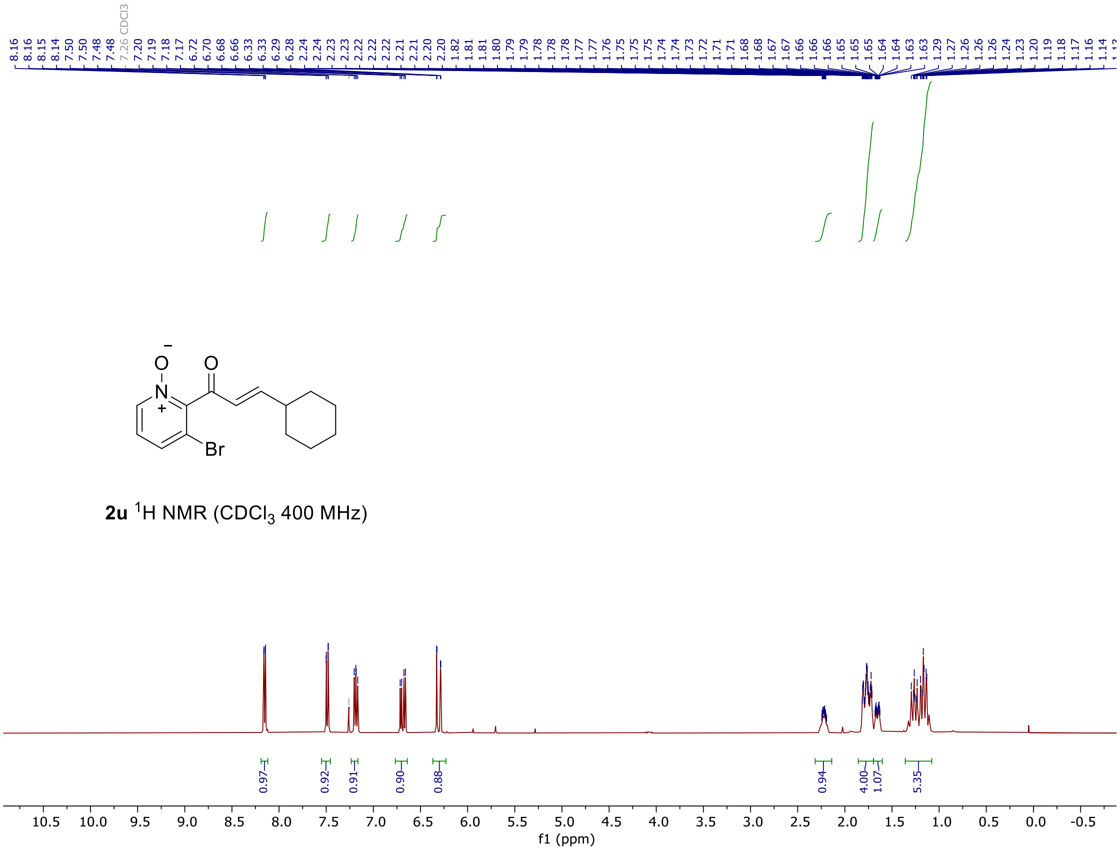

2u ^{1}H NMR ($CDCl_3$ 400 MHz)

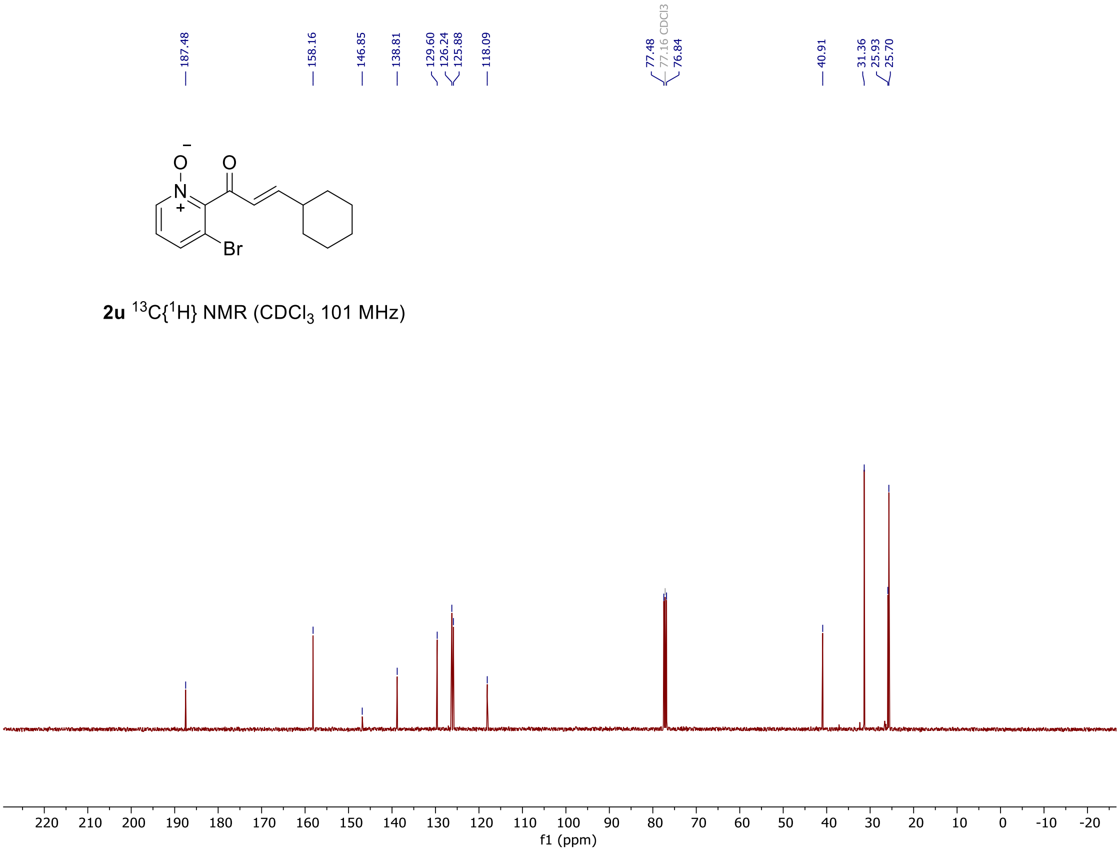

2u $^{13}C\{^1H\}$ NMR ($CDCl_3$ 101 MHz)

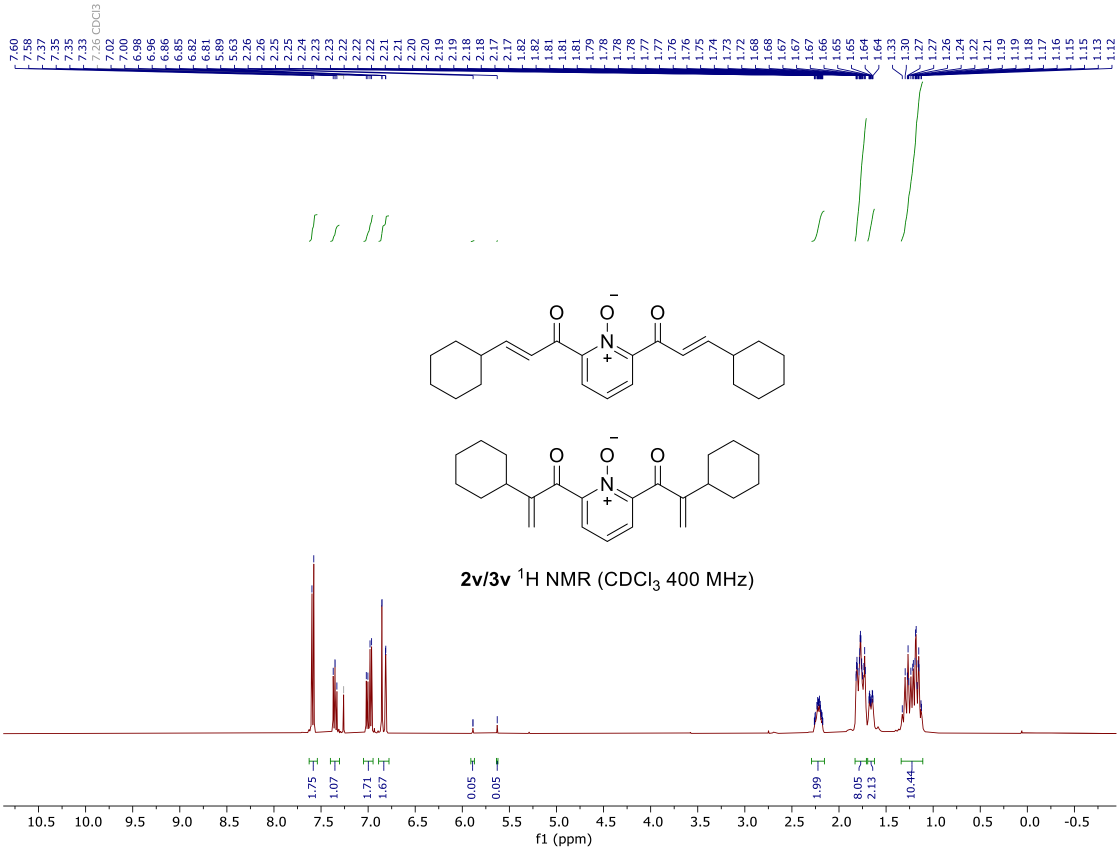

2v/3v ^{1}H NMR ($CDCl_3$ 400 MHz)

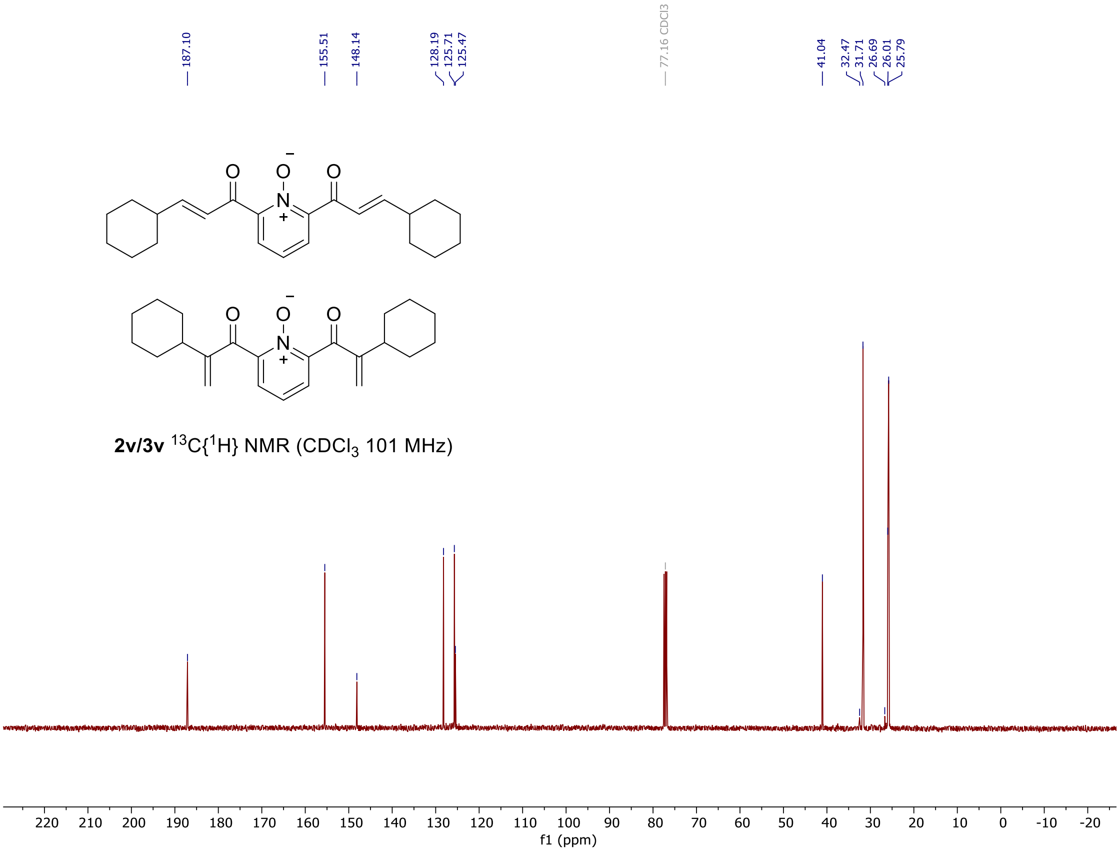

2v/3v ^{13}C{^{1}H} NMR ($CDCl_3$ 101 MHz)

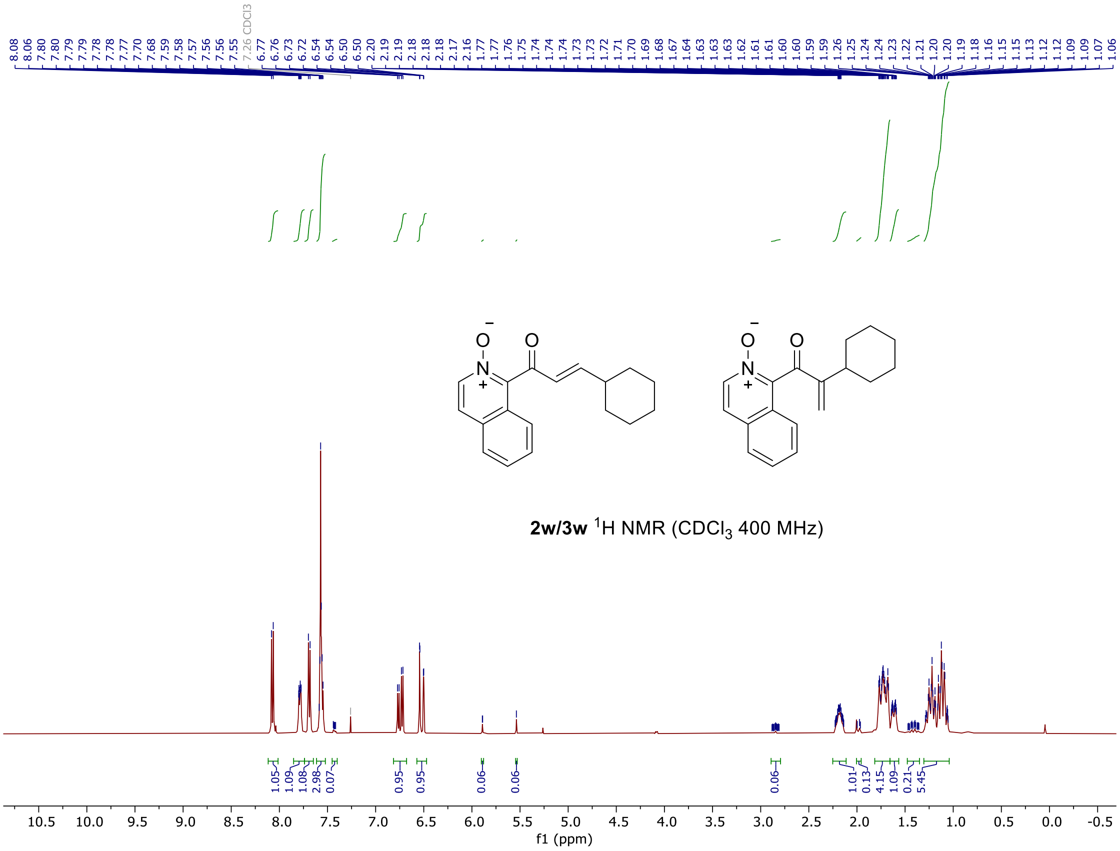

2w/3w ^{1}H NMR ($CDCl_3$ 400 MHz)

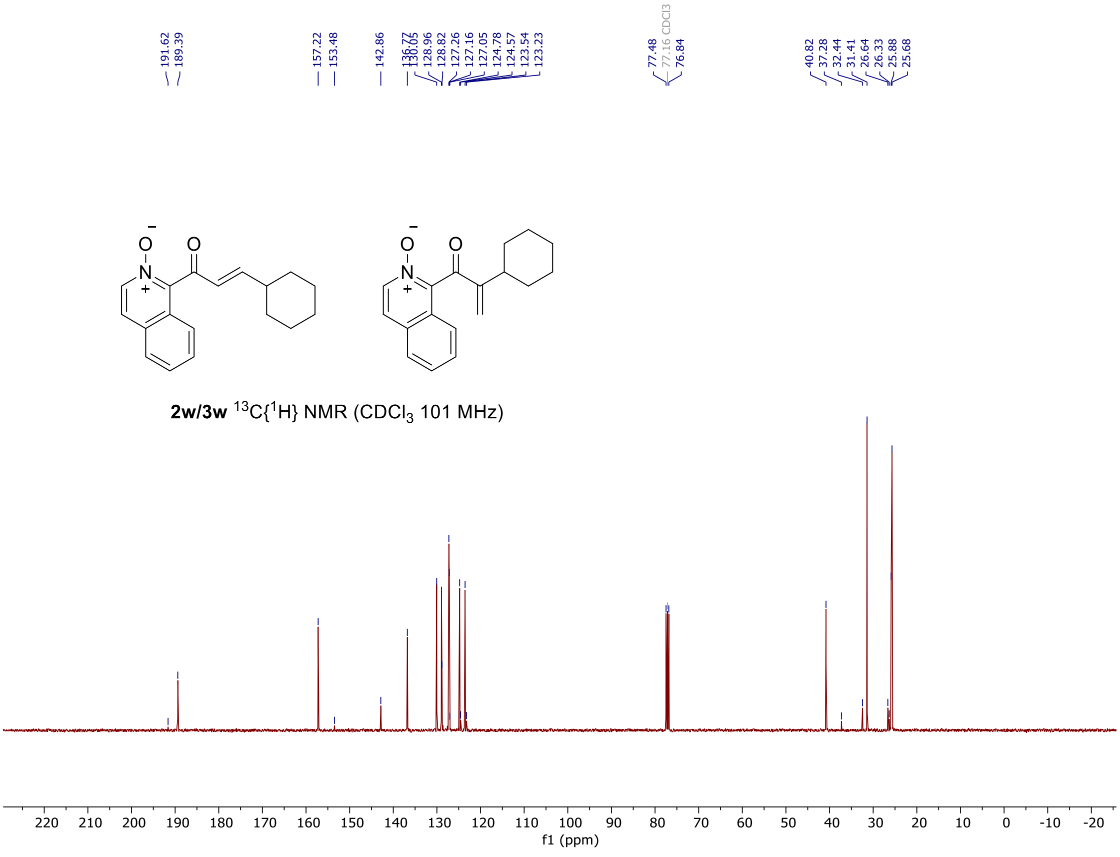

2w/3w ^{13}C{^{1}H} NMR ($CDCl_3$ 101 MHz)

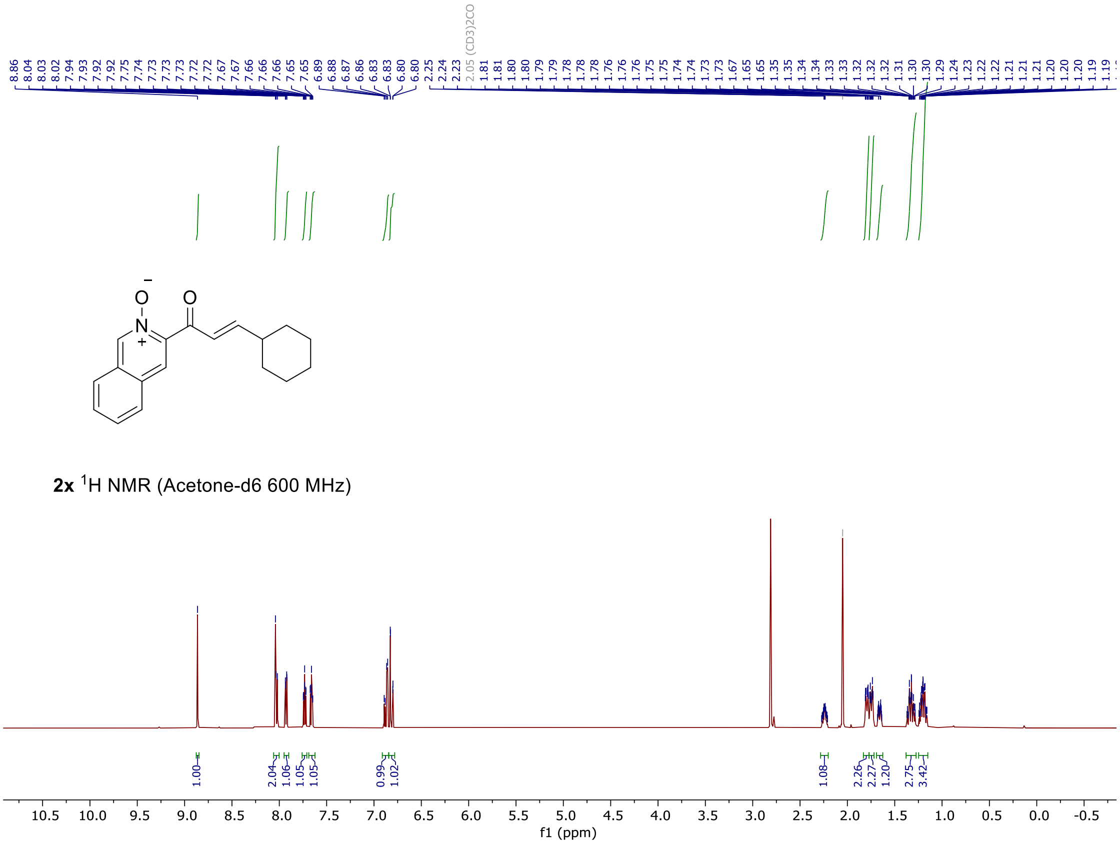

2x ^{1}H NMR (Acetone-d6 600 MHz)

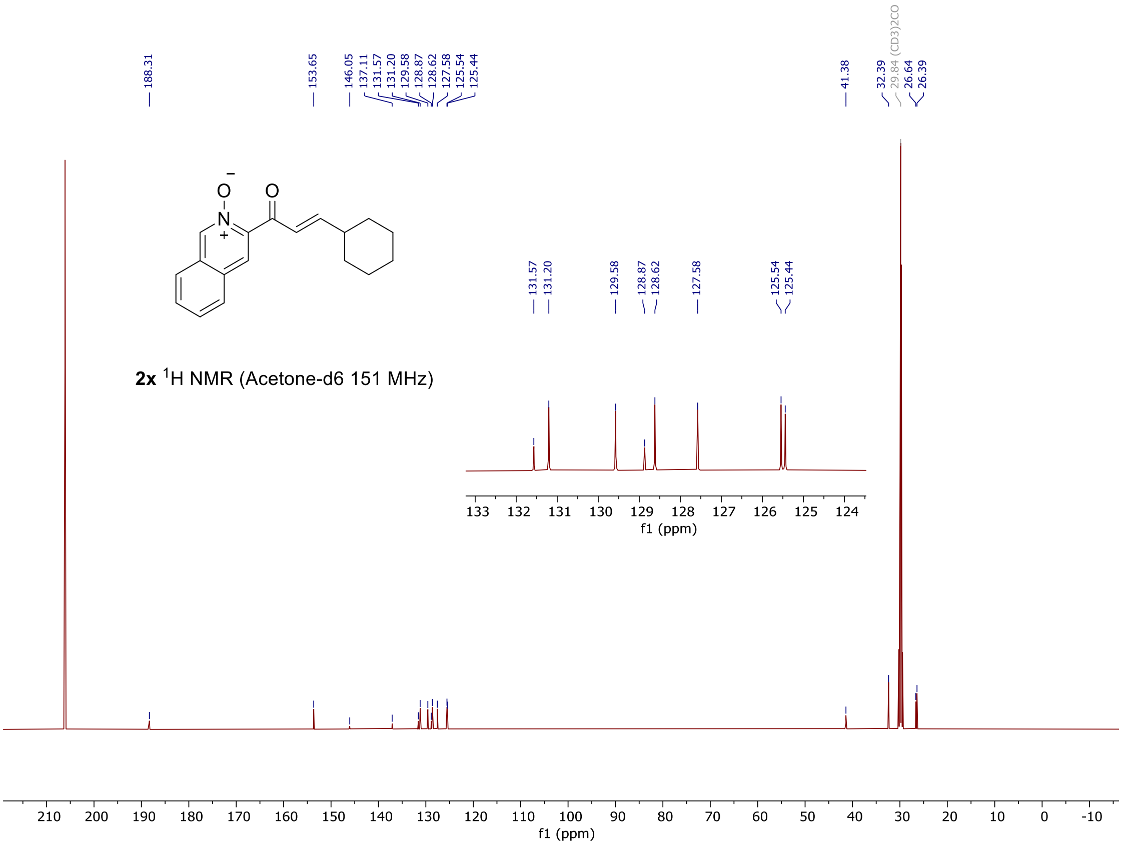

2x ^{1}H NMR (Acetone-d6 151 MHz)

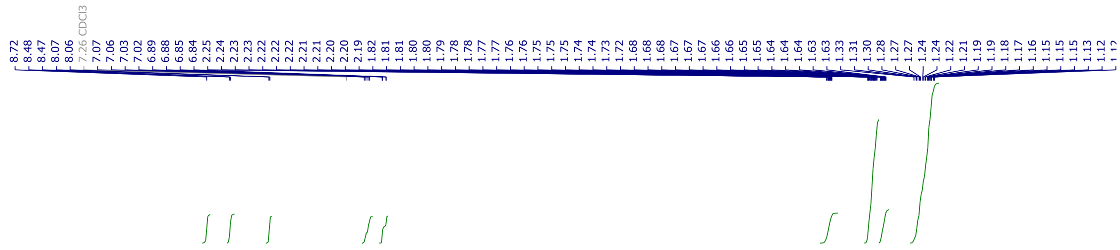

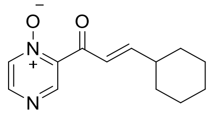

2y ^{1}H NMR ($CDCl_3$ 400 MHz)

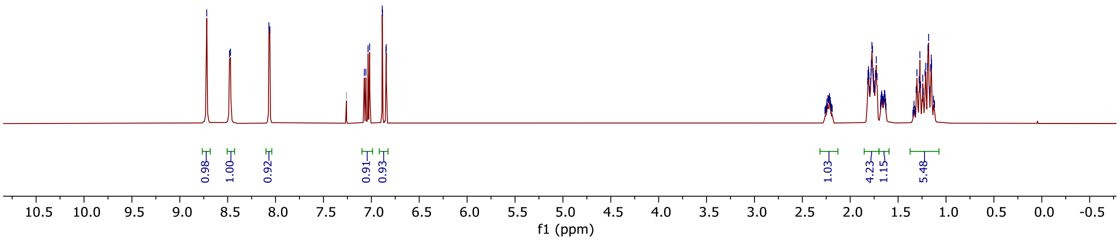

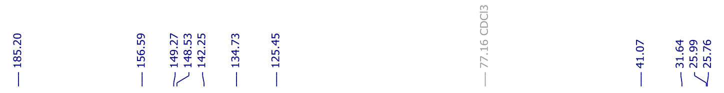

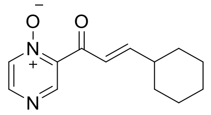

2y ^{13}C{^{1}H} NMR ($CDCl_3$ 101 MHz)

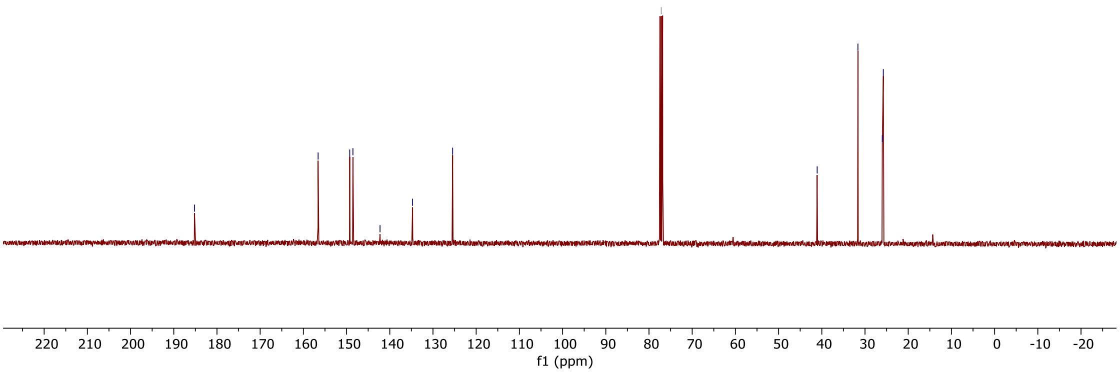

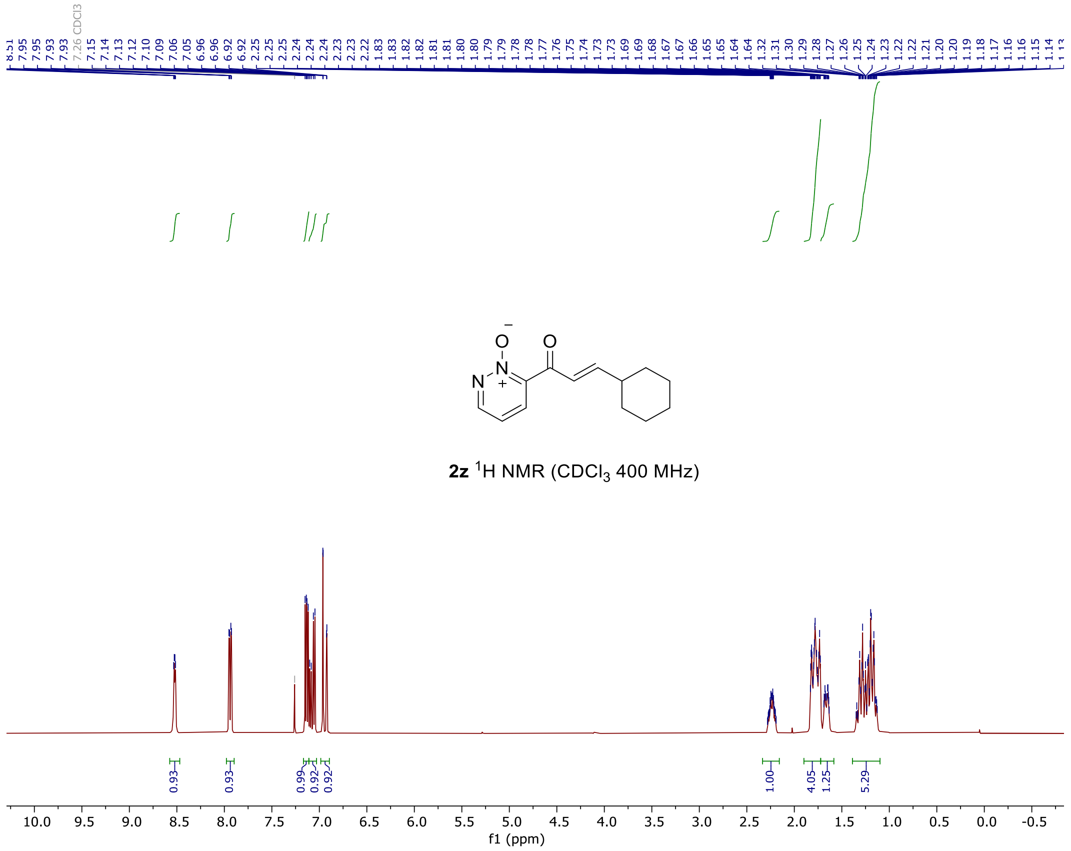

2z ^{1}H NMR ($CDCl_3$ 400 MHz)

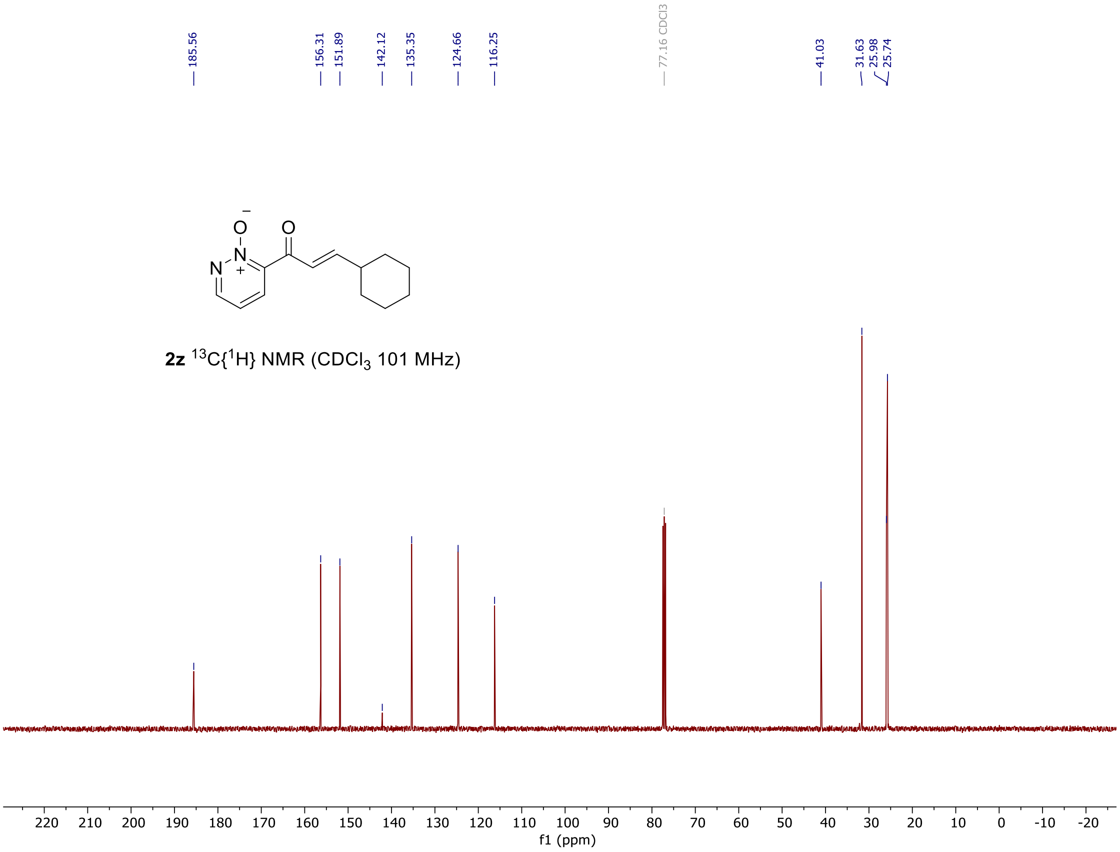

2z $^{13}C\{^{1}H\}$ NMR ($CDCl_3$ 101 MHz)

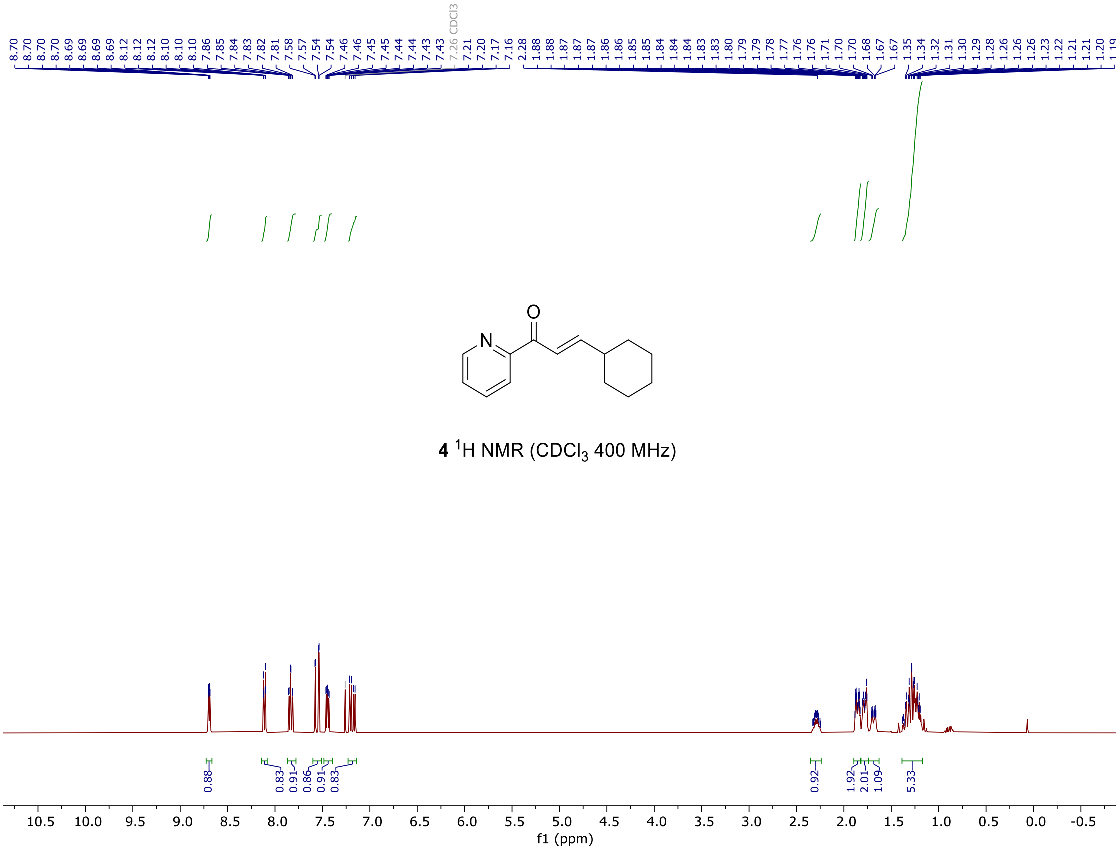

4 ^{1}H NMR ($CDCl_3$ 400 MHz)

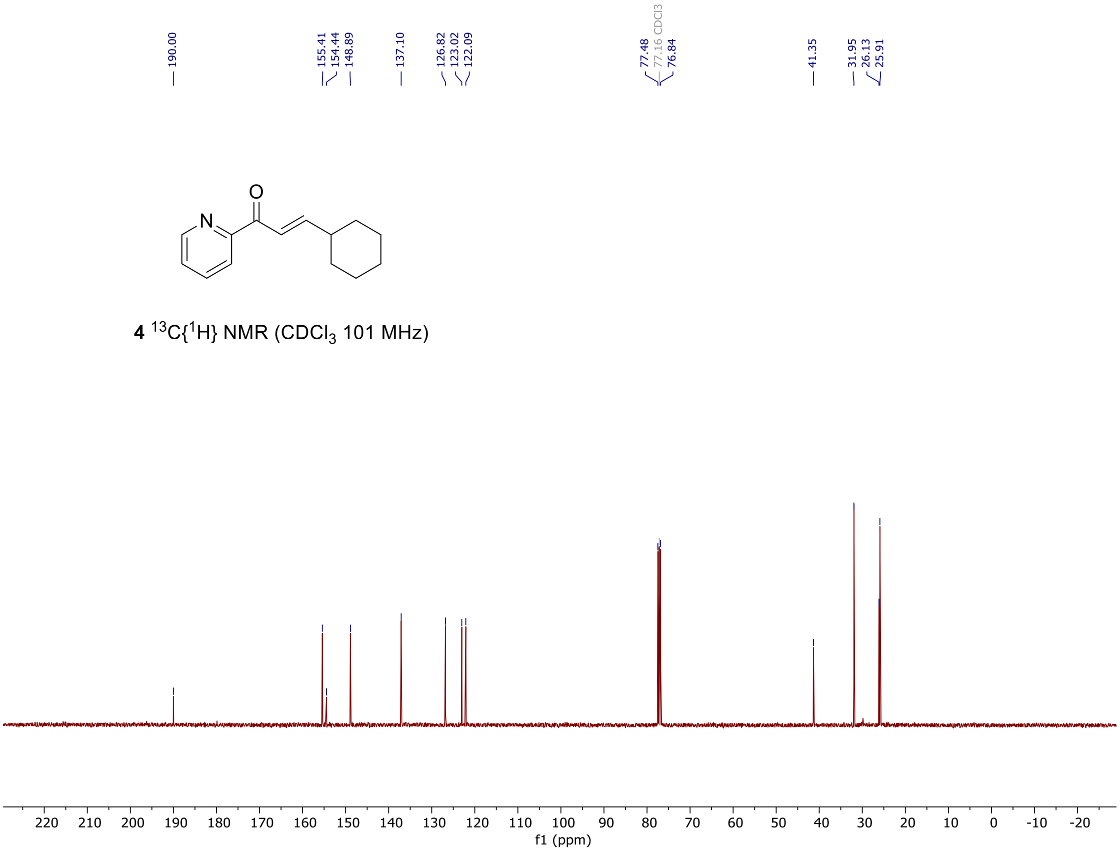

4 $^{13}C\{^{1}H\}$ NMR ($CDCl_3$ 101 MHz)

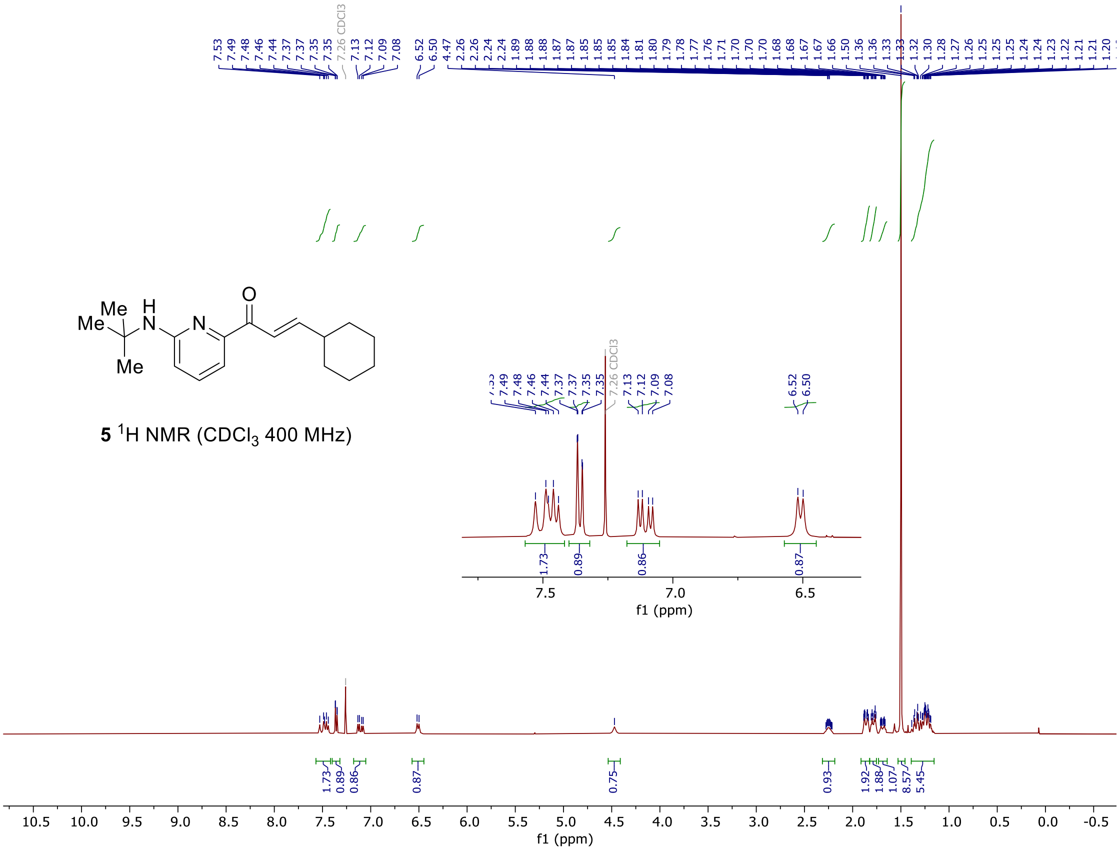

5 ^{1}H NMR ($CDCl_3$ 400 MHz)

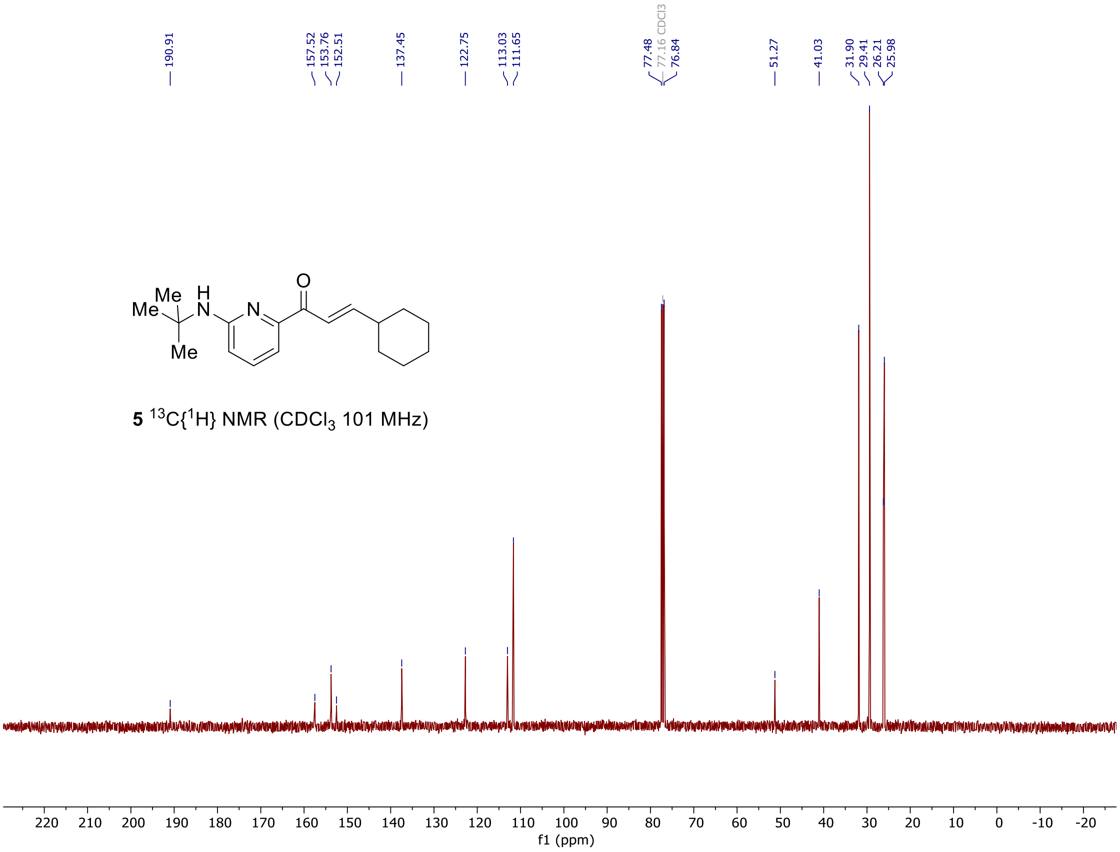

5 $^{13}C\{^{1}H\}$ NMR ($CDCl_3$ 101 MHz)

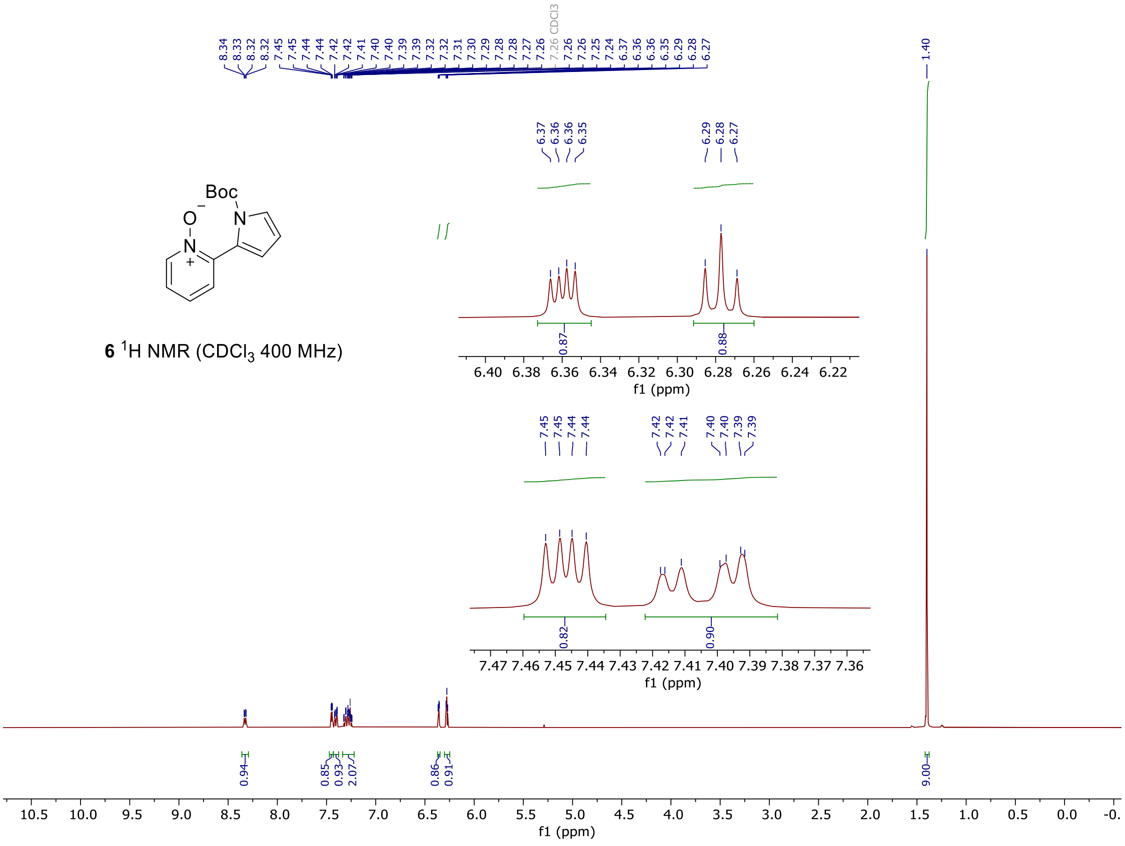

6 ^{1}H NMR ($CDCl_3$ 400 MHz)

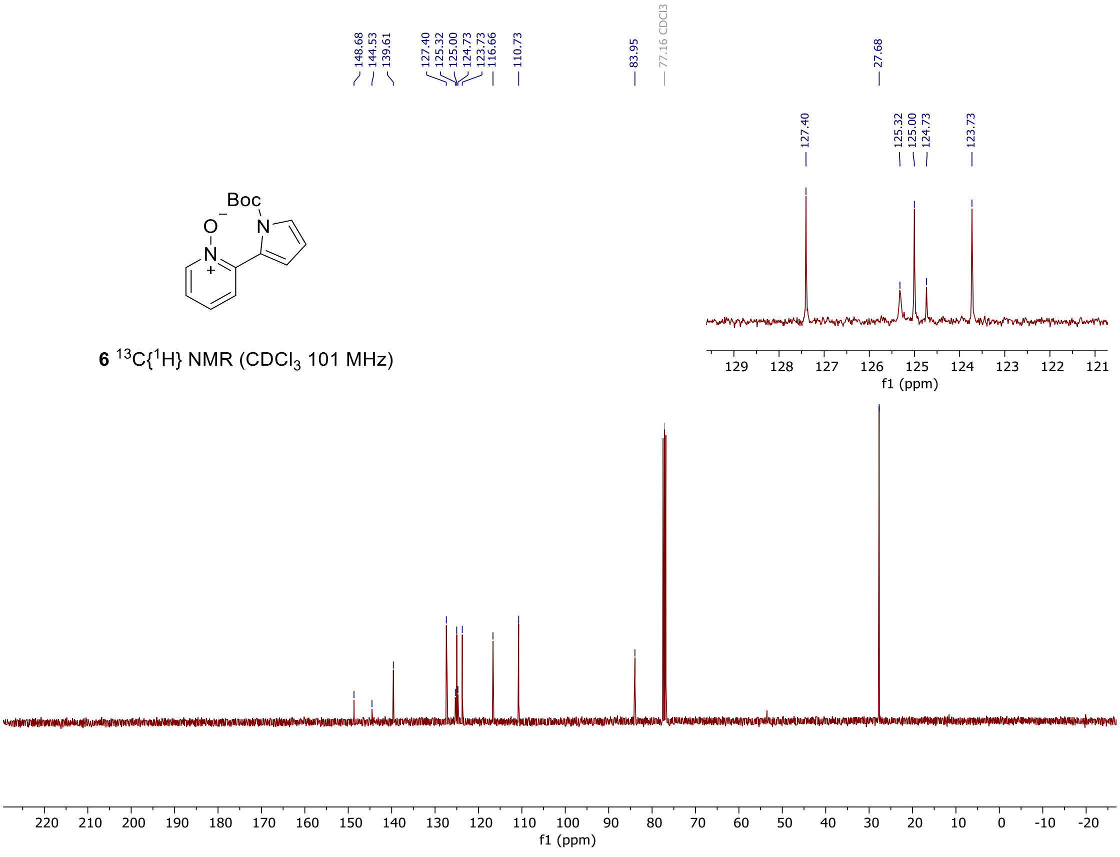

6 $^{13}C\{^{1}H\}$ NMR ($CDCl_3$ 101 MHz)
